# Carbon‐Supported Single Fe/Co/Ni Atom Catalysts for Water Oxidation: Unveiling the Dynamic Active Sites

**DOI:** 10.1002/anie.202424629

**Published:** 2025-05-06

**Authors:** Wenchao Wan, Liqun Kang, Alexander Schnegg, Olaf Ruediger, Zongkun Chen, Christopher S. Allen, Longxiang Liu, Sonia Chabbra, Serena DeBeer, Saskia Heumann

**Affiliations:** ^1^ Max Planck Institute for Chemical Energy Conversion 45470 Mülheim an der Ruhr Germany; ^2^ Electron Physical Science Imaging Center Diamond Light Source Ltd Didcot Oxfordshire OX11 0DE UK; ^3^ Department of Materials University of Oxford Parks Road Oxford OX1 3HP UK; ^4^ Department of Chemistry University College London 20 Gordon St London WC1H 0AJ UK

**Keywords:** Active site, In situ XAS, Single‐atom catalysis, Structural reconstruction, Water oxidation

## Abstract

Extensive research has been conducted on carbon‐supported single‐atom catalysts (SACs) for electrochemical applications, owing to their outstanding conductivity and high metal atom utilization. The atomic dispersion of active sites provides an ideal platform to investigate the structure–performance correlations. Despite this, the development of straightforward and scalable synthesis methods, along with the tracking of the dynamic active sites under catalytic conditions, remains a significant challenge. Herein, we introduce a biomass‐inspired coordination confinement strategy to construct a series of carbon‐supported SACs, incorporating various metal elements, such as Fe, Co, and Ni. We have systematically characterized their electronic and geometric structure using various spectroscopic and microscopic techniques. Through in situ X‐ray absorption spectroscopy (XAS), atomic scanning transmission electron microscopy (STEM), and electron paramagnetic resonance (EPR) analyses, it is demonstrated that the single atoms undergo structural rearrangement to form amorphous (oxy)hydroxide clusters during oxygen evolution reaction (OER), where the newly formed oxygen‐bridged dual metal M─O─M or M─O─M’ (M/M’ = Fe, Co, Ni) moieties within these clusters play key role in the OER performance. This work provides essential insights into tracking the actual active sites of SACs during electrochemical OER.

## Graphical abstract

Combined in situ XAS and electrochemical characterizations reveal the structural rearrangement of carbon‐supported Fe/Co/Ni single‐atom catalysts during water oxidation. Our findings highlight the formation of dual metal active sites bridged by oxygen atoms during the reaction, providing key mechanistic insights into the identification of true active sites, which are crucial for advancing future research on this class of catalysts.

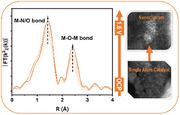

## Introduction

The continuous emission of carbon dioxide and its global environmental impact have attracted significant attention. Electrocatalytic water splitting, which aims at generating clean hydrogen to replace the current consumption of fossil fuels, stands as a vital and promising pathway toward achieving a carbon‐free energy cycle.^[^ ^1^, ^2^ ^]^ However, the overall efficiency of water splitting is severely hampered by the anodic oxygen evolution reaction (OER) due to its intrinsically slow four‐electron transfer process.^[^ ^3^, ^4^ ^]^

The recent emergence of single‐atom catalysts (SACs), featuring atomically dispersed metal atoms supported on conductive materials (such as layered carbon), has shown tremendous potential for OER and other electrocatalytic applications.^[^ ^5^, ^6^, ^7^, ^8^, ^9^, ^10^, ^11^, ^12^ ^]^ In the case of layered‐carbon supported transition metal SACs, the metal atoms are anchored to the surface of carbon matrix, forming well‐coordinated bonds with nonmetal atoms, such as N and C. The atomic dispersion of metal atoms on carbon, facilitated by the unique metal coordination structure, provides efficient atomic utilization and robust metal‐support interactions for catalytic applications.^[^ ^13^, ^14^ ^]^ Mechanistic studies on conventional OER catalysts, including alloys, oxides, and hydroxides, have revealed that nearly all catalysts undergo surface structural reconstruction during the alkaline OER, leading to the formation of new and more active phases that enhance OER performance.^[^ ^15^ ^]^ Additionally, integrating two or multiple metals, such as NiFe and CoFe, into catalysts can significantly enhance the OER performance.^[^ ^16^ ^]^ It is widely recognized that the most active phases for the OER comprise transition metal (oxy)hydroxides formed on the surface of catalysts, featuring double metal sites bridged by two oxygen atoms (M─O─M or M─O─M’, M/M’ = Fe, Co, Ni, etc.).^[^ ^17^, ^18^ ^]^ Inspired by this, recent research on SACs for OER has extended from single metal to dual metal and multiple metals.^[^ ^19^, ^20^, ^21^ ^]^

However, debates persist about how different metal atoms interact with each other, and whether they form new active phases, as observed in conventional OER catalysts during the reaction. These uncertainties stem from the lack of efficient methods to identify their atomic structures under operating conditions. A recent study on carbon‐supported Fe SAC for OER, using in situ X‐ray absorption spectroscopy (XAS) identified the active species for OER as an Fe^4+^ active site.^[^ ^22^ ^]^ However these changes may arise from structural reconstruction and not form part of the active site. Currently, most SACs for OER catalysis are described as single stable active sites.^[^ ^23^, ^24^ ^]^ A prevailing assumption in research on SACs is that catalytic reactions occur exclusively at isolated metal sites, and the local structure of the metal centers remains the same during OER. However, a few recent studies have started to reveal that the single atoms situated on carbon supports undergo structural reconstruction during catalytic reactions.^[^ ^25^, ^26^, ^27^, ^28^ ^]^ For example, in the reduction of nitrate to ammonia, carbon supported Cu SAC was found to be reduced into Cu clusters, which serves as the active species.^[^ ^28^ ^]^ This suggests a critical need to reevaluate the actual active species of this type of catalyst and understand the associated reaction mechanisms under more realistic catalytic conditions.

In this study, a straightforward and scalable coordination confinement strategy is presented using a bottom‐up approach based on cost‐effective graphitic carbon nitride (g‐C_3_N_4_), biomass‐derived tannic acid (TA) with multiple coordination sites, and metal salts as precursors. A series of N‐doped carbon‐based SACs, in the form of single element (Tan‐CN‐Fe/Co/Ni) and dual elements (Tan‐CN‐CoFe/NiFe), are constructed after a high temperature carbonization process. The atomic features of the as‐prepared catalysts are characterized by XAS and high‐angle annular dark‐field scanning transmission electron microscopy (HAADF‐STEM) studies. Among the synthesized SACs, the catalysts featuring dual metal elements (CoFe and NiFe) exhibit superior OER activity compared to those with only a single metal element. In situ XAS, combined with subsequent HAADF‐STEM and electron paramagnetic resonance (EPR) measurements, illustrates that the newly formed amorphous (oxy)hydroxides with typically oxygen‐bridging two metal atoms (M─O─M for single metal SACs or M─O─M/M’ for dual metal SACs, M/M’ = Fe, Co, Ni) represent the important active sites for the OER. This study introduces a universal and convenient coordination strategy for constructing carbon‐supported SACs, offering greater practical viability for potential applications compared to the existing top‐down strategies. Mechanistic insights into structural evolution shed light on the genuine active sites of carbon‐supported SACs during catalytic reactions.

## Results and Discussion

### Synthesis and Structural Characterizations

**Figure 1:**
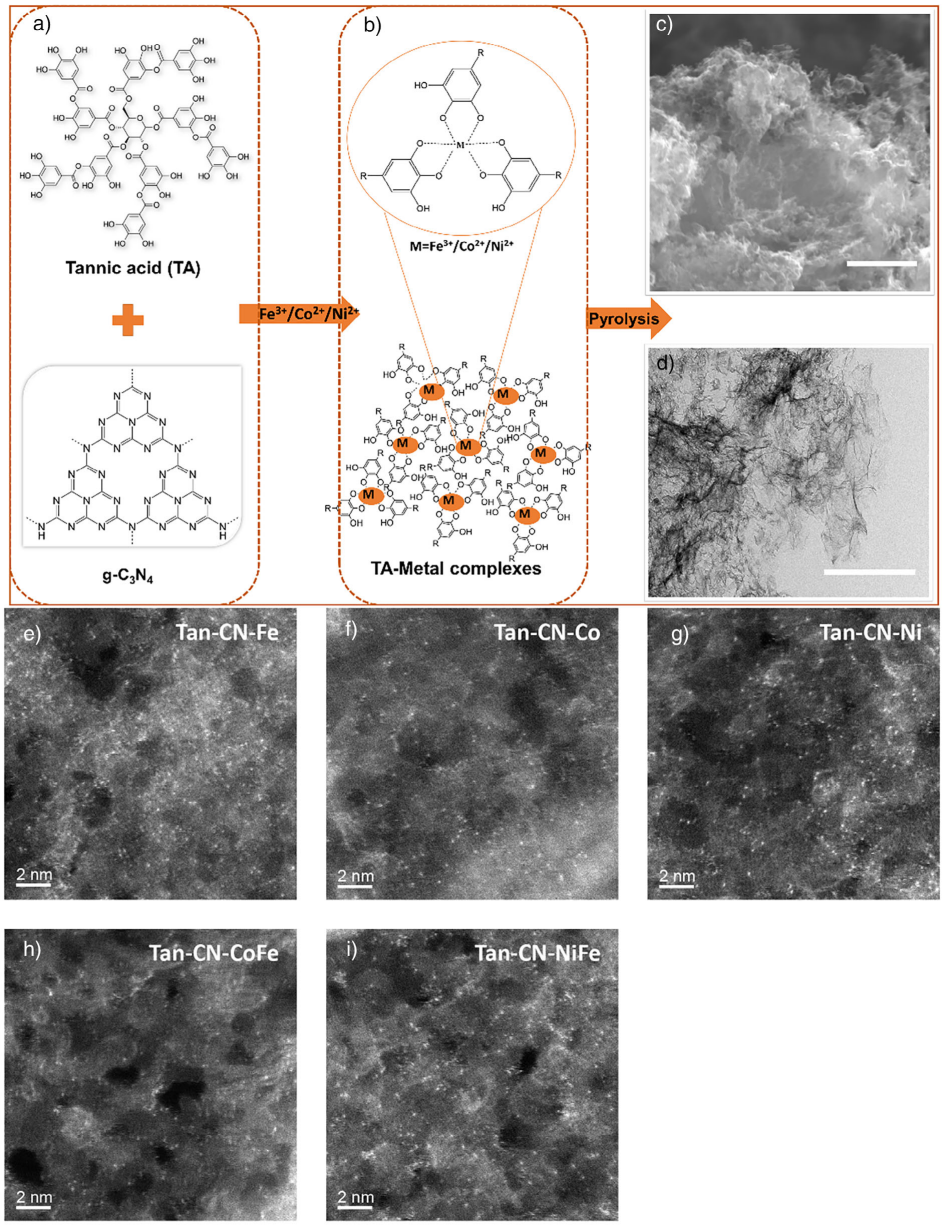
Scheme of the synthetic strategy of SACs. a) Molecular structures of tannic acid and g‐C_3_N_4_. b) Fe^3+^/Co^2+^/Ni^2+^ ions are coordinated with TA molecules. Morphological characterizations of the Tan‐CN‐Ni using c) scanning electron microscopy (SEM) and d) transmission electron microscopy (TEM) with the scale bar of 3 and 500 nm, respectively. HAADF‐STEM images of e) Tan‐CN‐Fe, f) Tan‐CN‐Co, g) Tan‐CN‐Ni, h) Tan‐CN‐CoFe, and i) Tan‐CN‐NiFe with the scale bar of 2 nm.

The carbon‐supported SACs were synthesized using a bottom‐up coordination confinement strategy, employing g‐C_3_N_4_ and TA‐coordinated metal complexes as precursors (Figure 1a,b). The atomic nature of the metal species was initially examined using conventional scanning electron microscopy (SEM), transmission electron microscopy (TEM) images, and powder X‐ray diffraction (PXRD), where no metallic particles were detected (Figures 1c,d and S1–S7). HAADF‐STEM was further conducted to directly observe the atomic dispersion of the metal atoms on the carbon substrate (Figures 1e–i and S8–S12). In these images, due to the power‐law dependence of HAADF‐STEM intensity on atomic number, heavier atomic species appear brighter. Atomically dispersed transition metals, such as Fe, Co, and Ni, are visualized as distinct bright dots on the carbon substrate. The composition and dispersion of the metals were further confirmed through energy‐dispersive X‐ray spectroscopy (EDS) measurements (Figure S13). Tan‐CN‐CoFe and Tan‐CN‐NiFe, which contain two different metal elements, exhibit similar results to single‐element samples, as seen in Figures 1 h,i, S14, and S15. The metal content for all catalysts, ranging from 1.0 to 5.0 wt.%, is determined by EDS and XRF and is presented in Table S1. The detailed synthetic procedure is provided in the Supporting Information.

**Figure 2:**
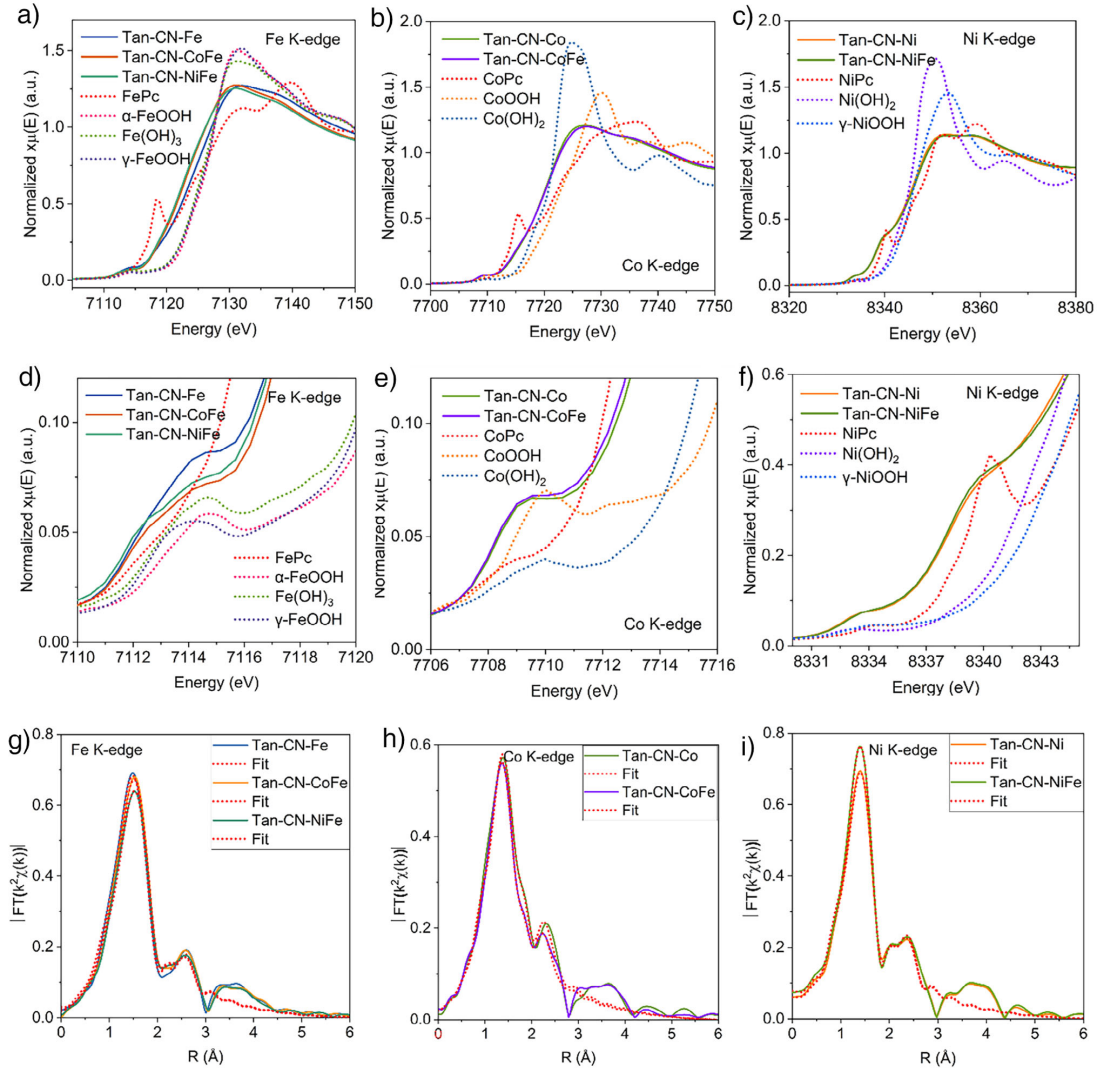
a–c) Experimental K‐edge XANES spectra of Tan‐CN‐M series along with their references and d–f) their corresponding pre‐edge and rising edge spectra. g–i) Fourier transform of the experimental K‐edge EXAFS signals of Tan‐CN‐M series.

The local structure of the SACs was further elucidated through XAS measurements. A comparison of the Fe, Co, and Ni K‐edge X‐ray absorption near edge structure (XANES) spectra of the SACs with their respective references reveals distinct differences in both the pre‐edge and rising edge regions (Figure 2a–f). Notably, all the SACs exhibit pronounced pre‐edge features due to the 1s to 3d transition, suggesting the nonmetallic valence states of the transition metal species. However, at both the Fe K‐edge and Co K‐edge of the Tan‐CN‐Fe/Co/CoFe catalysts, the rising edges are relatively featureless, unlike the corresponding phthalocyanine and (oxy)hydroxides references, suggesting that Co and Fe centers neither possess a symmetrical square planar nor octahedral configuration (Figure 2d,e).^[^ ^29^ ^]^ This is inconsistent with the widely reported square‐planar M‐N₄ configuration in the literature. However, based on XAS analysis, determining the precise coordination geometry is not feasible. It is also worth noting that XAS results represent a statistical ensemble. During high‐temperature pyrolysis, obtaining a uniform coordination structure similar to molecular catalysts is also unrealistic. In contrast, the Ni K‐edge XANES of Tan‐CN‐Ni and Tan‐CN‐NiFe display a noticeable shoulder peak on the rising edge at approximately 8340.3 eV due to the 1s to 4p_z_ transition, and both the pre‐edge and rising edge features have resemblance with those of NiPc (Figure 2f). The coordination number (C.N.) from the FT‐EXAFS fitting results are 3.8 ± 0.4 (Tan‐CN‐Ni) and 3.7 ± 0.4 (Tan‐CN‐NiFe), which may suggest a (distorted) square planar geometry for Ni. However, Fe species in Tan‐CN‐CoFe and Tan‐CN‐NiFe are slightly more reduced as their rising edges are shifted by 0.71 eV to lower energies compared to that of Tan‐CN‐Fe (Figure 2a), indicating slight reduction of Fe species in Tan‐CN‐CoFe and Tan‐CN‐NiFe. The oxidation states of the metal atoms were further characterized by X‐ray photoelectron spectroscopy (XPS), metal L‐edge XAS, and electron paramagnetic resonance (EPR) measurements (Figures S16–S18 and 4). The XPS signals indicate that Ni is predominantly in the Ni^2^⁺ state. EPR shows, all Fe‐containing samples have high‐spin (HS), Fe^3+^ (*S* = 5/2) contributions, which increase significantly during OER. The metal L‐edge XAS show Co is present as HS Co^2+^.

The coordination environment of the single metal sites was investigated using FT‐EXAFS analysis. Across the *R*‐space EXAFS spectra (without phase correction) for all samples, a consistent structural pattern was observed, showing primary coordination features at around 1.40–1.50 Å and additional features as shoulder peaks between 2.0 and 3.0 Å (Figure 2g–i). The fitting results suggest that the first coordination shell is the single scattering between metal centers and light ligand atoms (C/N/O), and not metal–metal bonds, which would be at longer distances (Figures S19–S25 and Table S2). The analysis of N 1s and O 1s XPS spectra of Tan‐CN‐M samples, compared to the nonmetal sample (Tan‐CN), suggests that the first coordination shell is most likely comprised of M─N/O bond. This is inferred from the notable decrease in the pyrrolic nitrogen signals and alterations in oxygen signals, with minimal changes observed in carbon signals after metal integration (Figure S26). It is worth noting that due to the close atomic number of N and O, XAS is not able to distinguish M─N and M─O bonds. The shoulder peak in the range of 2.0–3.0 Å could arise from either M─M or M─N/O/C paths. To gain further insights, wavelet transform (WT) EXAFS spectra for all the samples and their respective references were analyzed (Figures S27 and S28). WT‐EXAFS spectra of the reference compounds (metal oxides and metal phthalocyanines) typically show nonmetal M─N and M─O coordination features at around *k* = 4 Å^−1^, whereas M─M paths in metal and metal oxides are observed at approximately *k* = 7–8 Å^−1^ (Figure S27). In our Tan‐CN‐M SACs, only a single intensity maximum was observed at about 4 Å^−1^ in the second coordination shell, leading to the conclusion that the metal atoms in the catalysts are primarily coordinated with nonmetal N/O/C atoms in the second coordination shells (up to 3 Å). This finding is consistent with the fitting results (Figures S19–S25), where the first coordination shell was effectively fitted with the M─N/O path, and the shoulder peaks in the second coordination shell were attributed to M─C paths (Table S2).

### Electrocatalytic OER Characterization

**Figure 3:**
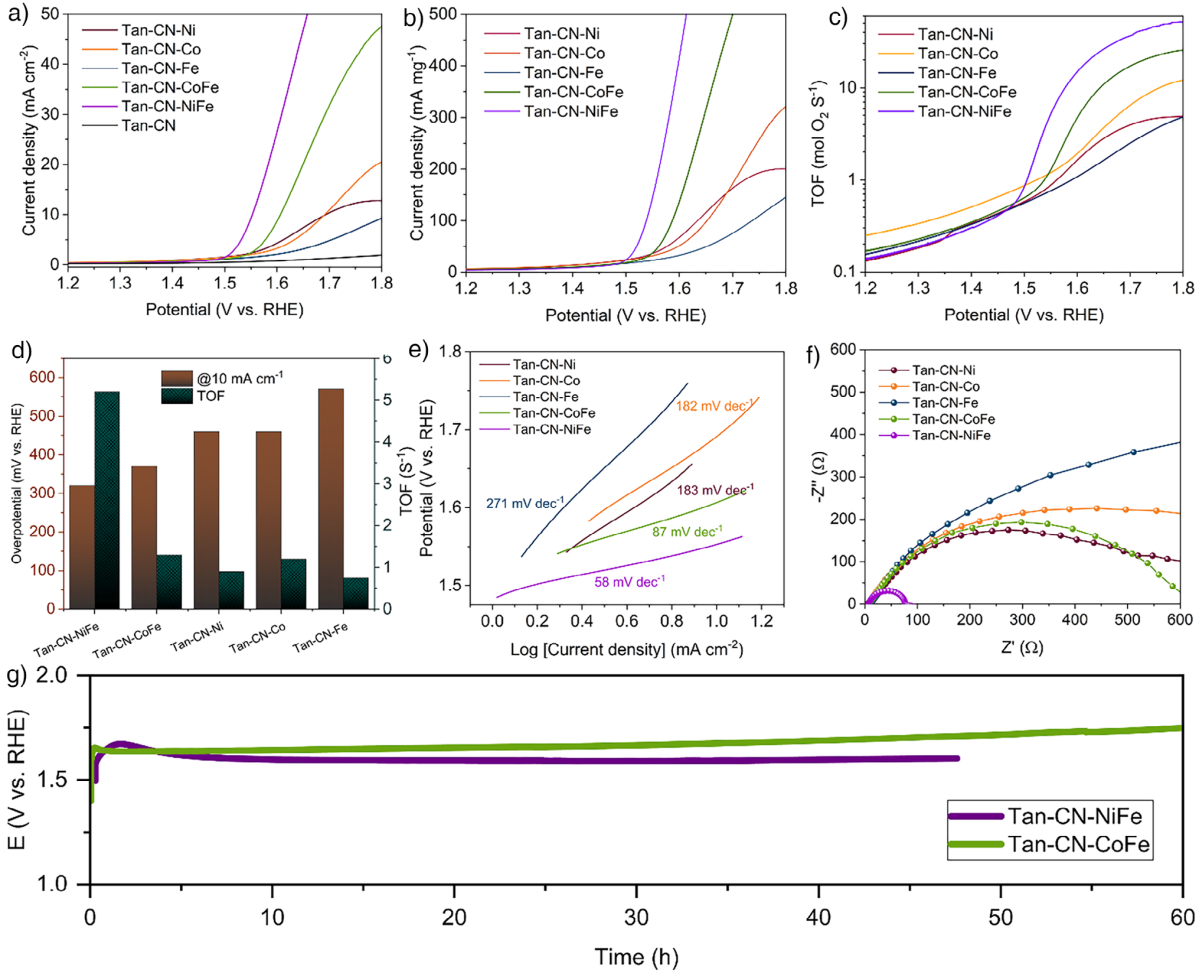
a) LSV curves with a scan rate of 10 mV s^−1^, b) corresponding mass activities, and c) TOF curves of the as‐prepared catalysts. d) Overpotentials and TOFs at the current density of 10 mA cm^−1^. e) Tafel slopes. f) EIS curves at 1.5 V versus RHE. g) Chronopotentiometry measurements of CoFe and NiFe SACs at 10 mA cm^−2^ on carbon cloth.

The OER performance of the catalysts was recorded in 1 M KOH on both glassy carbon (GC) and carbon cloth (CC) electrodes. All the linear sweep voltammetry (LSV) curves were acquired after 10 cyclic voltammetry (CV) cycles to activate the samples (Figures S29 and S30). The dual‐metal Tan‐CN‐CoFe and Tan‐CN‐NiFe SACs show significantly improved OER performance, compared to the single metal Fe/Co/Ni SACs, with overpotentials of 370 and 320 mV versus RHE, respectively, at the current density of 10 mA cm^−2^ (Figure 3a). The mass activity of Tan‐CN‐NiFe shows that only 1.6 V versus RHE is required to achieve 500 mA mg^−1^(Figure 3b). Furthermore, the turnover frequency (TOF) is as high as 5.2 s^−1^ (Figure 3c,d), which outperforms recently reported SACs for OER (Table S4).^[^ ^30^ ^]^ The corresponding Tafel slopes are only 87 and 58 mV dec^−1^, respectively, significantly smaller than those of single‐metal Fe/Co/Ni SACs (Figure 3e). The enhanced kinetics of NiFe SACs are further evidenced from the electrochemical impedance spectroscopy (EIS) measurements at OCP and 1.5 V versus RHE (Figures 3f and S31), where the charge‐transfer resistances are dramatically reduced (Figure 3f). The stabilities of CoFe and NiFe SACs were further assessed on CC through chronopotentiometry measurements, in which only 6.7% drop in voltage was observed for Tan‐CN‐CoFe over 50 h at a current density of 10 mA cm^−2^, and the change of Tan‐CN‐NiFe is negligible (Figure 3g). It is worth noting that the sharp increase in overpotential at the beginning may relate to the carbon corrosion process.^[^ ^31^ ^]^

The performances of the catalysts were also tested in purified KOH.^[^ ^32^, ^33^ ^]^ (Figures S32 and S33). Interestingly, we observed that the performance of monometallic Co/Ni catalysts gradually declined during CV testing in purified KOH, whereas their performance remained relatively stable in nonpurified commercial electrolyte. In contrast, the bimetallic CoFe and NiFe catalysts maintained relatively stable performance, regardless of whether the KOH electrolyte was purified or not. These results highlight the significant role that trace Fe impurities in the electrolyte play in affecting the activity and stability of non‐Fe‐based catalysts. Due to the limited amount of Fe impurities in the commercial electrolyte, the OER performance of monometallic Co/Ni catalysts remains lower than that of the bimetallic CoFe and NiFe catalysts.

The OER performances of physical mixtures of Tan‐CN‐Fe with Tan‐CN‐Co and Tan‐CN‐Ni were also investigated. However, significant enhancements in OER performances were not observed (Figure S32d,e). The enhanced OER performance of bimetallic SACs indicate a positive synergistic effect between Fe and Ni or Co species in OER. However, if all metal centers remain atomically dispersed during OER, such synergistic effect should not be expected. It is reasonable to believe that Fe and Ni/Co may form new species during the OER process. The combination of Fe and Co/Ni to enhance the OER performance has been widely investigated in Fe/Co/Ni‐based oxide and hydroxide;^[^ ^34^, ^35^ ^]^ however, its application in the realm of SACs has not been as widely recognized. In traditional oxide and hydroxide catalysts, the active phases typically consist of Fe‐O‐Ni and Fe‐O‐Co moieties, typically bridged by two oxygen atoms with the two metal atoms spaced approximately 2.80–3.20 Å.^[^ ^34^ ^]^ However, our XAS analysis of the material before catalysis revealed only the presence of M─N/O coordination sphere with no evidence for the M–M interactions that would be expected if such bimetallic active sites were formed. This indicates that the local structures of metal atoms may undergo structure rearrangements to form new active species during OER, which may account for the enhanced performance in CoFe and NiFe SACs.

### Identification of Actual Active Species for OER

**Figure 4:**
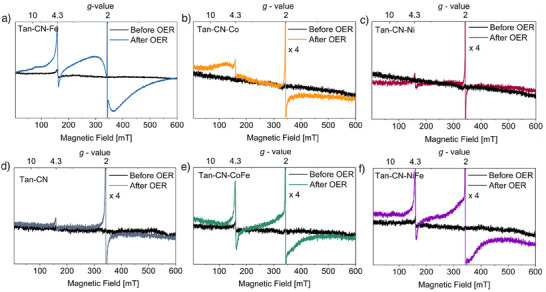
CW X‐band EPR spectra of a) Tan‐CN‐Fe, b) Tan‐CN‐Co, c) Tan‐CN‐Ni, d) nonmetal containing Tan‐CN, e) Tan‐CN‐CoFe, and f) Tan‐CN‐NiFe before and after OER (spectra in (b)–(f) are multiplied by a factor of four relative to Tan‐CN‐ Fe (a)). To highlight the spectral contributions of Fe and Co, the narrow signal at *g* = 2 is clipped. Full spectra are shown in Figure S34. Measurement parameters: *T* = 10 K, MW frequency: 9.64 GHz, MW power: 2 mW, modulation amplitude: 0.7 mT.

To validate our hypothesis and investigate the actual active sites of the as‐prepared SACs, EPR spectroscopy was employed to gain information on the changes of the catalysts before and after OER in oxidation/spin states and coordination structures (Figures 4 and S34). EPR spectra of the precursors Tan‐CN‐Fe‐P, Tan‐CN‐Co‐P, and Tan‐CN‐Ni‐P are shown in Figure S35. The EPR of Tan‐CN‐Fe‐P is dominated by an intense broad EPR peak, with a zero‐crossing point at *g* = 4.3, a shoulder at *g* = 9.6, and a narrow signal at *g *= 2.0. The broad signal is assigned to HS Fe^3+^ (*S* = 5/2) with rhombic zero field splitting (ZFS). The spectral shape is in accordance with a Fe^3+^ site coordinated by 6 oxygen atoms.^[^ ^36^ ^]^ The narrow signal at *g* = 2.0 most likely originates from defects in the carbon matrix like, e.g., carbon dangling bonds. The EPR spectrum of Tan‐CN‐Ni‐P precursor exhibits a similar spectral shape as the Fe precursor, though with a very much reduced Fe^3+^ component, which may result from Fe impurities in the Ni samples (it is worth noting that EPR is very sensitive to rhombic HS Fe^3+^). The EPR spectrum of the Tan‐CN‐Co‐P precursor shows a broad signal with a peak at *g* ∼ 5.0, which corresponds to the typical signature of HS Co^2+^(*S *= 3/2). EPR spectra of the as‐prepared catalysts, each before and after OER, are depicted in Figure 4. Before OER, the EPR spectra of the iron containing samples (Tan‐CN‐Fe, Tan‐CN‐CoFe, and Tan‐CN‐NiFe) exhibit a weak contribution of HS Fe^3+^ at *g* = 4.3. This signal is more pronounced in Tan‐CN‐Fe as compared to Tan‐CN‐CoFe and Tan‐CN‐NiFe. The line is characteristic for HS ferric iron, with rhombic ZFS. It is not in accordance with a ferric FeN_4_ site, for which an axial ZFS with a maximum of the EPR absorption at *g* = 6 would be expected.^[^ ^37^, ^38^ ^]^ EPR spectra of the Co containing catalysts (Tan‐CN‐Co, Tan‐CN‐CoFe) before OER, show weak EPR signals extending over the whole magnetic field range (Figure S36). These signals are assigned to high‐spin (*S* = 3/2) Co^2+^. The oxidation state of Co^2+^ has been confirmed by both XPS and metal L‐edge XAS (Figure S18). Possible reasons for the weak Co^2+^ EPR are either a very pronounced EPR line broadening due to fast relaxation of the Co^2+^ electron spins or a large negative ZFS, which can render ground‐state spin transitions at liquid helium temperatures EPR‐forbidden and thus make the detectable EPR spectra weak.^[^ ^39^ ^]^ After OER, the Fe^3+^ high‐spin EPR signal is significantly enhanced in Tan‐CN‐Fe (Figure 4a), Tan‐CN‐CoFe, and Tan‐CN‐NiFe and even the EPR spectra of Tan‐CN‐Co and Tan‐CN‐Ni show small Fe^3+^ impurities (probably from KOH). In Tan‐CN‐Co after OER, an additional broad component appears between *g* = 9.0 and 5.0 (see orange trace of Figure 4b), which is assigned to high spin (*S* = 3/2) Co^2+^. In addition, pronounced changes are observed in the region around *g *= 2.0, where a very intense narrow signal (width ∼ 0.4 mT FWHM) from defects in the carbon matrix is observed. The strong increase of defects indicates degradation of the carbon matrix during OER. In Tan‐CN‐Fe, Tan‐CN‐CoFe, and Tan‐CN‐NiFe, an additional broad signal (width ∼50–100 mT) is observed around *g* = 2.0. The observed EPR line shape of a well resolved contribution between *g* = 9.6 and *g* = 4.3 and a broad signal centered at *g* = 2.0 is characteristic for HS Fe^3+^ in oxygenic coordination environments, such as layered double hydroxides,^[^ ^40^ ^]^ iron containing glasses,^[^ ^41^, ^42^, ^43^, ^44^ ^]^ and zeolites.^[^ ^45^, ^46^ ^]^ The observed changes in the EPR line shape and intensity suggest the postulated formation of transition metal (oxy)hydroxides during OER.

**Figure 5:**
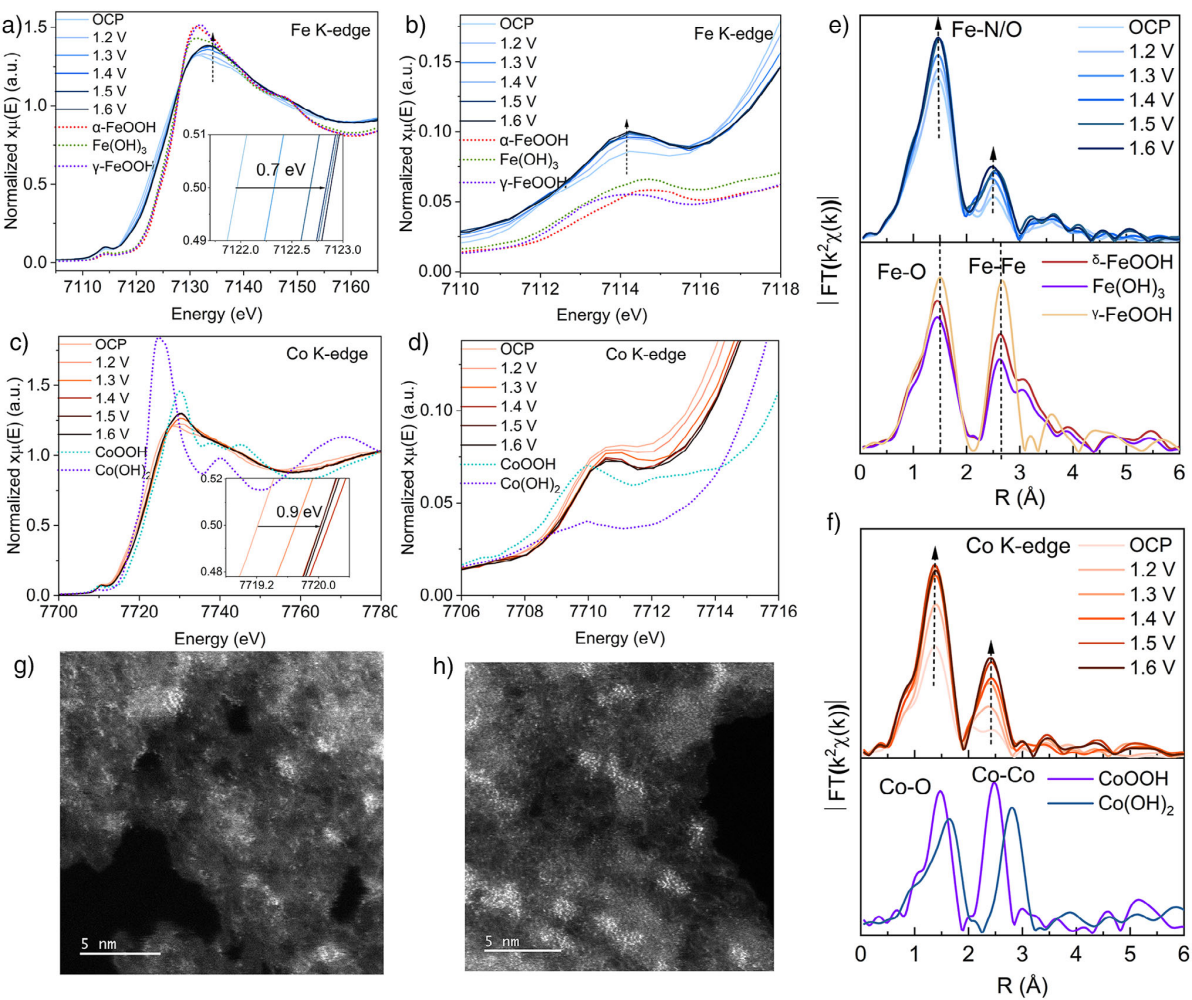
Experimental Co and Fe K‐edge a) and c) XANES spectra, b) and d) pre‐edge spectra, and e) and f) Fourier transform EXAFS signals of Tan‐CN‐CoFe. g) and h) HAADF‐STEM images of Tan‐CN‐CoFe after in situ XAS measurements.

To further substantiate this hypothesis, operando XAS experiments were conducted under electrocatalytic conditions. The details of the experiment are provided in Supporting Information (Figure S37). Prior to measurements, all electrodes were subjected to 10 CV cycles for activation. The measurements were initiated from the open circuit potential (OCP) and then progressively increased to 1.7 V versus RHE. All XANES spectra displayed distinct isosbestic points, indicating structural rearrangements during the OER (Figures 5a,c and S38–S41).^[^ ^47^ ^]^ However, the observed changes in the samples containing different metals differed subtly among the samples containing different metals. In the Fe K‐edge XANES spectra of Tan‐CN‐CoFe, an increase in the pre‐edge at around 7114 eV suggests distortion of the coordination geometry in the first coordination shell or formation of completely new species (Figure 5b).^[^ ^48^, ^49^ ^]^ The enhancement of the pre‐edge feature and the white line (indicated by the dotted arrow) in Tan‐CN‐CoFe signifies the alteration of the electronic configuration of the Fe 3d orbitals, as well as the evolution of the geometric structure and coordination environment. This could possibly be attributed to the adsorption of *OH/H_2_O at the Fe centers during the OER.^[^ ^29^ ^]^ Changes are also observed in the pre‐edge and white line of the Co K‐edge XANES, indicating comparable structural modifications at the Co sites (Figure 5b,d). Notably, the rising edge in both the Fe and Co K‐edge XANES at 1.7 V during OER are shifted about 0.9 eV to higher energy compared to the XANES collected at OCP (inset of Figure 5a,c), implying the increased average valence states for both Fe and Co during the OER.

In the *R*‐space FT‐EXAFS spectra of Tan‐CN‐CoFe, a notable trend was observed at both Fe K‐edge and Co K‐edge, showing increased peak intensity within the first coordination and second coordination shell as the applied potential was increased (Figure 5e,f). Such structural modifications are more notably displayed in the corresponding WT‐EXAFS spectra (Figure S42) The fitting results of the EXAFS spectra at 1.6 V align closely with the structure of FeOOH and CoOOH (Tables S3 and S6), which are stable species in 1 M KOH solution. For the Fe K‐edge FT‐EXAFS spectra (Figure 5e), the C.N. of the first shell (Fe‐N/O) stayed around 4.0 for all applied potentials. However, the increase of intensity together with decrease of the Debye–Waller from 0.0104 to 0.0087 indicates the local structure became more ordered. Giving that these changes are within fitting error, they may be attributed to disorder around metal atoms. There was a significant increase in the C.N. of Fe─Fe/Co path from 0.9 ± 0.4 to 2.3 ± 0.9, indicating the migration and aggregation of the Fe and Co species during the OER to form more Fe─Fe/Co moieties (dimer, trimer, and clusters). The distance of Fe─Fe/Co is about 2.98 Å, which is shorter than the Fe─Fe distance (3.03 Å) in Tan‐CN‐Fe at 1.6 V, but longer than the distance of Co─Co in Tan‐CN‐Co (2.84 Å) at 1.6 V. This further suggests the formation of Fe─O─Co moieties in Tan‐CN‐CoFe. The Co K‐edge shows similar changes with increased C.N. for Co─Co/Fe path, rising from 1.0 ± 0.3 to 2.3 ± 0.4 (Figure 5f). The distance of Co─Co/Fe in Tan‐CN‐CoFe does not change significantly compared to that in Tan‐CN‐Co, likely due to the high Co content (1.63 wt.%), which is approximately twice as much as Fe (0.91 wt.%) in the catalyst (Tables S1 and S3). Therefore, the formation of Fe─O─Co moieties is not sufficient to cause significant changes in bond lengths. The in situ XAS of Tan‐CN‐Fe and Tan‐CN‐Co show very similar changes (Figures S39 and S40). Overall, the XANES, pre‐edge and EXAFS analyses suggest that Co and Fe atoms form similar structures to their oxyhydroxides (CoOOH and FeOOH), and the formation of Co‐O‐Fe moiety is the key active species for OER. In addition, the increase of oxidation state of both Co and Fe atoms observed in XANES suggest that both metals may participate in the OER.

For Tan‐CN‐NiFe, the Fe K‐edge XANES spectra display similar changes to those of Tan‐CN‐CoFe. Positive shifts in the rising edges and the increase of the white line intensities indicate an increase of oxidation state and formation of new phases (Figure S38a). However, distinct changes are observed in the Ni K‐edge XANES of Tan‐CN‐NiFe (Figure S38c). Weak pre‐edges, attributed to the 1s to 3d transition, are observed in all spectra. In addition, a shoulder in the rising edge attributed to the 1s to 4p transition are also observed, which serves as a fingerprint for a square‐planar metal‐N_4_ moiety, suggesting the persistence of a similar unsaturated coordination structure to the NiPc reference during the OER.^[^ ^50^ ^]^ Significant changes in the Ni K‐edge FT‐EXAFS spectra of Tan‐CN‐NiFe are seen (Figure S38e). The CN of the first coordination shell increases from 5.0 to 5.8 as the potential increases, which suggests the formation of the mixture of single Ni site and (oxy)hydroxides after activation (20 CVs), and further conversion of single Ni sites to (oxy)hydrixides at 1.6 V (Table S3). Furthermore, two Ni─O─Ni/Fe bonds with the bond lengths of 2.75 and 3.07 Å are observed when the applied potential is up to 1.4 V versus RHE. These two Ni─O─Ni bonds also appears in Tan‐CN‐Ni at 1.5 V, which implies that two new species were formed during the OER. The fitting results demonstrate the coexistence of NiOOH and Ni(OH)_2_ (Figure S38e and Table S6). In the Fe K‐edge FT‐EXAFS spectra of Tan‐CN‐NiFe, the increase in the intensities in both coordination shells may result from structural orders under applied potentials. However, the signal at 2.50 Å (corresponding to the actual Fe─Fe/Co distance of 2.98 Å) in Tan‐CN‐CoFe is shorter than Fe─Fe/Ni distance at 2.75 Å (corresponds to the actual length of 3.09 Å) in Tan‐CN‐NiFe. This suggests that Fe atoms prefer to bond with Ni atoms to form a longer Fe─O─Ni bond (Table S6). This preference is likely, given that the bond length of Fe─O─Fe in FeOOH is similar to the bond length of Ni─O─Ni in Ni(OH)_2_ (Figure S38d and Table S6).^[^ ^51^ ^]^ The structural evolution is also evident from the WT‐EXAFS spectra (Figure S43). It is worth noting that the C.N. of M─M bonds in the SACs is generally small, between 2.0 and 3.0 (Table S3), suggesting that most the clusters are small and consist of only a few atoms.

The energy shifts of the K‐edge XANES for single Fe/Co/Ni metal catalysts are similar to those of the dual metal catalysts, indicating the metal atoms underwent similar oxidation changes (Figures S39–S41). The coordination numbers of the first shell in all single Fe/Co/Ni catalysts show negligible changes, which closely resembles the behaviors of their corresponding hybrid catalysts. Most changes in the K‐edge FT‐EXAFS spectra for single Fe/Co/Ni are also very similar to these of Tan‐CN‐CoFe and Tan‐CN‐NiFe. However, the Fe species in Tan‐CN‐CoFe appears to undergo reconstruction more readily with its coordination number changed from 0.9 to 2.3, compared to the changes from 0.9 to 1.6 in Tan‐CN‐Fe and from 1.6 to 1.5 in Tan‐CN‐NiFe, respectively. Two Ni species that are similar to the Ni(OH)₂ and NiOOH clusters are observed in Tan‐CN‐Ni. In contrast, only the CoOOH cluster is present in Tan‐CN‐Co, as determined by fitting (Figure S40 and Table S3). The reconstruction process is also interpreted through WT‐EXAFS spectra of Tan‐CN‐Fe/Co/Ni at different operation potentials (Figure S44). All fitting results and reference compounds are present in Tables S3 and S6 and Figures S45–S83.

**Figure 6:**
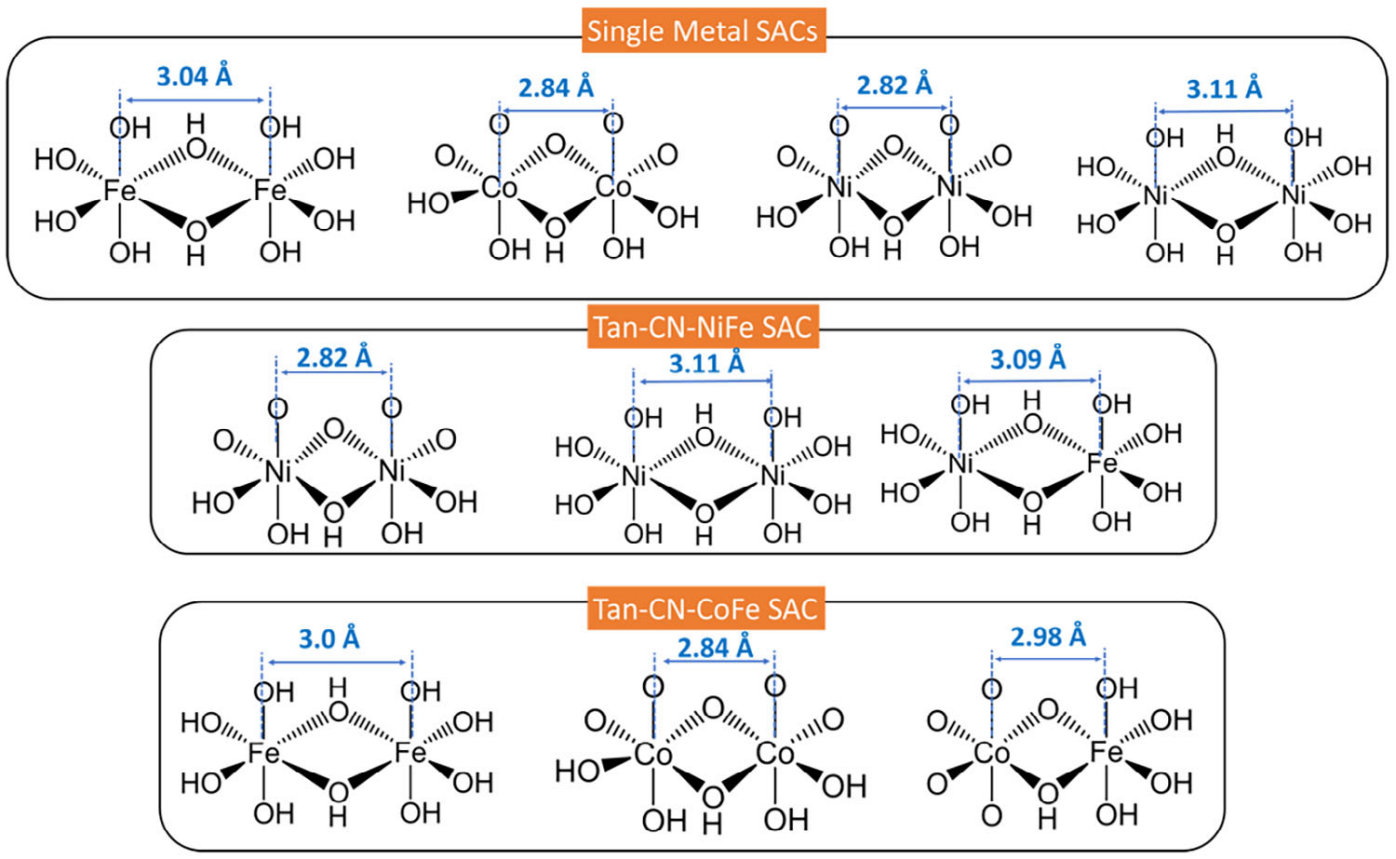
Newly formed dual metal active sites with different distances in the SACs.

The migration and aggregation of the metal species on the carbon support after the OER are also evidenced from the HAADF‐STEM images (Figures 5g–h and S84–S87). It is worth mentioning that the newly formed M─O─M (M = Fe, Co and Ni) moieties may not be the only active sites for OER as a considerable amount of isolated metal sites remain on the carbon support based on the HAADF‐STEM results. Raman spectra of the electrodes after the in situ XAS measurements show no substantial hydroxide or oxyhydroxide phases. Only catalysts containing Co elements exhibit minimal Co─O signals, which potentially originate from the CoOOH‐like phase (Figure S88).^[^ ^52^ ^]^ The absence of Fe and Ni (oxy)hydroxide signals may be related to the small size and heterogeneity of newly formed clusters. In situ XAS shows that Co tends to form CoOOH more easily, even at lower voltages. Additionally, the coordination numbers indicate that the M─O─M bonds are limited in all SACs, with cluster sizes consisting of only a few atoms. This may also contribute to the difficulty in detecting distinct hydroxide signals. It is also plausible that the M─O─M moieties may be embedded into the carbon matrix. Regardless of the exact nature of these metal moieties, it is clear that at least some of the single Fe/Co/Ni sites in the carbon structure transformed into M─O─M structures during the OER. When comparing the metal species in all the catalysts (Table 1), we observe that while all catalysts contain single metal sites and M─O─M sites, only Tan‐CN‐CoFe and Tan‐CN‐NiFe, which contain Fe─O─Co and Fe─O─Ni sites, demonstrate decent OER performance. Therefore, it is reasonable to deduce that the enhanced OER performance in dual metal SACs is attributed to these Fe─O─Co/Ni moieties in the newly formed clusters. In single metal SACs, Fe atoms prefer to form FeOOH clusters with the Fe─O─Fe bond length of about above 3.0 Å. Co is more inclined to form the CoOOH clusters with the Co─O─Co bond length of about 2.84 Å. The situation with Ni is more complex as two species that are similar to both NiOOH and Ni(OH)_2_ are present. In the dual metal SACs, in addition to the previously mentioned single‐metal dual active sites, Fe─O─Ni and Fe─O─Co bonds were also formed, with bond lengths of 3.09 and 2.98 Å, respectively. The newly formed M─O─M sites in the SACs are summarized in Figure 6 and Table S2. It is worth noting that, although dual sites are formed during OER, a significant number of single sites, as well as dimer and trimer clusters, are still observable under STEM.

**Table 1:** Summary of the metal sites identified in different SACs.

***Tan‐CN‐Fe***	Atomic Fe	Fe‐O‐Fe			
***Tan‐CN‐Co***	Atomic Co	Co‐O‐Co			
***Tan‐CN‐Ni***	Atomic Ni	Ni‐O‐Ni			
***Tan‐CN‐CoFe***	Atomic Fe	Atomic Co	Co‐O‐Co	Fe‐O‐Fe	Co‐O‐Fe
***Tan‐CN‐NIFe***	Atomic Fe	Atomic Ni	Ni‐O‐Ni	Fe‐O‐Fe	Ni‐O‐Fe

The formation of clusters can be attributed to the carbon corrosion during OER as evidenced by the brown coloration of the electrolyte after prolonged measurement (Figure S89). The obtained carbon catalysts comprise a significant mix of Fe/Co/Ni/N/O atoms, leading to amorphous and defective structures in the carbon substrate, which is less stable than a perfect carbon lattice. Carbon corrosion during OER is further supported by the EPR measurements after OER (Figure 5), where the narrow signal at *g* = 2.0 indicates a strongly increased number of defects in the carbon network resulted from corrosion. This phenomenon of carbon corrosion has been reported in our previous studies.^[^ ^31^, ^53^ ^]^ The breaking of C─C/N bonds as a result of carbon corrosion at the areas of edge or defect allows metal atoms to leach out and subsequently react with OH^−^ in the electrolyte to form (oxy)hydroxide clusters. However, the inductively coupled plasma mass spectrometry (ICP‐MS) measurements of the electrolyte before and after OER demonstrate no significant difference in the concentration of metal ions. This could be attributed to the extremely low metal content in catalysts, with only a small fraction being leached, as observed in post‐STEM measurements. Moreover, the leached metal ions near the electrode are likely to rapidly redeposit onto the electrode surface forming (oxy)hydroxides under oxidative potential and high concentration of OH^−^, rather than diffusing into the electrolyte. Nevertheless, the similar corrosion of carbon supported SACs was also reported very recently.^[^ ^54^ ^]^ On the other hand, the surface migration pathway cannot be ruled out. We performed the OER tests of the catalysts in the presence of EDTA, which is a well‐known strong chelating agent for Fe, Co, and Ni ions in highly alkaline solution (Figure S90).^[^ ^55^ ^]^ Our results indicate that the bimetallic CoFe and NiFe single‐atom catalysts still exhibit superior OER performance compared to their monometallic Fe, Co, and Ni counterparts in the presence of 0.3 M EDTA. Moreover, the catalysts after the electrochemical tests were conducted the STEM measurements (Figure S91). Clearly clusters were still observed although the overall densities of the clusters are lower than the samples without using EDTA. This suggests that even when the leaching–redeposition mechanism is entirely suppressed, metal site migration must still occur on the catalyst surface. Therefore, the surface migration mechanism cannot be entirely ruled out. Based on the above discussion, we infer that leaching–redeposition and surface migration pathways are coexisted during OER.

Identifying the true active sites in a single‐atom system remains a considerable challenge. To shed light on this topic, CV tests were applied to all samples in 1 M purified KOH and compared the results with these obtained in non‐purified KOH (Figure S32). Monometallic Fe, Co, and Ni catalysts exhibited a significant decline in performance after 10 CV cycles in purified KOH. However, their CV performance remained relatively stable in nonpurified KOH, suggesting that Fe impurities in the electrolyte play a crucial role in enhancing the activity and stability of these monometallic catalysts. In contrast, the bimetallic NiFe and CoFe catalysts demonstrated exceptional stability and high performance in both purified and nonpurified KOH. This indicates that the superior activity of bimetallic catalysts primarily originates from the formation of Fe─O─Ni/Co clusters, whereas the formation of monometallic Fe, Co, and Ni clusters appears to suppress OER activity in monometallic Fe, Co, and Ni catalysts. Therefore, it is reasonable to infer that isolated single‐metal sites in the monometallic catalysts play a decisive role in OER, whereas the formation of Fe‐O‐Ni/Co clusters in the bimetallic catalysts serves as the primary active sites. The above discussions demonstrate that the as‐prepared SACs served as the “precatalysts” rather than the actual catalysts, with the true active species synchronously generated during catalytic OER. Therefore, the assignment of the real active sites in this class of catalyst requires careful consideration. The corrosion of the carbon support as a result of structural reconstruction of the metal centers should be considered when investigating active site‐related reaction mechanisms in such catalysts.

## Conclusion

In this study, we introduced a coordination confinement strategy for the successful construction of atomic metal sites on carbon support using tannic acid and carbon nitride as the precursors. The atomically dispersed nature of the metal active sites was confirmed through direct observation and structural identification by STEM and XAS studies, respectively. The as‐prepared catalysts exhibit impressive OER performance. Notably, the presence of Co─Fe and Ni─Fe pairs on the same carbon support significantly enhances the catalytic OER performances compared to their single metal counterparts, achieving remarkable OER performance with overpotentials of 320 and 370 mV versus RHE, respectively. Most importantly, the postcatalytic EPR and in situ XAS studies of the catalysts containing Fe, Co, and Ni metals demonstrate that the single metal sites undergo structural reconstruction during the OER, leading to the formation of (oxy)hydroxides clusters with M─O─M/M’ (M/M’ = Fe, Co and Ni) bonds, which likely serves as the true active species for OER. The formation of clusters has been further confirmed by STEM measurements before and after OER. Therefore, careful consideration is needed when assigning real active sites in this class of catalysts, and the degradation of the carbon support as a result of structural reconstruction of the metal centers as well as the stability should be taken into account when investigating active site‐related reaction mechanisms. In summary, we provide an economical and convenient strategy for synthesizing a diverse range of carbon‐supported single‐site catalysts. The success in monitoring the dynamic structural reconstruction of the metal sites during working conditions provides mechanistic insight into the understanding the active centers of carbon‐supported single‐atom catalysts, which is essential and impactful for future research in determining true active sites in this class of materials.

## Conflict of Interests

The authors declare no conflict of interest.

## Supporting information



Supporting information

## Data Availability

The data that support the findings of this study are available from the corresponding author upon reasonable request.
