# Supplementary material for: Carbon‐Supported Single Fe/Co/Ni Atom Catalysts for Water Oxidation: Unveiling the Dynamic Active Sites
Source: Angew Chem Int Ed Engl. 2025 May 6;64(25):e202424629. doi: 10.1002/anie.202424629 (PMC12171328; doi:10.1002/anie.202424629)
Supplement: Supplementary file 1 — Supporting information [file ANIE-64-e202424629-s001.docx]

**Carbon Supported Single Fe/Co/Ni Atom Catalysts for Water Oxidation: Unveiling the Dynamic Active Sites**

Wenchao Wan,^a,1^ Liqun Kang,^a,1^ Alexander Schnegg,^a^ Olaf Ruediger,^a^ Zongkun Chen,^a^ Christopher S. Allen,^b,c^ Longxiang Liu,^d^ Sonia Chabbra,^a^ Serena DeBeer,^a,*^ Saskia Heumann^a,*^

[a] Dr. Wenchao Wan, Dr. Liqun Kang, Dr. Alexander Schnegg, Dr. Olaf Ruediger, Dr. Zongkun Chen, Dr. Sonia Chabbra, Prof. Dr. Serena DeBeer, Dr. Saskia Heumann
Max Planck Institute for Chemical Energy Conversion
45470 Mülheim an der Ruhr, Germany
E-mail: [serena.debeer@cec.mpg.de](mailto:serena.debeer@cec.mpg.de); [saskia.heumann@cec.mpg.de](mailto:saskia.heumann@cec.mpg.de)

[b] Dr. Christopher S. Allen
Electron Physical Science Imaging Center
Diamond Light Source Ltd
Didcot, Oxfordshire, OX11 0DE, UK

[c] Dr. Christopher S. Allen
Department of Materials
University of Oxford
Parks Road, Oxford, OX1 3HP, UK

[d] Dr. Longxiang Liu
Department of Chemistry
University College London
20 Gordon St, London WC1H 0AJ, UK

[1] These authors contributed equally.

[*] Corresponding authors.

# 1. Materials

Dicyandiamide (C_2_H_4_N_4_) (≥99.0 %), Ni(NO_3_)_2_∙6H_2_O (≥99.990 %), Co(NO_3_)_2_∙6H_2_O (≥99.99 %), Fe(NO_3_)_3_∙9H_2_O (≥99.99 %), Tannic acid (C_76_H_52_O_46_) (ACS reagent), Platin(II)-acetylacetonat (C_10_H_14_O_4_Pt) (≥97.0%) were purchased from Sigma-Aldrich. Argon gas (Ar ≥99.999 %) was used in the experiment. Potassium hydroxide 1 M KOH (1N) volumetric solution was purchased from Fluka. The water used for all experiments is ultra-pure water.

# 2. Syntheses

**g-C_3_N_4_**. g-C_3_N_4_ was obtained via a thermopolymerization procedure, in which dicyandiamide (C_2_H_4_N_4_) was placed into a crucible covered with a lid and heated up to 550 °C in the furnace with a ramp rate of 2.5 °C/min and held for 2 h in air. The yellow bulk was ground into powder for further use.

**Single Atom Catalysts (SACs).** 1g g-C_3_N_4_, 0.06 mmol metal ions (for Fe, Co and Ni SSCs) and 300 mg Tannic acid were mixed into 40 mL in deionized water. After ultrasonication (3 h) and magnetic stirring (24 h) treatments, the mixture was further subjected to an evaporation procedure at 60 °C to remove the water. For CoFe and NiFe SSCs, keep the total amount of metal the same, but fix the ratio of Co/Ni to Fe at 3:1. The above solid was further ground into a fine powder and subsequently treated with a pyrolysis procedure for 2 h in a tube furnace at 900 °C with a ramp rate of 2.5 °C/min in Ar atmosphere.

**Free-standing electrodes**. The above ground fine powder was pressed into pallets using a pressing device with the weight of 5 tons for 5 min and subsequently treated with a pyrolysis procedure for 2 h in a tube furnace at 900 °C with a ramp rate of 2.5 °C/min in Ar atmosphere.

# 3. Electrochemical measurements

Electrochemical water oxidation measurements were carried out on a BioLogic VMP3 Potentiostat with the standard three-electrode system in 1 M KOH electrolyte. Hg/HgO filled with 1M KOH solution and Pt mesh were used as reference electrode and counter electrode, respectively. A glassy carbon (GC) electrode with a diameter of 5 mm loaded with catalyst was used as the working electrode. 2.5 mg of catalyst was dispersed in 1 mL ethanol. After 30 min of ultrasonication, 50 μL of 5 wt % Nafion solution was further added into the above solution with another 60 min of sonication to form a homogeneous ink. Then 10 μL of the catalyst ink (containing 25 μg of catalyst) was loaded onto a glassy-carbon electrode (5 mm in diameter). All the catalyst electrodes were continuously scanned for 10 CV cycles before measuring polarization curves. The scan rates for all the linear sweep voltammetry (LSV) and cyclic voltammetry (CV) measurements were set to 10 and 50 mV/s ranging from 0 to 1.8 V vs. RHE to minimize the capacitive current. The potentials were referenced to the RHE (E_RHE_ = E_Hg/HgO_ + 0.098 + 0.0591*pH V). EIS measurements were conducted within a frequency range from 0.01 Hz to 10 kHz with a 5 mV amplitude at1.5 V and OCP vs. RHE.

The number of active sites was calculated based on the hypothesis that all the metal atoms loaded onto the electrode participate in the reaction. The actual number of metal active sites should be lower than the calculated value. The metal content was determined by the XRF measurement. Therefore, the total active sites (N) are:

$$N=(\frac{0.0125 mg*metal content \left( wt. \% \right)}{0.19625 {cm}^{2}*mole mass})(6.02*{10}^{23})$$

in which, 0.0125 mg is the total mass of the catalyst loaded onto the glassy carbon electrode, 0.19625 cm^2^ is the surface area of the glass carbon electrode. It is worth noting that the actual number of metal active sites should be lower than the calculated value, therefore the calculated TOFs are also lower than the real TOFs. The metal content determined by XRF reflects the real active sites exposed to the electrolyte.

The total number of oxygens turn overs was calculated from the current density according to:^[1]^

$${\#O}_{2}=\left( j\frac{\mathrm{mA}}{\mathrm{cm}^{2}} \right)\left( 1\frac{\frac{C}{s}}{1000 mA} \right)\left( \frac{1 mol e^{-}}{96485.3 C} \right)\left( \frac{1 mol O_{2}}{4 mol e^{-}} \right)\left( \frac{6.02*{10}^{23} O_{2}\mathrm{molecues}}{1 \mathrm{mol} O_{2}} \right)=1.56* {10}^{15}\frac{O_{2}}{\mathrm{cm}^{2}} per \frac{\mathrm{mA}}{\mathrm{cm}^{2}}$$

The turnover frequency (TOF) was calculated to be:

$$TOF=\frac{1.56*{10}^{15}\frac{O_{2}/s}{{cm}^{2}} j}{N\frac{\mathrm{atoms}}{{cm}^{2}}}$$

In which, j is the current density, N is the calculated number of active sites.

**KOH Electrolyte Puriﬁcation**

**Method 1:** The electrolyte purification was carried out electrochemically using a two-electrode setup, where a NiS_3_–MoS_2_ catalyst was deposited on Ni foam electrodes (3 × 1 cm² geometric surface area, with a high porosity of 95% from Goodfellow). The molar content of the MoS_2_ catalyst on the Ni foam was 8%. During the electrolysis, a constant current of 100 mA was applied in a 1 L standard polystyrene bottle. Over a 12-hour period, samples were collected and analyzed using ICP-OES to measure the residual Fe content. The voltage supplied by the potentiostat was approximately 1.9 V, gradually decreasing throughout the 12-hour electrochemical purification process. This decrease signalled the activation of the Ni foam catalyst and indicated the removal of Fe from the KOH electrolyte, with Fe being incorporated into the Ni foam electrode. After the process, the electrolyte was filtered and stored for further use, while the Ni foam electrodes were thoroughly washed with deionized water and dried under a stream of argon for several minutes.

**Method 2:** In a H_2_SO_4_-cleaned 50 mL polypropylene centrifuge tube, approximately 2 g of 99.999% Ni(NO_3_) _2_·6H_2_O was dissolved in around 4 mL of 18.2 MΩ·cm H_2_O. Then, 20 mL of 1 M KOH was added to precipitate high-purity Ni(OH) _2_. The mixture was shaken and centrifuged, and the supernatant was decanted. The Ni(OH) _2_ was then washed three times by adding approximately 20 mL of 18.2 MΩ·cm H_2_O and 2 mL of 1 M KOH to the tube, re-dispersing the solid, centrifuging, and decanting the supernatant. Finally, the tube was filled with 50 mL of 1 M KOH for purification. The solid was re-dispersed and mechanically agitated for at least 10 minutes, followed by a rest period of at least 3 hours. The mixture was centrifuged, and the purified KOH supernatant was decanted into an H_2_SO_4_-cleaned polypropylene bottle for storage.

# 4. Material characterizations

## Powder X-ray diffraction (PXRD)

PXRD patterns were recorded on Bragg-Brentano geometry on a Bruker AXS D8 Advance II theta/theta diffractometer using Cu Kα X-ray source.

## Scanning Electron Microscopy (SEM)

SEM images were acquired by Phenom Pharos G1 with a Magnification of 80-500000x and a resolution of 3nm.

## Scanning transmission electron microscopy (STEM)

High-resolution aberration-corrected Bright Field (ABF) and High Angle Annular Dark Field (HAADF) STEM images were acquired using a probe-corrected (COSMO) JEOL ARM300CF electron microscope in the E02 lab of the electron Physical Science Imaging Centre (ePSIC) at Diamond Light Source (DLS, UK). The acceleration voltage for the electron gun was 80 kV. The probe size was set to 8C (Spot 8) with a 20 μm probe-forming aperture (CL aperture) selected, resulting in a probe convergence semi-angle of 15.44 mrad and a beam current of 11.0 pA. The STEM camera length was set to 9.0 cm, which allowed the ADF detector to integrate the scattered electron intensity between 73.7 ± 1.8 and 155.4 ± 1.8 mrad. In addition, a 3 mm aperture was inserted for the BF imaging, corresponding to a semi-angle of 14.8 ± 1.2 mrad (outer angle) for the BF detector. For each sample, a small amount of dry powder was first suspended in ethanol using ultrasonication. About 5 µL of this mixture was dispersed on a 400-mesh Cu grid with lacey carbon support film. The grid with sample loaded was then baked inside a vacuum chamber at 60 ^o^C for 15 minutes. Before taking any STEM images, each sample was exposed to an intense electron beam for 10-15 minutes ('beam shower’) to eliminate the accumulation of carbon contamination. Gatan Microscopy Suite software was used for image data acquisition.

STEM-EDS measurements were conducted via a Hitachi HD-2700 CS-corrected dedicated STEM 200 kV, Cold FEG. EDX: EDAX Octane T Ultra W 200 mm^2^ SDD, TEAM-Software.

Additional STEM-EDS data were acquired on a probe-corrected (CEOS) JEOL ARM200CF electron microscope in the E01 lab of ePSIC. A probe size of 5C (Spot 5) was selected and a 30 μm probe-forming aperture was used. The beam current was 47 pA with a 22.6 mrad probe convergence semi-angle at 80 kV operation voltage. The BF and HAADF signals were collected simultaneously at 8.0 cm STEM camera length, integrating the scattered electron intensity below 23.1 mrad and between 39.5 to 135.0 mrad, respectively. The elemental mapping data were acquired in STEM mode by two large solid-angle dual EDS detectors. The EDS elemental mapping area was 100 × 100 pixels (0.1 s exposure time per pixel) with spatial drifting correction every 30 s. Gatan Microscopy Suite Software was used for the EDS spectrum imaging data acquisition.

## Transmission Electron Microscopy (TEM)

The TEM images were collected on the FEI Tecnai G2 Spirit instrument with 120 kV transmission electron microscope equipped with two digital CCD cameras.

## Thermogravimetry-Gas chromatography–Mass spectrometry (TG-GC-MS)

The TGA-GC-MS measurements for all samples were conducted on the PerkinElmer Thermogravimetric-Gas Chromatography-Mass Spectrometry with Helium as the carrier gas. Before the measurements, all samples were dried in an oven at 100 °C. Powder samples were then put on the TGA and heated up from 100 °C to 1000 °C with a heating rate of 50 °C/min. The analyses of the GC and MS were performed every few minutes. The samples used for the measurements were around 4~6 mg and the ratios of different components are the same as described in the syntheses.

## Raman spectroscopy

Ramam spectra were collected on a Thermo Scientific DXR Raman Microscope with a 50 × magnification and 532 nm laser. The measurements before OER were performed using powder samples. Results after OER were acquired using samples dropped on carbon cloth electrodes.

## Electron Paramagnetic Resonance (EPR)

The EPR spectra were obtained using an X-band (~9.64 GHz) Bruker Elexsys E500 EPR spectrometer equipped with a ER4116DM dual mode resonator and an ESR 900 He cryostat. The sample temperature was stabilised at 10 K using a He flow cryostat (Oxford Instruments). The EPR spectra were obtained using a range of microwave power between 20 mW and 6.3 mW for power saturation. The optimized microwave power used for individual samples are mentioned in the corresponding figures. The EPR spectra were recorded at 10 and 30 K with 700 mT field sweep centred at 355 mT, a modulation amplitude of 0.7 mT, a time constant of 40.96 ms, a sweep time of 84 s and a modulation frequency of 100 KHz.

## X-ray Fluorescence (XRF)

XRF results were collected by the Xepos C, Spectro with the measurement time of 10 mins for each sample in the atmosphere (during measurement) of helium/air mixture, using energy range (to be scanned) of 3keV – 19keV. Foil (what the bottom of the sample cuvette is made of): Polypropylene 12µm.

## Near Edge X-ray Absorption Fine Structure (NEXAFS)

NEXAFS spectra were collected at the ISISS facility of BESSY II (Germany).^[2]^ At the ISISS beamline the X-ray produced by a bending magnet source (D41 dipole) and monochromatized using a PGM (600 l/mm), delivering a photon flux of 6× 10^3^ photons/s with a focused size of 100 µm (H) × 80 µm (V). The NEXAFS experiment was performed using a NAP-HE-XPS endstation (SPECS GmbH). The endstation is equipped with a differentially pumped near ambient pressure hemispherical energy analyser, which was employed for both XPS and NEXAFS measurements. Co L_3,2_-edge NEXAFS for the fresh Tan-CN-Co and Tan-CN-CoFe catalysts (in pellet format) were acquired from 770 eV to 805 eV with a step size of 0.05 eV and a dwell time of 0.1 s. The Auger electron yield (AEY) signal was collected at a kinetic energy of 645 eV with an energy pass (Epass) of 30 eV, which corresponds to the Co L_3_M_23_M_23_ Auger emission. Meanwhile the sample drain current was also monitored for the total electron yield (TEY) signal.

## X-ray photoelectron spectroscopy (XPS)

XPS analysis was carried out on an ESCALAB 250 XPS system with a monochromatized Al Kα X-ray source (1486.6 eV). Pellets samples were used for the measurements. A Shirley background was subtracted from the area of the peaks to estimate their intensity. In this work, the main component of the C 1s signal was used as an internal reference, which was centered at 284.7 eV.

Additional Co 2p XPS spectra for fresh Tan-CN-Co and Tan-CN-CoFe catalysts were collected at the ISISS facility of BESSY II (Germany).^[2]^ The detailed beamline configurations are described previously in the section for NEXAFS measurement. The Co 2p XPS spectra were acquired from binding energy of 776 eV to 812 eV with a step size of 0.05 eV using an excitation photon energy of 1430 eV. The dwell time and energy pass (Epass) were set to 0.1 s and 10 eV to improve the signal to noise ratio. It is worth noting that, at an excitation energy of 1430 eV, the Co L_3_M_23_M_23_ and Fe L_3_M_23_M_45_ (^1^P) Auger emission peaks (located at kinetic energies of approximately 649 eV and 648 eV, respectively) may appear at binding energies of 781 eV and 782 eV. This overlap could potentially interfere with the Co 2p_3/2_ feature in the XPS spectra.

## X-ray absorption spectroscopy (XAS)

All *ex situ* samples were measured at the P65 beamline of PETRA III (P65 Applied X-ray Absorption Spectroscopy).^[3,4]^ At P65 beamline, monochromatic beam was introduced through an 11-periods undulator and a Si (311) double crystal monochromator (DCM) with energy resolution *Δ*E/E of 6.0 × 10^-5^. The DCM was operated in QEXAFS mode, and the undulator offset to the DCM was calibrated to have the maximum photon flux. The beam size at the sample was approx. 0.5 x 1.0 mm^2^ (V x H) and the photon flux was ~10^11^ photons/s (without attenuation). The energy of the DCM was calibrated for each absorption edge using the corresponding metal foil. The energy of the incident beam was calibrated by assigning the energy of the first inflection in the first derivative XANES of Fe foil, Co foil and Ni foil to 7112.2 eV, 7709 eV and 8333 eV, respectively. The *ex situ* samples were prepared into pellets and measured in transmission mode with the corresponding metal foil measured simultaneously. The intensity of incident beam (I_0_) and the transmitted beam (I_t_) was monitored by ionization chambers (filled with mixture of N_2_ and Ar). For each sample, 3~10 repetition scans were acquired and merged to improve the signal to noise ratio.

The in situ XAS characterizations were performed at the SAMBA beamline in SOLEIL (France).^[5,6]^ X-ray beam is generated through a bending magnet (E_c_=8.65 keV) and monochromatized using a Si(220) double crystal monochromator (DCM) with a resolving power of 6×10^-5^. The beam spot on the sample has a FWHM of 200 μm ×300 μm (V×H) with a photon flux of 3×10^12^ ph/s. The DCM was operated in QEXAFS mode, allowing continues collection of XANES and full EXAFS spectrum every ~5 minutes. The energy calibration was similar to the P65 beamline by measuring the metal foils. In situ XAS measurements were performed using in-house designed electrochemical cell. The working electrodes were prepared using the following procedure: 2.5 mg of catalyst was dispersed in 1 mL ethanol. After 30 min of ultrasonication, 50 μL of 5 wt % Nafion solution was further added into the above solution with another 60 min of sonication to form a homogeneous ink. Then 10 μL of the catalyst ink loaded onto a carbon cloth electrode (1cm^2^). All the catalyst electrodes were continuously scanned for 10 CV cycles before measuring polarization curves. The metal loading concentration on the working electrodes were optimized for electrochemical test, which is significantly lower than that of powder/pellet samples for ex situ transmission measurements. Therefore, multiple XAS scan repetitions in fluorescence mode were collected to improve the signal to noise ratio. A Canberra 35-elements monolithic planar Ge pixel array detector was used for collecting the fluorescence data in partial fluorescence yield mode (PFY). In addition, prior to the full XAS spectra collection, each catalyst was checked for radiation damage under the full flux of the incident beam by performing consecutive 1-minute short XANES scans at the absorption edge on the same sample spot for 20 times (in total of 20 minutes radiation exposure). The radiation damage tests showed that all Tan-CN-M samples were stable for 20 minutes. Based on this information, for each working electrode, 10-12 scans were collected on 8-12 sample spots with around 120 minutes of beam exposure for each spot (horizontal and vertical spacings are 10 mm and 10 mm, respectively). To further reduce the plausible radiation damage on the sample, a fast shutter was installed between the ionization chamber I0 and the *in situ* cell. The fast shutter only opened when the XAFS scan was executed.

The XAS spectra were analyzed using the Demeter software package (including Athena and Artemis programs, version 0.9.26).^[7]^Pre-edge background subtraction and post-edge normalization of the XAFS data were performed using the Athena program. A linear regression background was determined, and a quadratic polynomial regression for post-edge normalization was applied. The spectra were splined from k=0 Å^-1^ to k=14 Å^-1^ with rbkg of 1.0 and k-weight of 2. The fitting of EXAFS spectra (R range: 1 to 3 Å, k range: 3.7 to 11.5 Å^-1^) was performed using the Artemis program based on scattering paths generated from FEFF6. The amplitude reduction factors S_0_^2^ were determined by fitting of k^2^-weighted R-space EXAFS of Fe foil, Co foil and Ni foil for each absorption edge respectively. The S_0_^2^ values for Fe K-edge (0.75), Co K-edge (0.72) and Ni K-edge (0.77) were used as fixed parameter for the EXAFS fitting of other samples. The wavelet transform EXAFS spectra were produced using the Cauchy wavelet transform function built in the xraylarch python library (version 0.9.74).^[8,9]^

The XAFS of α-FeOOH), γ-FeOOH, Fe(OH)_3_, CoOOH, Co(OH)_2_, NiOOH and Ni(OH)_2_ were standard spectra indexed in the BL14B2 XAFS Standard Sample Database,^[10]^ and the energy calibration was performed by aligning the XANES of Fe/Co/Ni foils measured at BL14B2 and SAMBA beamline.

# 5. Supplementary Figures


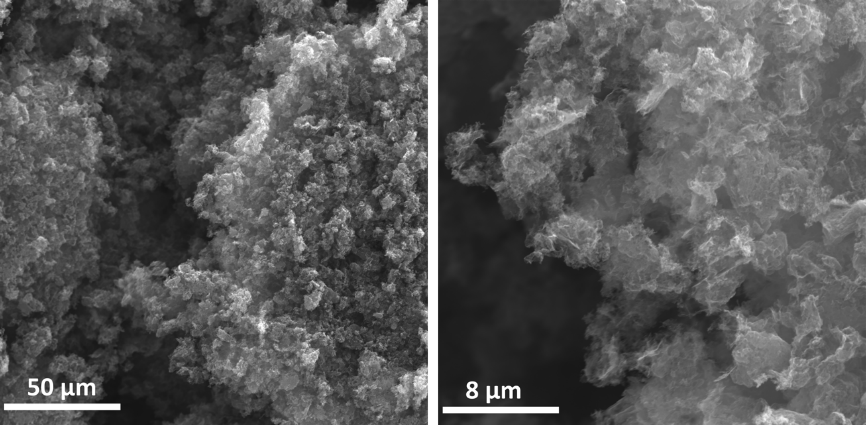


**Figure S1.** SEM images of Tan-CN-Ni.


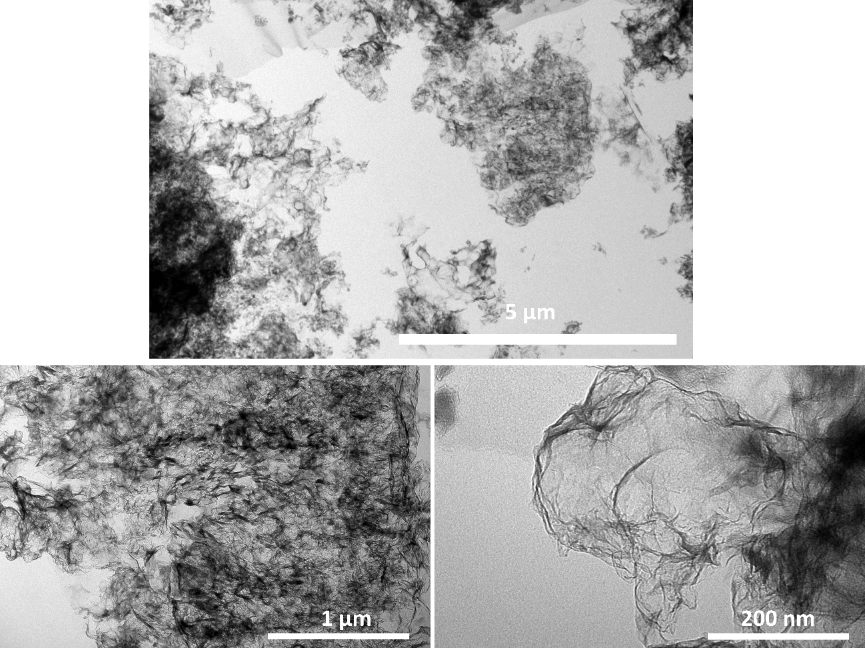


**Figure S2.** TEM images of Tan-CN-Fe.


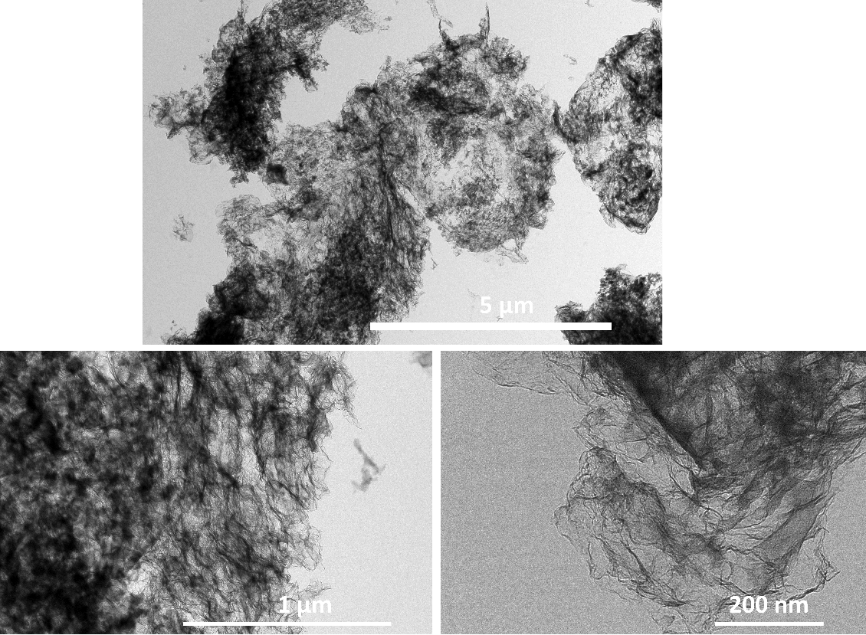


**Figure S3.** TEM images of Tan-CN-Co.


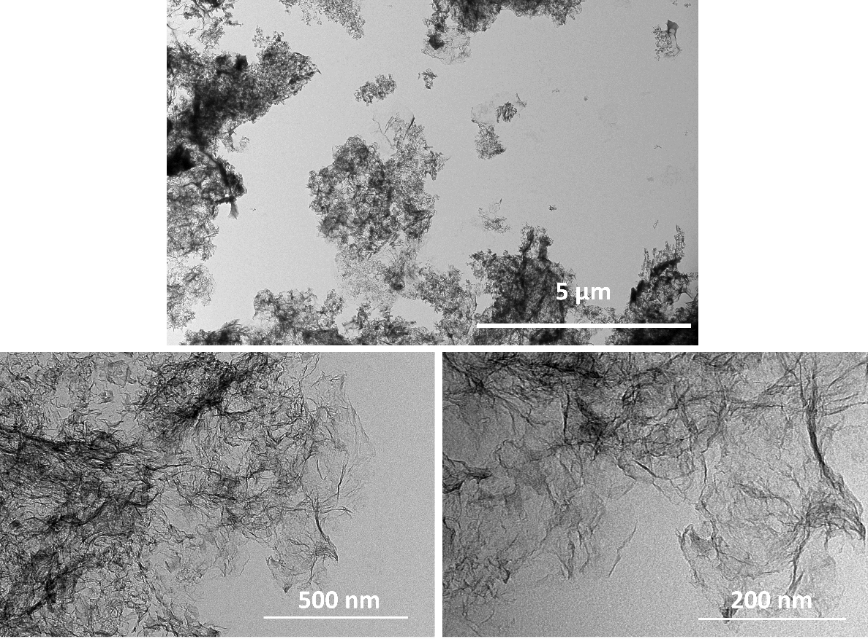


**Figure S4.** TEM images of Tan-CN-Ni.


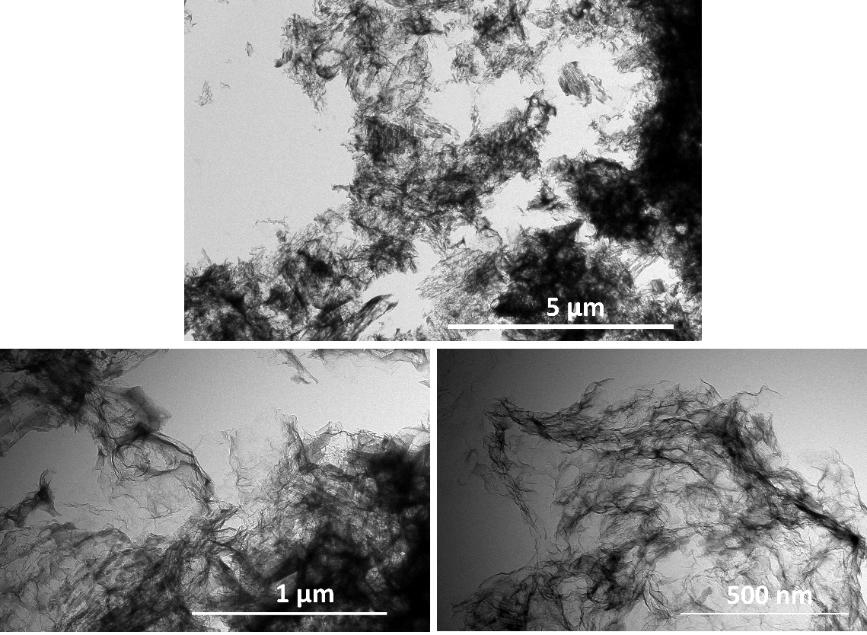


**Figure S5.** TEM images of Tan-CN-CoFe.


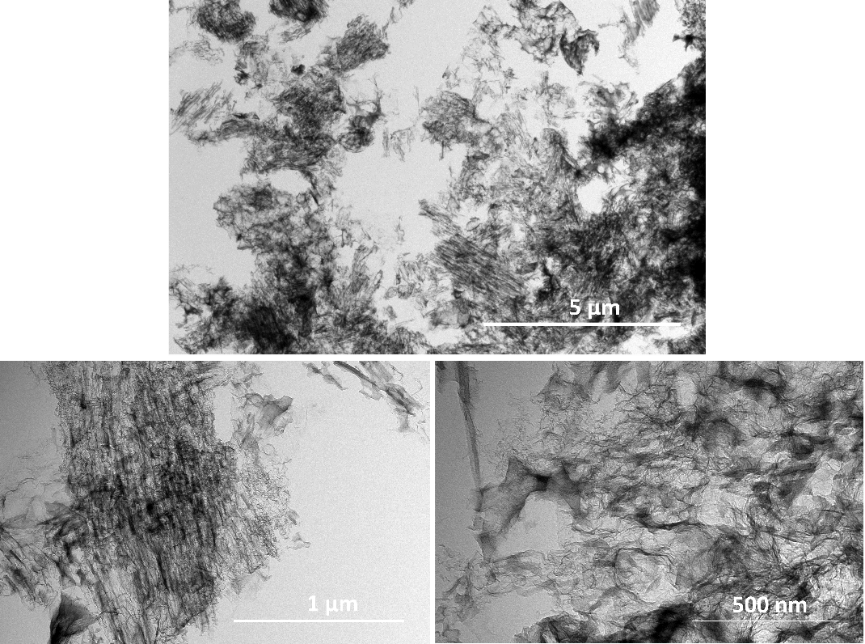


**Figure S6.** TEM images of Tan-CN-NiFe.


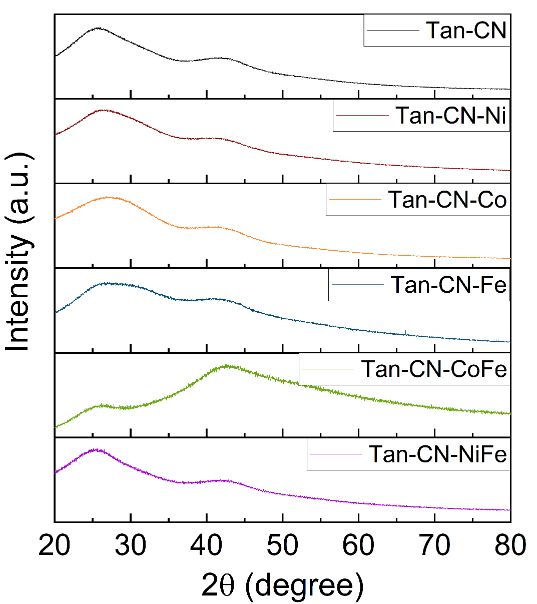


**Figure S7**. PXRD patterns of metal free Tan-CN and Tan-CN-Fe/Co/Ni/CoFe/NiFe.


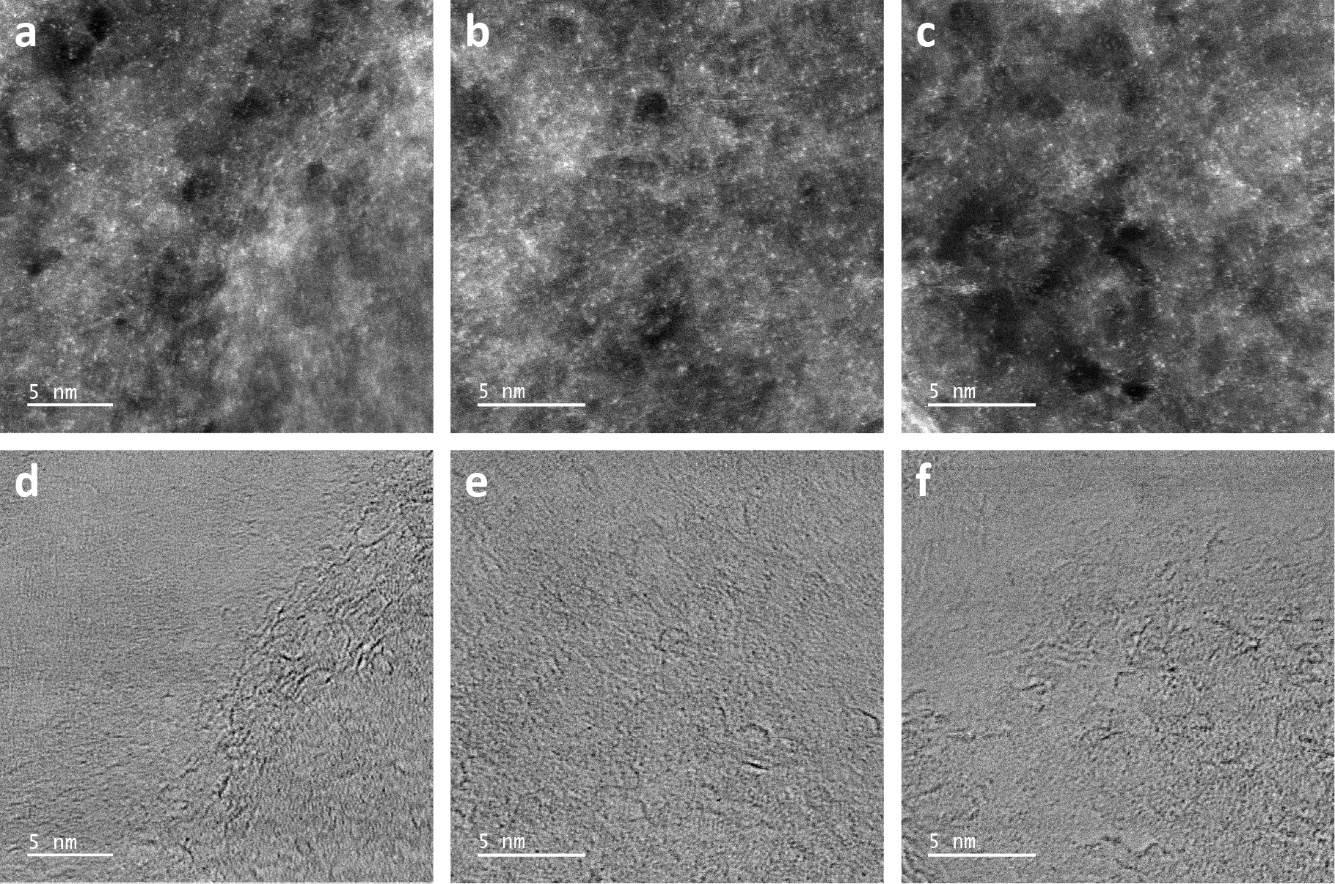


**Figure S8.** (a-c) HAADF-STEM images of Tan-CN-Fe. (d-f) Corresponding BF-STEM images.


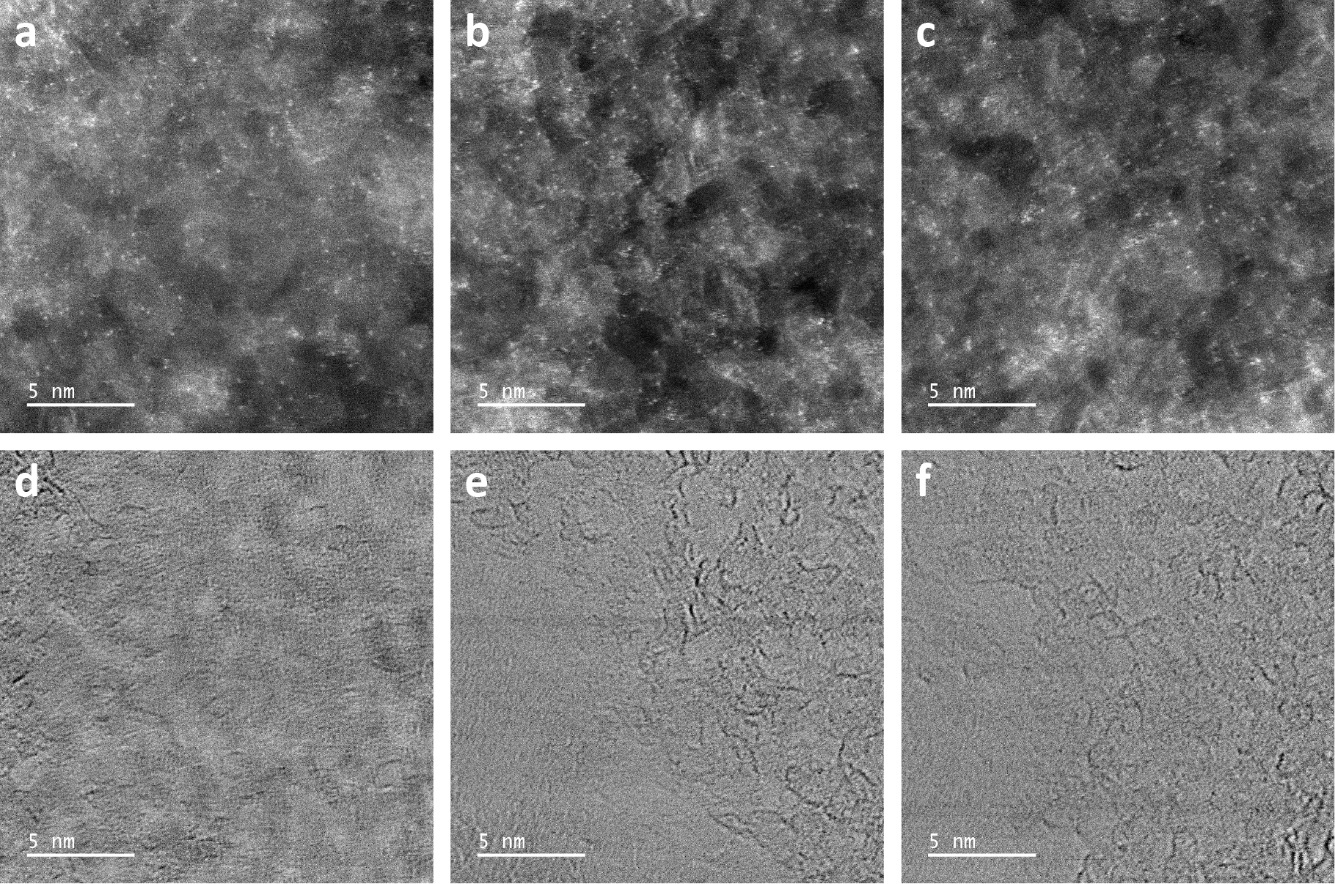


**Figure S9.** (a-c) HAADF-STEM images of Tan-CN-Co. (d-f) Corresponding BF-STEM images.


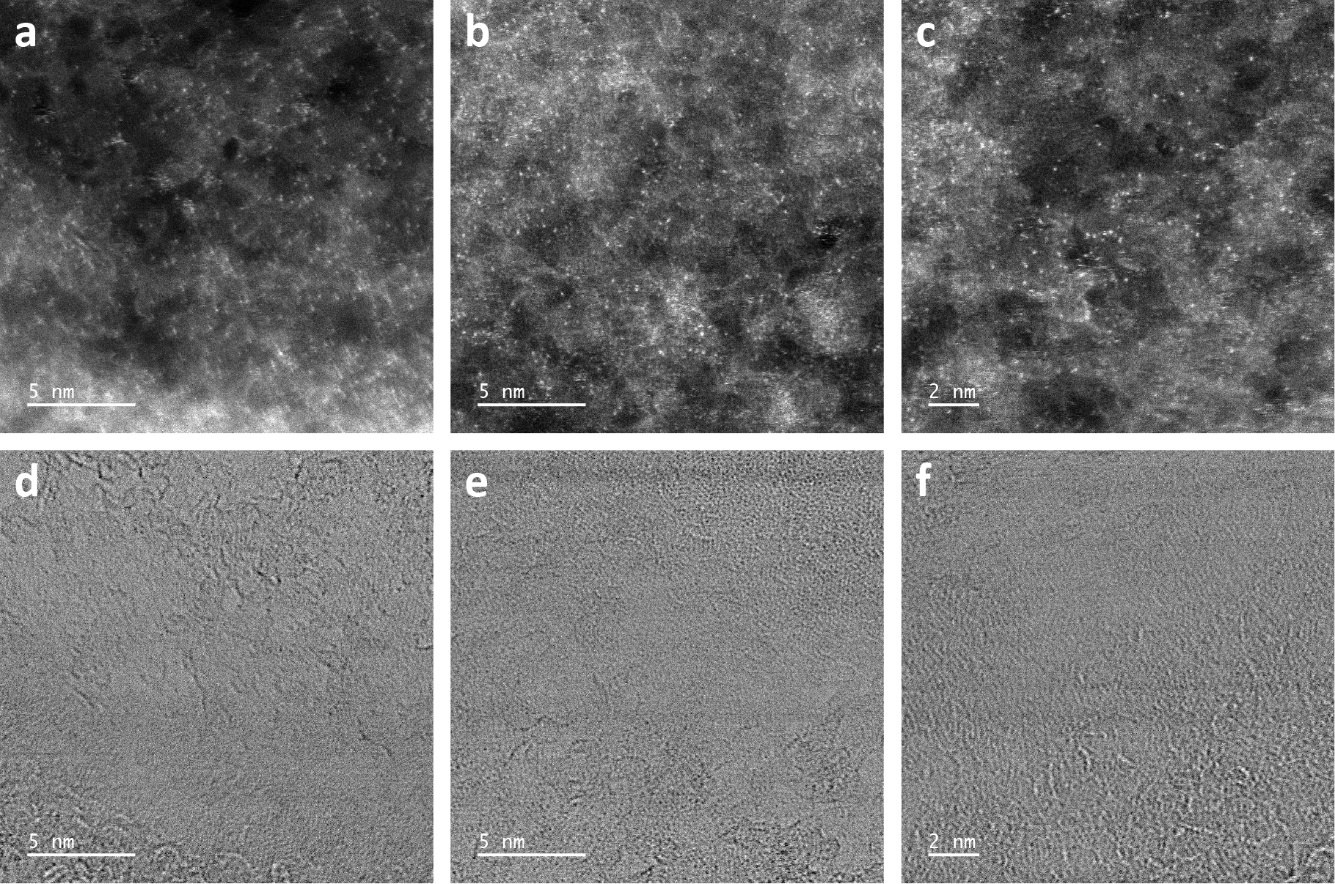


**Figure S10.** (a-c) HAADF-STEM images of Tan-CN-Ni. (d-f) Corresponding BF-STEM images.


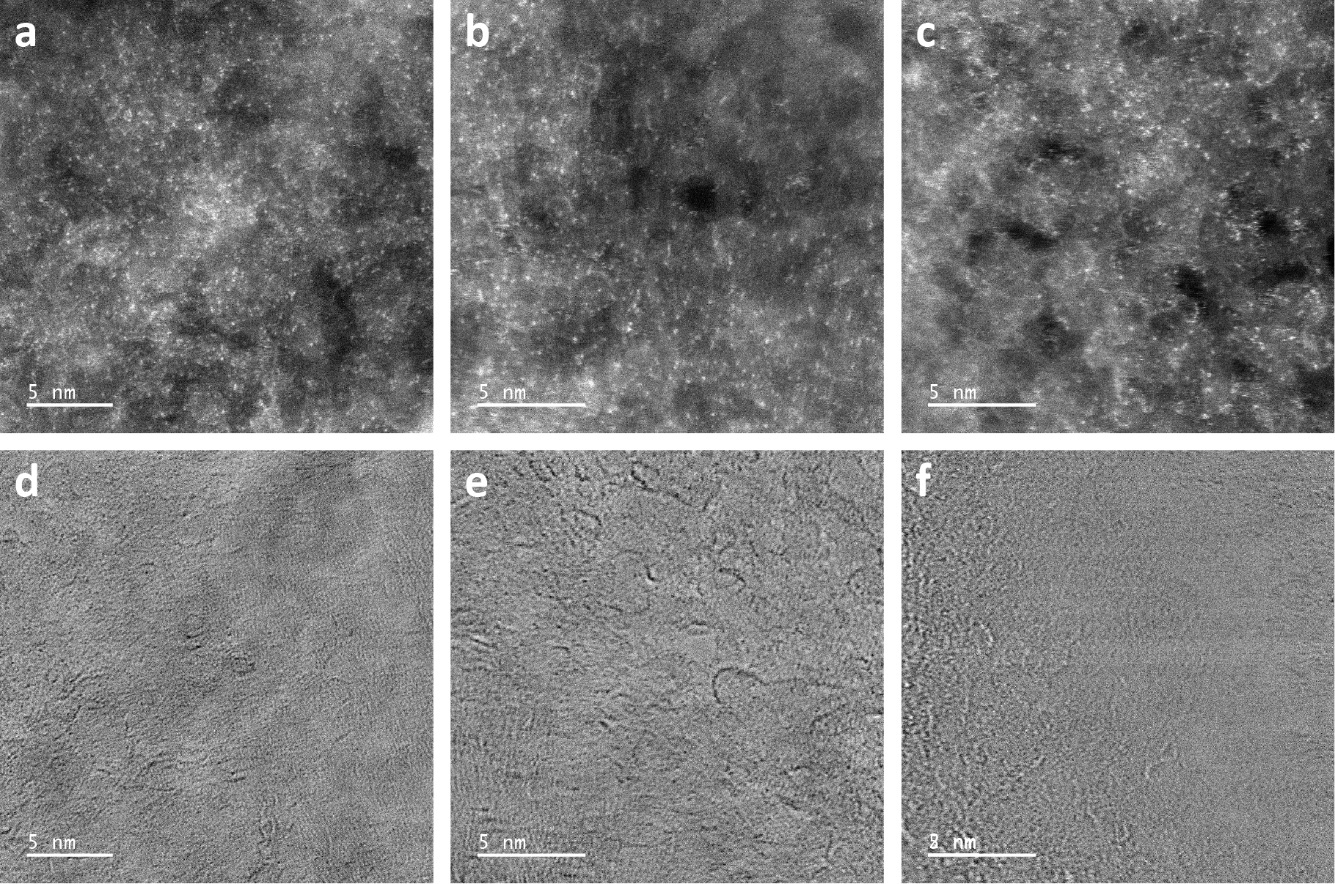


**Figure S11.** (a-c) HAADF-STEM images of Tan-CN-CoFe. (d-f) Corresponding BF-STEM images.


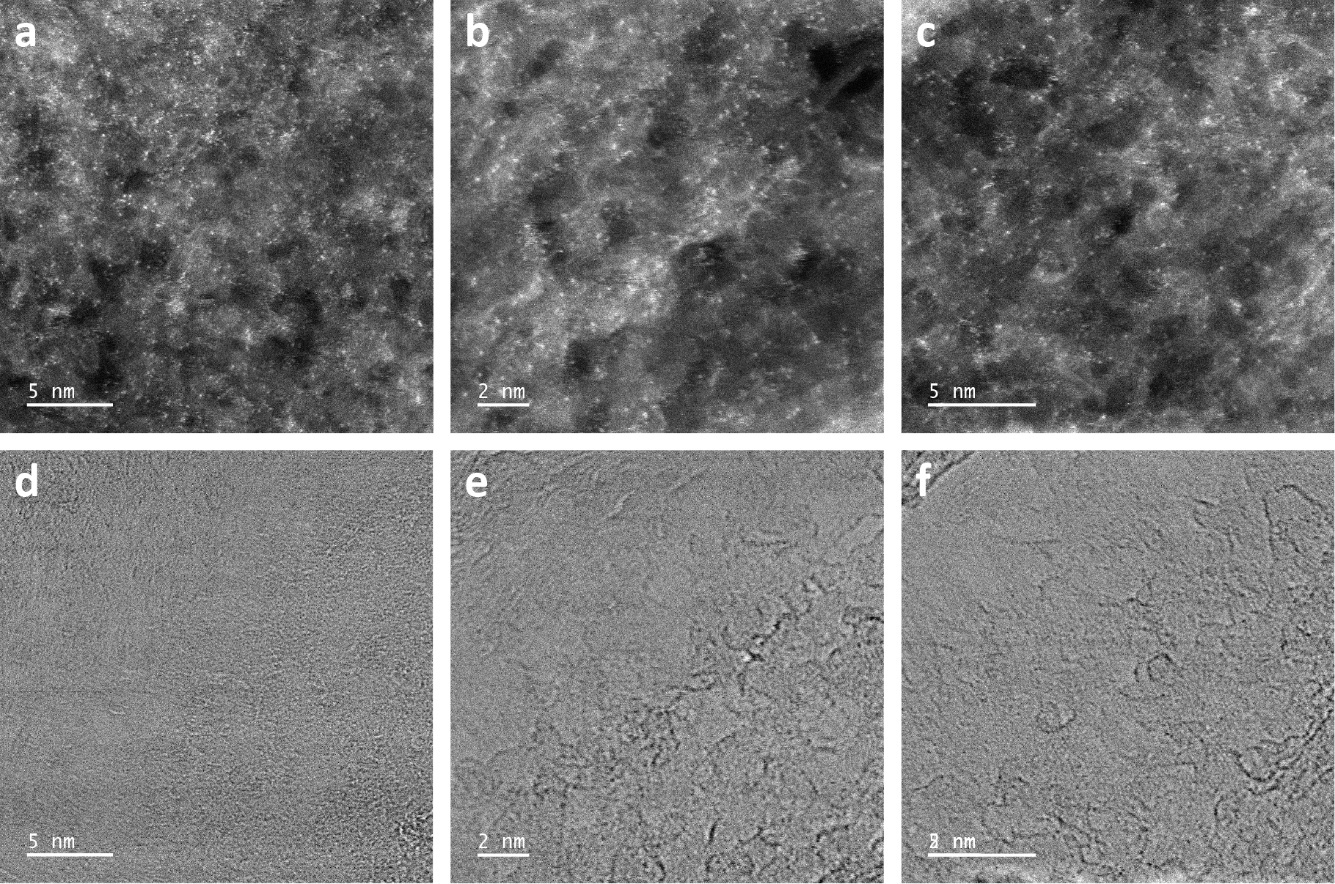


**Figure S12.** (a-c) HAADF-STEM images of Tan-CN-NiFe. (d-f) Corresponding BF-STEM images.


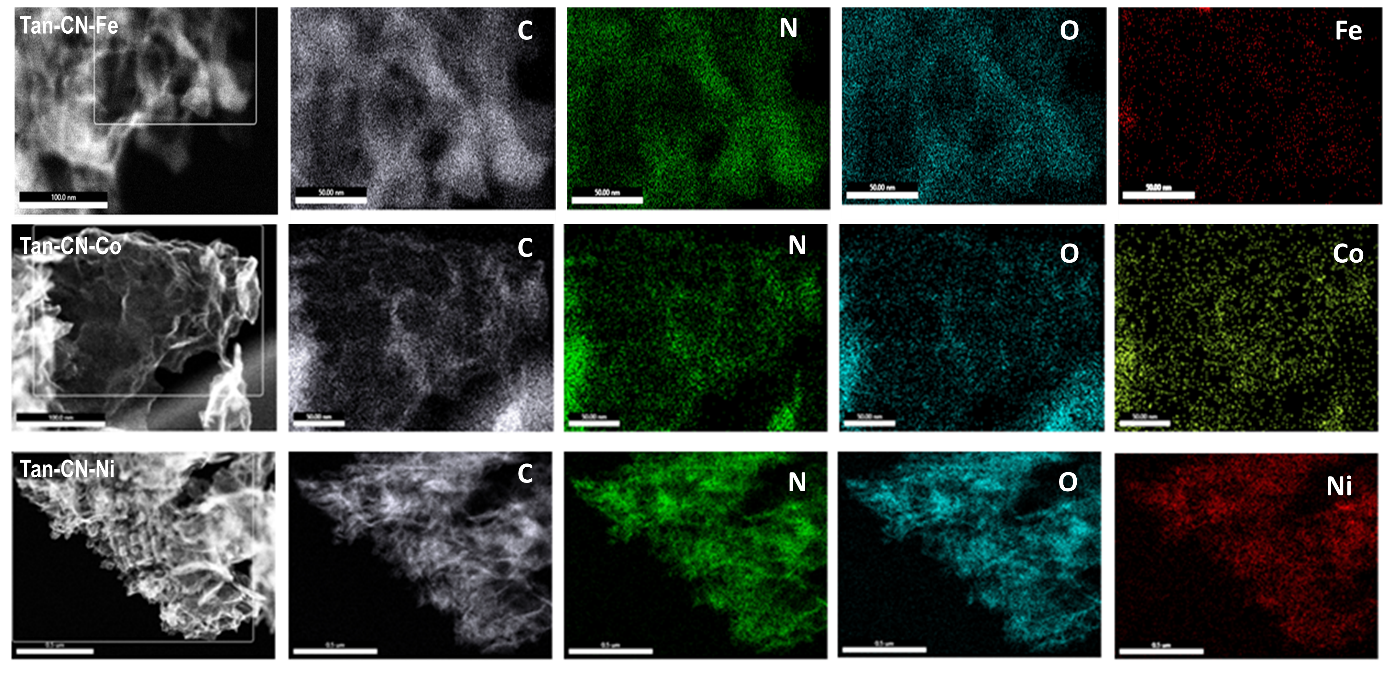


**Figure S13.** EDS elemental mapping of Tan-CN-Fe, Tan-CN-Co, and Tan-CN-Ni recorded along with their HAADF-STEM images with the scale bars of 50 nm, 50 nm and 500 nm, respectively (data acquired using Hitachi HD-2700).


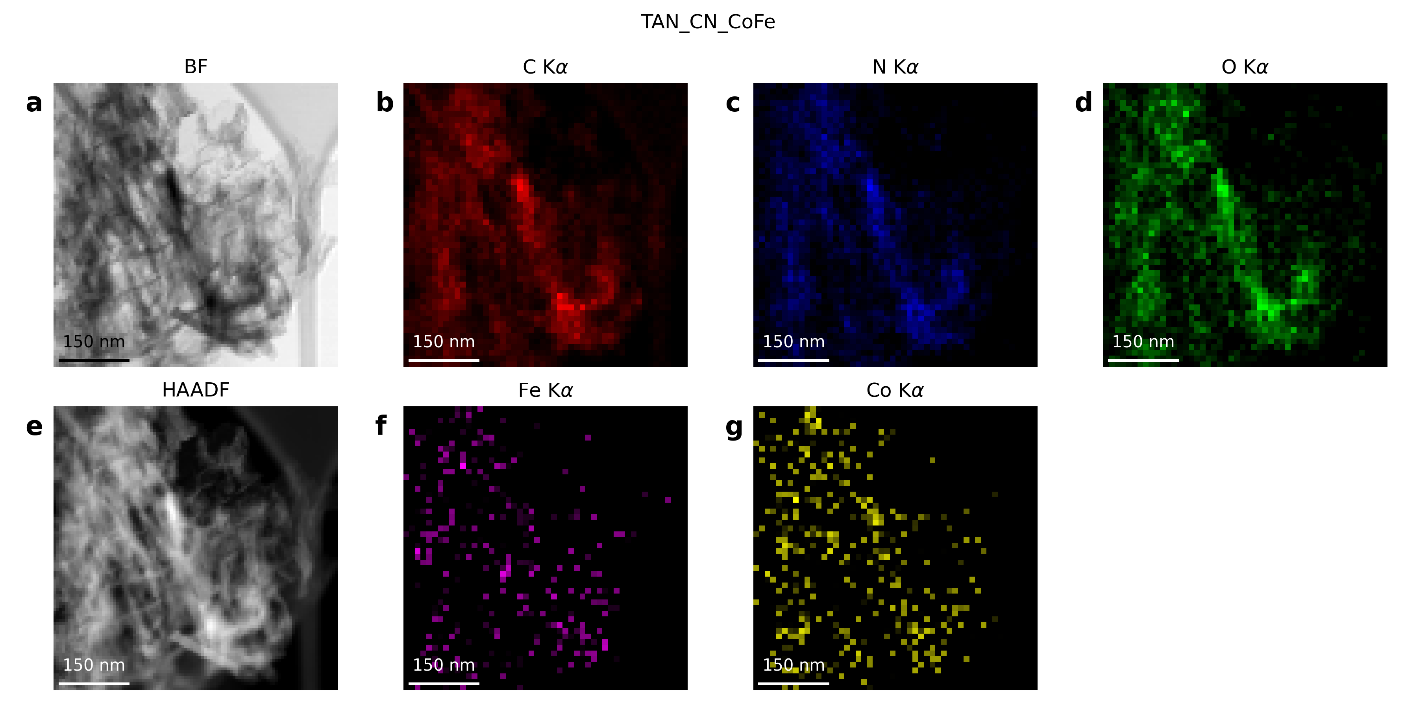


**Figure S14.** EDS elemental mapping of Tan-CN-CoFe was recorded along with their HAADF-STEM images with the scale bars of 150 nm (data acquired using JEOL ARM200CF).


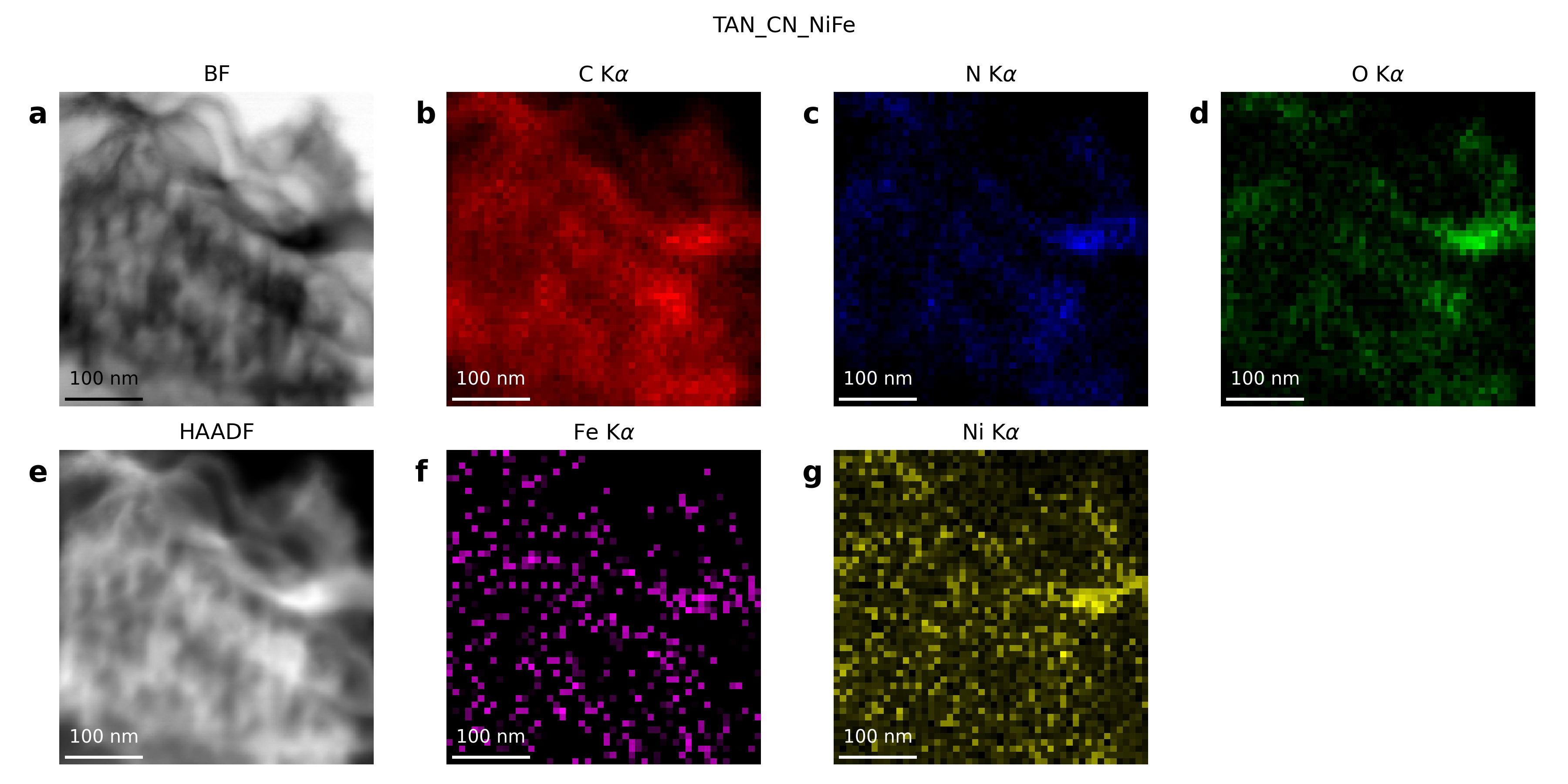


**Figure S15.** EDS elemental mapping of Tan-CN-NiFe recorded along with their HAADF-STEM images with the scale bars of 100 nm (data acquired using JEOL ARM200CF).


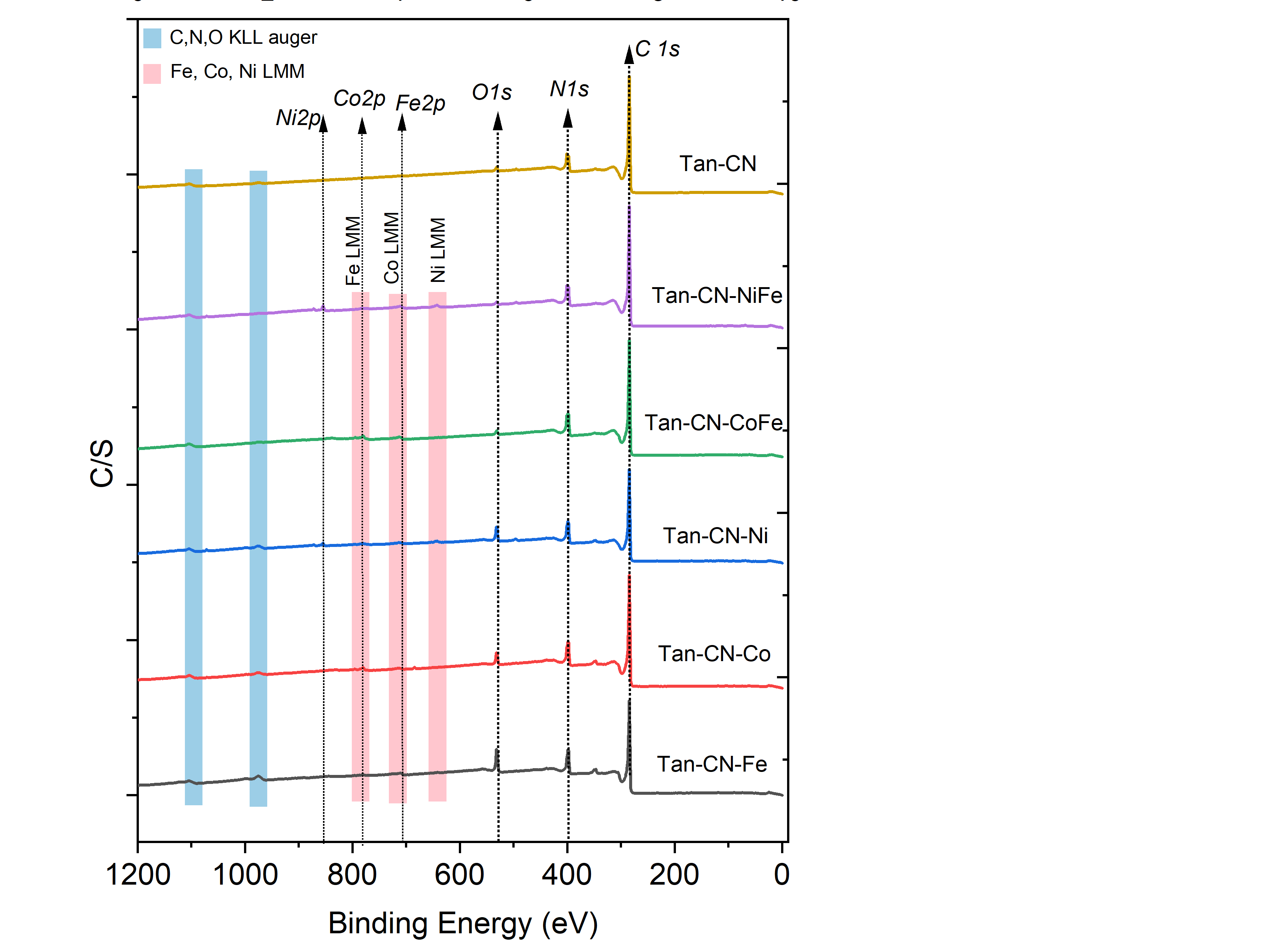


**Figure S16.** XPS survey scans for the Tan-CN sample series with excitation photon energy at Al Kα 1486.6 eV.


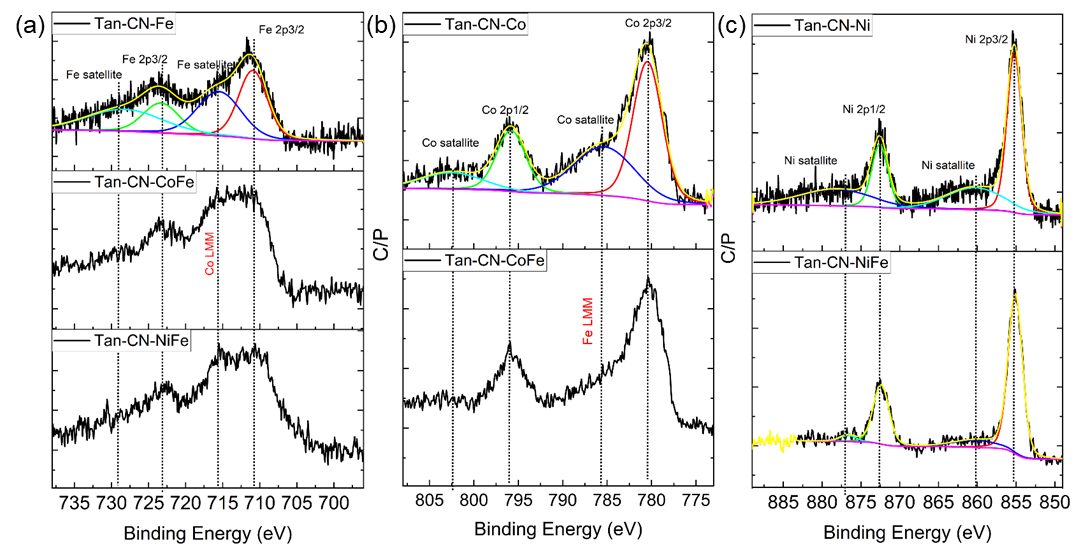


**Figure S17.** (a) Fe 2p XPS spectra for Tan-CN-Fe, Tan-CN-CoFe and Tan-CN-NiFe. (b) Co 2p XPS spectra for Tan-CN-Co and Tan-CN-CoFe. (c) Ni 2p XPS spectra for Tan-CN-Ni and Tan-CN-NiFe. These XPS spectra are acquired with excitation photon energy at Al Kα 1486.6 eV. Due to the overlapping of Co LMM and Fe LMM Auger emission features, the peak deconvolution of some spectra is not performed. The peak deconvolution results are showed in **Table S5**.


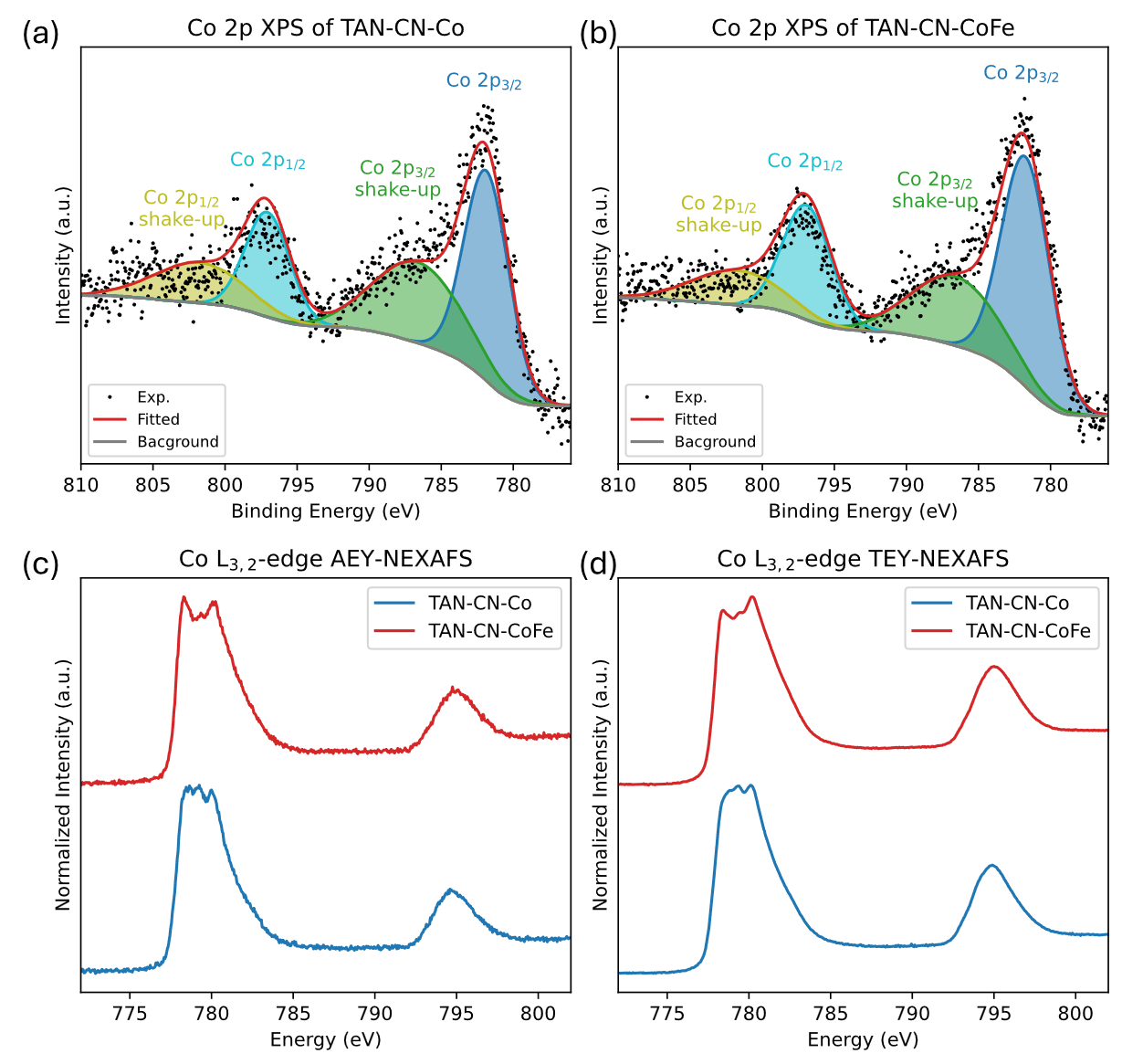


**Figure S18.** Synchrotron XPS and NEXAFS results. (a) Co 2p XPS spectra of Tan-CN-Co and the peak deconvolution results. (b) Co 2p XPS spectra of Tan-CN-CoFe and the peak deconvolution results. These XPS spectra are collected using excitation photon energy of 1430 eV at the ISISS beamline of BESSY II. The Peak deconvolution results are showed in **Table S5**. (c) Co L_3,2_-edge AEY NEXAFS of Tan-CN-Co and Tan-CN-CoFe. (c) Co L_3,2_-edge TEY NEXAFS of Tan-CN-Co and Tan-CN-CoFe.


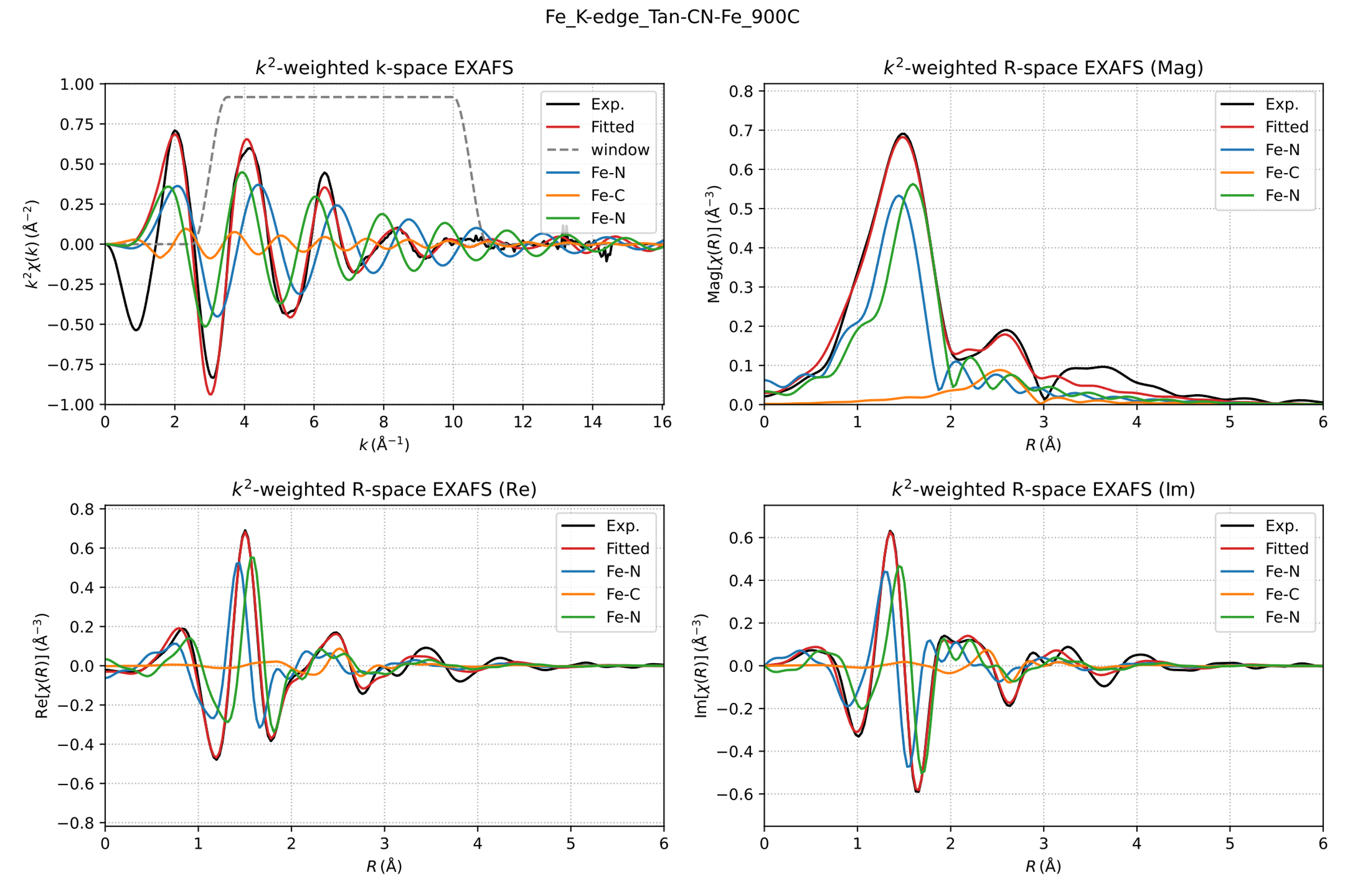


**Figure S19.** Fitting results of Fe K-edge *k*^2^-weighted k-space and R-space FT-EXAFS spectra of Tan-CN-Fe in (a) k-space, (b) R-space magnitude, (c) R-space real part and (d) R-space imaginary part. The R-space spectra are plotted without phase correction.


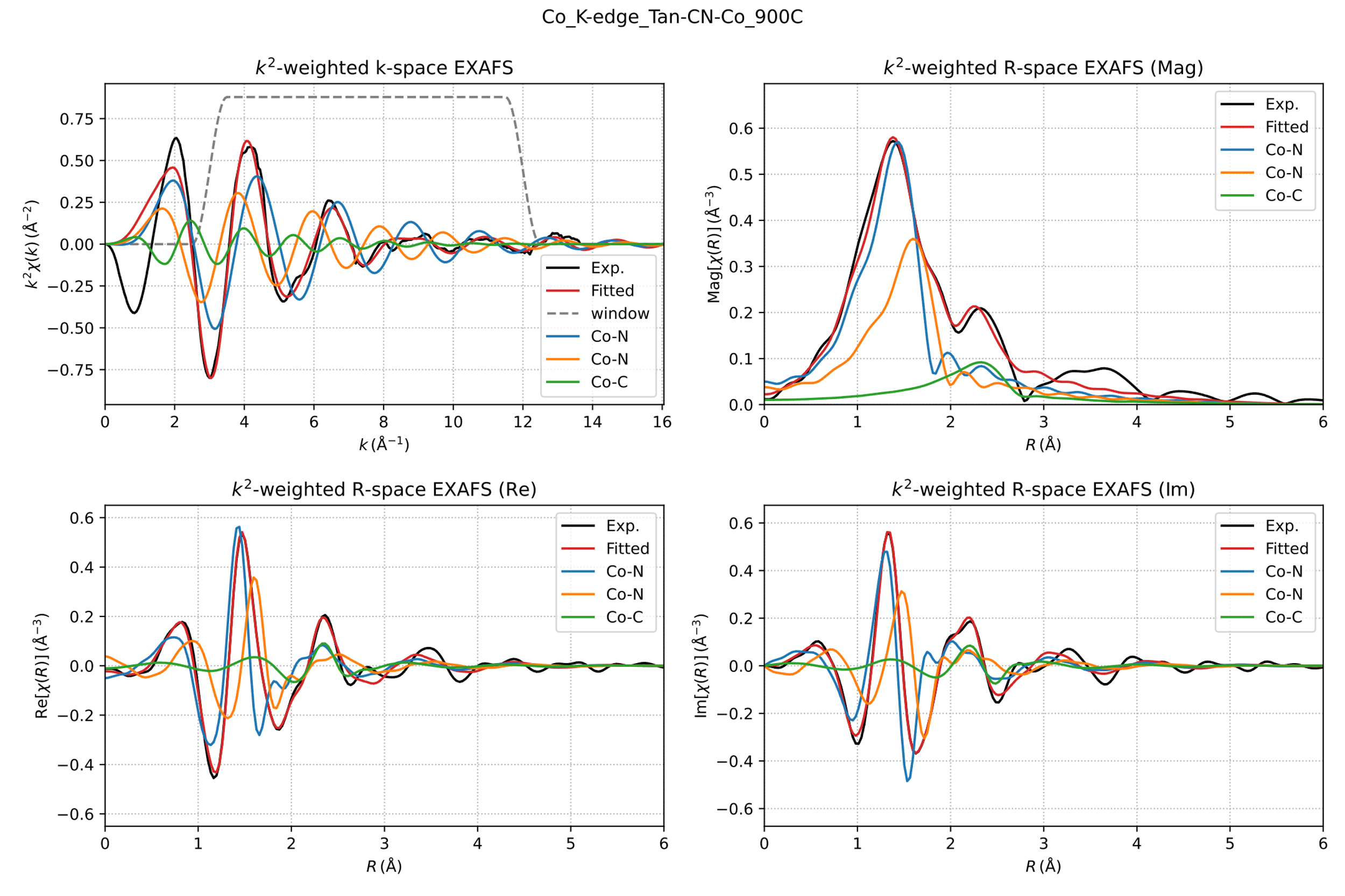
re

**Figure S20.** Fitting results of Co K-edge *k*^2^-weighted k-space and R-space FT-EXAFS spectra of Tan-CN-Co in (a) k-space, (b) R-space magnitude, (c) R-space real part and (d) R-space imaginary part. The R-space spectra are plotted without phase correction.


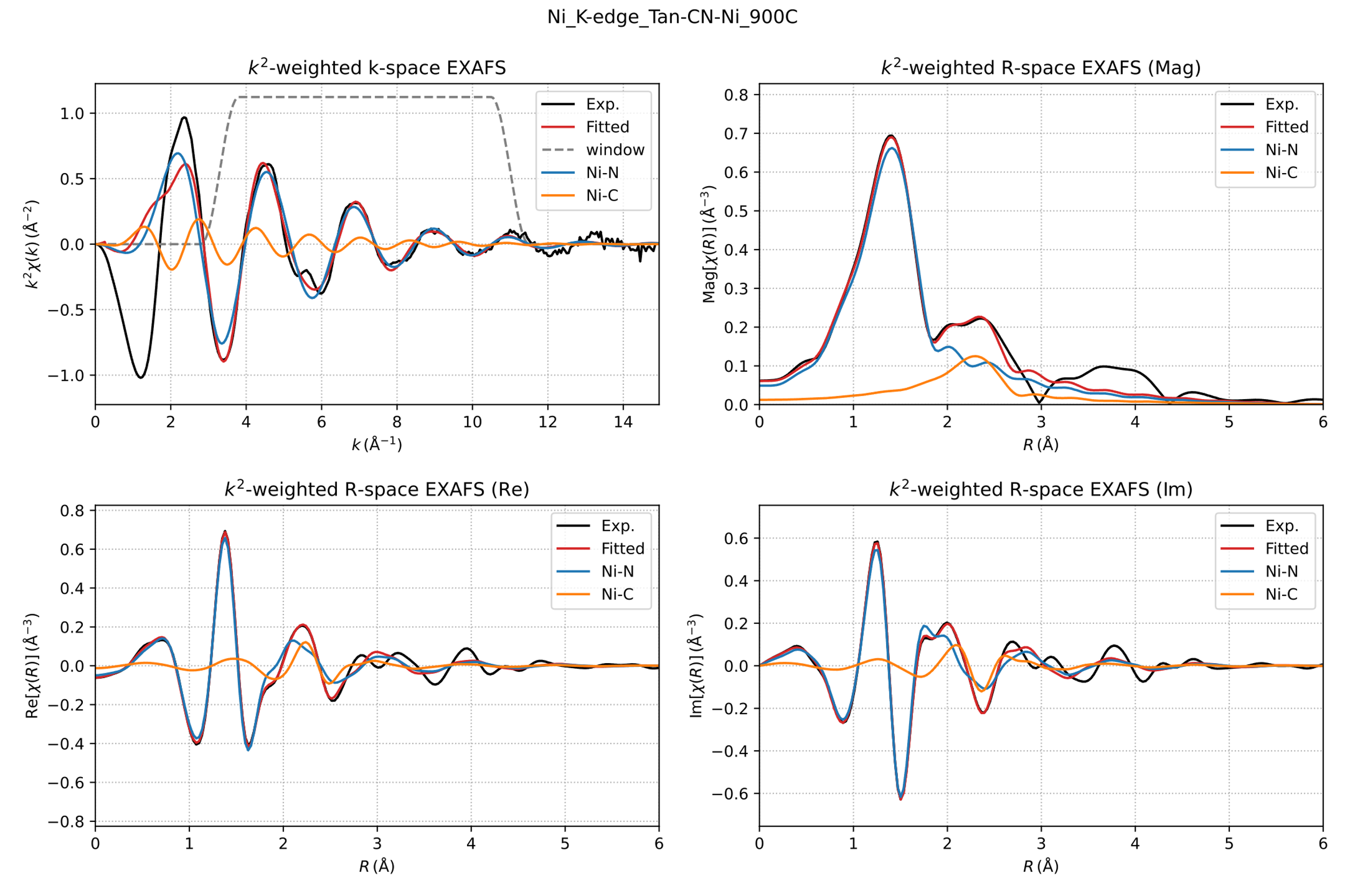


**Figure S21.** Fitting results of Ni K-edge *k*^2^-weighted k-space and R-space FT-EXAFS spectra of Tan-CN-Ni in (a) k-space, (b) R-space magnitude, (c) R-space real part and (d) R-space imaginary part. The R-space spectra are plotted without phase correction.


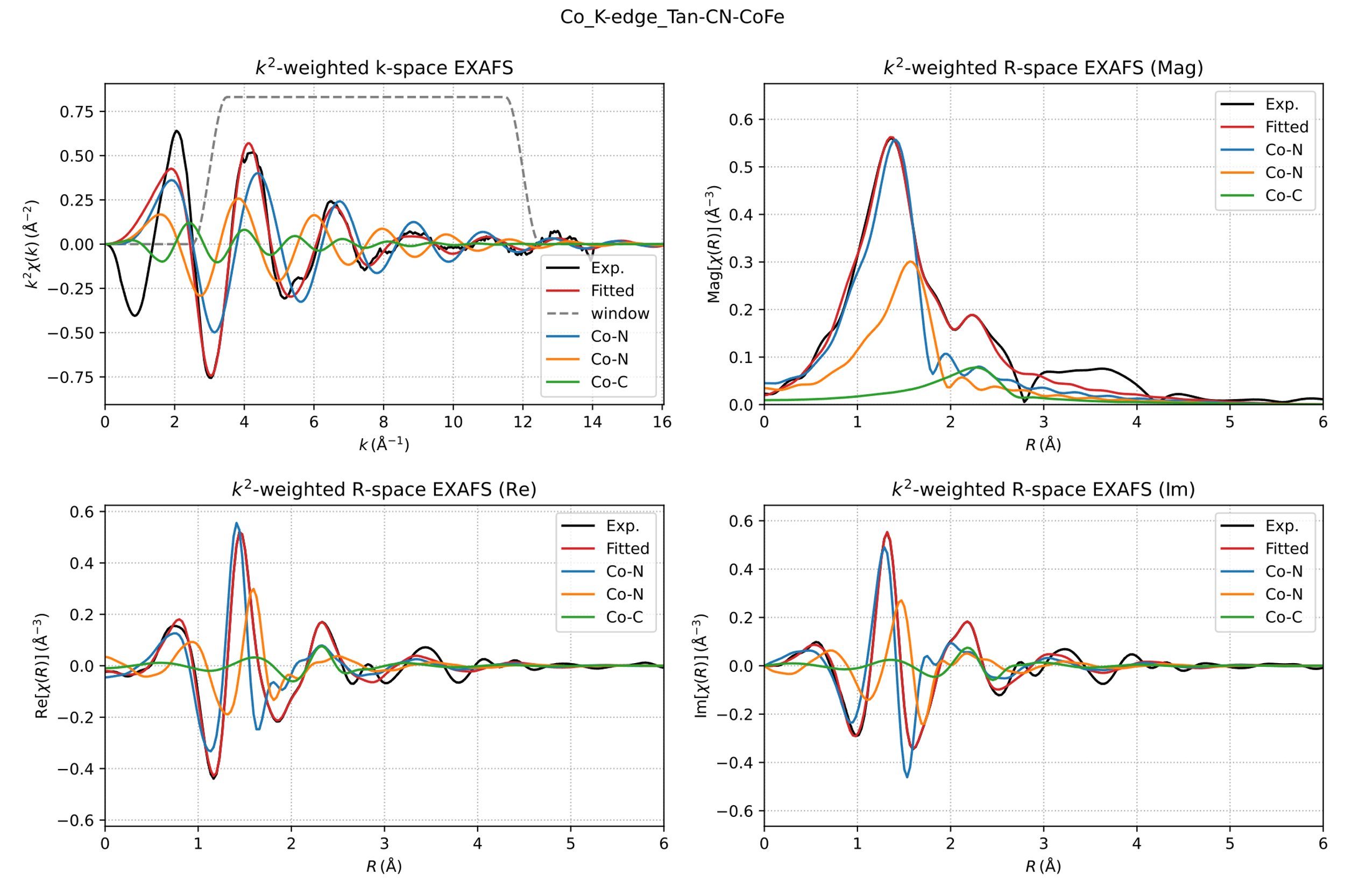


**Figure S22.** Fitting results of Co K-edge *k*^2^-weighted k-space and R-space FT-EXAFS spectra of Tan-CN-CoFe in (a) k-space, (b) R-space magnitude, (c) R-space real part and (d) R-space imaginary part. The R-space spectra are plotted without phase correction.


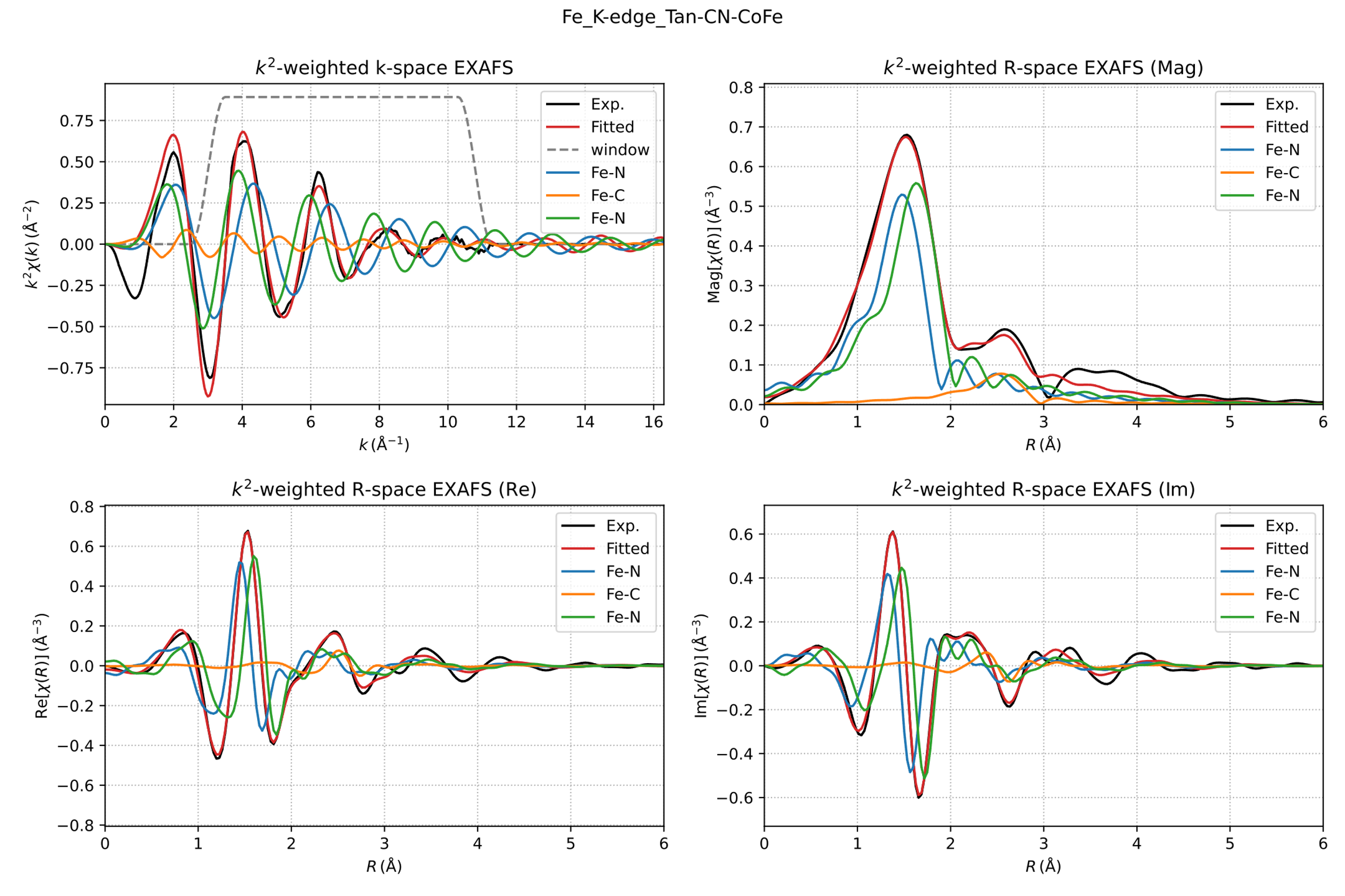


**Figure S23.** Fitting results of Fe K-edge *k*^2^-weighted k-space and R-space FT-EXAFS spectra of Tan-CN-CoFe in (a) k-space, (b) R-space magnitude, (c) R-space real part and (d) R-space imaginary part. The R-space spectra are plotted without phase correction.


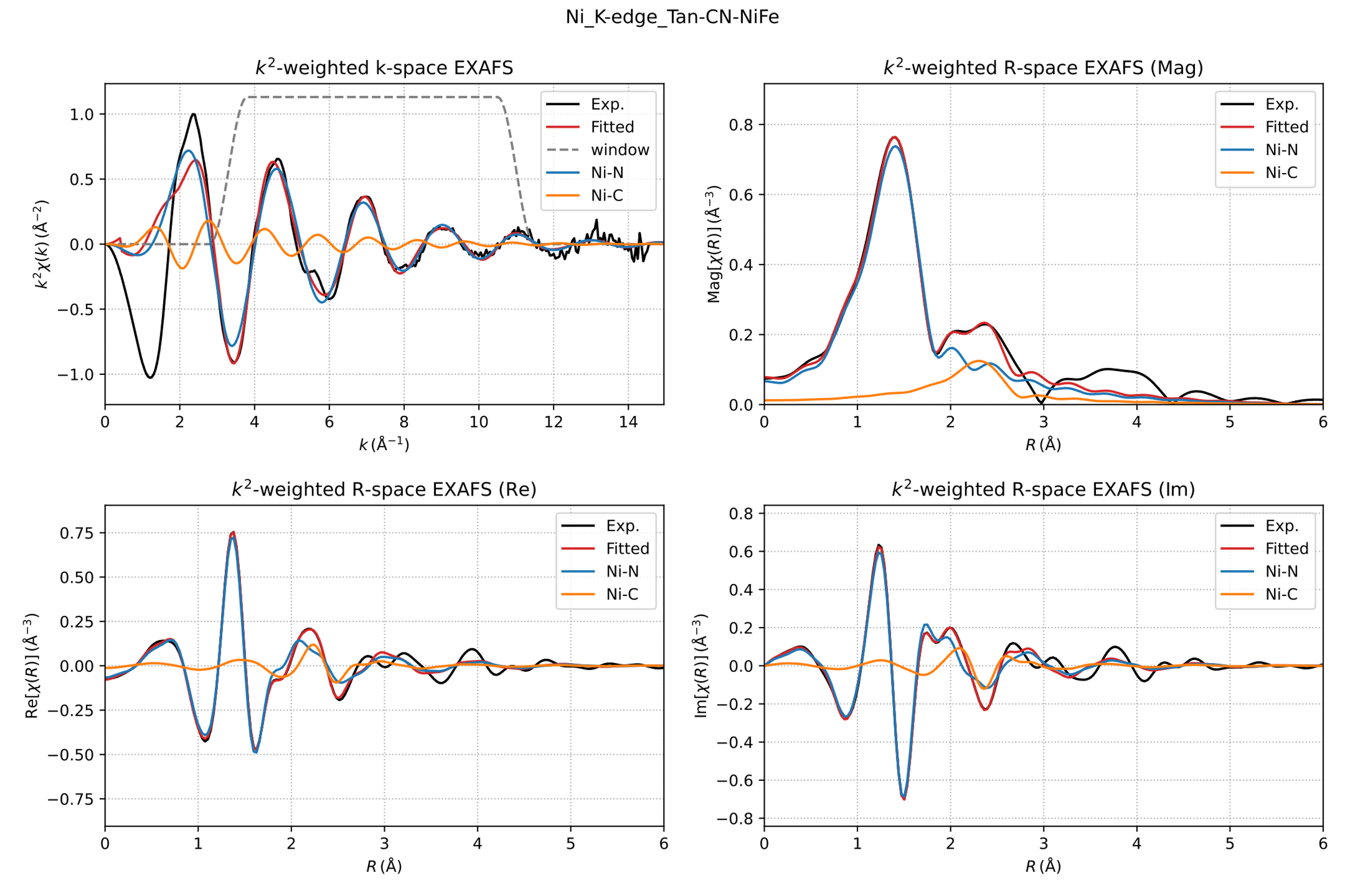


**Figure S24.** Fitting results of Ni K-edge *k*^2^-weighted k-space and R-space FT-EXAFS spectra of Tan-CN-NiFe in (a) k-space, (b) R-space magnitude, (c) R-space real part and (d) R-space imaginary part. The R-space spectra are plotted without phase correction.


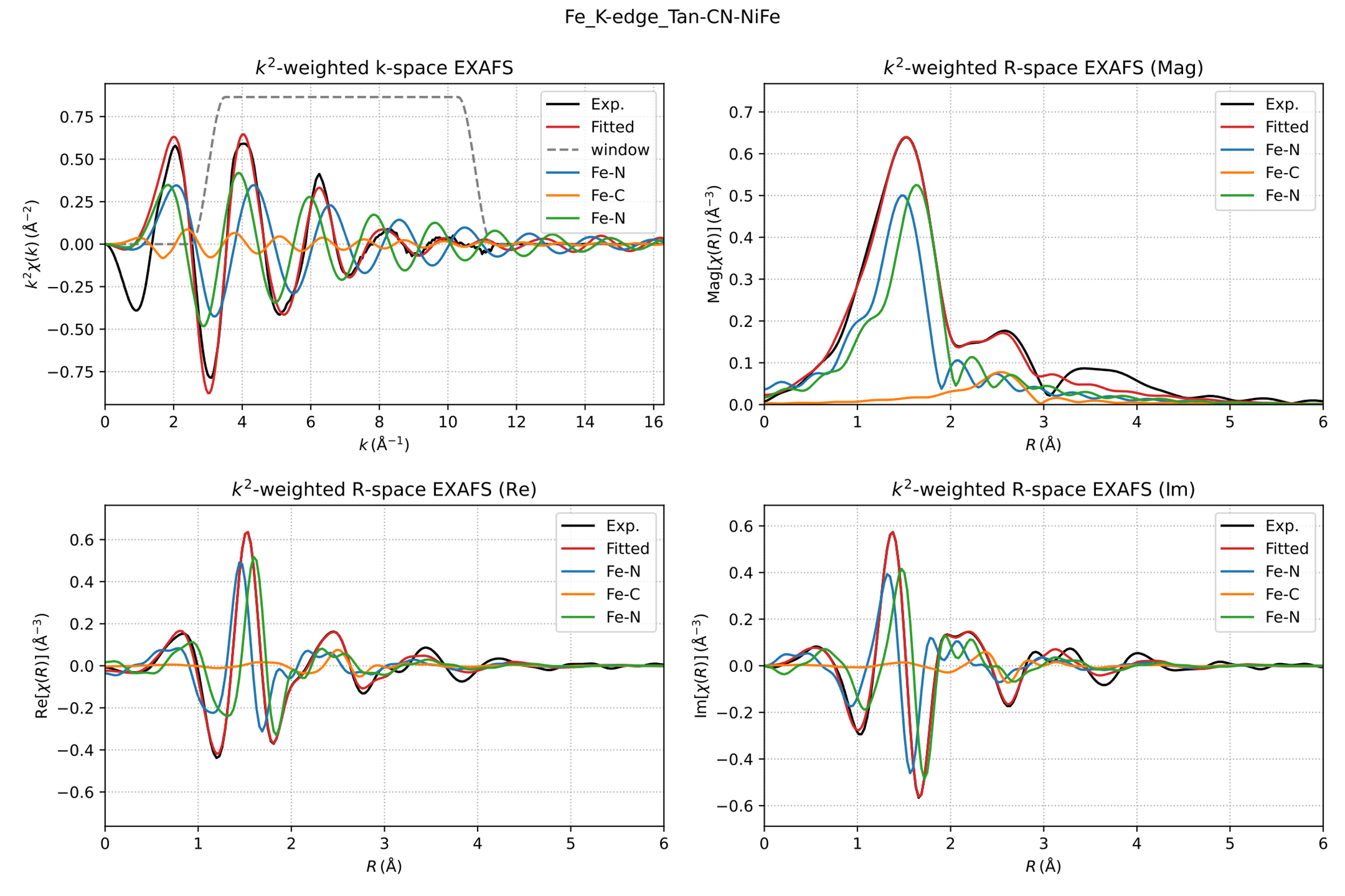


**Figure S25.** Fitting results of *k*^2^-weighted k-space and R-space FT-EXAFS spectra of Tan-CN-NiFe Fe K-edge in (a) k-space, (b) R-space magnitude, (c) R-space real part and (d) R-space imaginary part. The R-space spectra are plotted without phase correction.


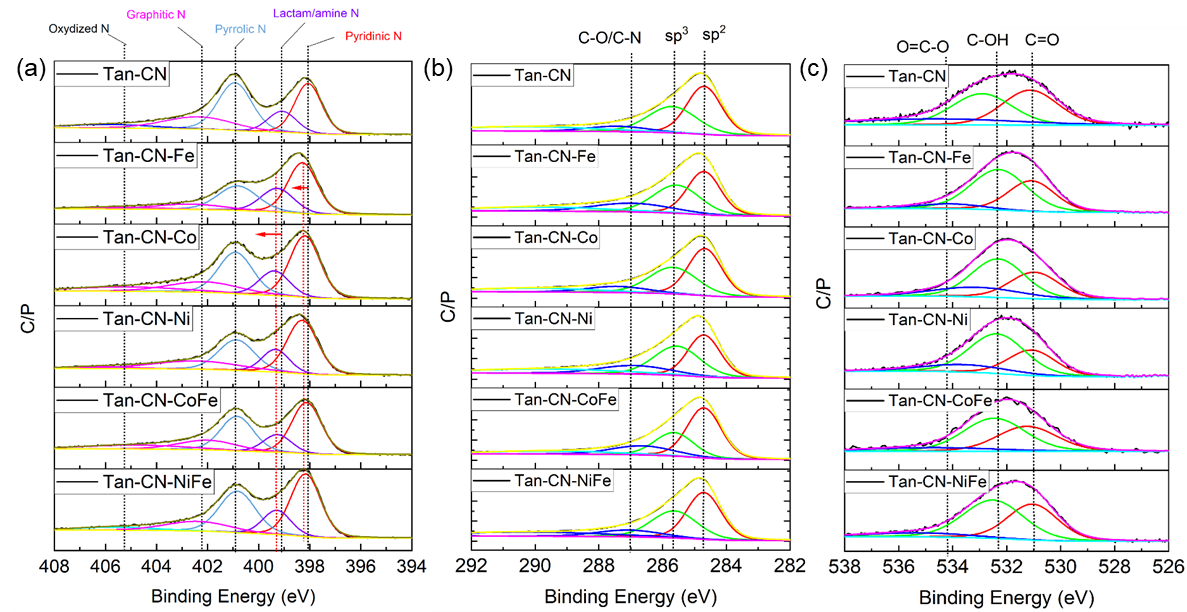


**Figure S26.** (a) N 1s (b) C 1s and (c) O 1s XPS spectra of Tan-CN-Fe, Tan-CN-Co, Tan-CN-Ni, Tan-CN-CoFe and Tan-CN-NiFe.


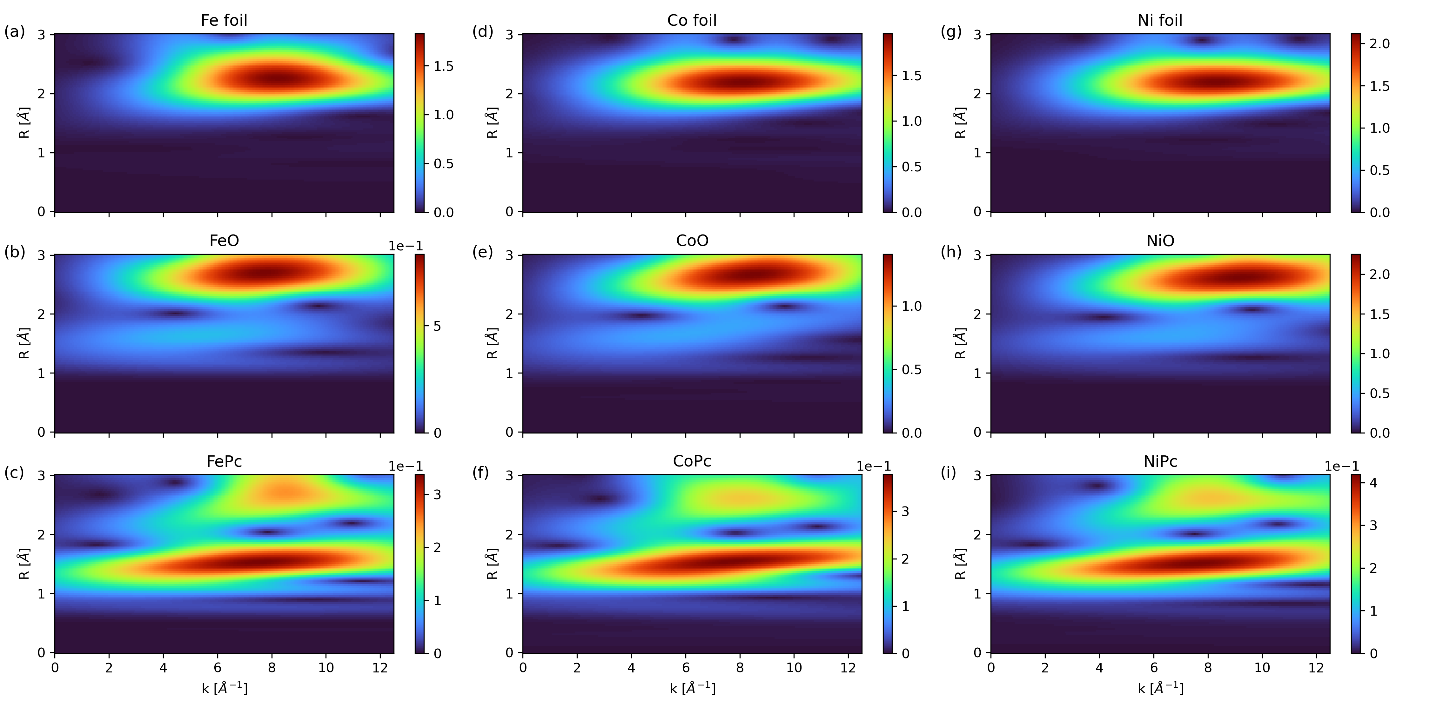


**Figure S27.** Wavelet transforms for the *k*^2^-weighted EXAFS signals of Fe, Co, and Ni in metal foil, metal oxide, and phthalocyanine (Pc) forms at (a-c) Fe K-edge, (d-f) Co K-edge and (g-i) Ni K-edge. Note: In the metal foils and metal oxides (a, b, d, e, g, h), the strongest feature near 8 Å^-1^ arises predominantly from second-shell single scattering (M-M or M-O-M), as these heavier scatterers at distances of 3~3.5 Å yield strong EXAFS oscillations in this k-range. By contrast, in the phthalocyanines (c, f, i), the intense peak at 8 Å^-1^ primarily originates from multiple-scattering pathways within the highly symmetric rigid planar macrocycle. The nearly linear arrangement of the central metal, nitrogen, and carbon atoms leads to constructive interference at higher k, enhancing the wavelet amplitude in this region.


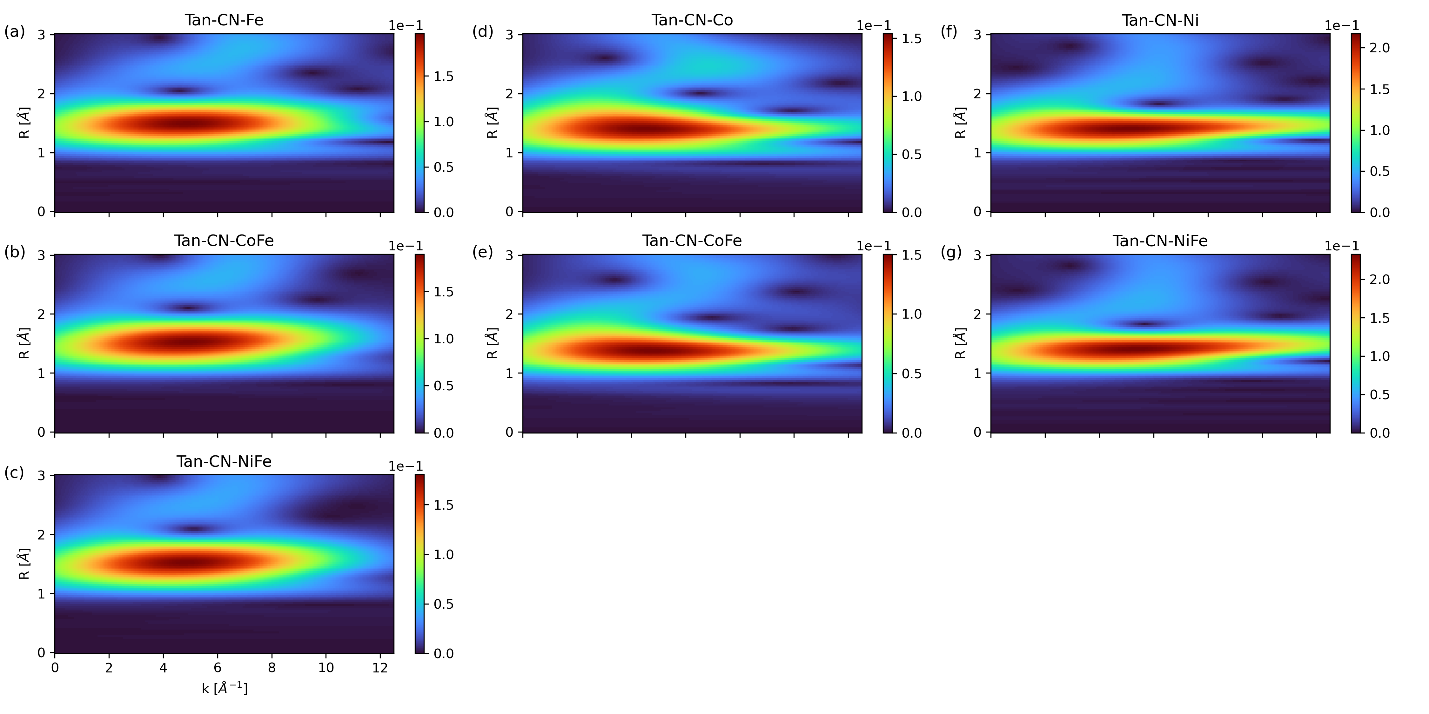


**Figure S 28.** Wavelet transforms for the *k*^2^-weighted EXAFS signals of Tan-CN-Fe, Tan-CN-Co, Tan-CN-Ni, Tan-CN-CoFe, and Tan-CN-NiFe at (a-c) Fe K-edge, (d-f) Co K-edge and (g-i) Ni K-edge.


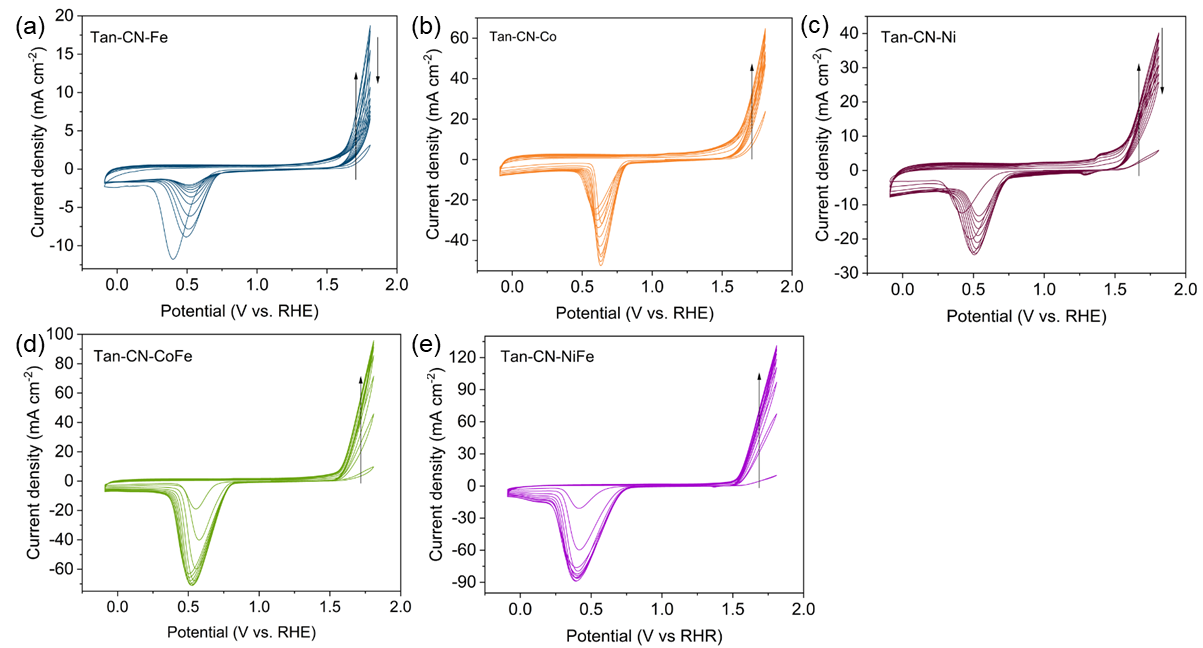


**Figure S29.** CV curves of the as-prepared catalysts on glassy carbon prior to LSV measurements.


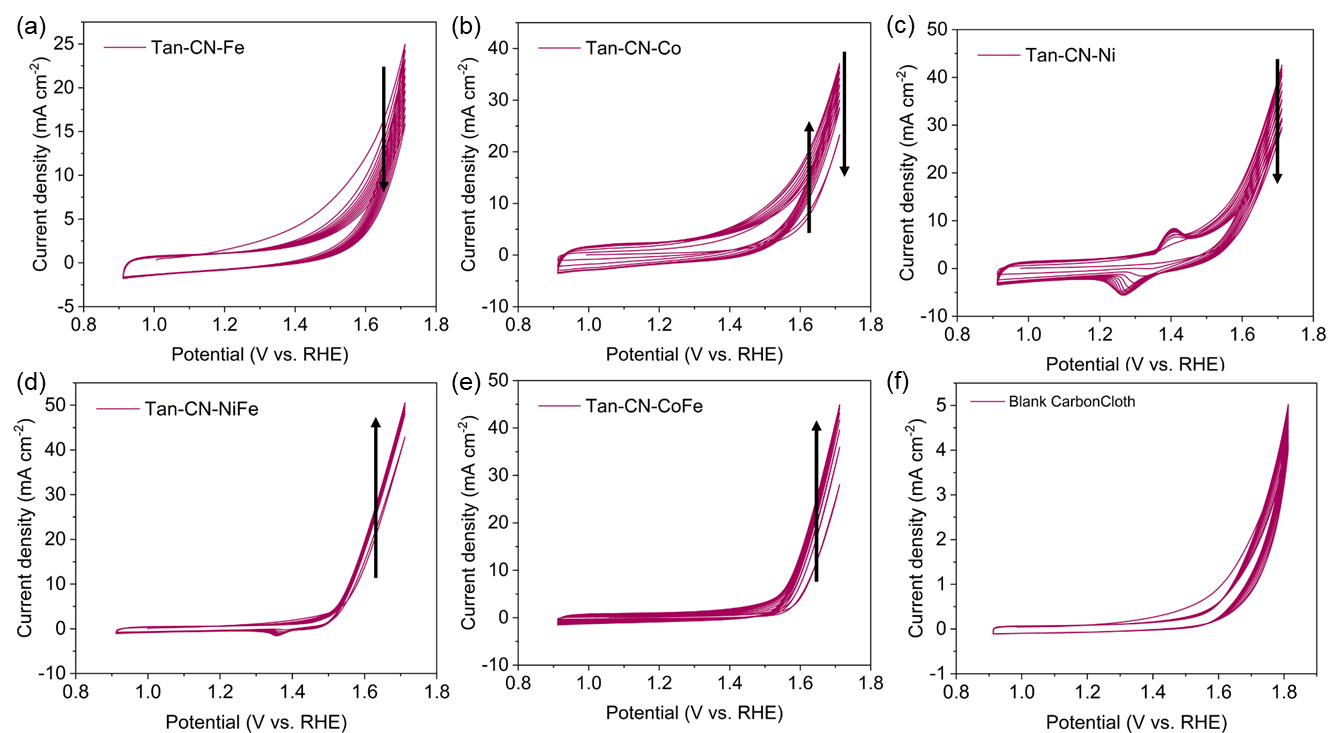


**Figure S30****.** CV curves of the as-prepared catalysts on carbon cloth.


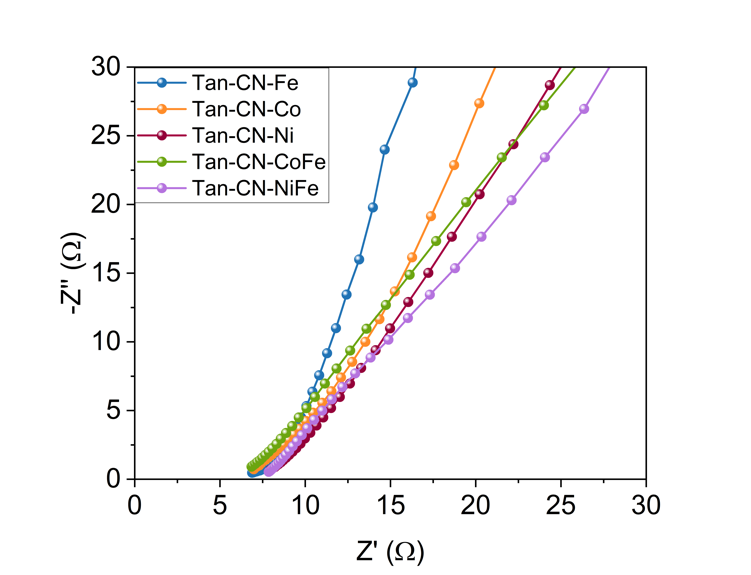


**Figure S31.** EIS measurements at open-circuit potential (OCP) for Tan-CN-Fe, Tan-CN-Co, Tan-CN-Ni, Tan-CN-CoFe and Tan-CN-NiFe SACs.


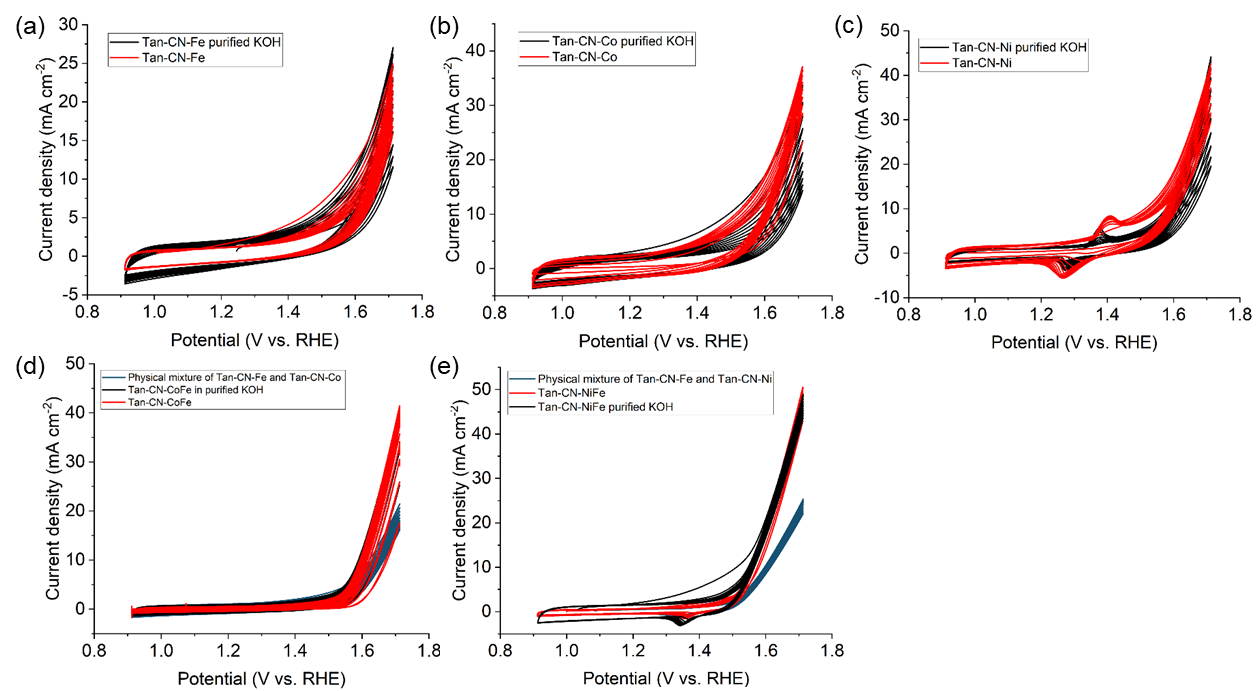


**Figure S32.** CV curves of Tan-CN-M series tested in commercial (red) and purified KOH (black) using the purification **method 1**, and comparison of CV curves of Tan-CN-CoFe, Tan-CN-NiFe, and the physical mixture of Tan-CN-Fe and Tan-CN-Co, Tan-CN-Ni.


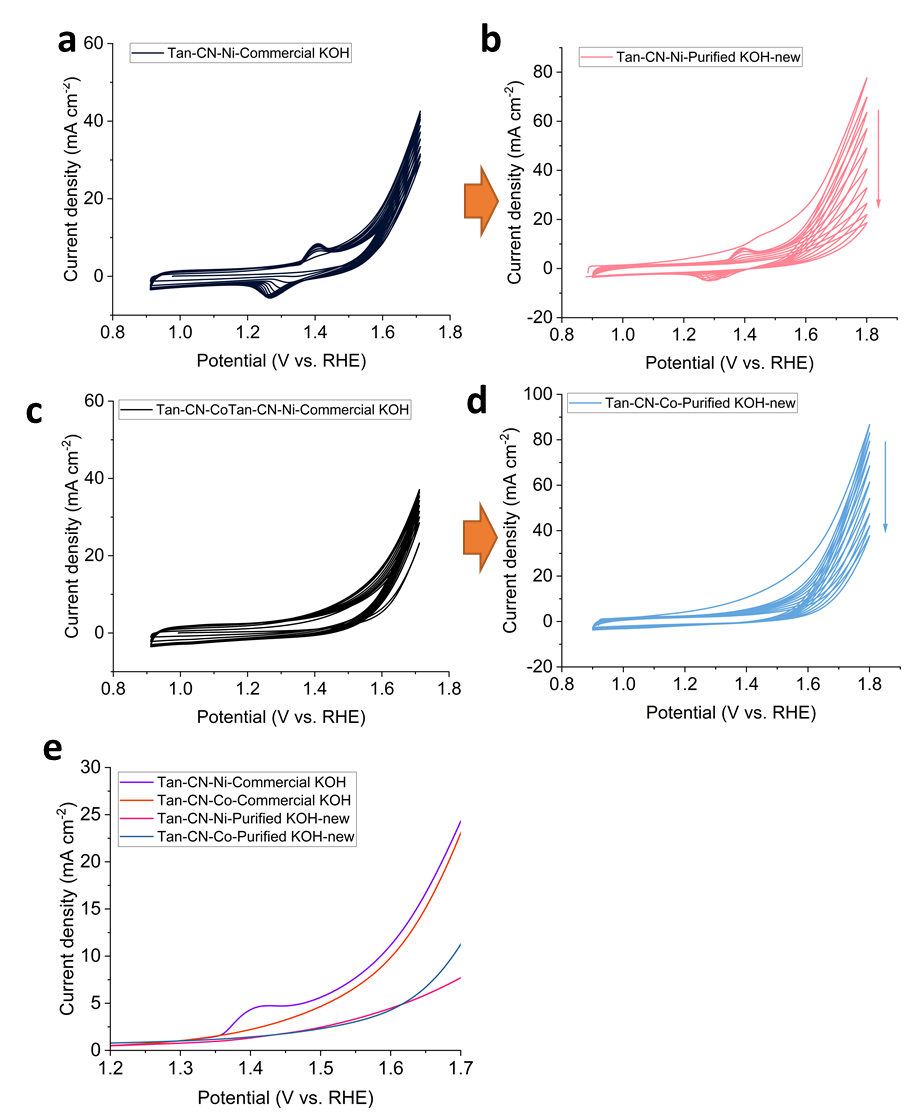


**Figure S33.** CV and LSV tests of the SACs in commercial 1 M KOH and purified 1 M KOH using purification **method 2**.


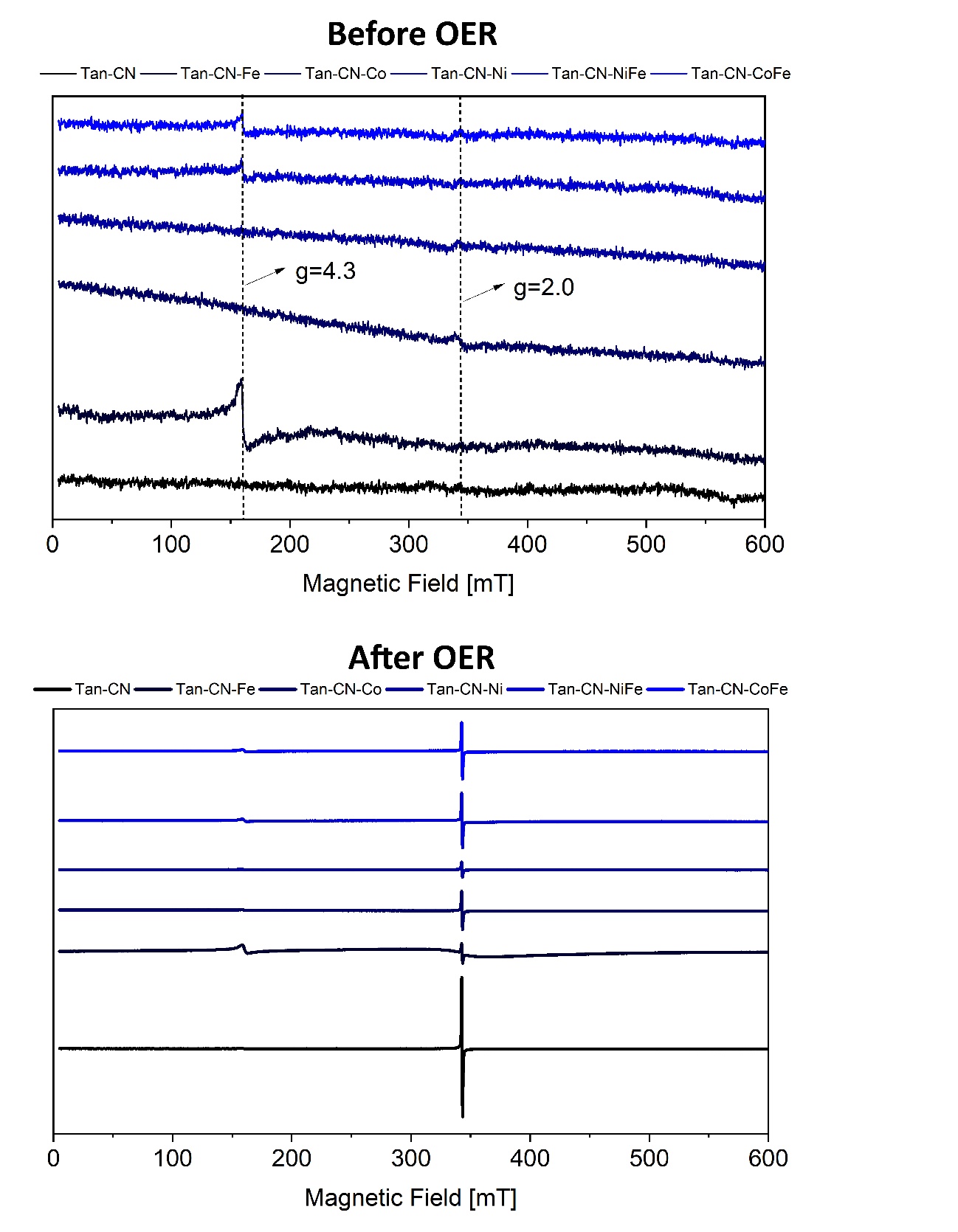


**Figure S34.** CW X-band EPR spectra of undoped Tan-CN, Tan-CN-Fe/Co/Ni/NiFe/CoFe before (top) and after OER (bottom). Spectra are plotted offset for better visibility. Measurement parameters: *T* = 10 K, MW frequency: 9.64 GHz, MW power: 2 mW, modulation amplitude: 0.7 mT.


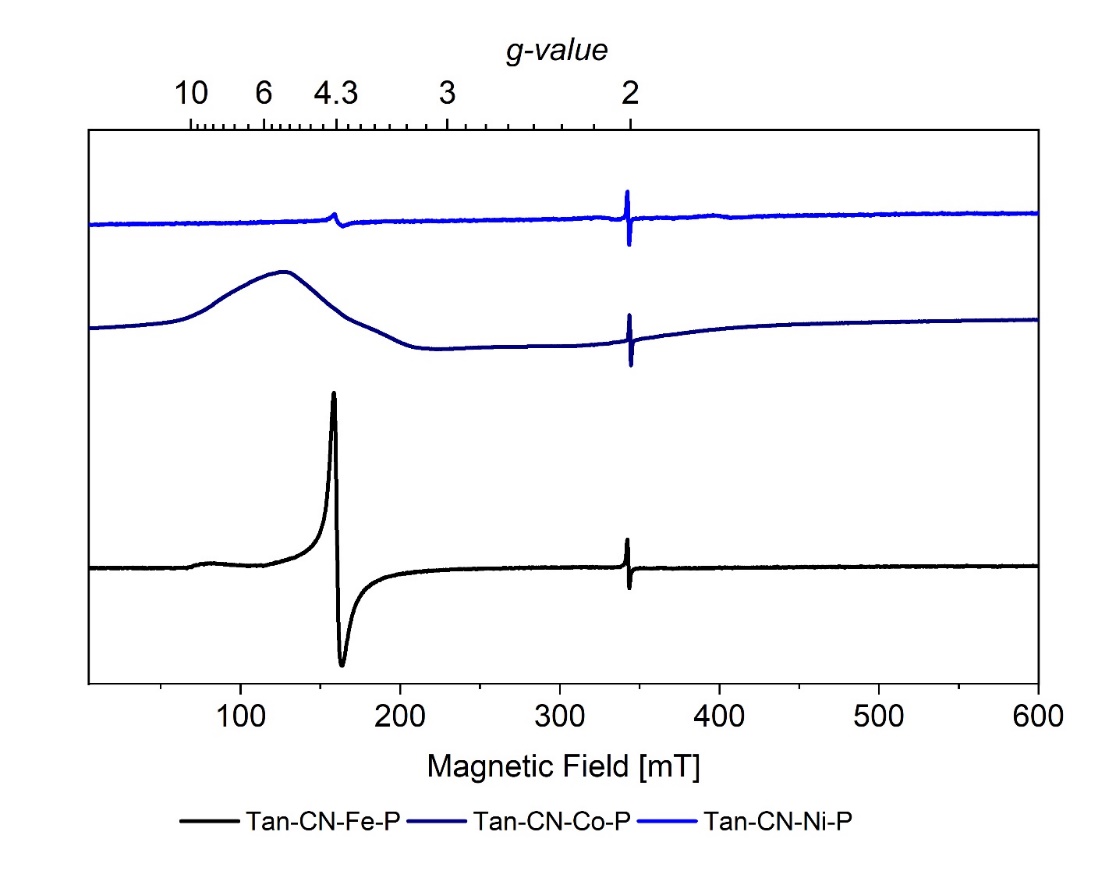


**Figure S35**. CW X-band EPR spectra of the precursors Tan-CN-Fe-P (lower), Tan-CN-Co-P (middle) and Tan-CN-Ni-P (top). Spectra are plotted offset for better visibility. Measurement parameters: T = 10 K, MW frequency: 9.64 GHz, MW power: 2 mW, modulation amplitude: 0.7 mT (Tan-CN-Fe-P and Tan-CN-Ni-P) and T = 5 K, MW frequency: 9.58 GHz, MW power: 20 mW, modulation amplitude: 0.7 mT (Tan-CN-Co-P).


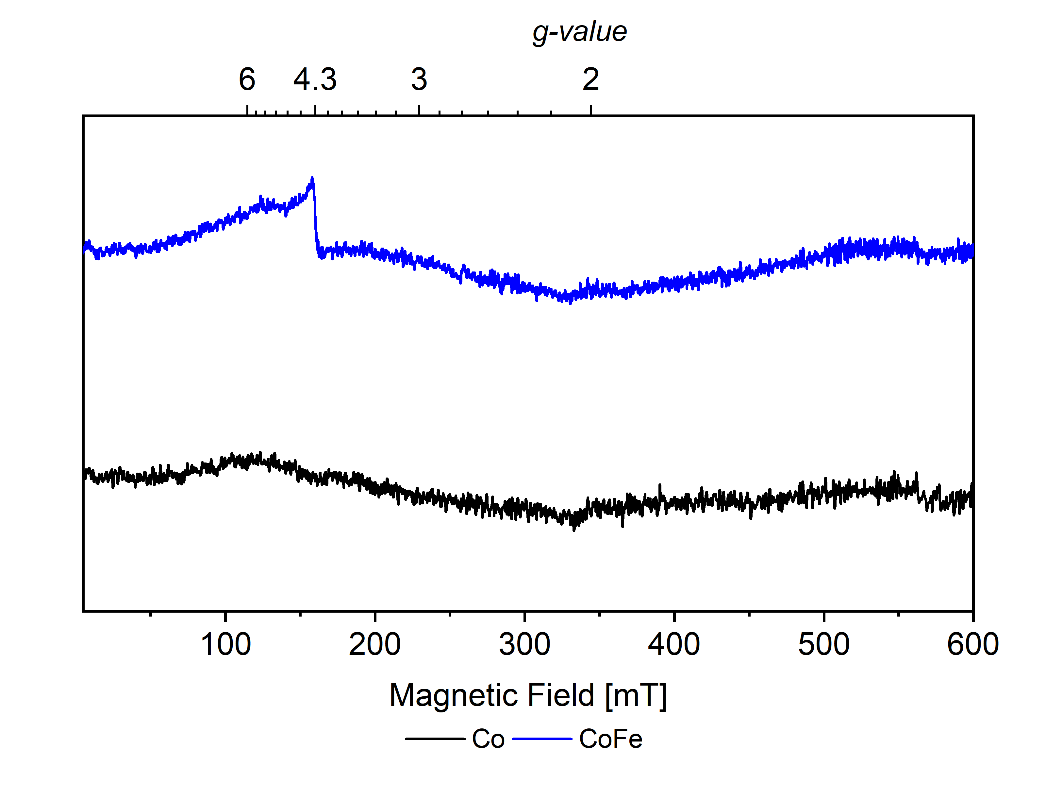


**Figure S36**. CW X-band EPR spectra of the precursors Tan-CN-Co and Tan-CN-CoFe. Measurement parameters: *T* = 5 K, MW frequency: 9.58 GHz, MW power: 20 mW, modulation amplitude: 0.7 mT.


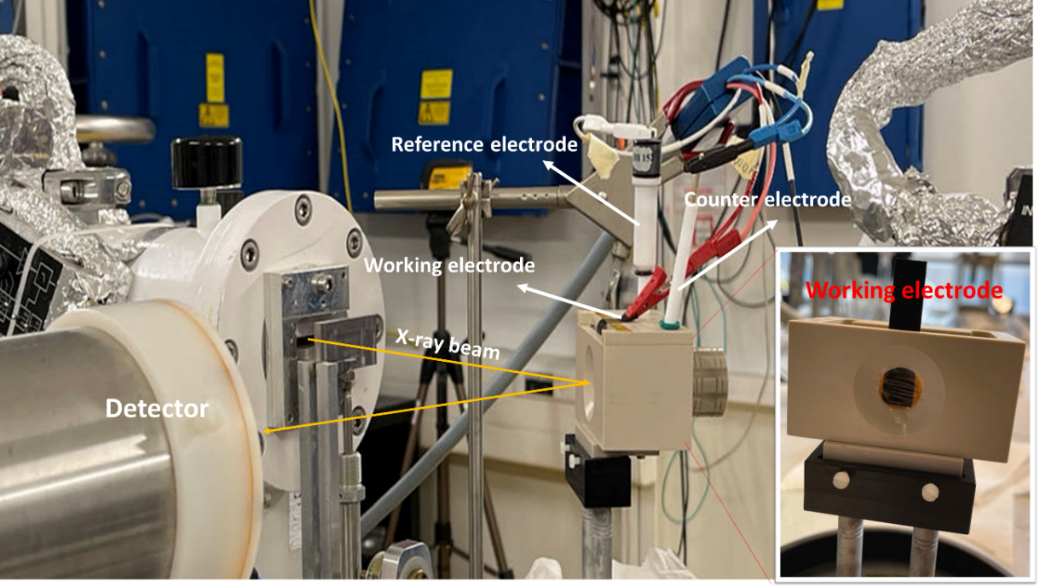


**Figure S****37.** Picture of the experiment setup for *in situ* XAS measurements.


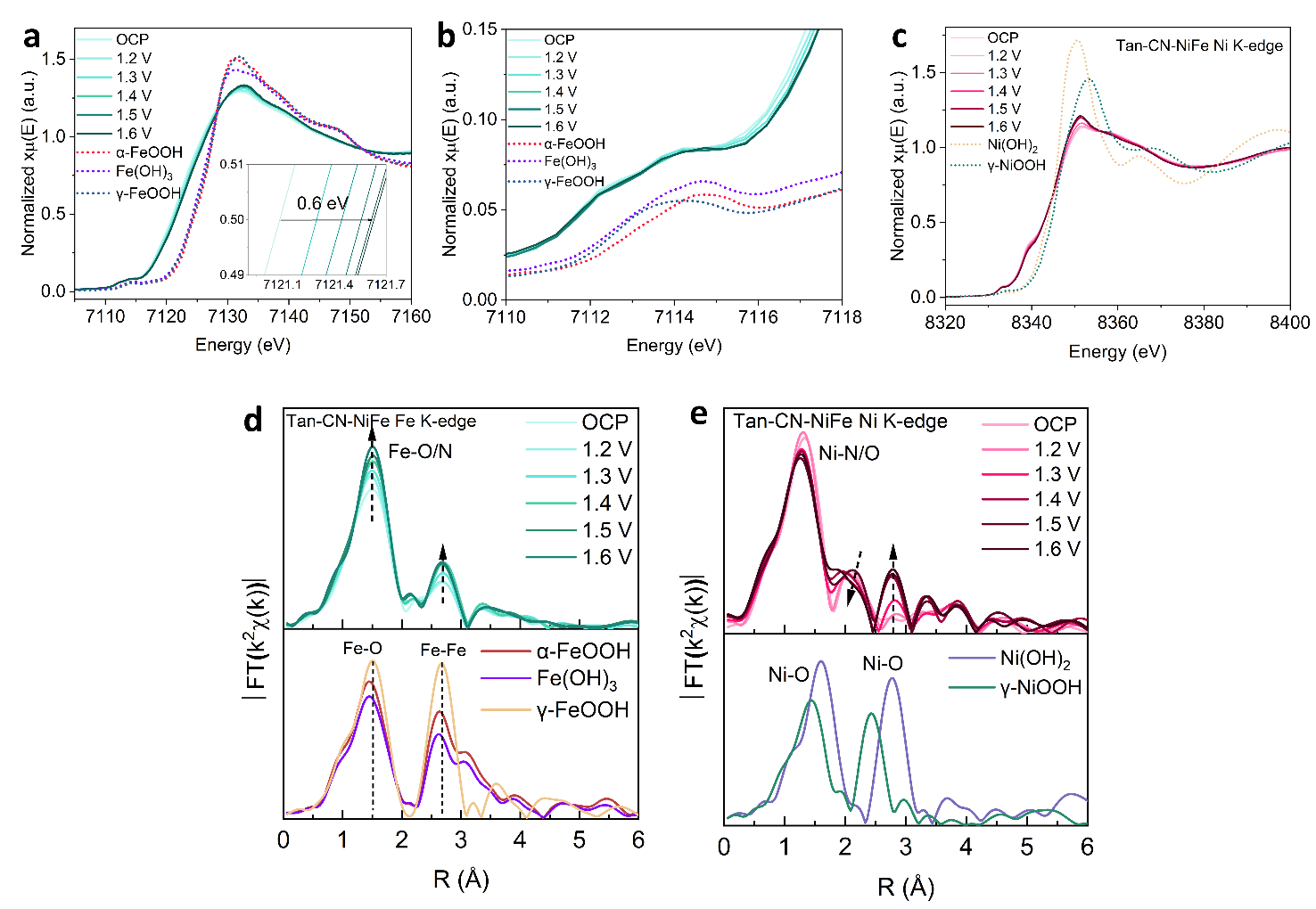


**Figure S38.** *In situ* XANES and EXAFS for Tan-CN-NiFe catalyst under different applied potentials: (a) Fe K-edge XANES, (b) the corresponding pre-edge spectra, (c) Ni K-edge XANES, (d) Fe K-edge k^2^-weighted R-space FT- EXAFS, (e) Ni K-edge k^2^-weighted R-space FT- EXAFS. The EXAFS spectra are plotted without phase correction.


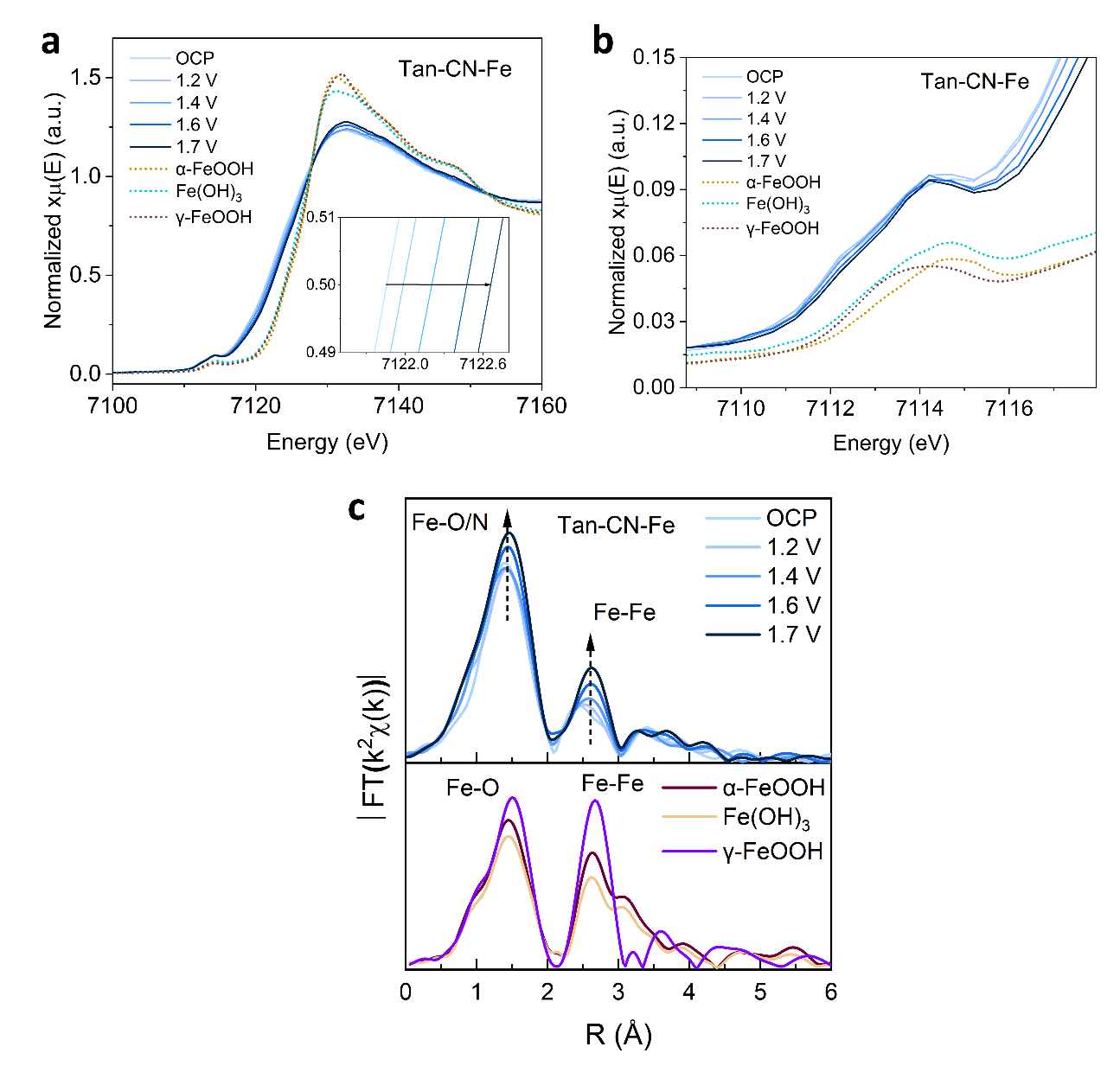


**Figure S****39.** *In situ* Fe K-edge (a) XANES, (b) Pre-edge, and (c) EXAFS for Tan-CN-Fe catalyst under different applied potential. The EXAFS spectra are plotted without phase correction.


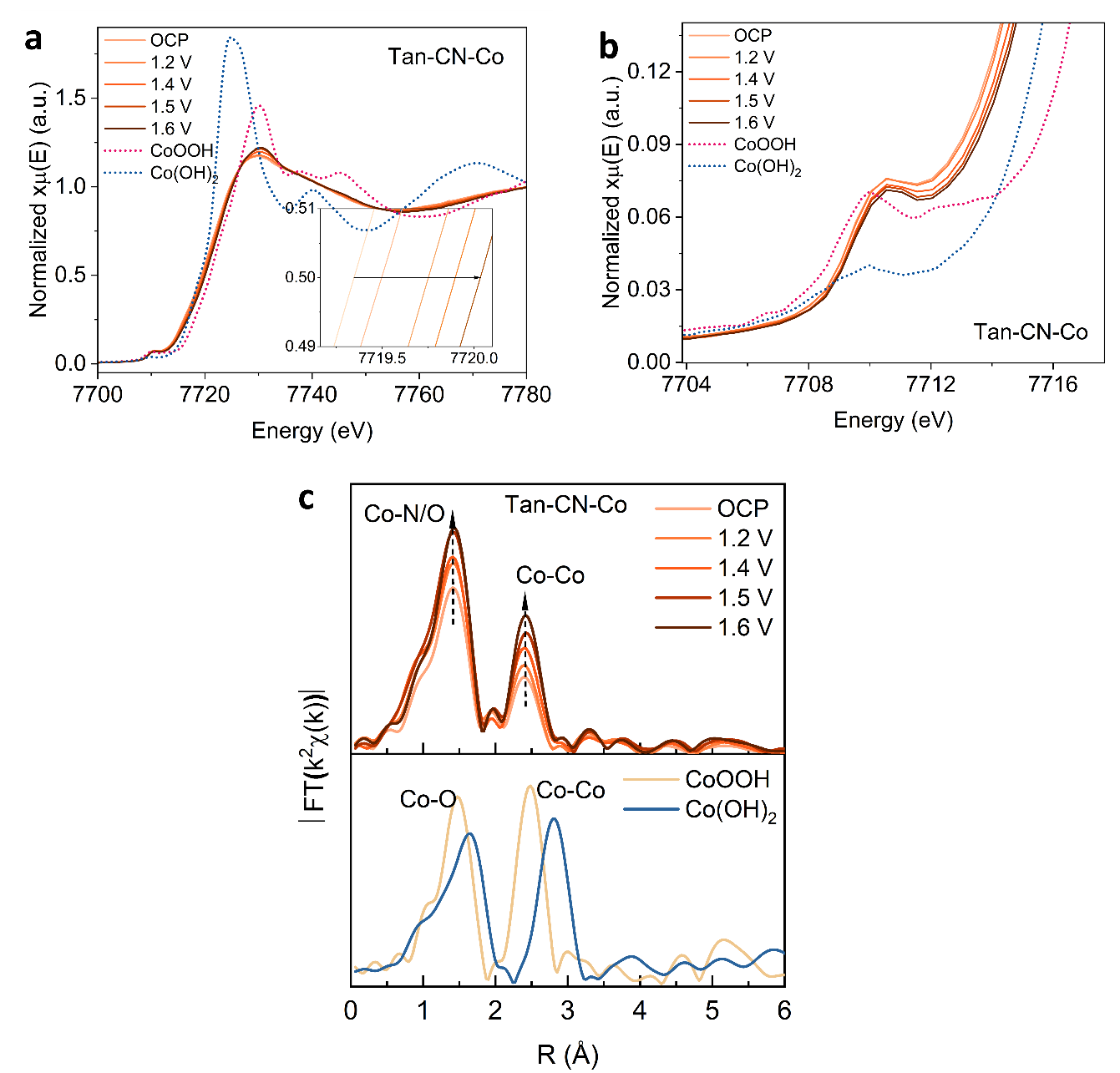


**Figure S40.** *In situ* Co K-edge (a) XANES, (b) Pre-edge, and (c) EXAFS for Tan-CN-Co catalyst under different applied potential. The EXAFS spectra are plotted without phase correction.


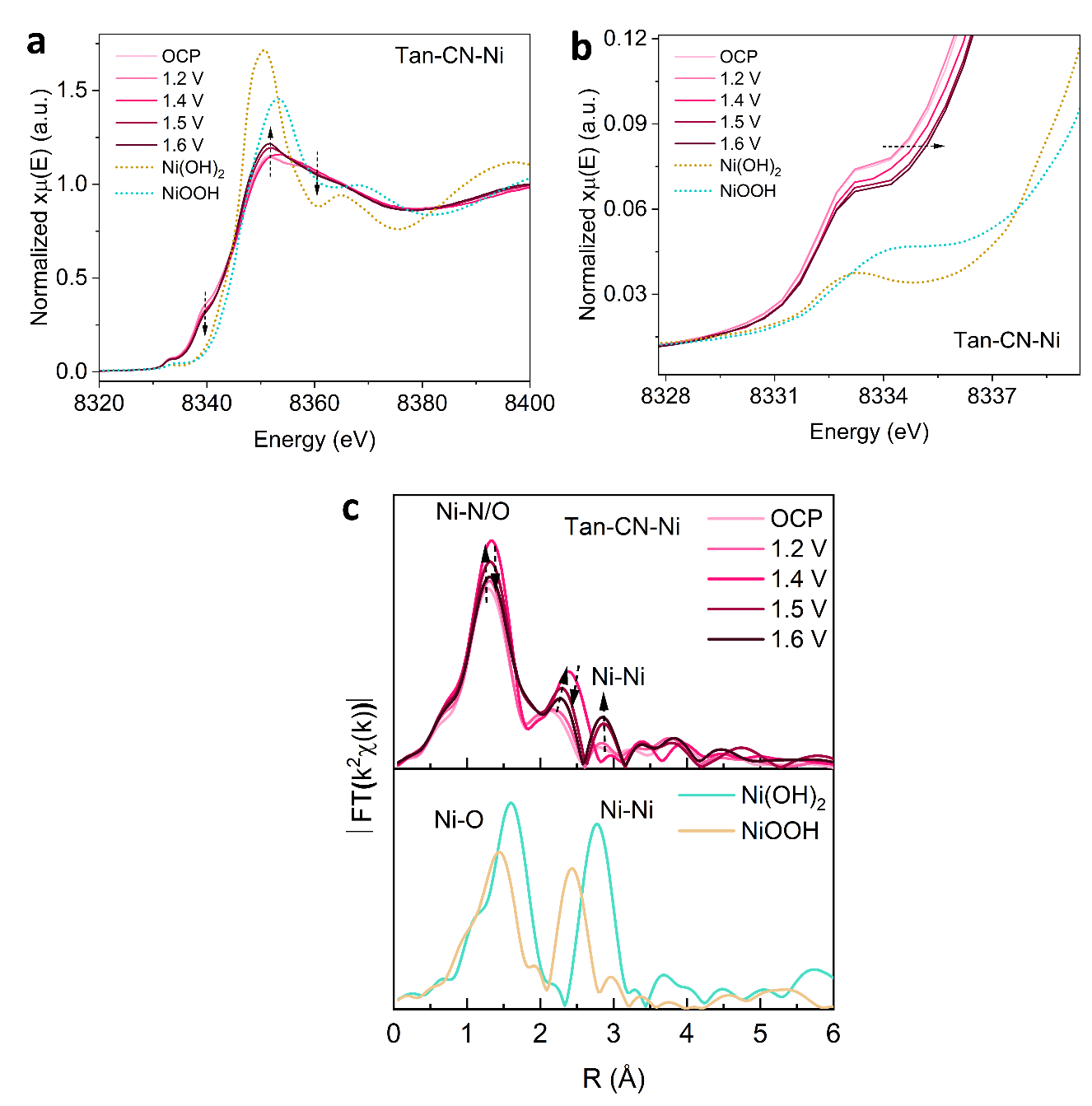


**Figure S41.** *In situ* Ni K-edge (a) XANES, (b) Pre-edge, and (c) EXAFS for Tan-CN-Ni catalyst under different applied potential. The EXAFS spectra are plotted without phase correction.


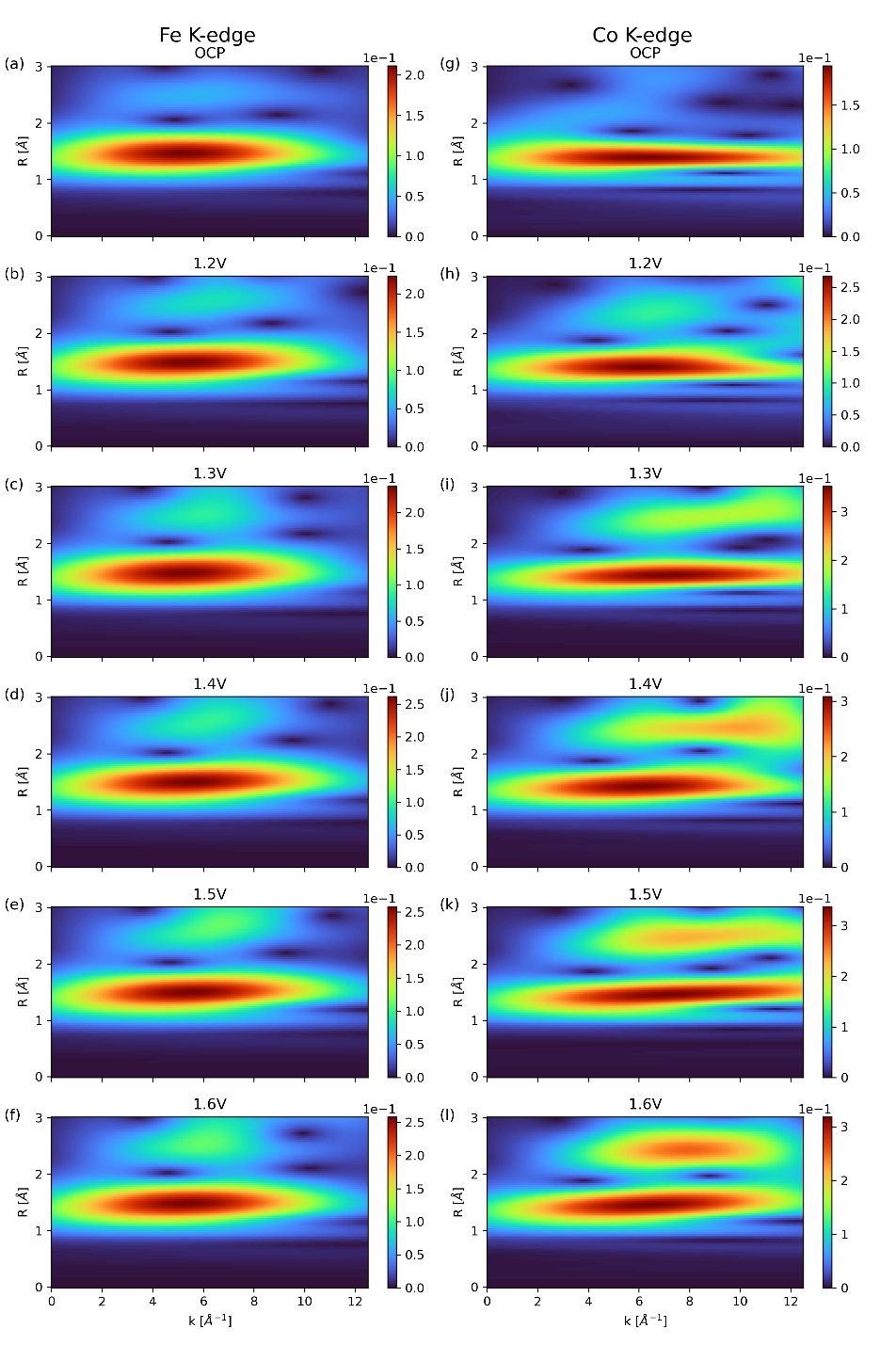


**Figure S42.** k^3^-weighted WT-EXAFS spectra of Tan-CN-CoFe at different operation potentials.


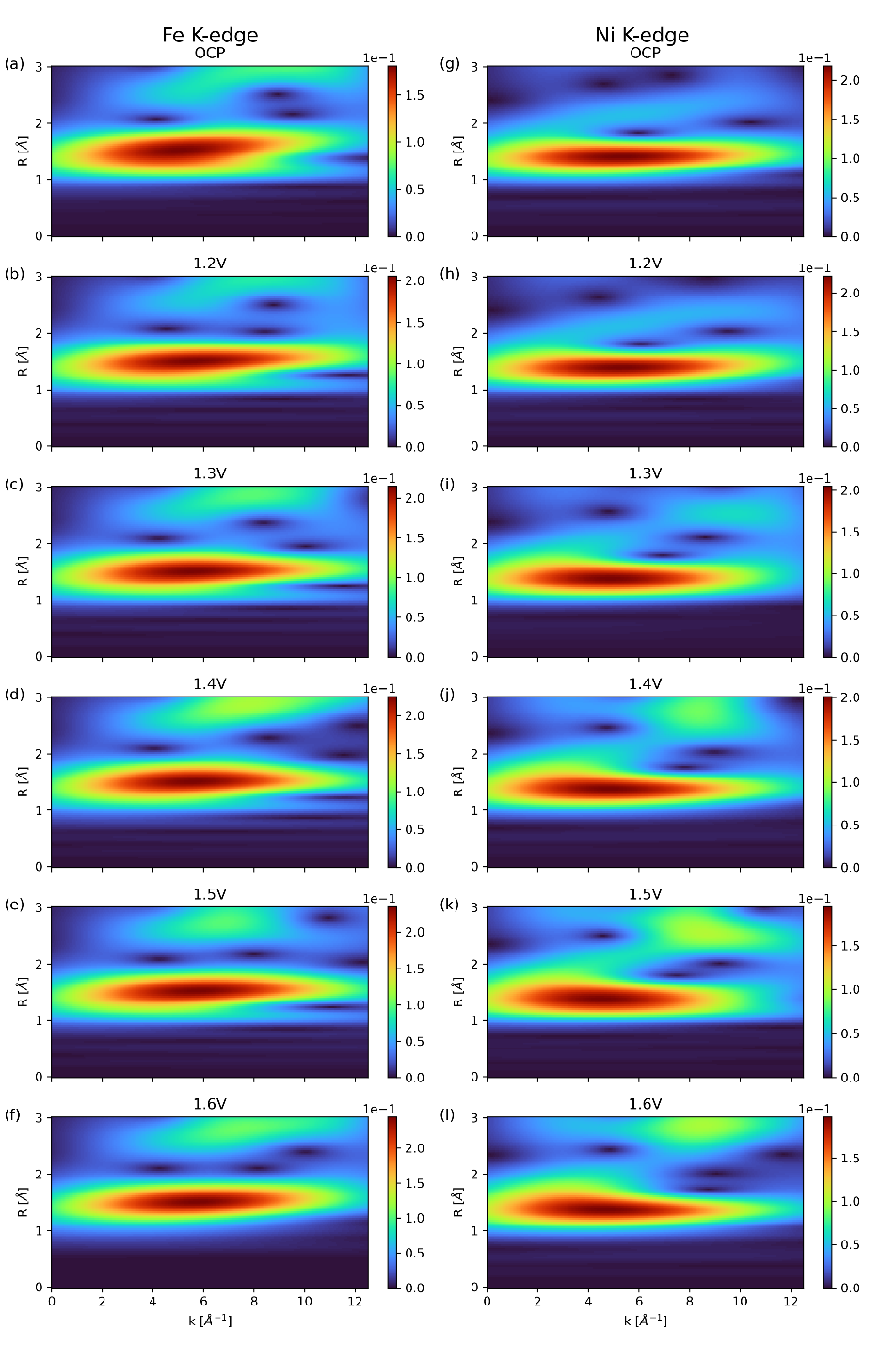


**Figure S43.** k^3^-weighted WT-EXAFS spectra of Tan-CN-NiFe at different operation potentials.


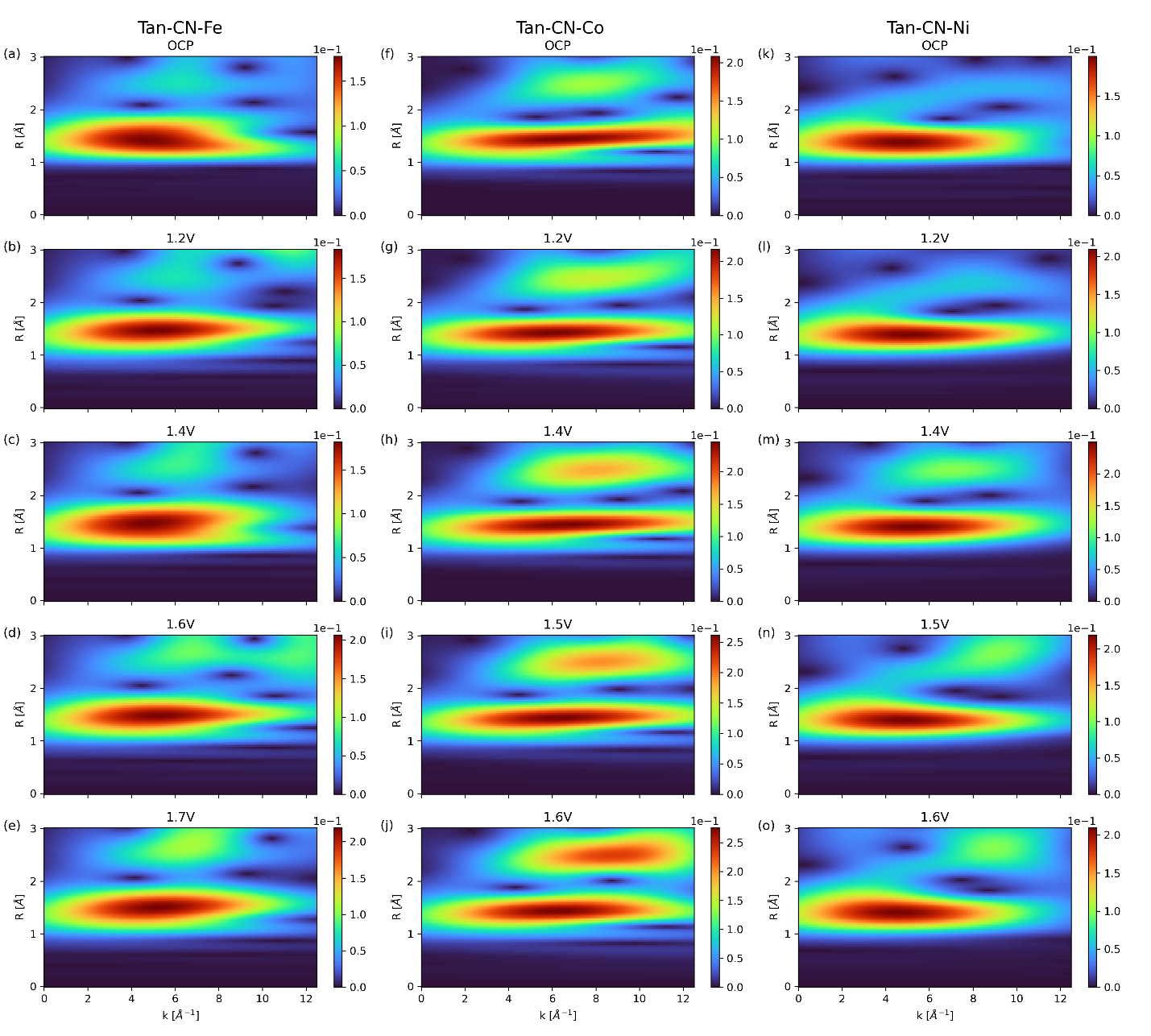


**Figure S44.** k^3^-weighted WT-EXAFS spectra of Tan-CN-Fe/Co/Ni at different operation potentials.


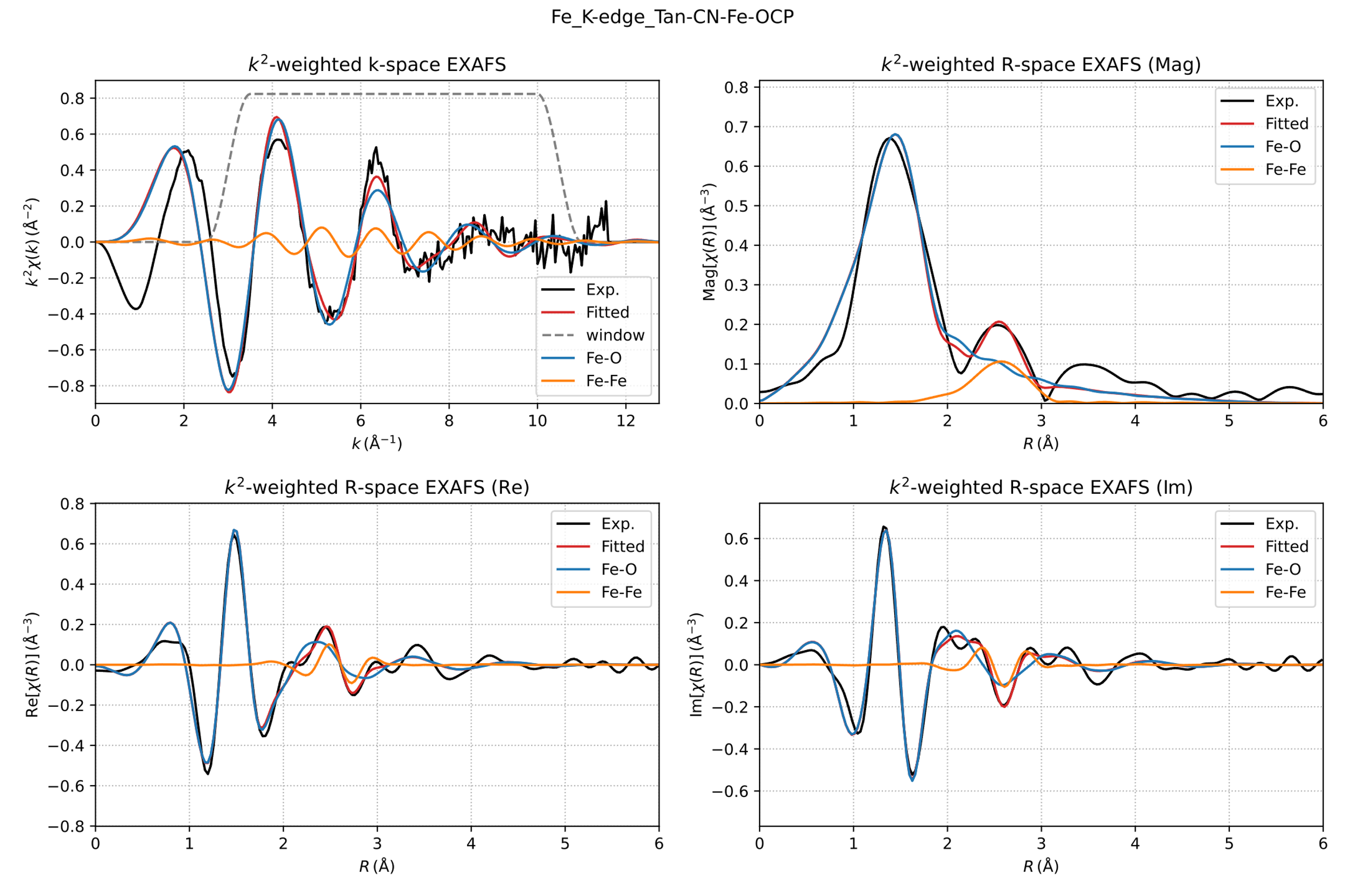


**Figure S45.** Fitting results of Fe K-edge *k*^2^-weighted k-space and R-space FT-EXAFS spectra of Tan-CN-Fe under OCP condition in (a) k-space, (b) R-space magnitude, (c) R-space real part and (d) R-space imaginary part. The R-space spectra are plotted without phase correction.


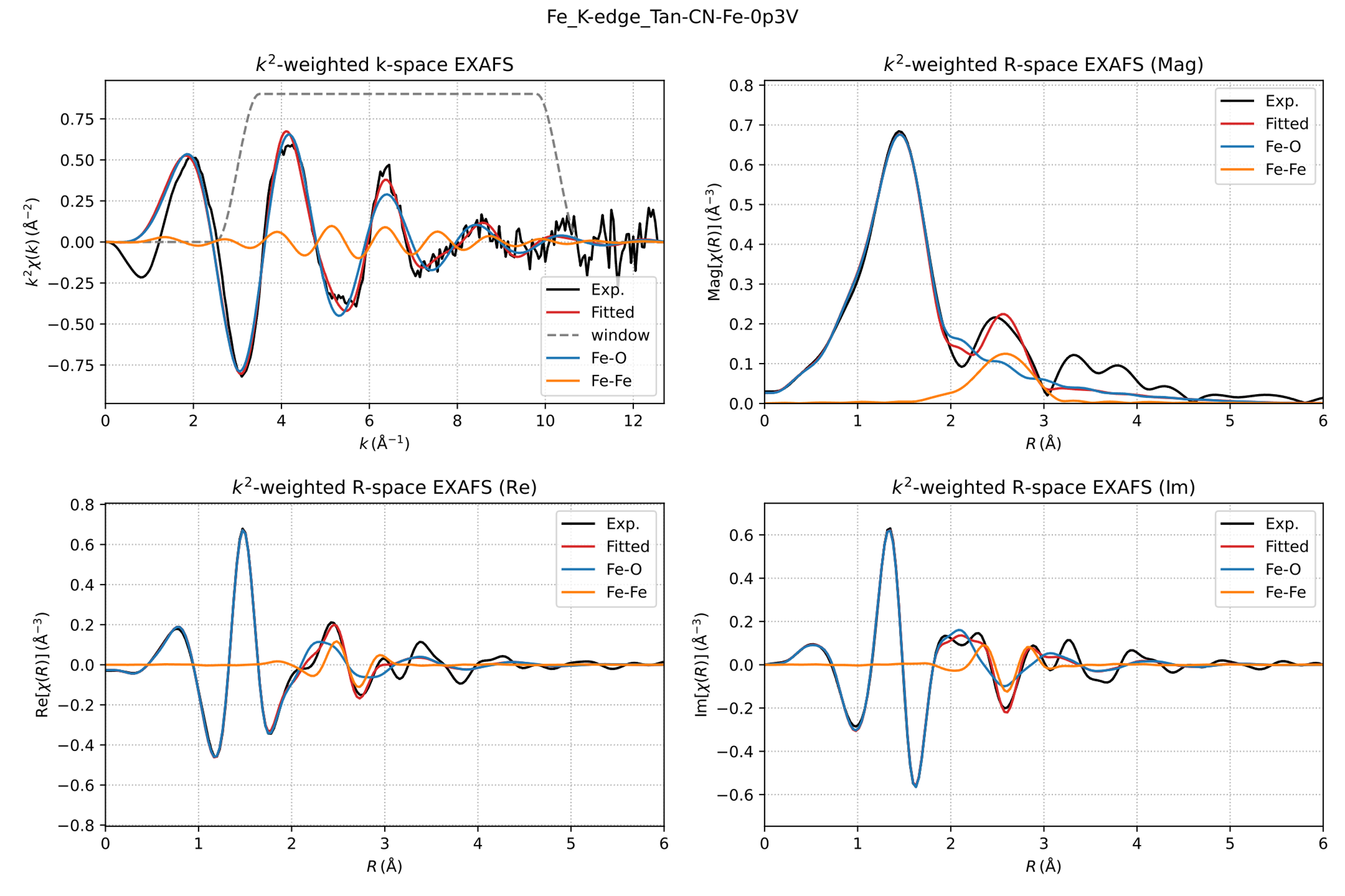


**Figure S46.** Fitting results of Fe K-edge *k*^2^-weighted k-space and R-space FT-EXAFS spectra of Tan-CN-Fe at 1.2 V vs. RHE in (a) k-space, (b) R-space magnitude, (c) R-space real part and (d) R-space imaginary part. The R-space spectra are plotted without phase correction.


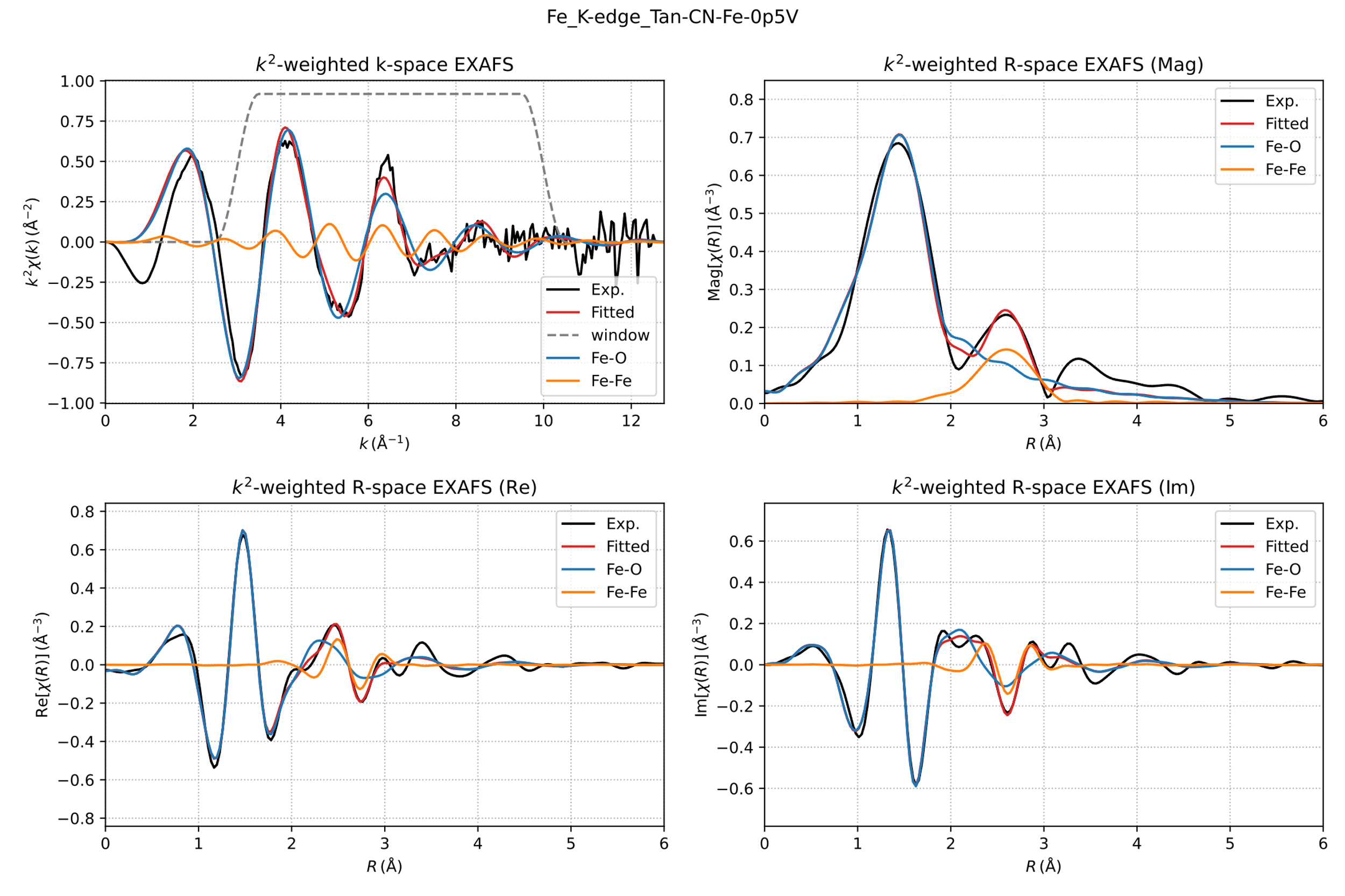


**Figure S47.** Fitting results of Fe K-edge *k*^2^-weighted k-space and R-space FT-EXAFS spectra of Tan-CN-Fe at 1.4 V vs. RHE in (a) k-space, (b) R-space magnitude, (c) R-space real part and (d) R-space imaginary part. The R-space spectra are plotted without phase correction.


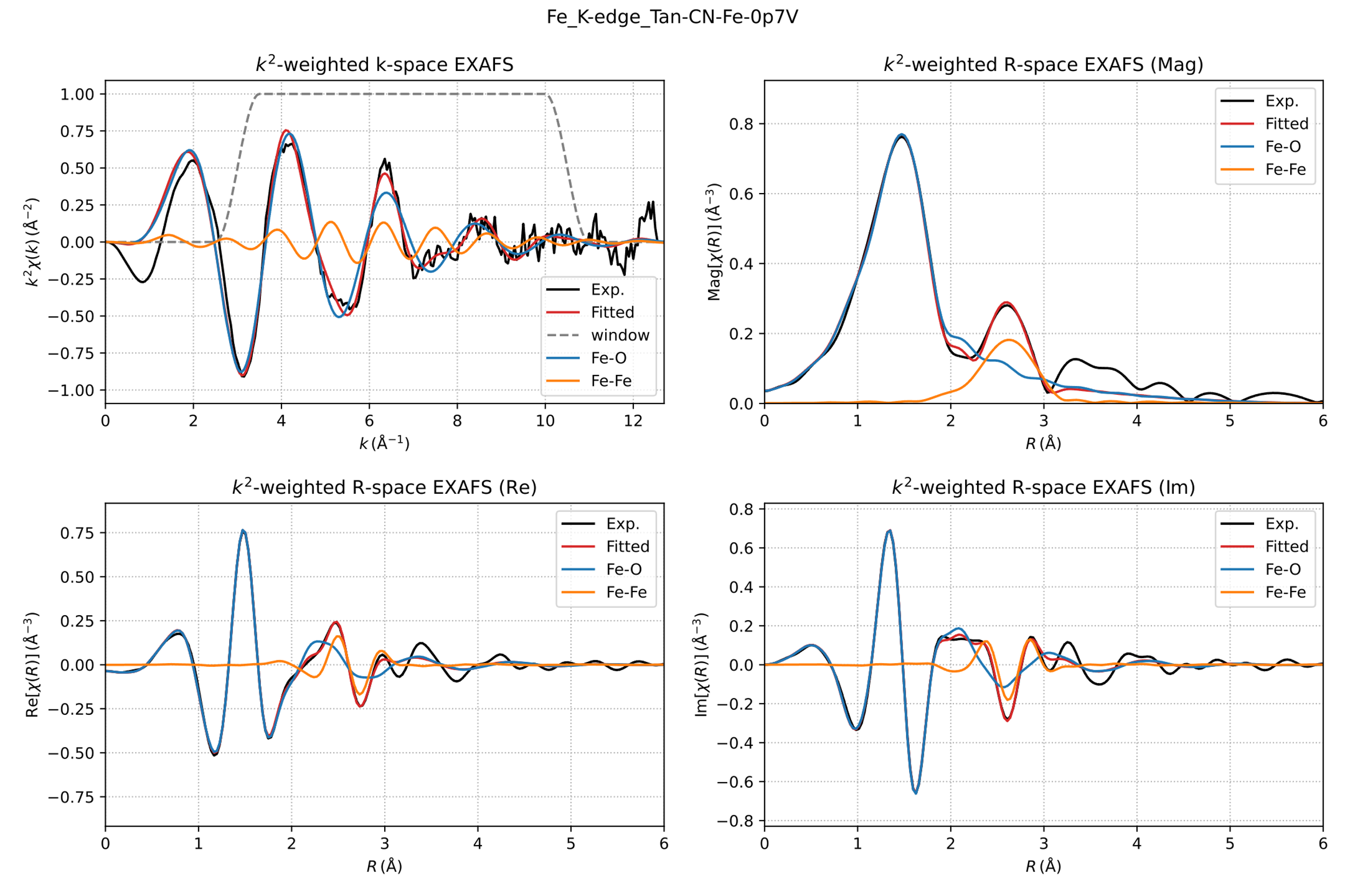


**Figure S48.** Fitting results of Fe K-edge *k*^2^-weighted k-space and R-space FT-EXAFS spectra of Tan-CN-Fe at 1.6 V vs. RHE in (a) k-space, (b) R-space magnitude, (c) R-space real part and (d) R-space imaginary part. The R-space spectra are plotted without phase correction.


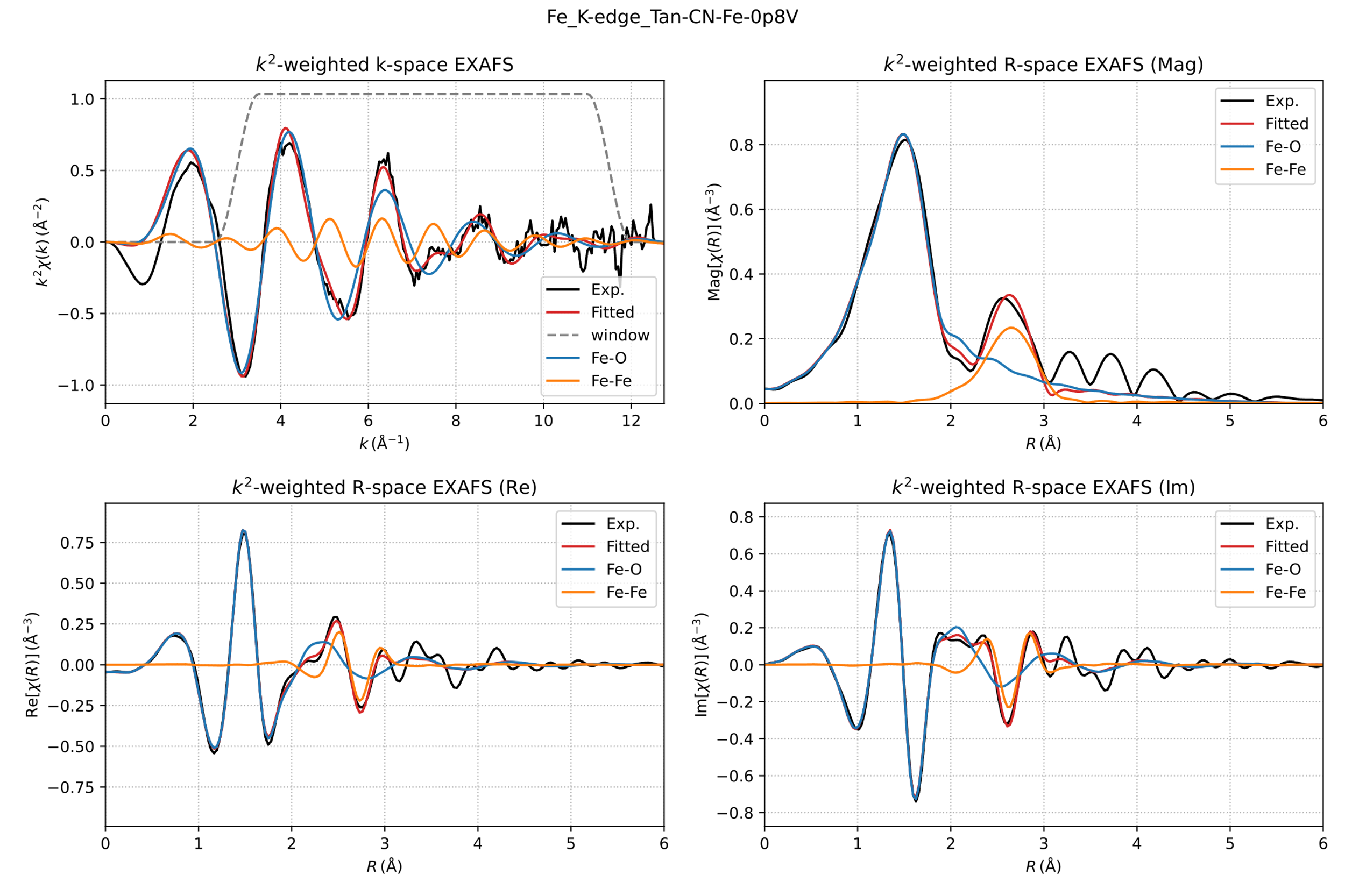


**Figure S49.** Fitting results of Fe K-edge *k*^2^-weighted k-space and R-space FT-EXAFS spectra of Tan-CN-Fe at 1.7 V vs. RHE in (a) k-space, (b) R-space magnitude, (c) R-space real part and (d) R-space imaginary part. The R-space spectra are plotted without phase correction.


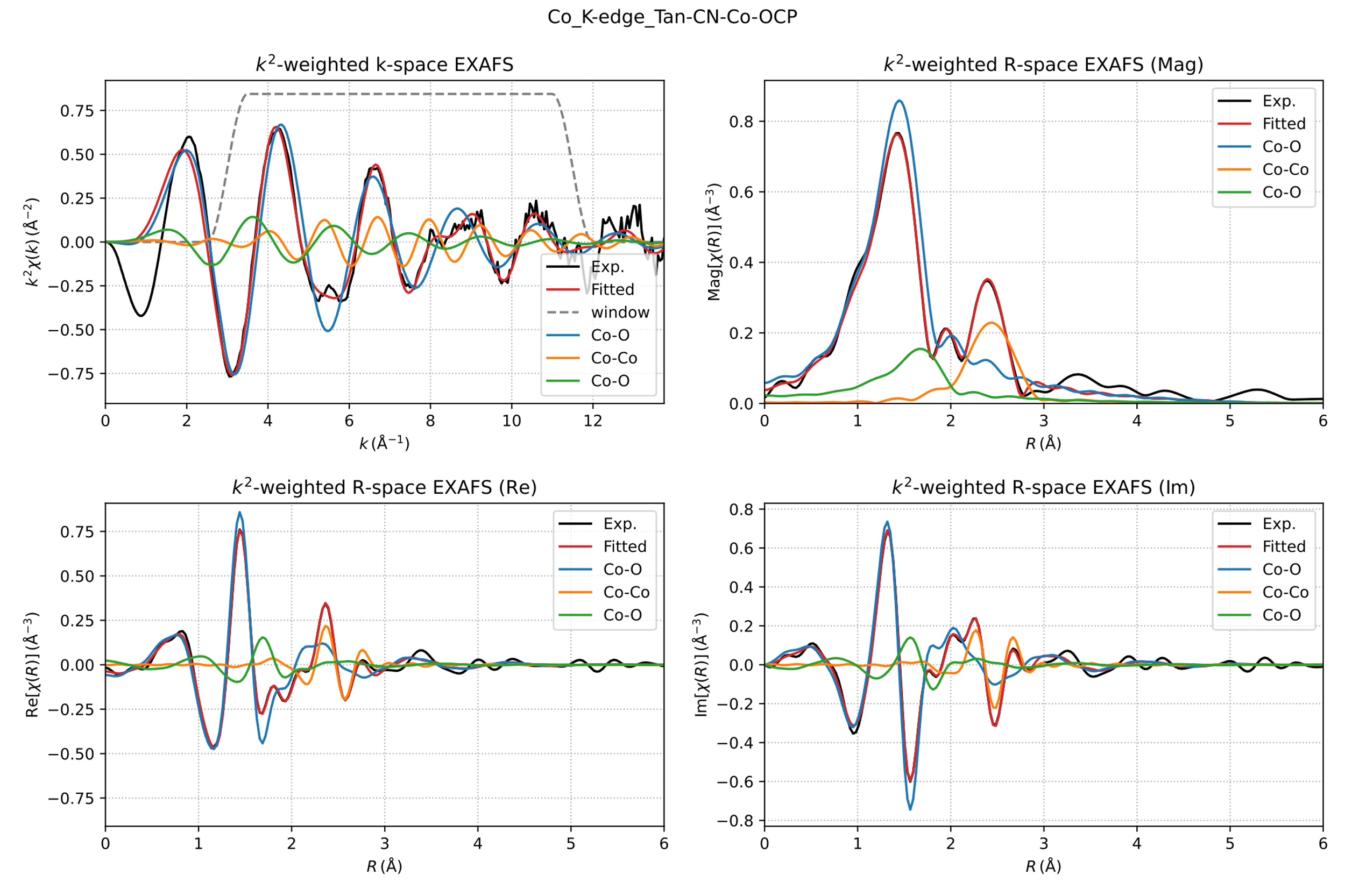


**Figure S50.** Fitting results of Co K-edge *k*^2^-weighted k-space and R-space FT-EXAFS spectra of Tan-CN-Co under OCP conditions in (a) k-space, (b) R-space magnitude, (c) R-space real part and (d) R-space imaginary part. The R-space spectra are plotted without phase correction.

**
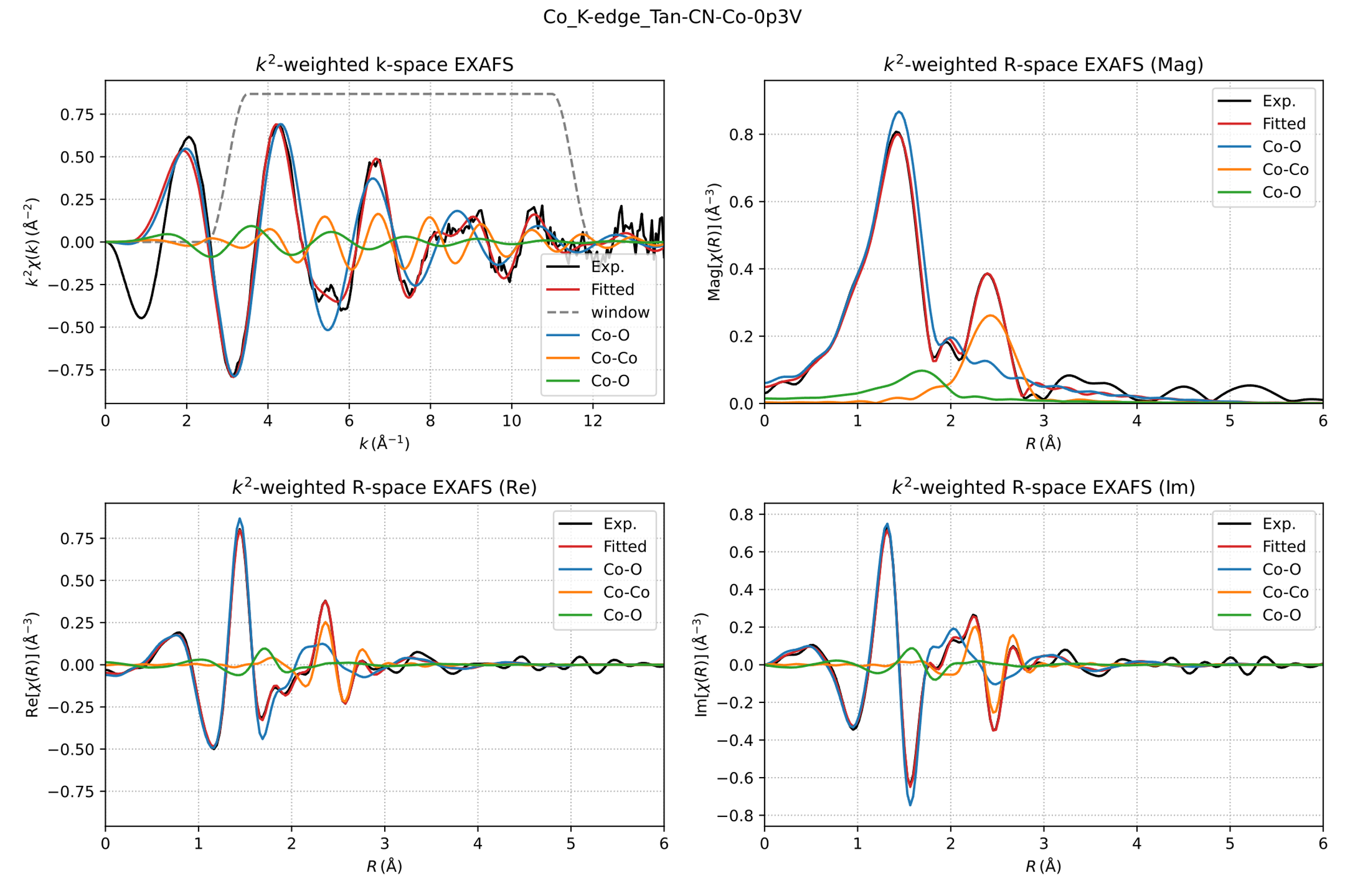
**

**Figure S51.** Fitting results of Co K-edge *k*^2^-weighted k-space and R-space FT-EXAFS spectra of Tan-CN-Co at 1.2 V vs. RHE in (a) k-space, (b) R-space magnitude, (c) R-space real part and (d) R-space imaginary part. The R-space spectra are plotted without phase correction.


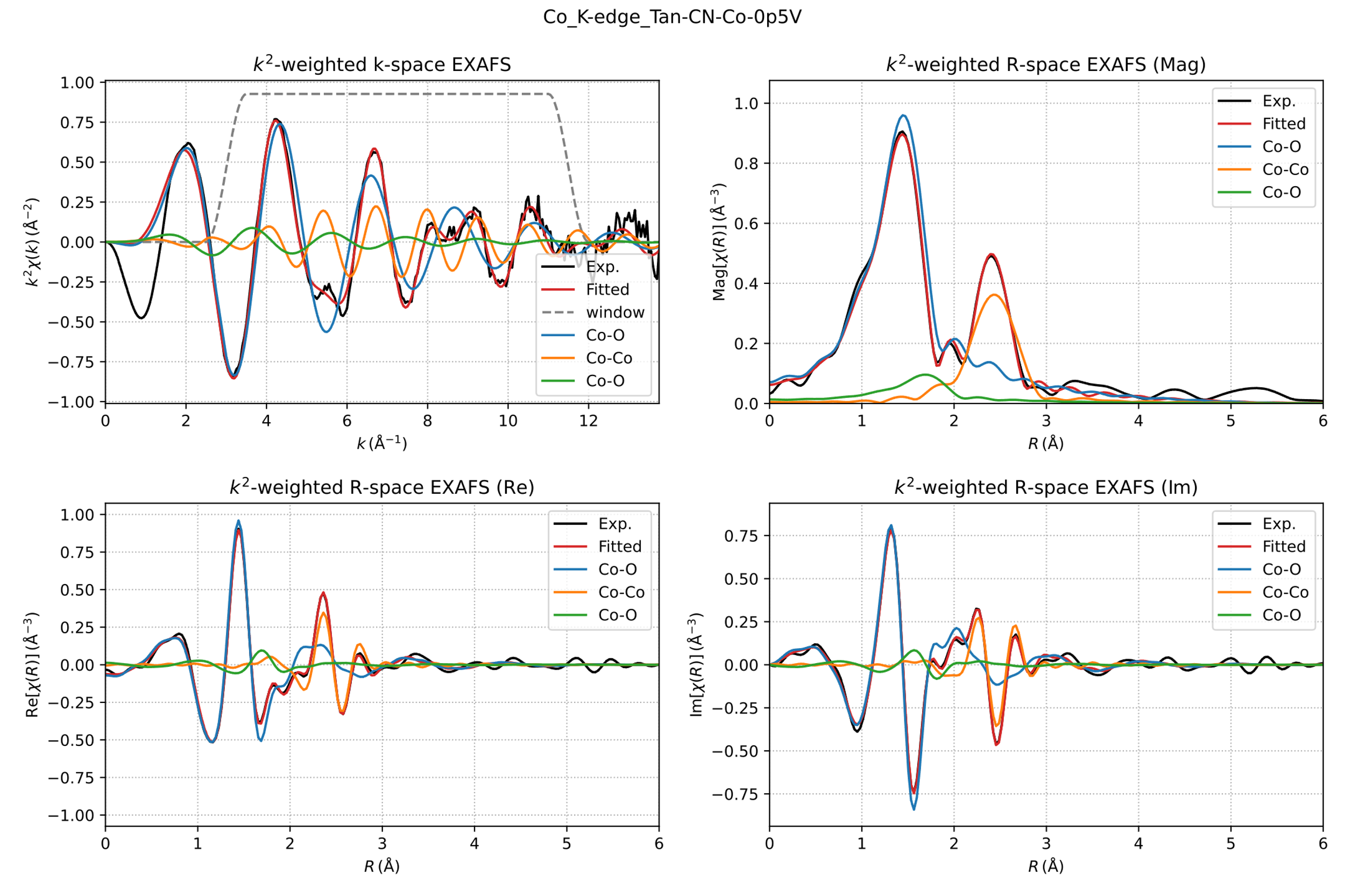


**Figure S52.** Fitting results of Co K-edge *k*^2^-weighted k-space and R-space FT-EXAFS spectra of Tan-CN-Co at 1.4 V vs. RHE in (a) k-space, (b) R-space magnitude, (c) R-space real part and (d) R-space imaginary part. The R-space spectra are plotted without phase correction.


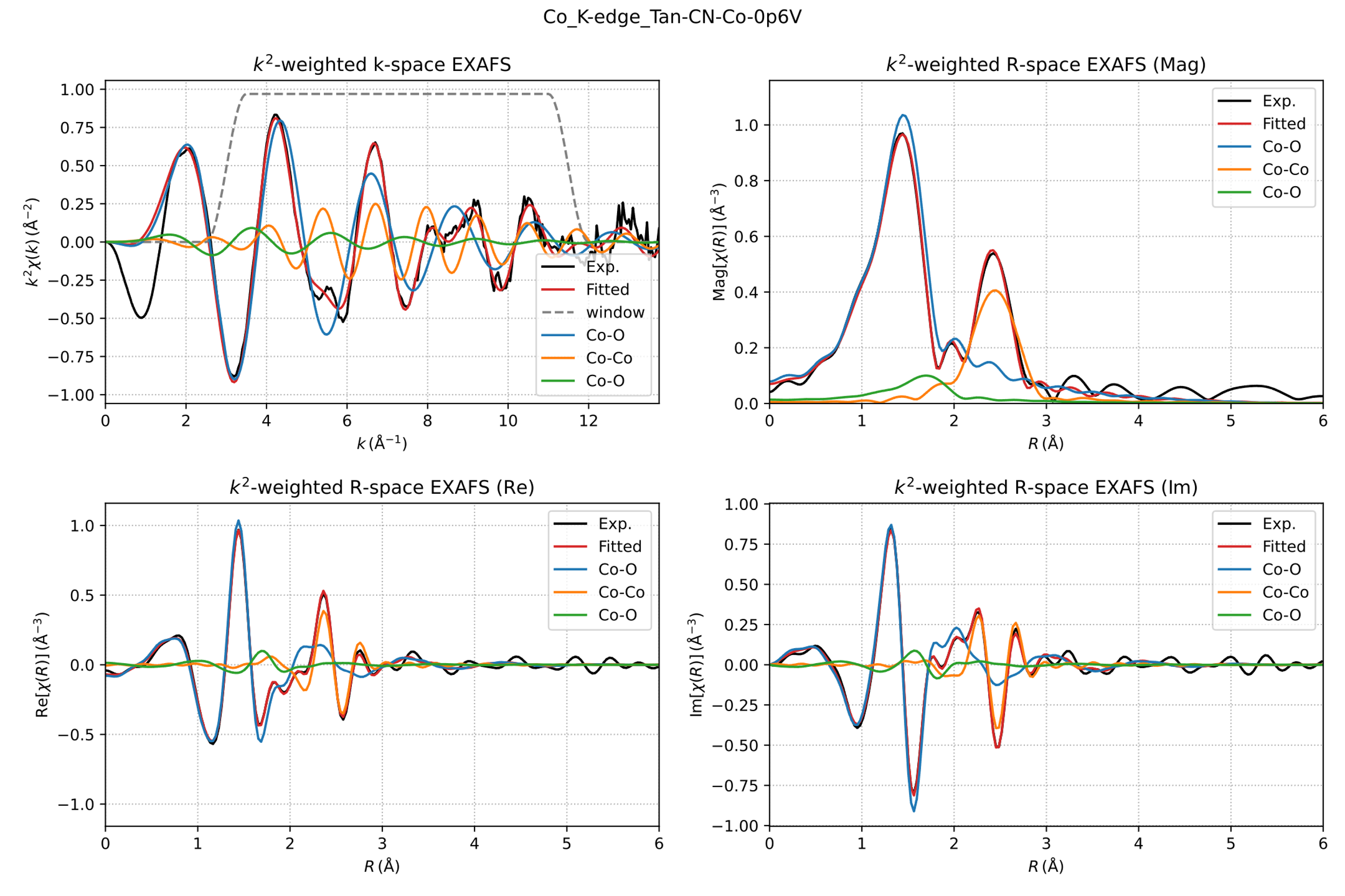


**Figure S53.** Fitting results of Co K-edge *k*^2^-weighted k-space and R-space FT-EXAFS spectra of Tan-CN-Co at 1.5 V vs. RHE in (a) k-space, (b) R-space magnitude, (c) R-space real part and (d) R-space imaginary part. The R-space spectra are plotted without phase correction.


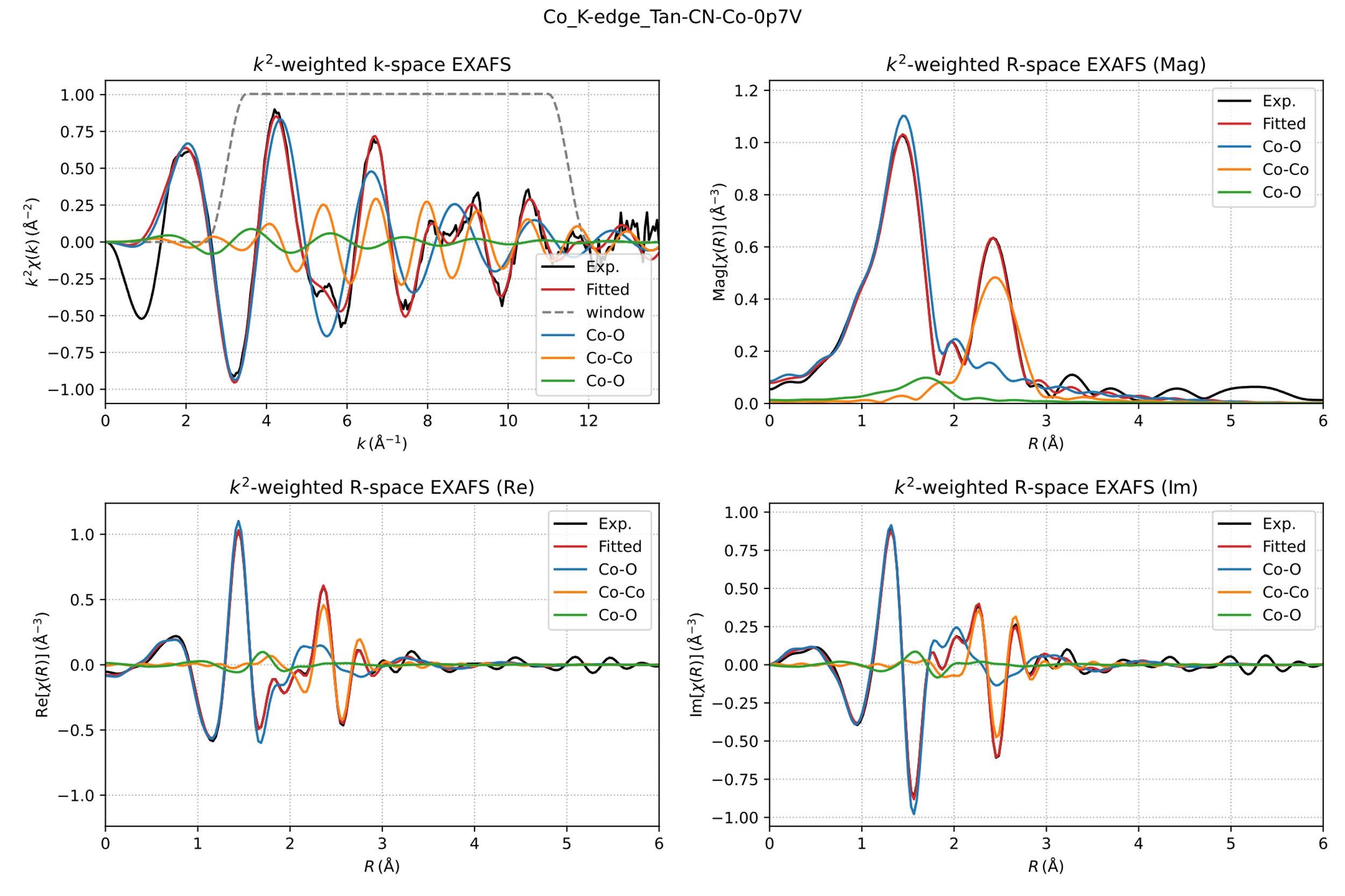


**Figure S54.** Fitting results of Co K-edge *k*^2^-weighted k-space and R-space FT-EXAFS spectra of Tan-CN-Co at 1.6 V vs. RHE in (a) k-space, (b) R-space magnitude, (c) R-space real part and (d) R-space imaginary part. The R-space spectra are plotted without phase correction.


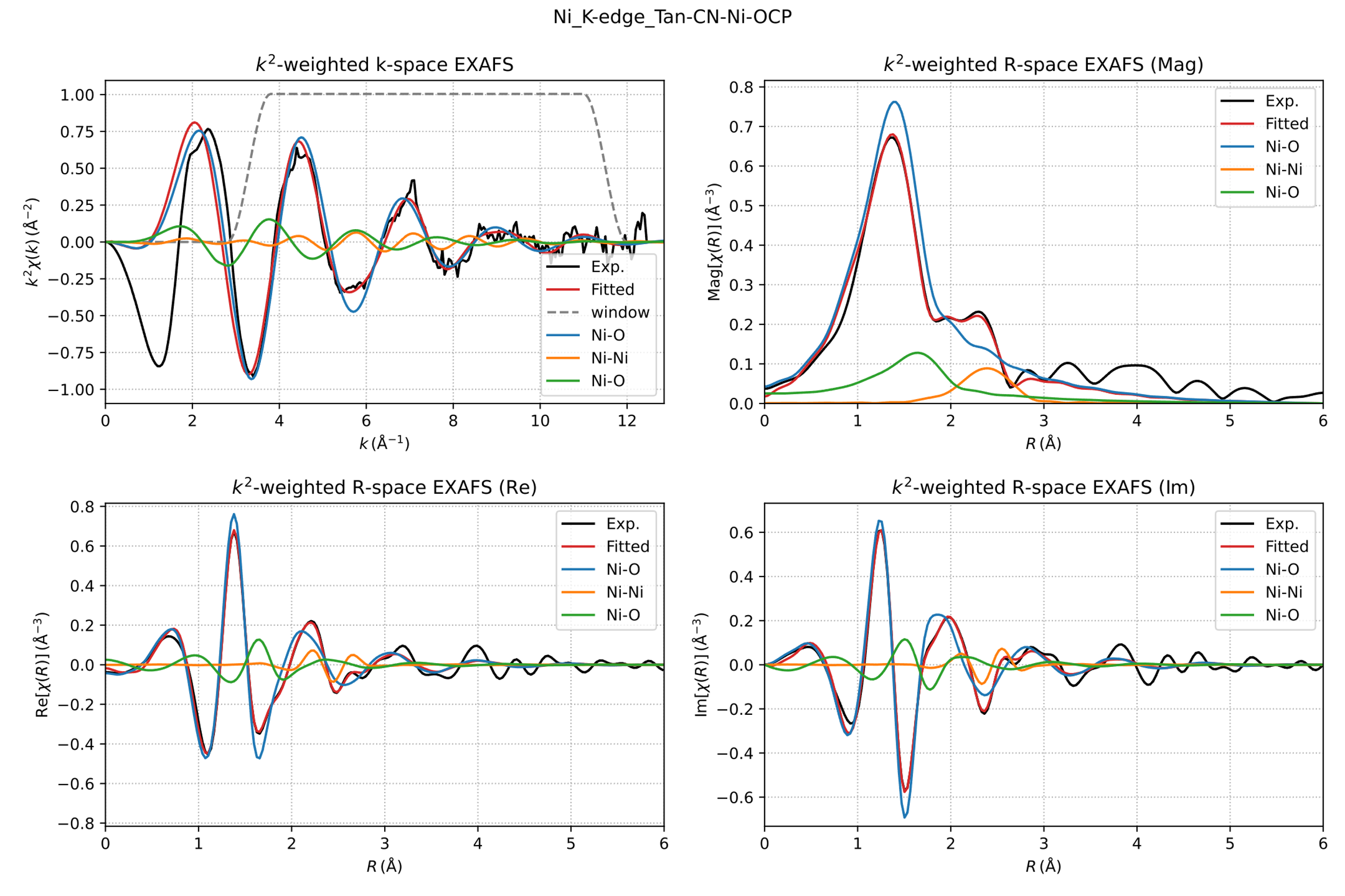


**Figure S55.** Fitting results of Ni K-edge *k*^2^-weighted k-space and R-space FT-EXAFS spectra of Tan-CN-Ni under OCP conditions in (a) k-space, (b) R-space magnitude, (c) R-space real part and (d) R-space imaginary part. The R-space spectra are plotted without phase correction.


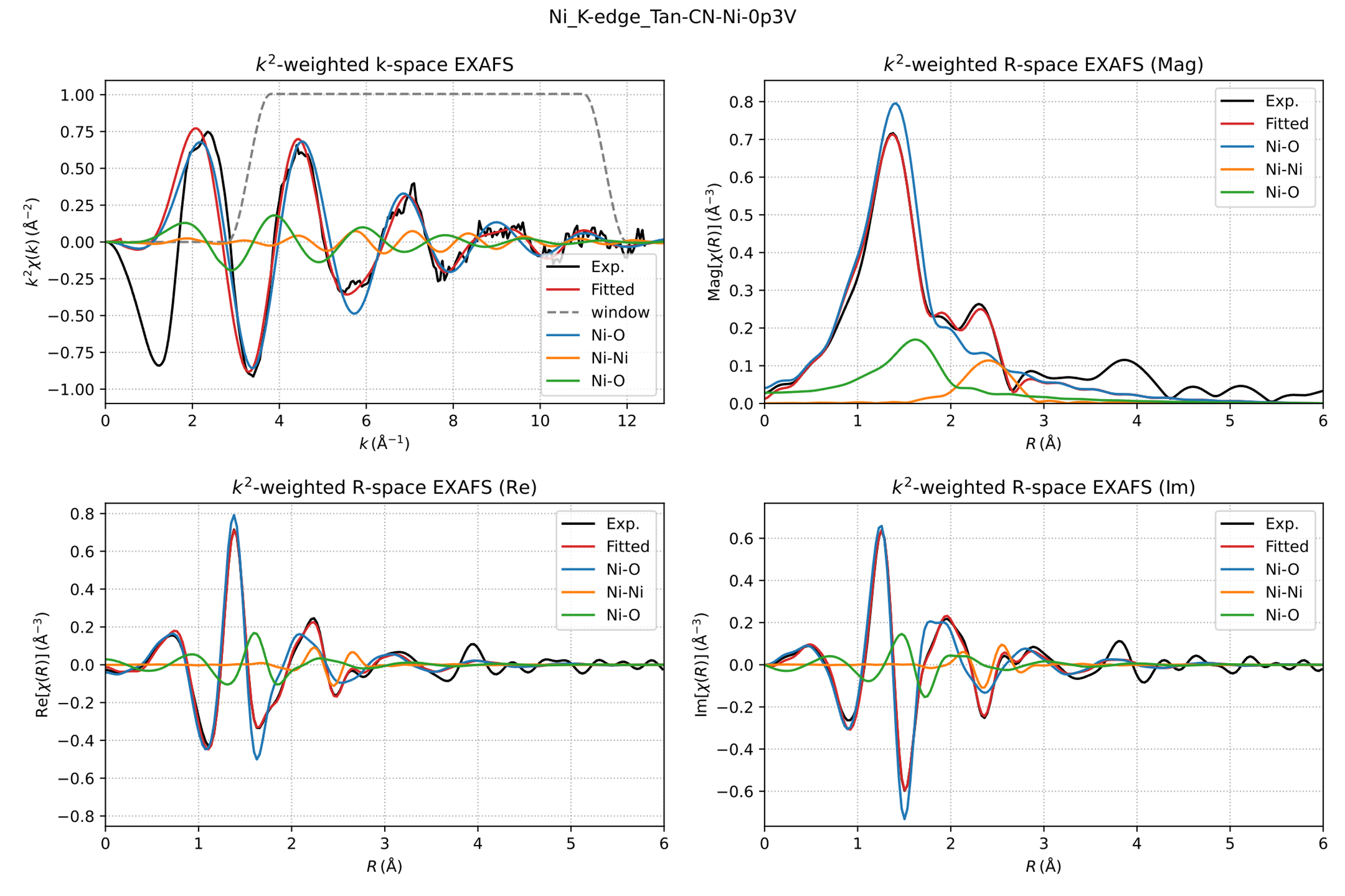


**Figure S56.** Fitting results of Ni K-edge *k*^2^-weighted k-space and R-space FT-EXAFS spectra of Tan-CN-Ni at 1.2 V vs. RHE in (a) k-space, (b) R-space magnitude, (c) R-space real part and (d) R-space imaginary part. The R-space spectra are plotted without phase correction.


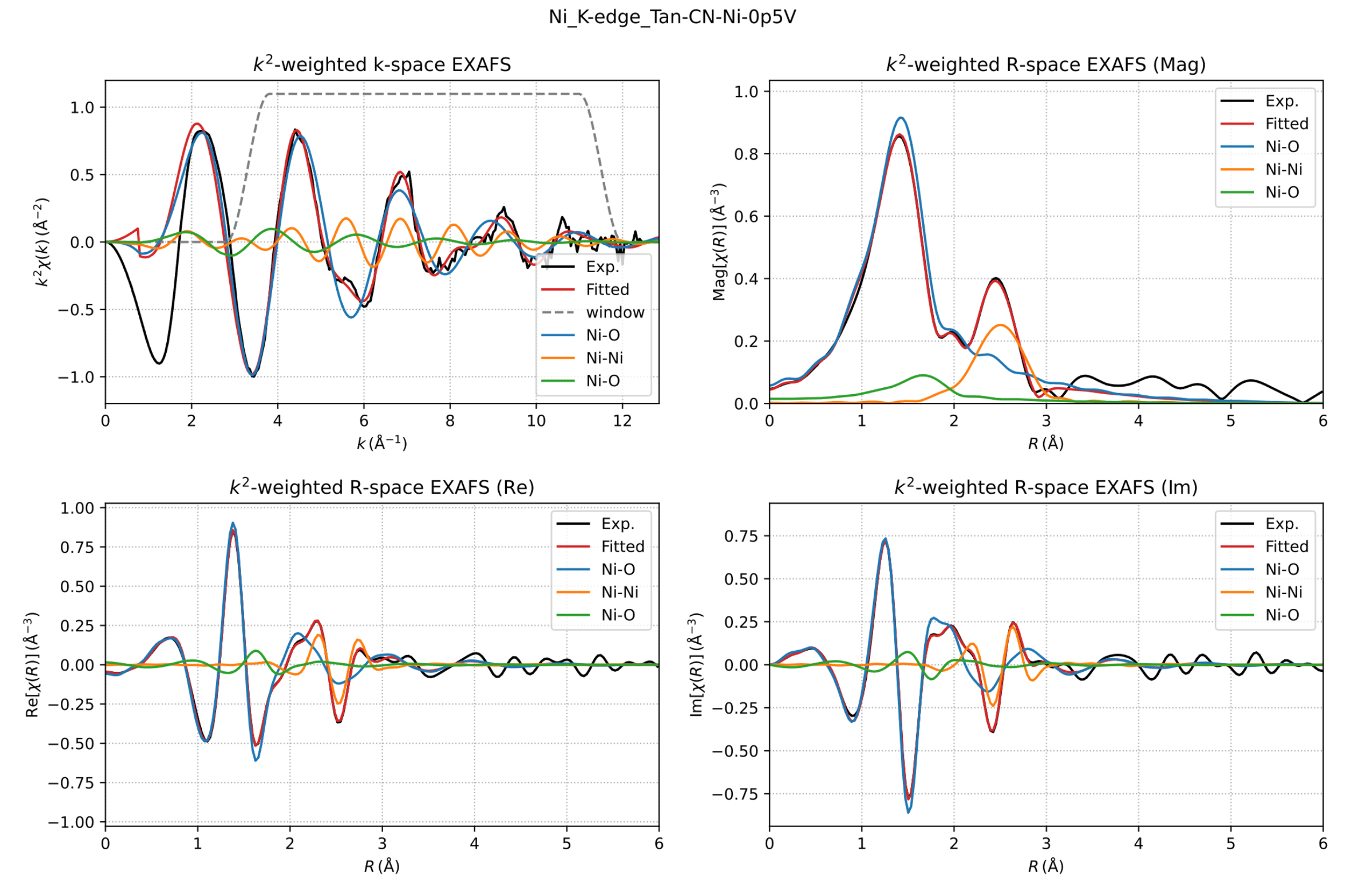


**Figure S57.** Fitting results of Ni K-edge *k*^2^-weighted k-space and R-space FT-EXAFS spectra of Tan-CN-Ni at 1.4 V vs. RHE in (a) k-space, (b) R-space magnitude, (c) R-space real part and (d) R-space imaginary part. The R-space spectra are plotted without phase correction.

**
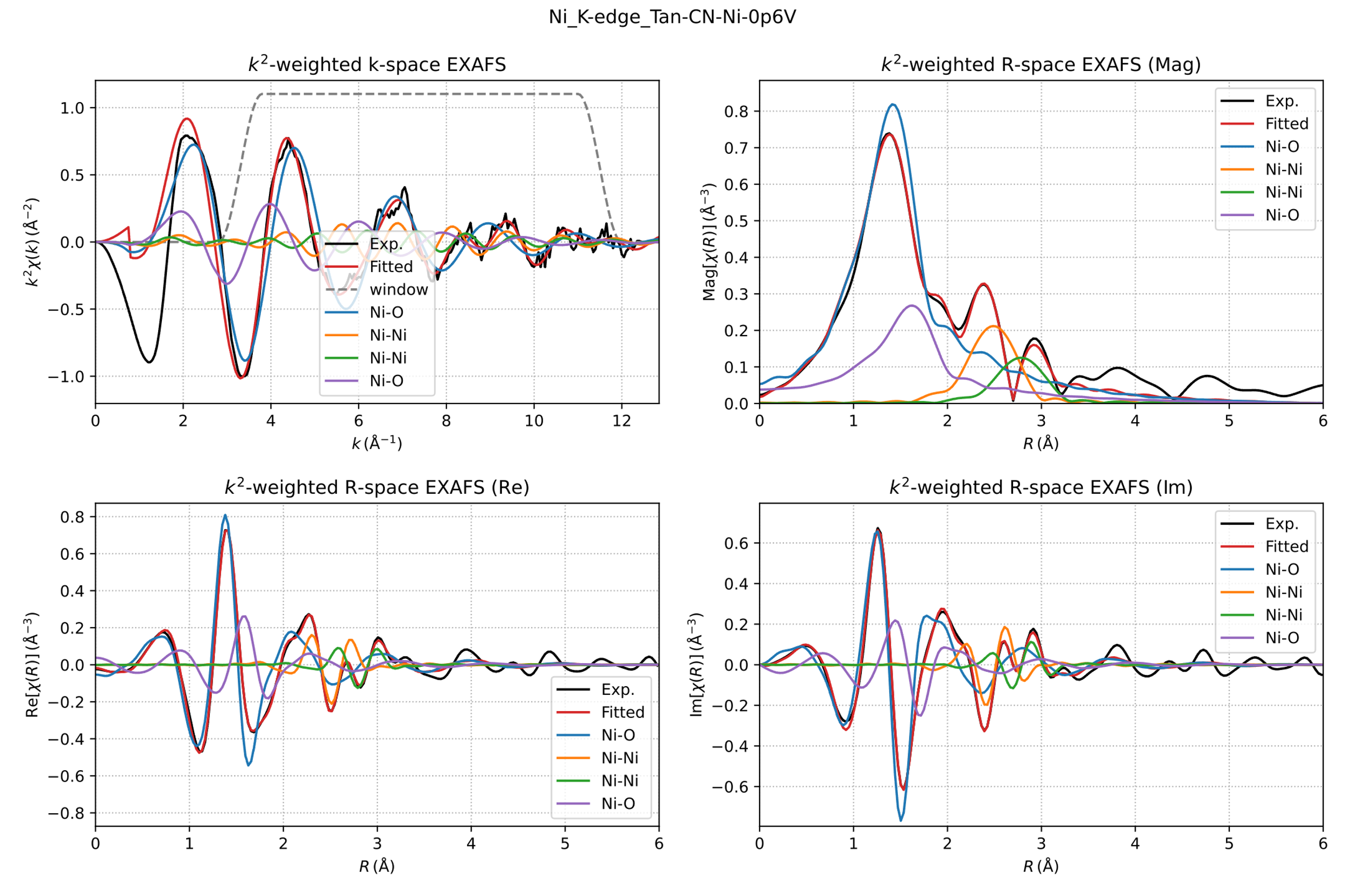
**

**Figure S58.** Fitting results of Ni K-edge *k*^2^-weighted k-space and R-space FT-EXAFS spectra of Tan-CN-Ni at 1.5 V vs. RHE in (a) k-space, (b) R-space magnitude, (c) R-space real part and (d) R-space imaginary part. The R-space spectra are plotted without phase correction.

**
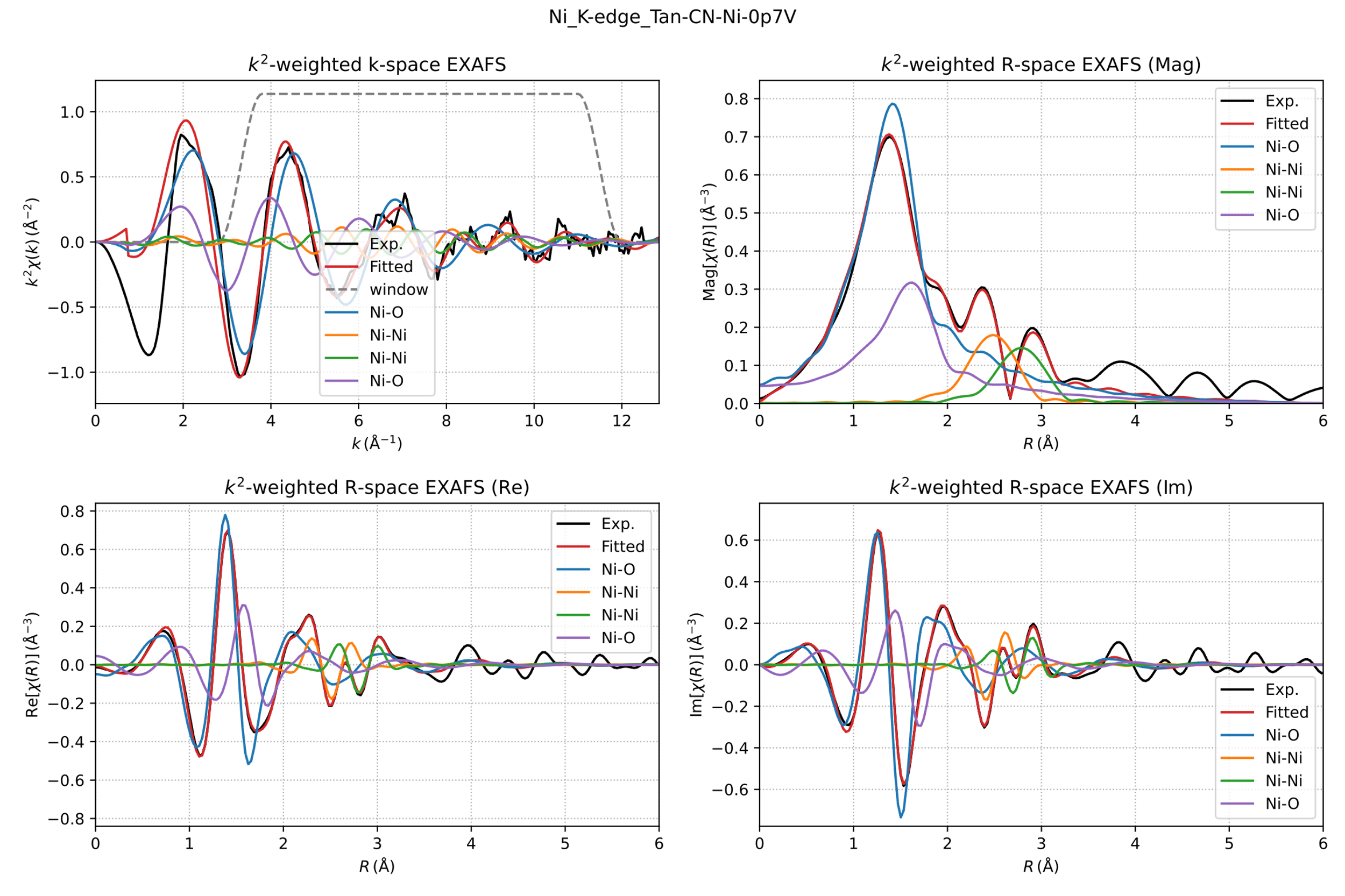
**

**Figure S59.** Fitting results of Ni K-edge *k*^2^-weighted k-space and R-space FT-EXAFS spectra of Tan-CN-Ni at 1.6 V vs. RHE in (a) k-space, (b) R-space magnitude, (c) R-space real part and (d) R-space imaginary part. The R-space spectra are plotted without phase correction.


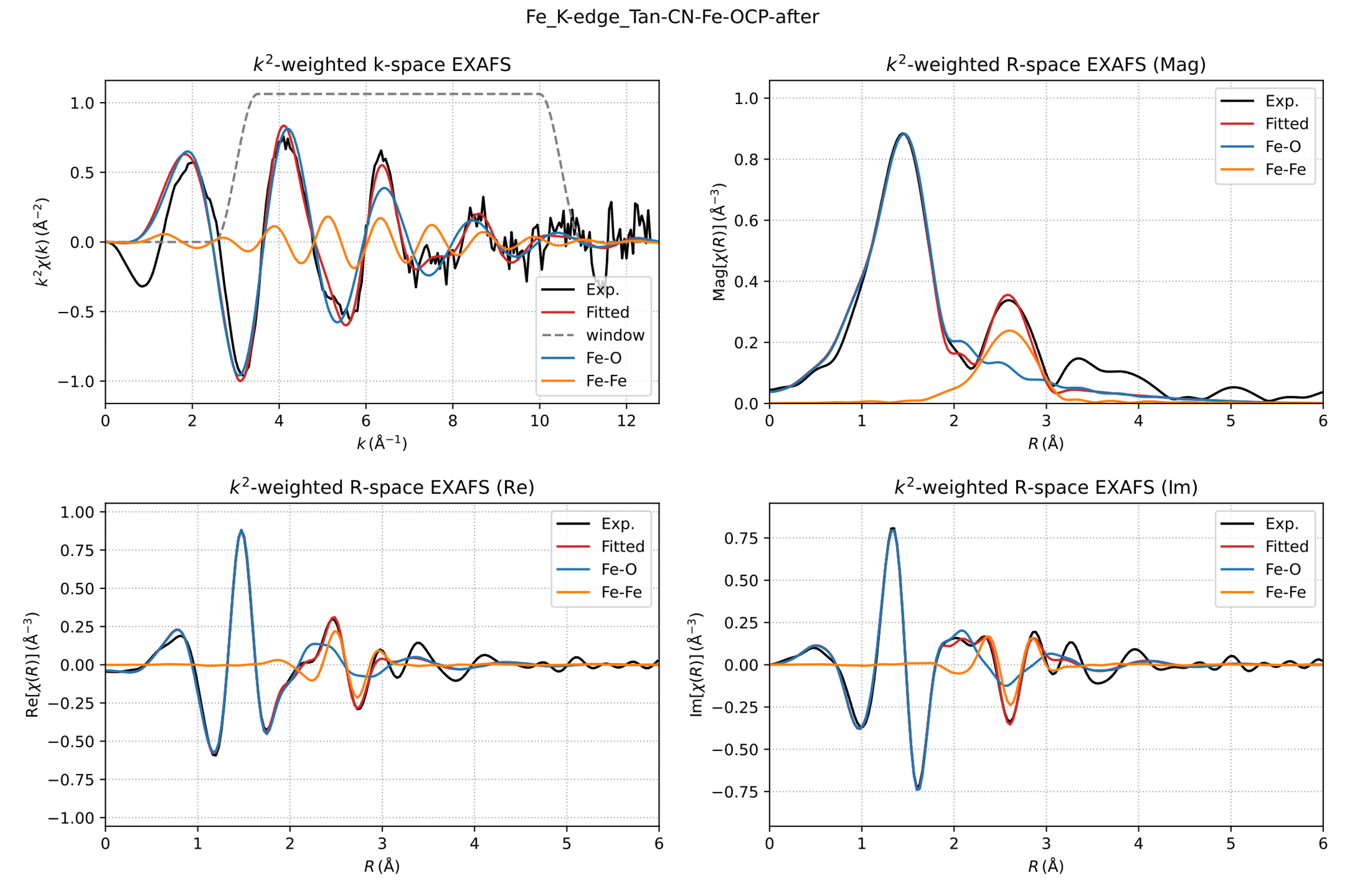


**Figure S60.** Fitting results of Fe K-edge *k*^2^-weighted k-space and R-space FT-EXAFS spectra of Tan-CN-CoFe under OCP conditions in (a) k-space, (b) R-space magnitude, (c) R-space real part and (d) R-space imaginary part. The R-space spectra are plotted without phase correction.

**
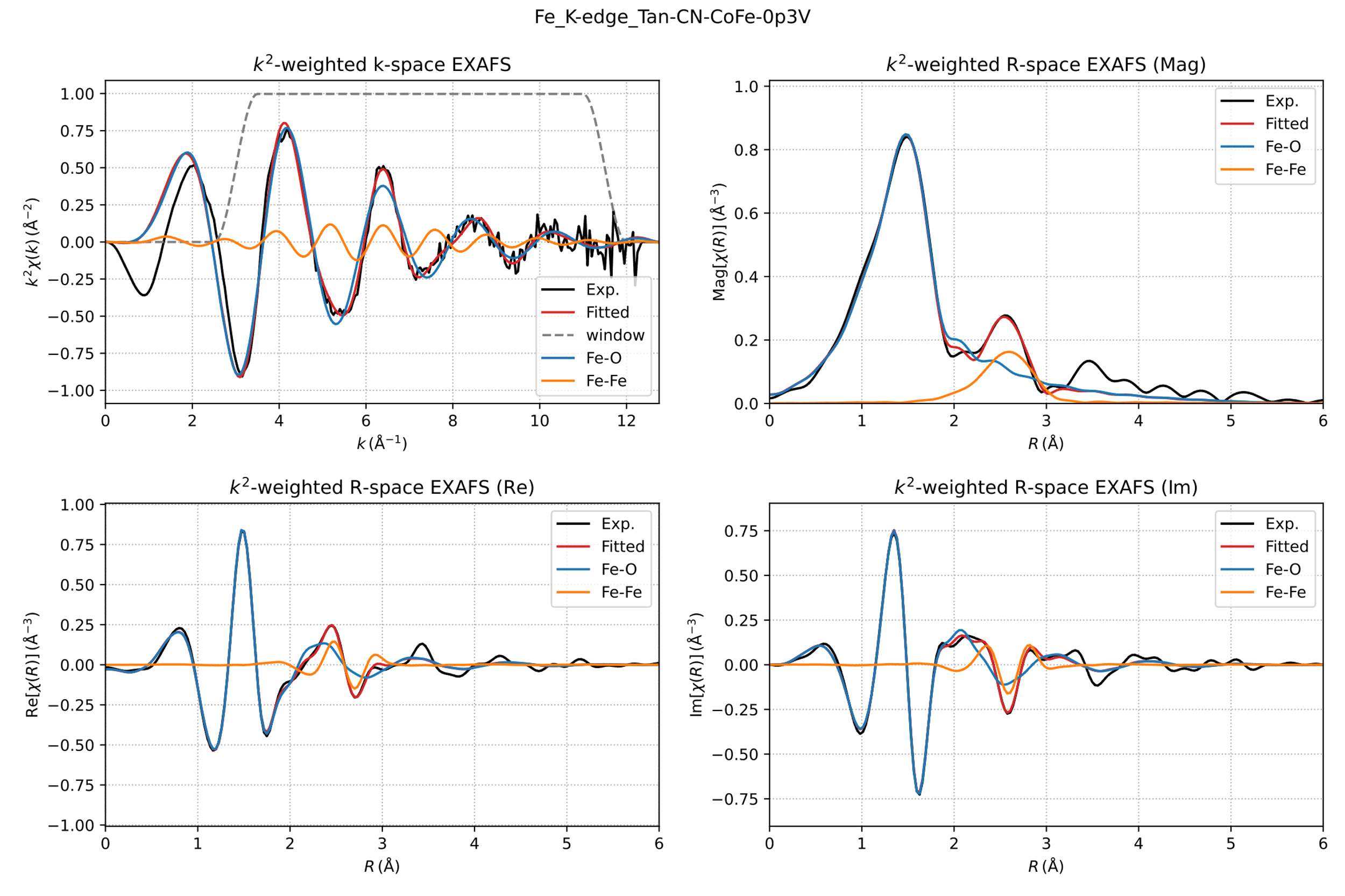
**

**Figure S61.** Fitting results of Fe K-edge *k*^2^-weighted k-space and R-space FT-EXAFS spectra of Tan-CN-CoFe at 1.2 V vs. RHE in (a) k-space, (b) R-space magnitude, (c) R-space real part and (d) R-space imaginary part. The R-space spectra are plotted without phase correction.

**
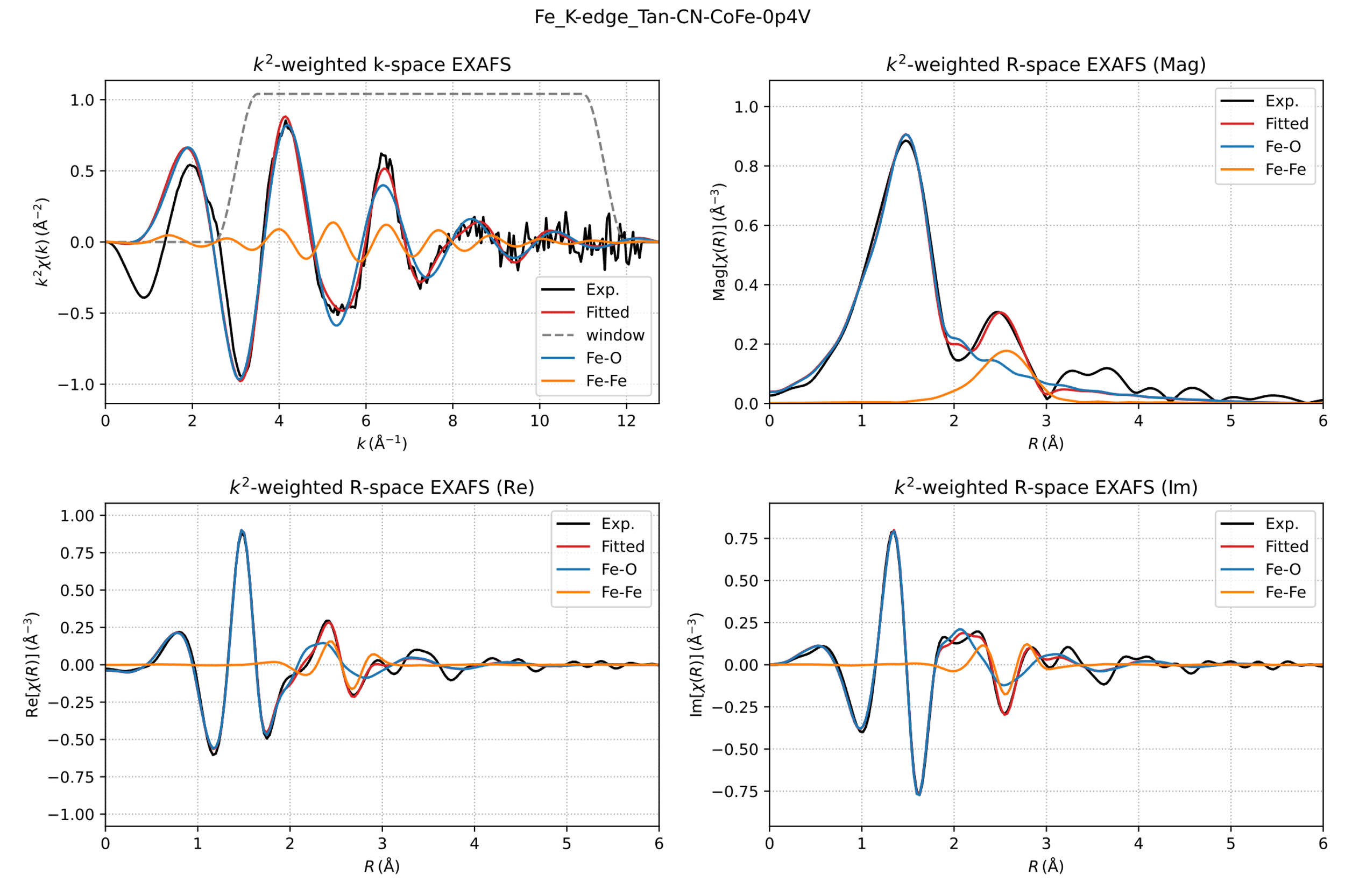
**

**Figure S62.** Fitting results of Fe K-edge *k*^2^-weighted k-space and R-space FT-EXAFS spectra of Tan-CN-CoFe at 1.3 V vs. RHE in (a) k-space, (b) R-space magnitude, (c) R-space real part and (d) R-space imaginary part. The R-space spectra are plotted without phase correction.


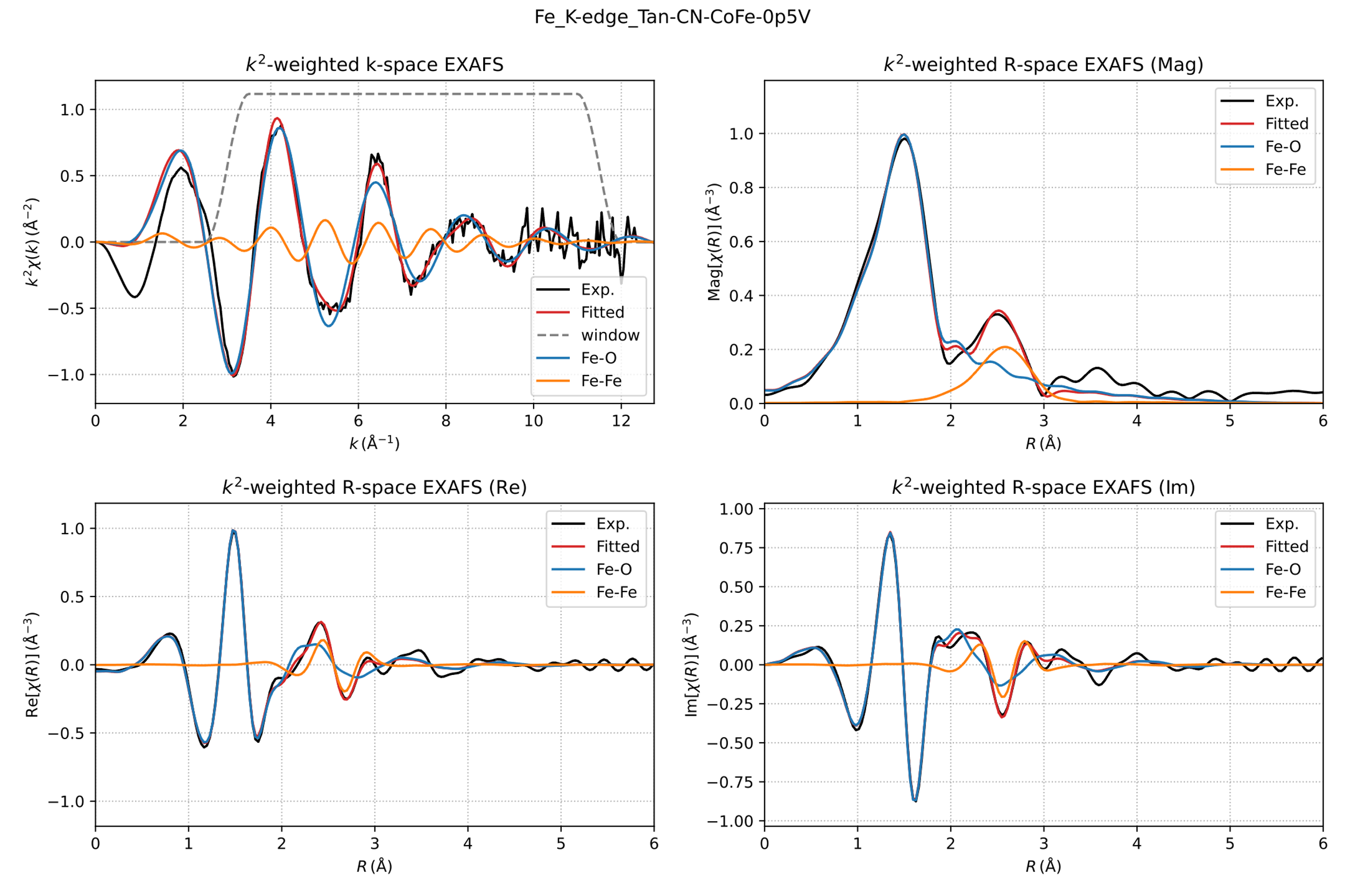


**Figure S63.** Fitting results of Fe K-edge *k*^2^-weighted k-space and R-space FT-EXAFS spectra of Tan-CN-CoFe at 1.4 V vs. RHE in (a) k-space, (b) R-space magnitude, (c) R-space real part and (d) R-space imaginary part. The R-space spectra are plotted without phase correction.

**
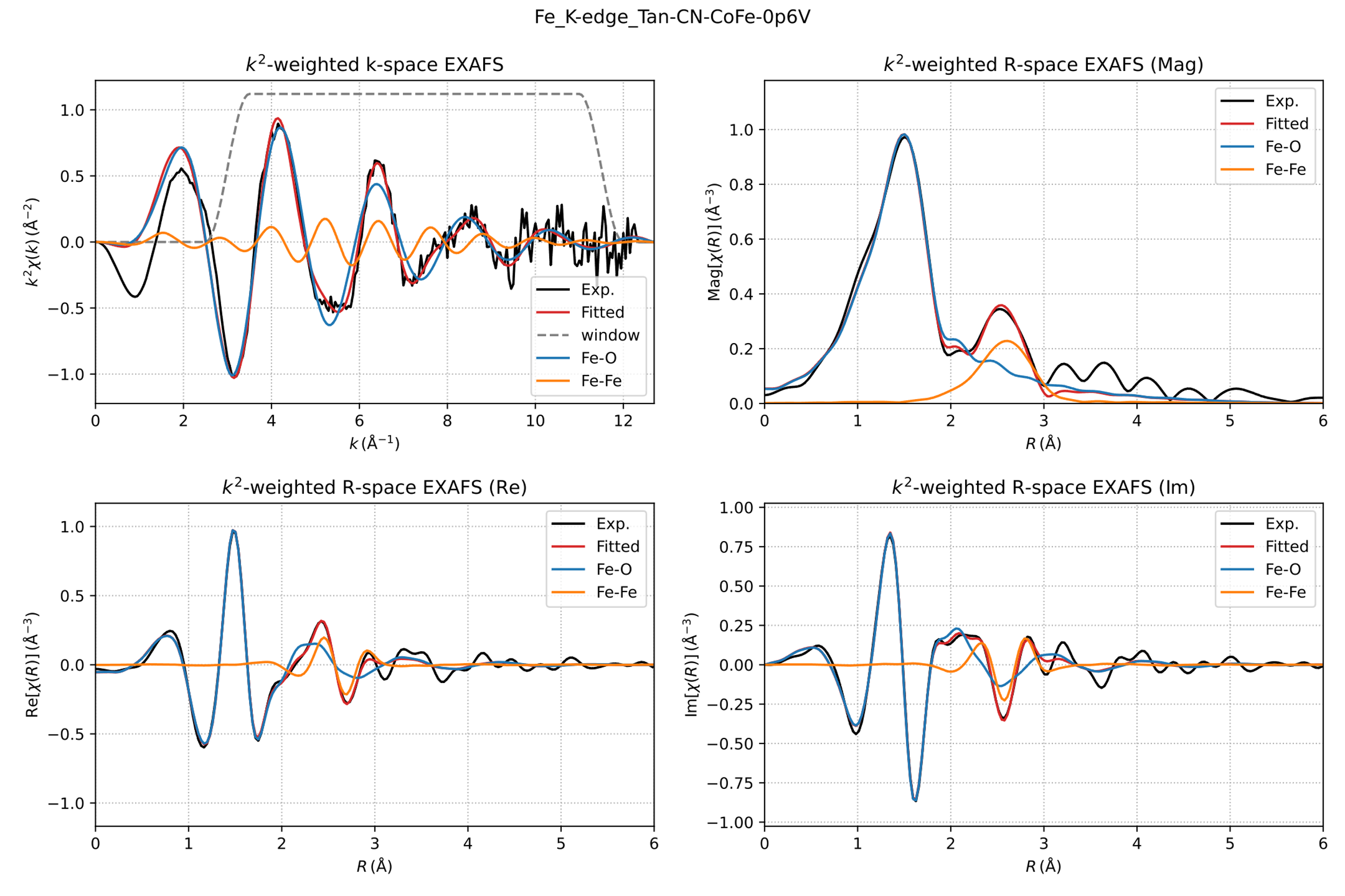
**

**Figure S64.** Fitting results of Fe K-edge *k*^2^-weighted k-space and R-space FT-EXAFS spectra of Tan-CN-CoFe at 1.5 V vs. RHE in (a) k-space, (b) R-space magnitude, (c) R-space real part and (d) R-space imaginary part. The R-space spectra are plotted without phase correction.

**
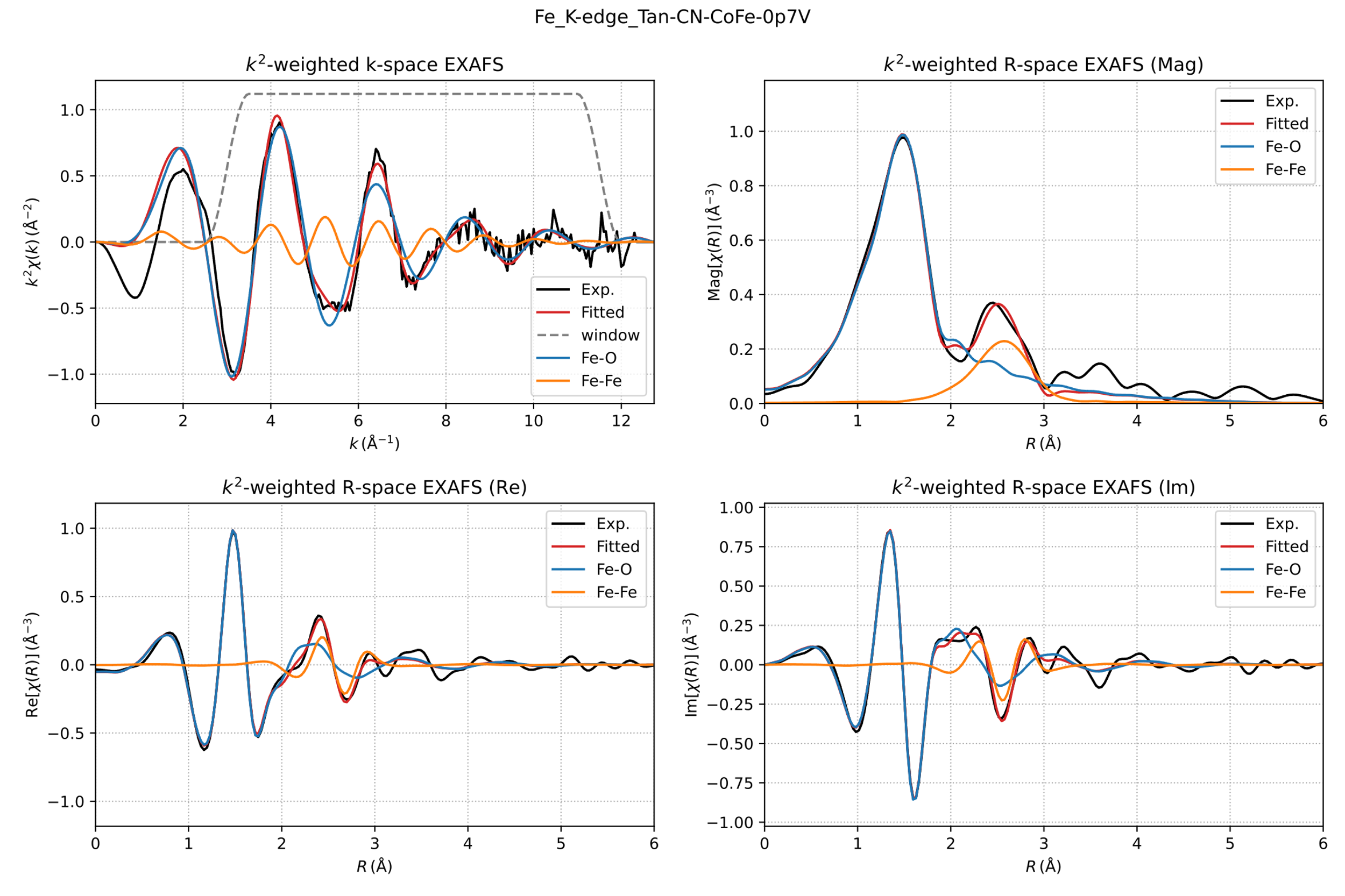
**

**Figure S65.** Fitting results of Fe K-edge *k*^2^-weighted k-space and R-space FT-EXAFS spectra of Tan-CN-CoFe at 1.6 V vs. RHE in (a) k-space, (b) R-space magnitude, (c) R-space real part and (d) R-space imaginary part. The R-space spectra are plotted without phase correction.

**
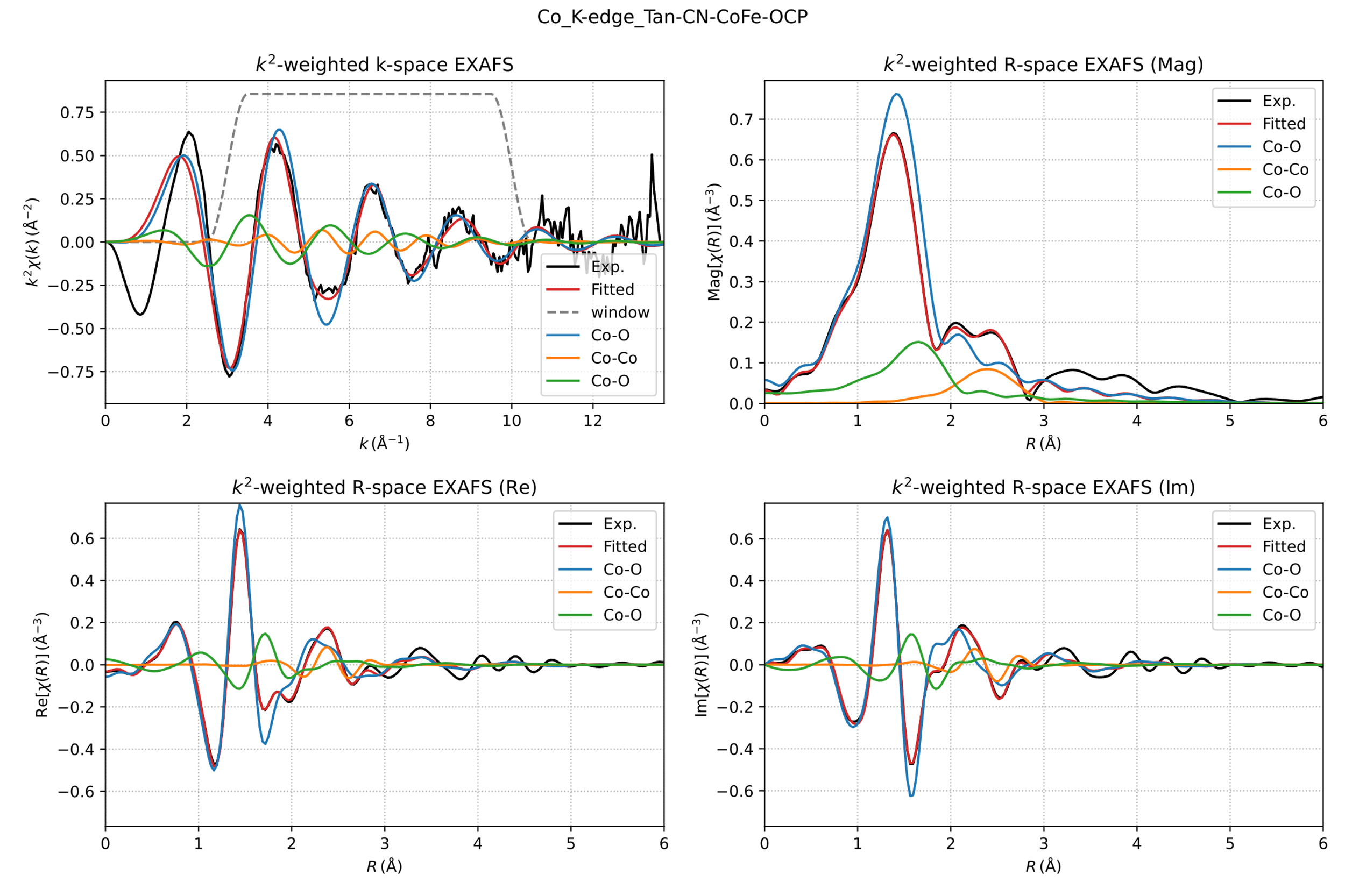
**

**Figure S66.** Fitting results of Co K-edge *k*^2^-weighted k-space and R-space FT-EXAFS spectra of Tan-CN-CoFe under OCP conditions in (a) k-space, (b) R-space magnitude, (c) R-space real part and (d) R-space imaginary part. The R-space spectra are plotted without phase correction.

**
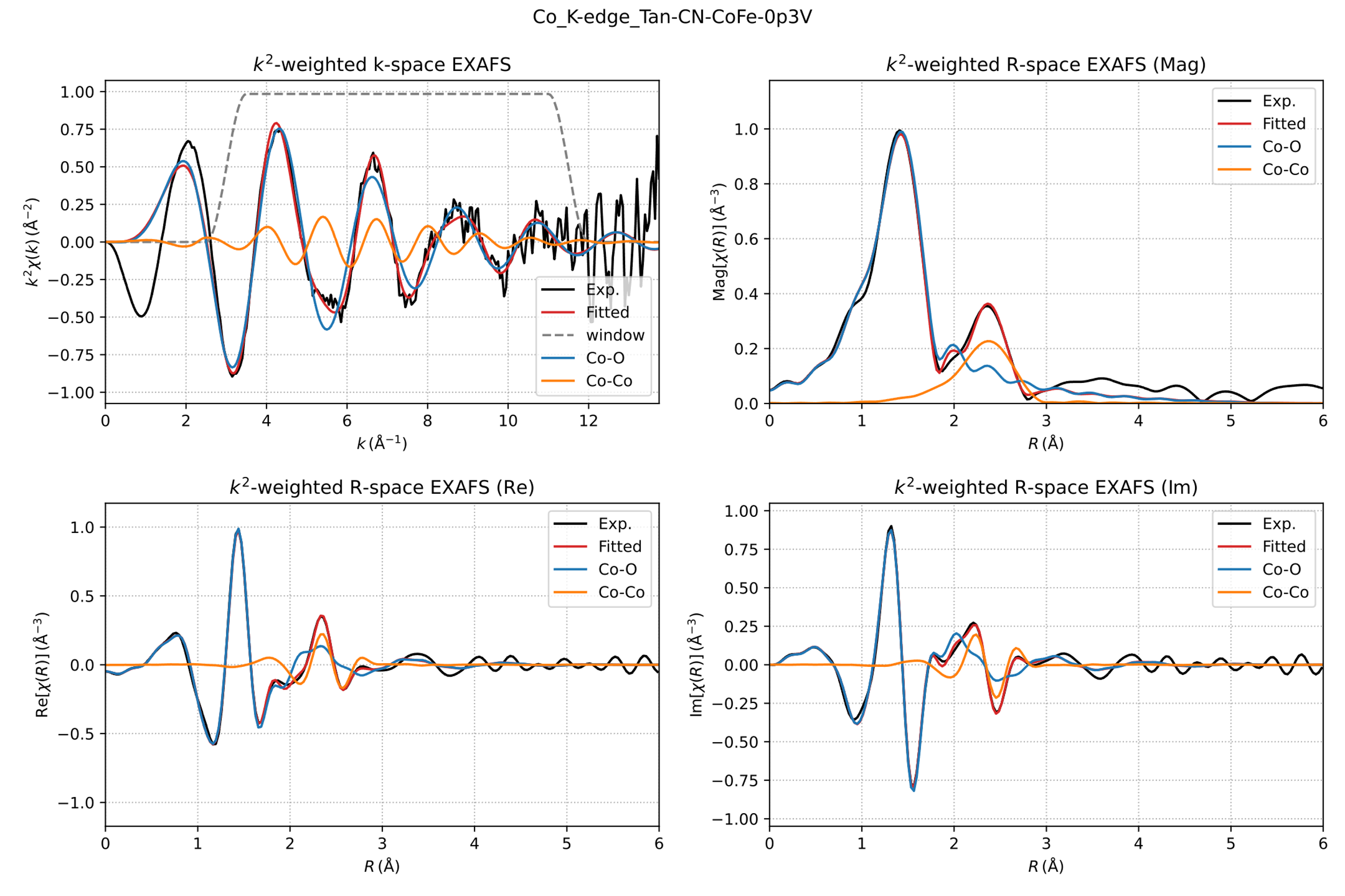
**

**Figure S67.** Fitting results of Co K-edge *k*^2^-weighted k-space and R-space FT-EXAFS spectra of Tan-CN-CoFe at 1.2 V vs. RHE in (a) k-space, (b) R-space magnitude, (c) R-space real part and (d) R-space imaginary part. The R-space spectra are plotted without phase correction.

**
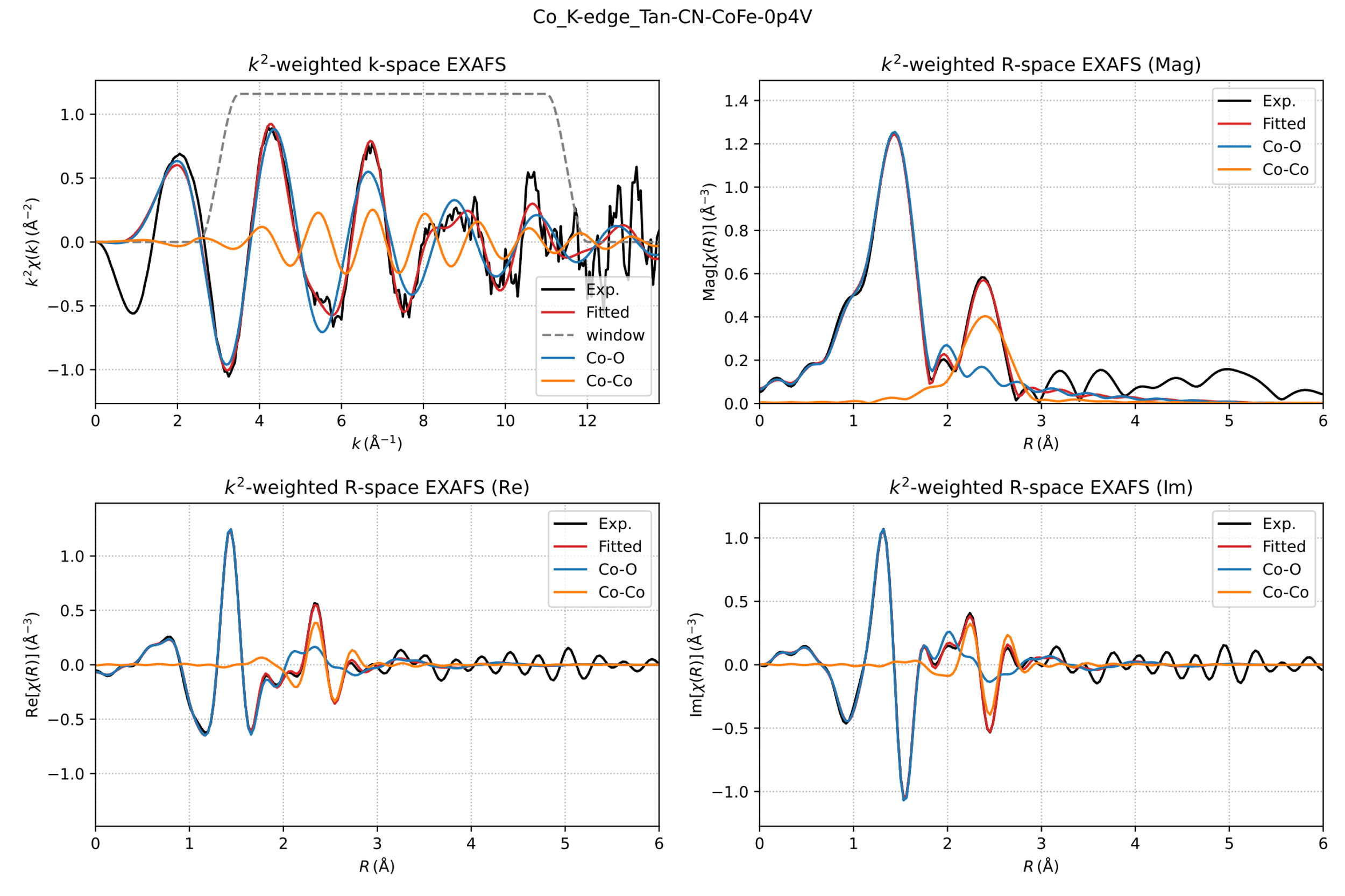
**

**Figure S68.** Fitting results of Co K-edge *k*^2^-weighted k-space and R-space FT-EXAFS spectra of Tan-CN-CoFe at 1.3 V vs. RHE in (a) k-space, (b) R-space magnitude, (c) R-space real part and (d) R-space imaginary part. The R-space spectra are plotted without phase correction.

**
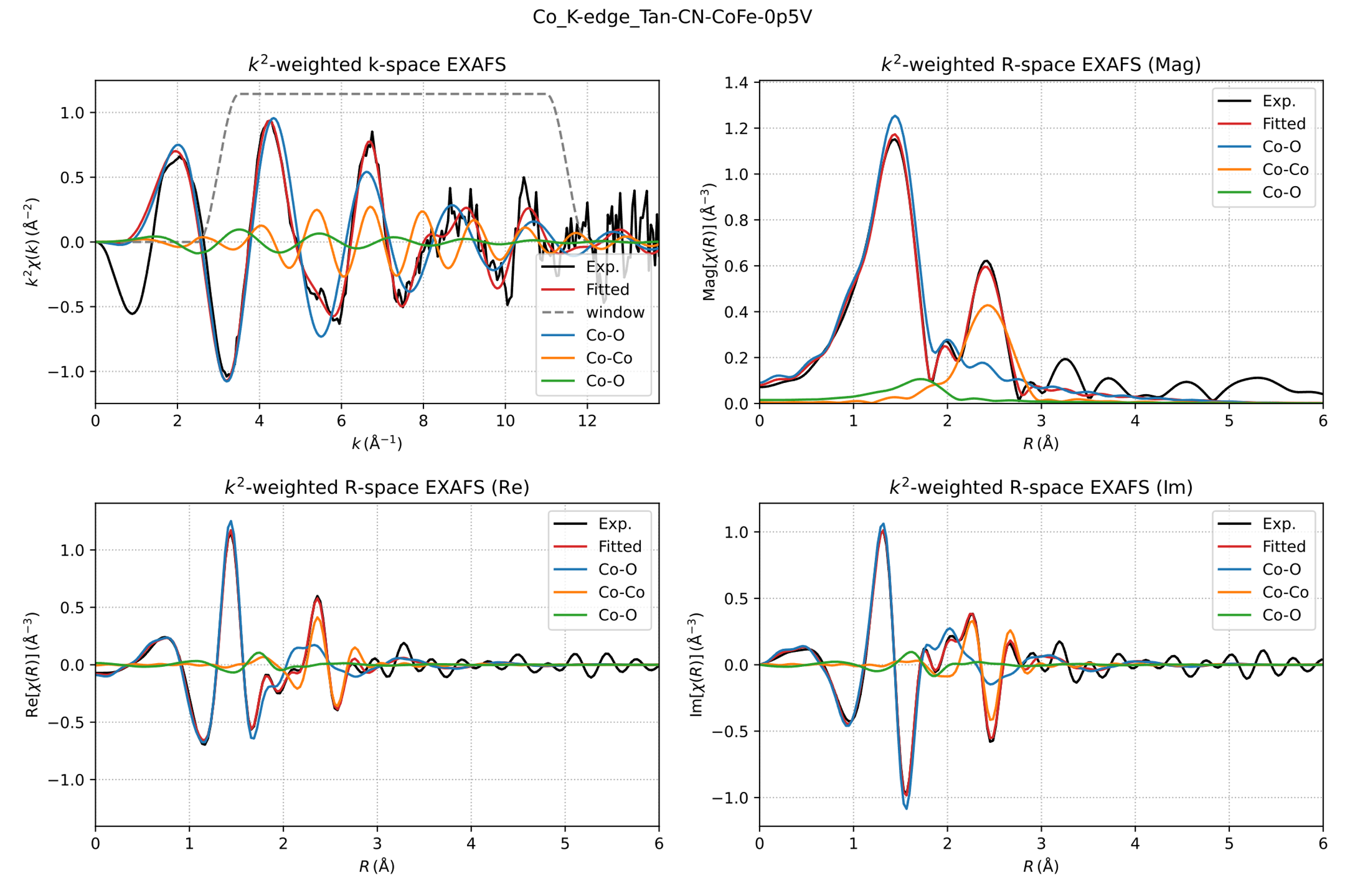
**

**Figure S69.** Fitting results of Co K-edge *k*^2^-weighted k-space and R-space FT-EXAFS spectra of Tan-CN-CoFe at 1.4 V vs. RHE in (a) k-space, (b) R-space magnitude, (c) R-space real part and (d) R-space imaginary part. The R-space spectra are plotted without phase correction.

**
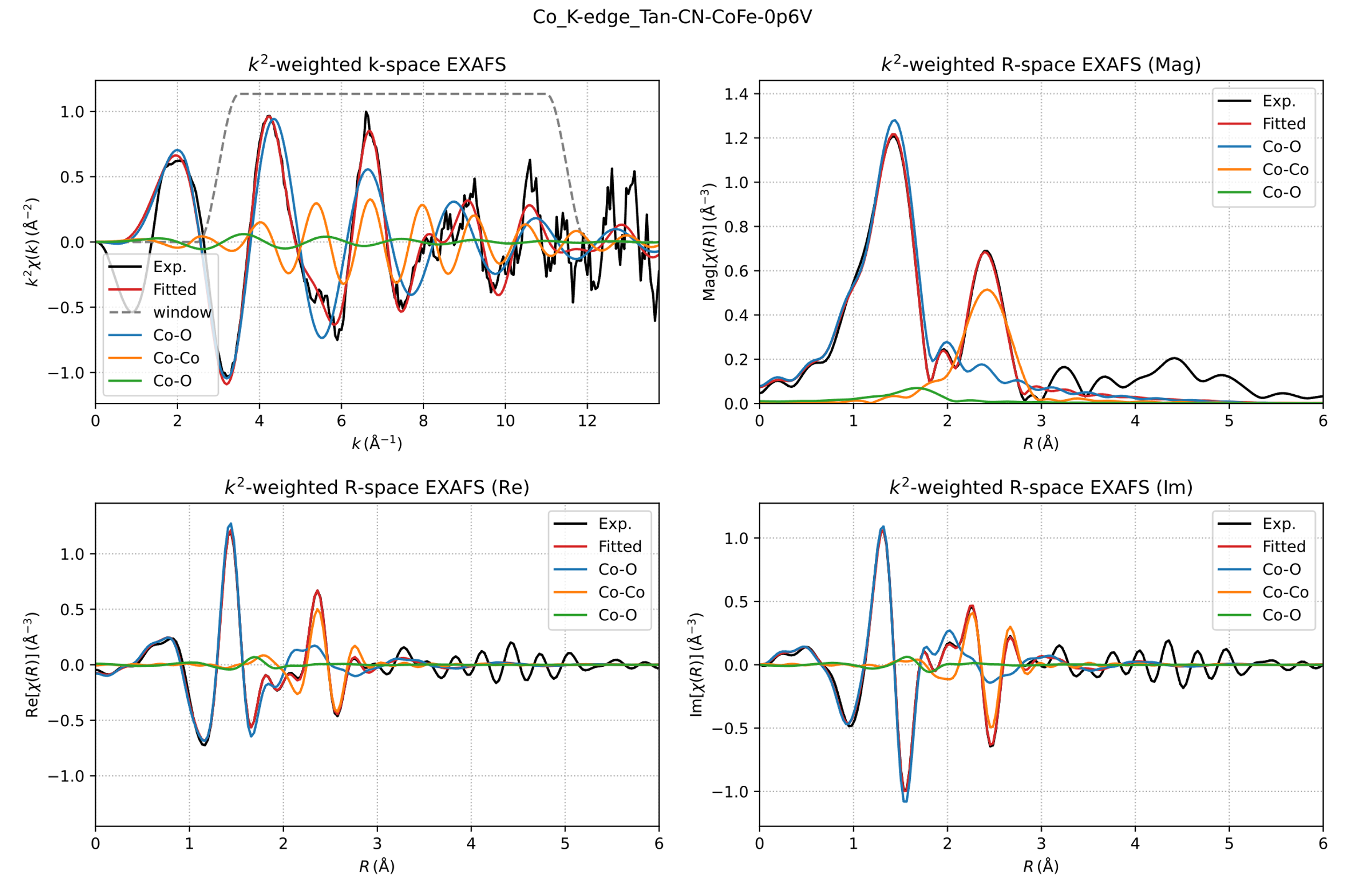
**

**Figure S70.** Fitting results of Co K-edge *k*^2^-weighted k-space and R-space FT-EXAFS spectra of Tan-CN-CoFe at 1.5 V vs. RHE in (a) k-space, (b) R-space magnitude, (c) R-space real part and (d) R-space imaginary part. The R-space spectra are plotted without phase correction.

**
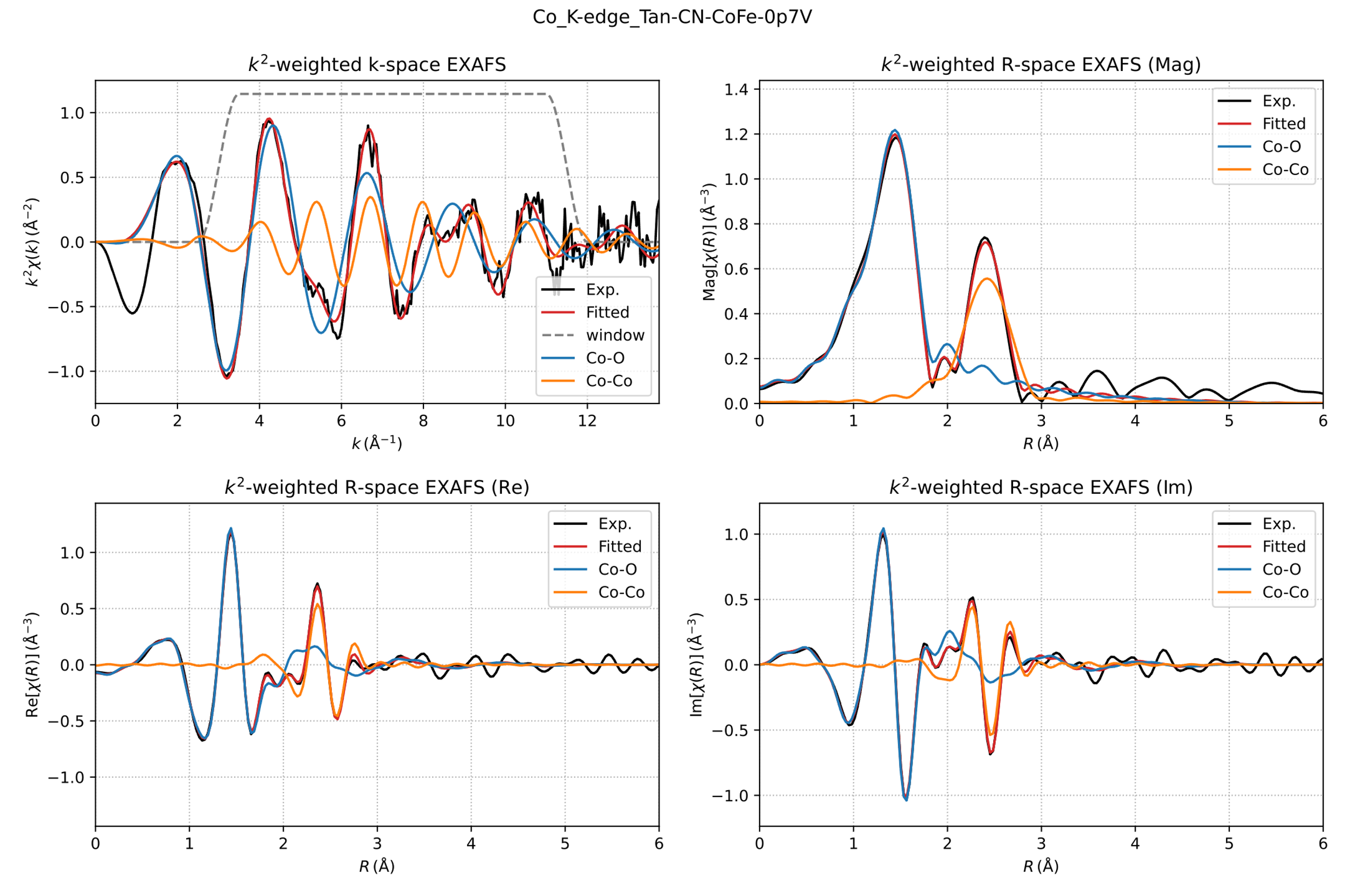
**

**Figure S71.** Fitting results of Co K-edge *k*^2^-weighted k-space and R-space FT-EXAFS spectra of Tan-CN-CoFe at 1.6 V vs. RHE in (a) k-space, (b) R-space magnitude, (c) R-space real part and (d) R-space imaginary part. The R-space spectra are plotted without phase correction.

**
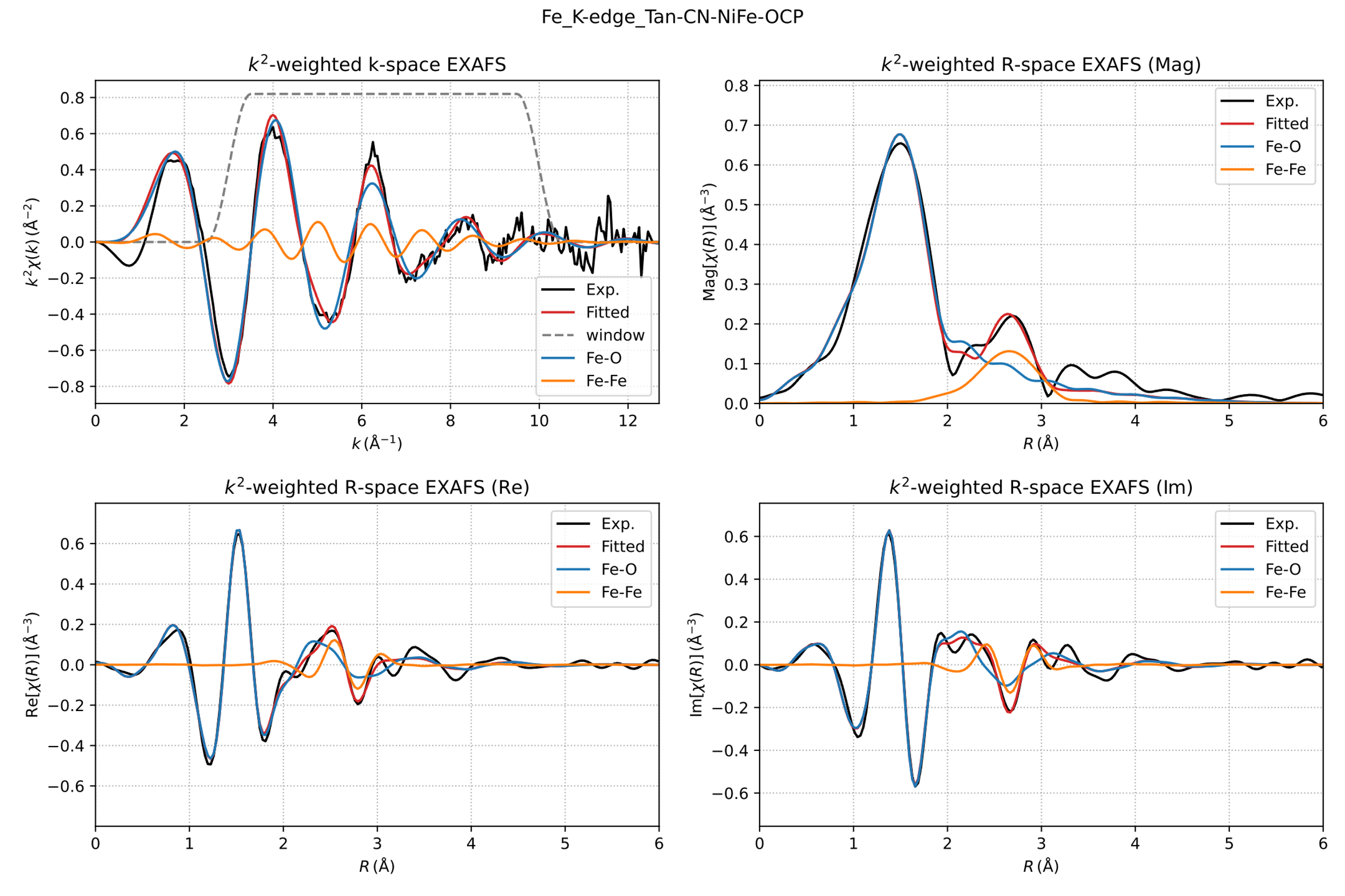
**

**Figure S72.** Fitting results of Fe K-edge *k*^2^-weighted k-space and R-space FT-EXAFS spectra of Tan-CN-NiFe under OCP conditions in (a) k-space, (b) R-space magnitude, (c) R-space real part and (d) R-space imaginary part. The R-space spectra are plotted without phase correction.

**
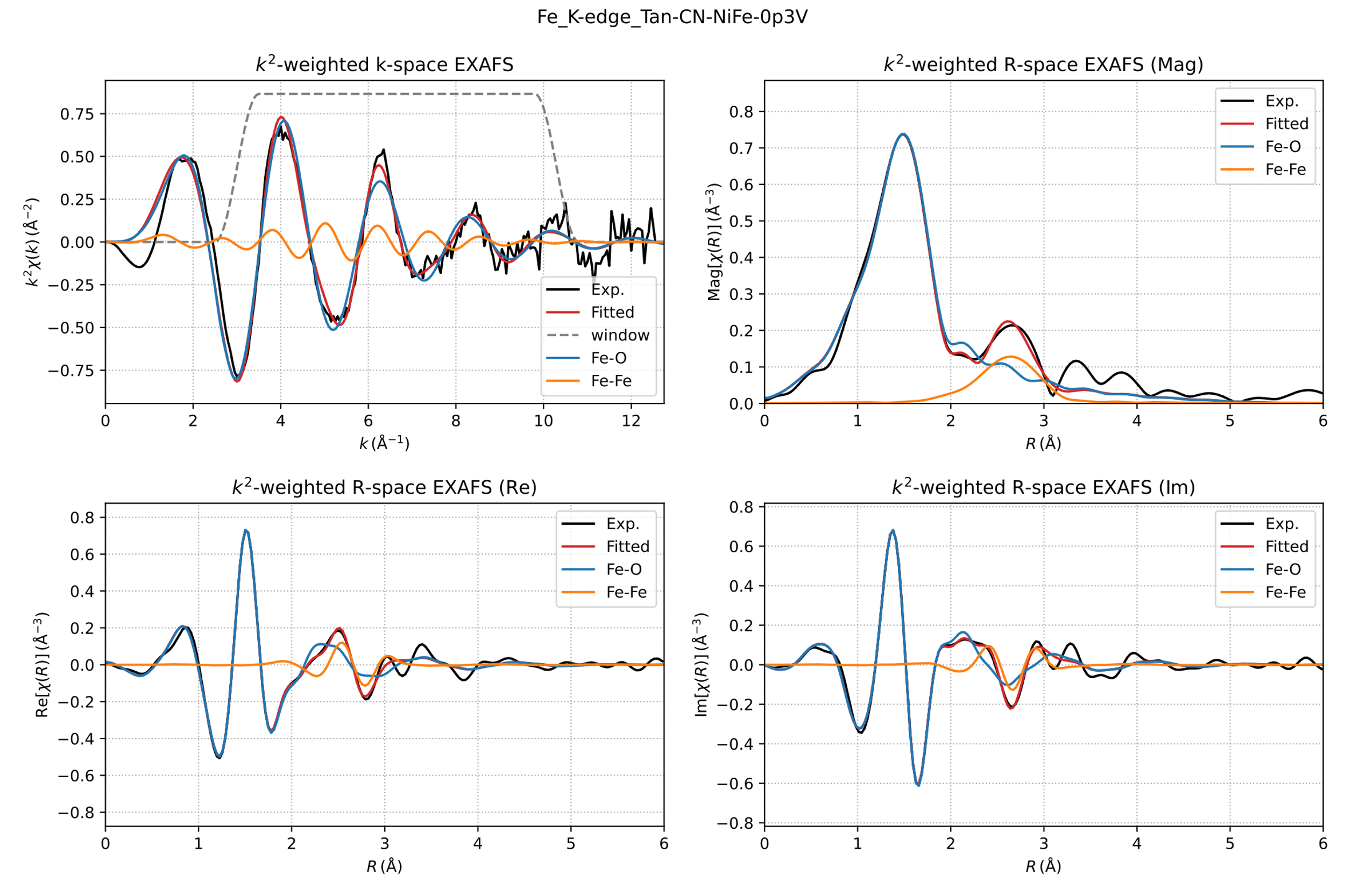
**

**Figure S73.** Fitting results of Fe K-edge *k*^2^-weighted k-space and R-space FT-EXAFS spectra of Tan-CN-NiFe at 1.2 V vs. RHE in (a) k-space, (b) R-space magnitude, (c) R-space real part and (d) R-space imaginary part. The R-space spectra are plotted without phase correction.

**
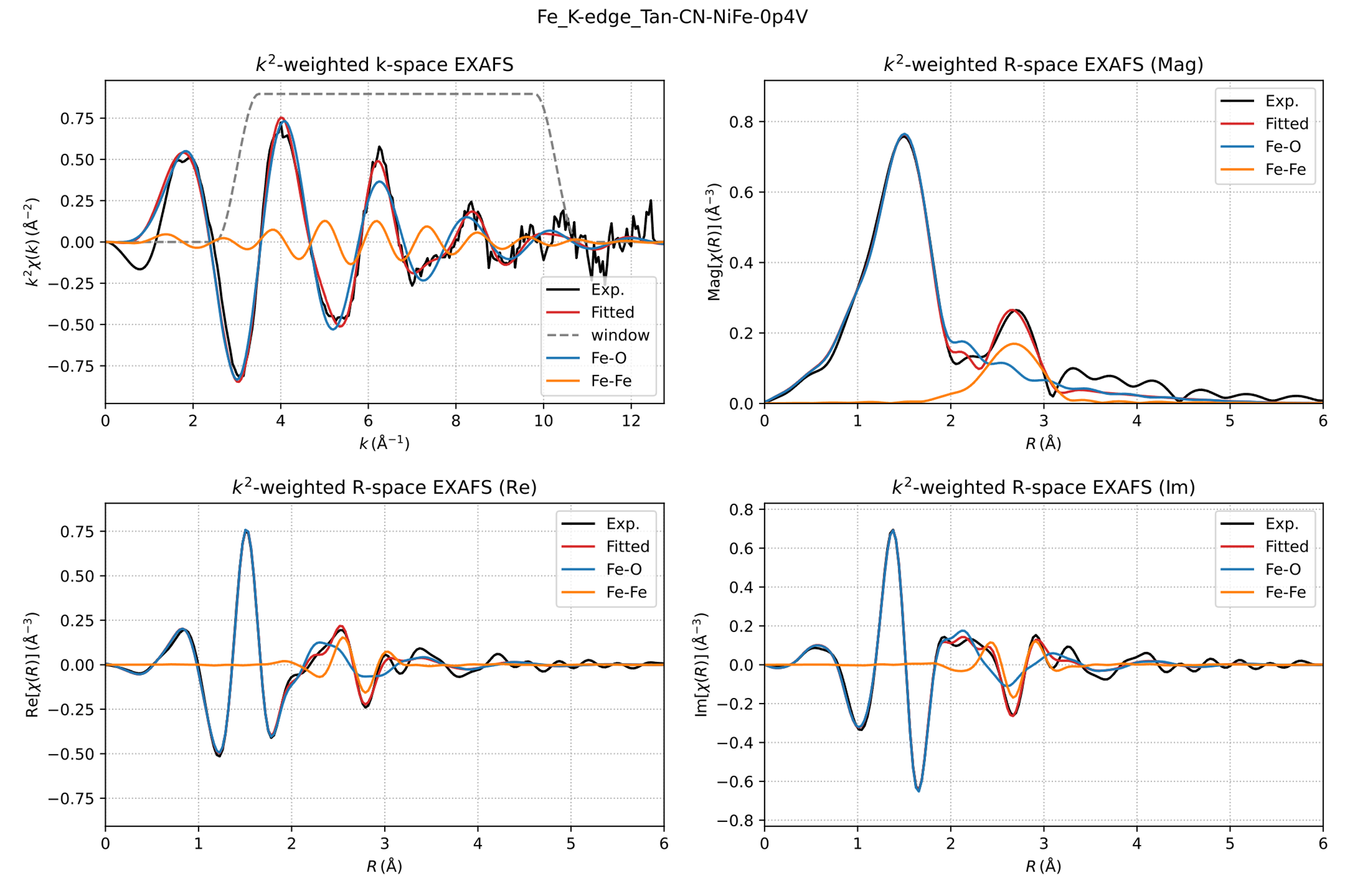
**

**Figure S74.** Fitting results of Fe K-edge *k*^2^-weighted k-space and R-space FT-EXAFS spectra of Tan-CN-NiFe at 1.3 V vs. RHE in (a) k-space, (b) R-space magnitude, (c) R-space real part and (d) R-space imaginary part. The R-space spectra are plotted without phase correction.

**
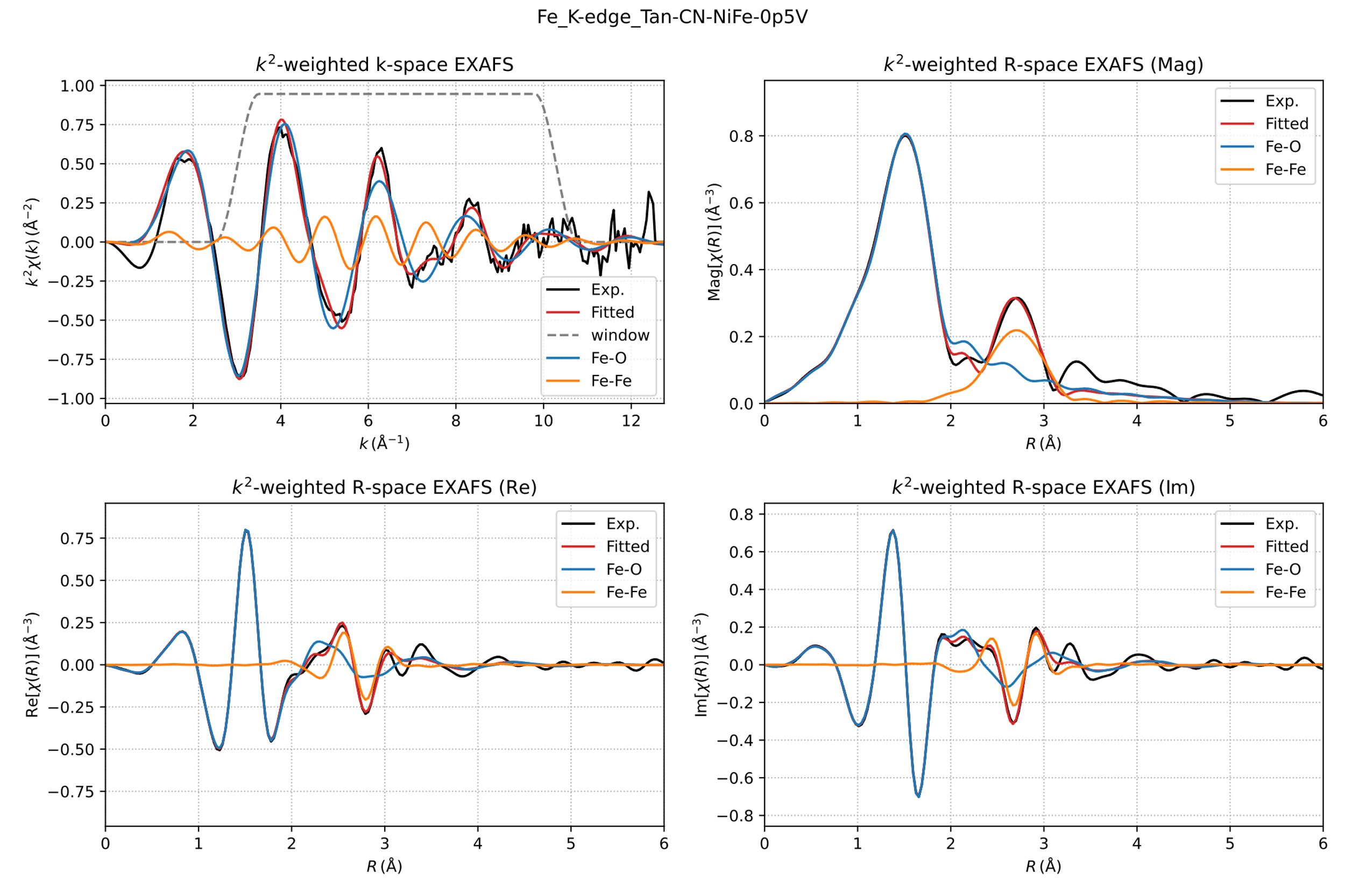
**

**Figure S75.** Fitting results of Fe K-edge *k*^2^-weighted k-space and R-space FT-EXAFS spectra of Tan-CN-NiFe at 1.4 V vs. RHE in (a) k-space, (b) R-space magnitude, (c) R-space real part and (d) R-space imaginary part. The R-space spectra are plotted without phase correction.

**
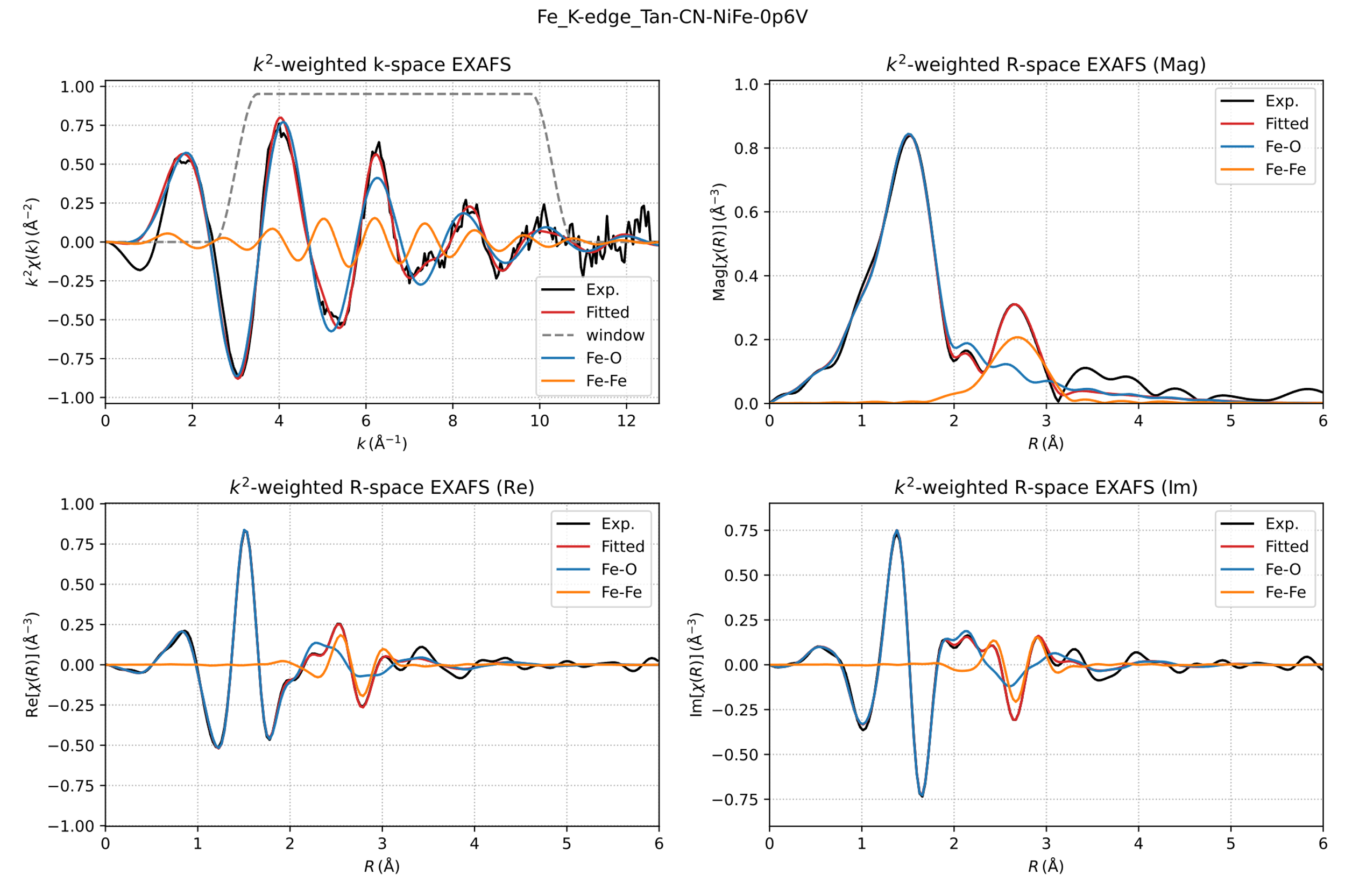
**

**Figure S76.** Fitting results of Fe K-edge *k*^2^-weighted k-space and R-space FT-EXAFS spectra of Tan-CN-NiFe at 1.5 V vs. RHE in (a) k-space, (b) R-space magnitude, (c) R-space real part and (d) R-space imaginary part. The R-space spectra are plotted without phase correction.

**
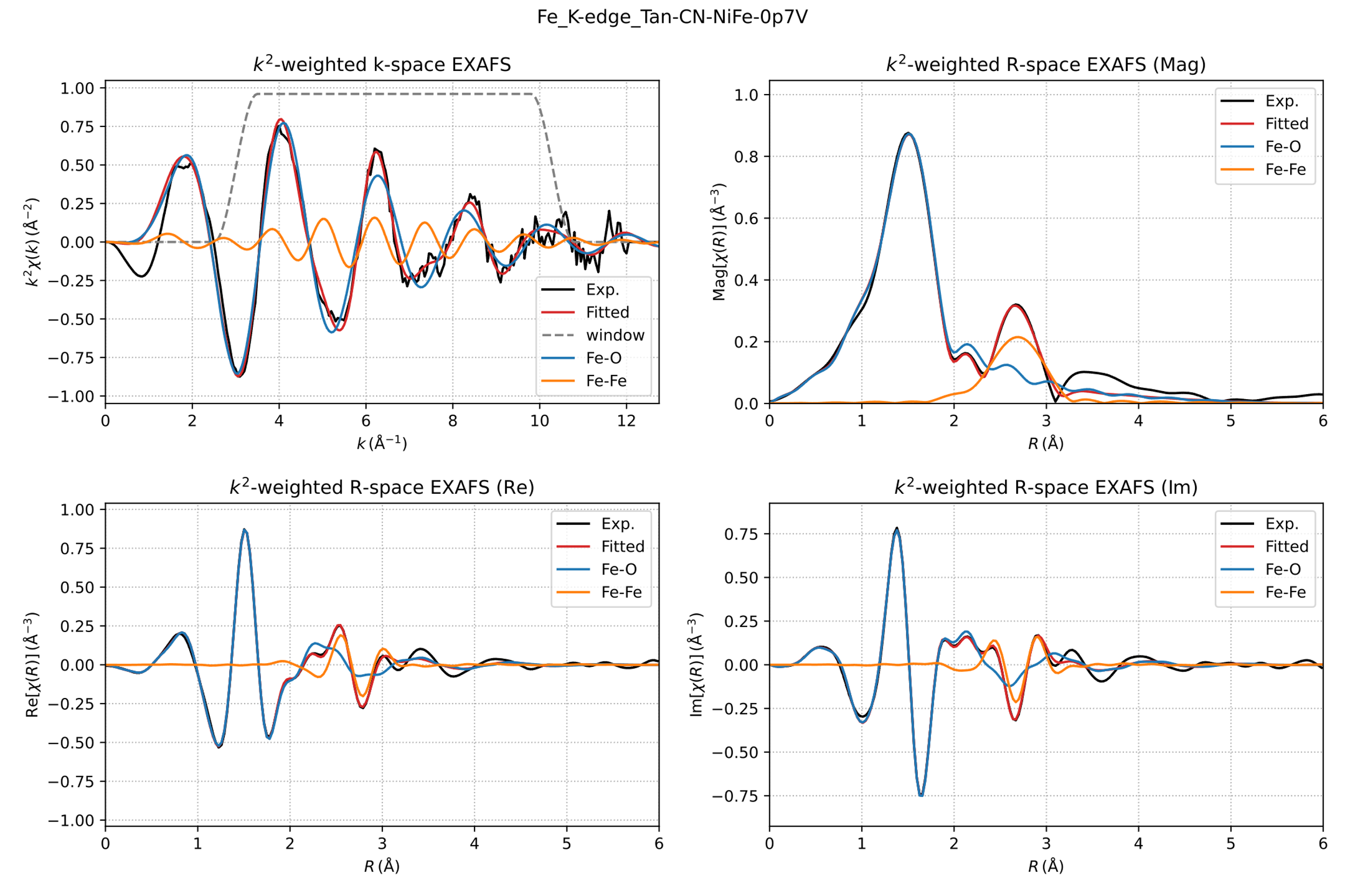
**

**Figure S77.** Fitting results of Fe K-edge *k*^2^-weighted k-space and R-space FT-EXAFS spectra of Tan-CN-NiFe at 1.6 V vs. RHE in (a) k-space, (b) R-space magnitude, (c) R-space real part and (d) R-space imaginary part. The R-space spectra are plotted without phase correction.

**
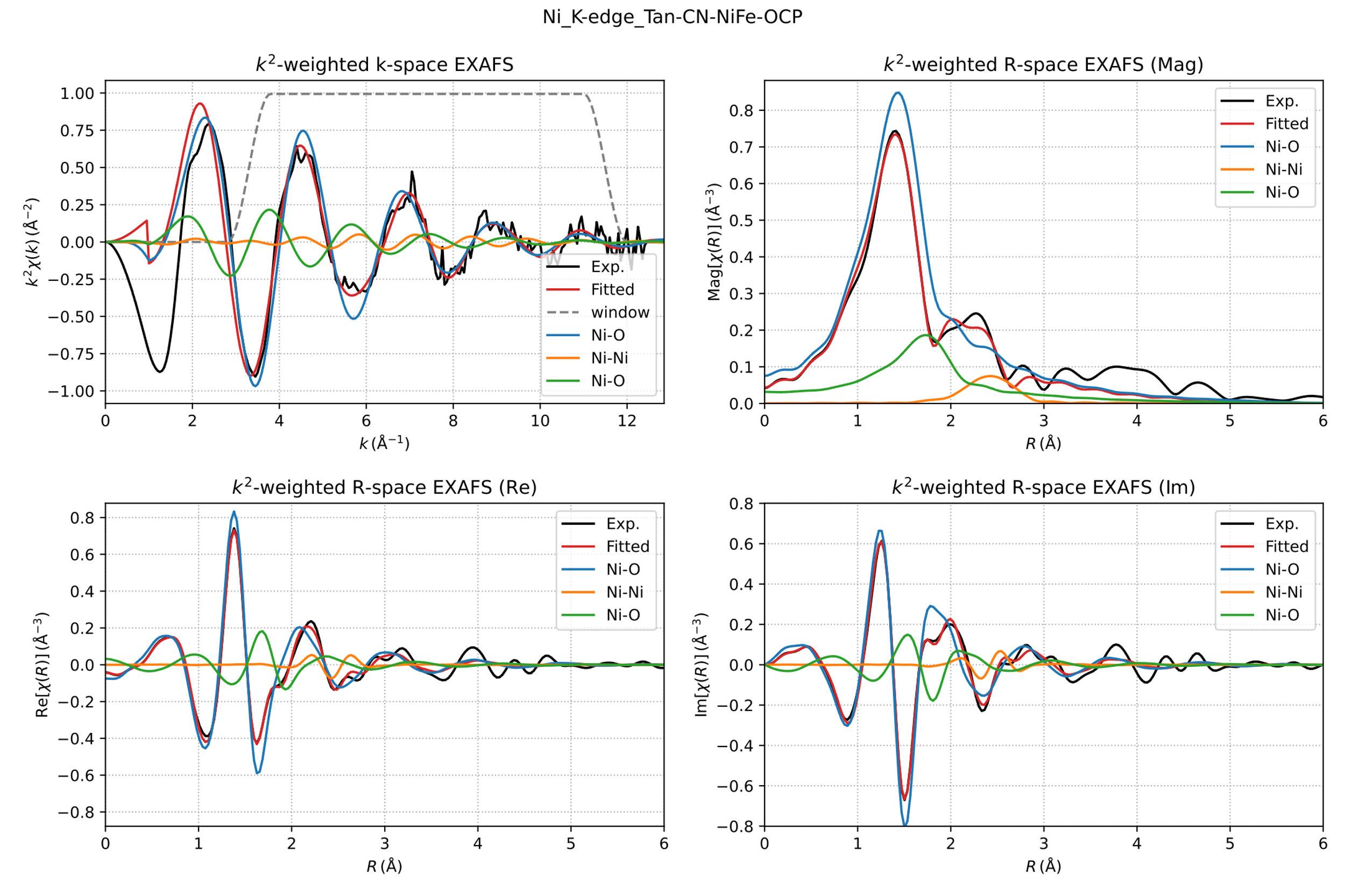
**

**Figure S78.** Fitting results of Ni K-edge *k*^2^-weighted k-space and R-space FT-EXAFS spectra of Tan-CN-NiFe under OCP conditions in (a) k-space, (b) R-space magnitude, (c) R-space real part and (d) R-space imaginary part. The R-space spectra are plotted without phase correction.

**
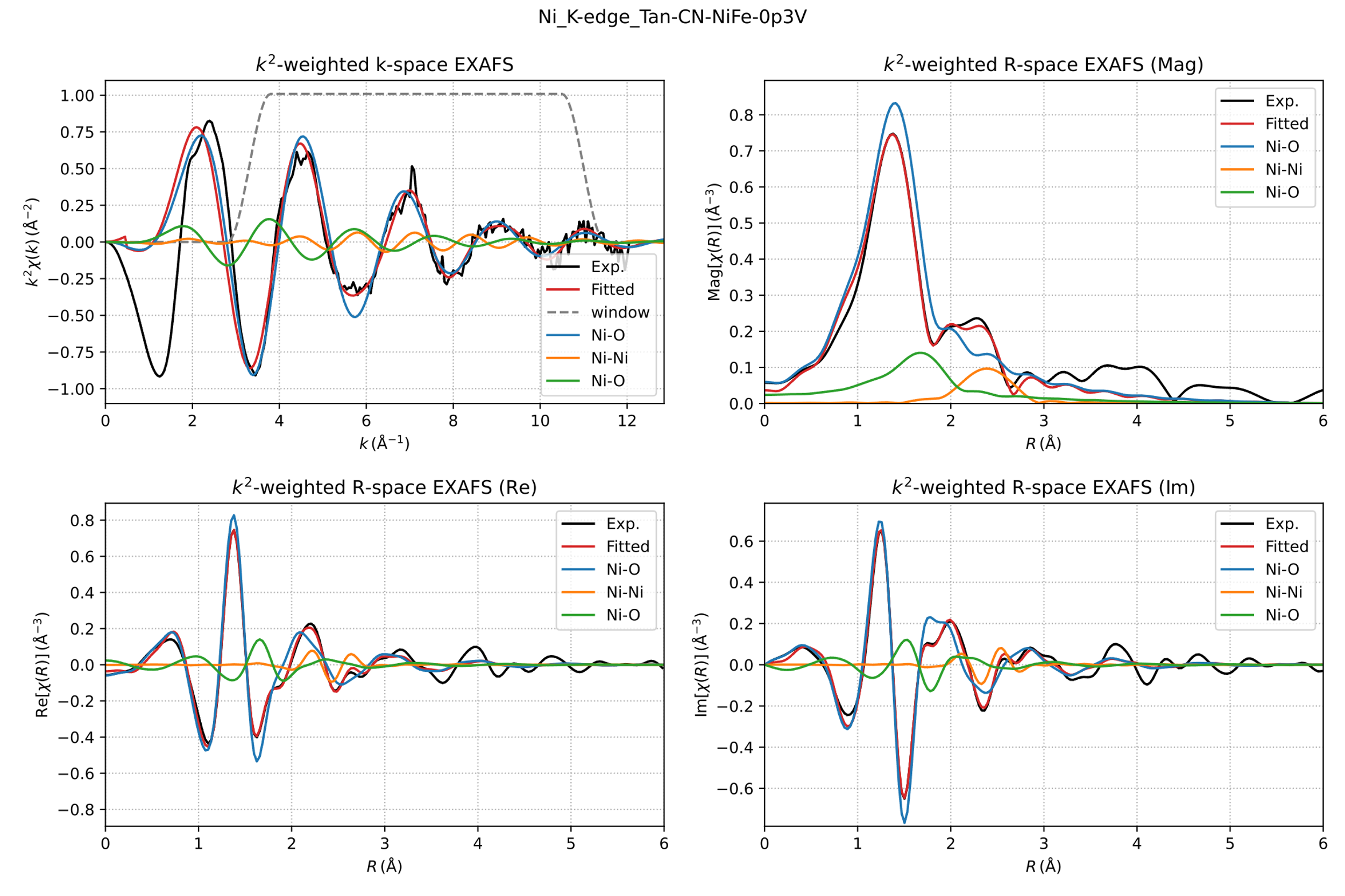
**

**Figure S79.** Fitting results of Ni K-edge *k*^2^-weighted k-space and R-space FT-EXAFS spectra of Tan-CN-NiFe at 1.2 V vs. RHE in (a) k-space, (b) R-space magnitude, (c) R-space real part and (d) R-space imaginary part. The R-space spectra are plotted without phase correction.

**
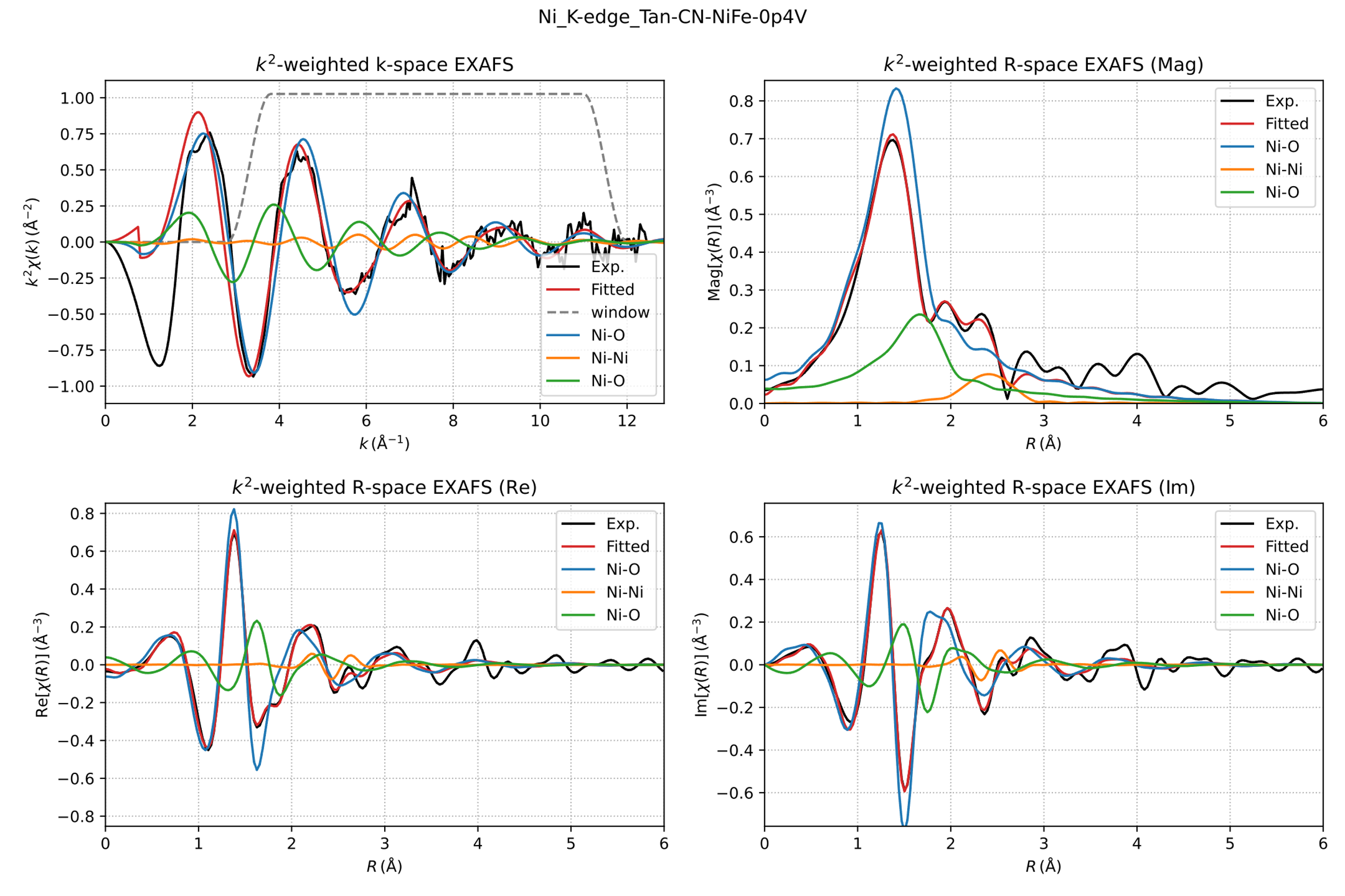
**

**Figure S80.** Fitting results of Ni K-edge *k*^2^-weighted k-space and R-space FT-EXAFS spectra of Tan-CN-NiFe at 1.3 V vs. RHE in (a) k-space, (b) R-space magnitude, (c) R-space real part and (d) R-space imaginary part. The R-space spectra are plotted without phase correction.

**
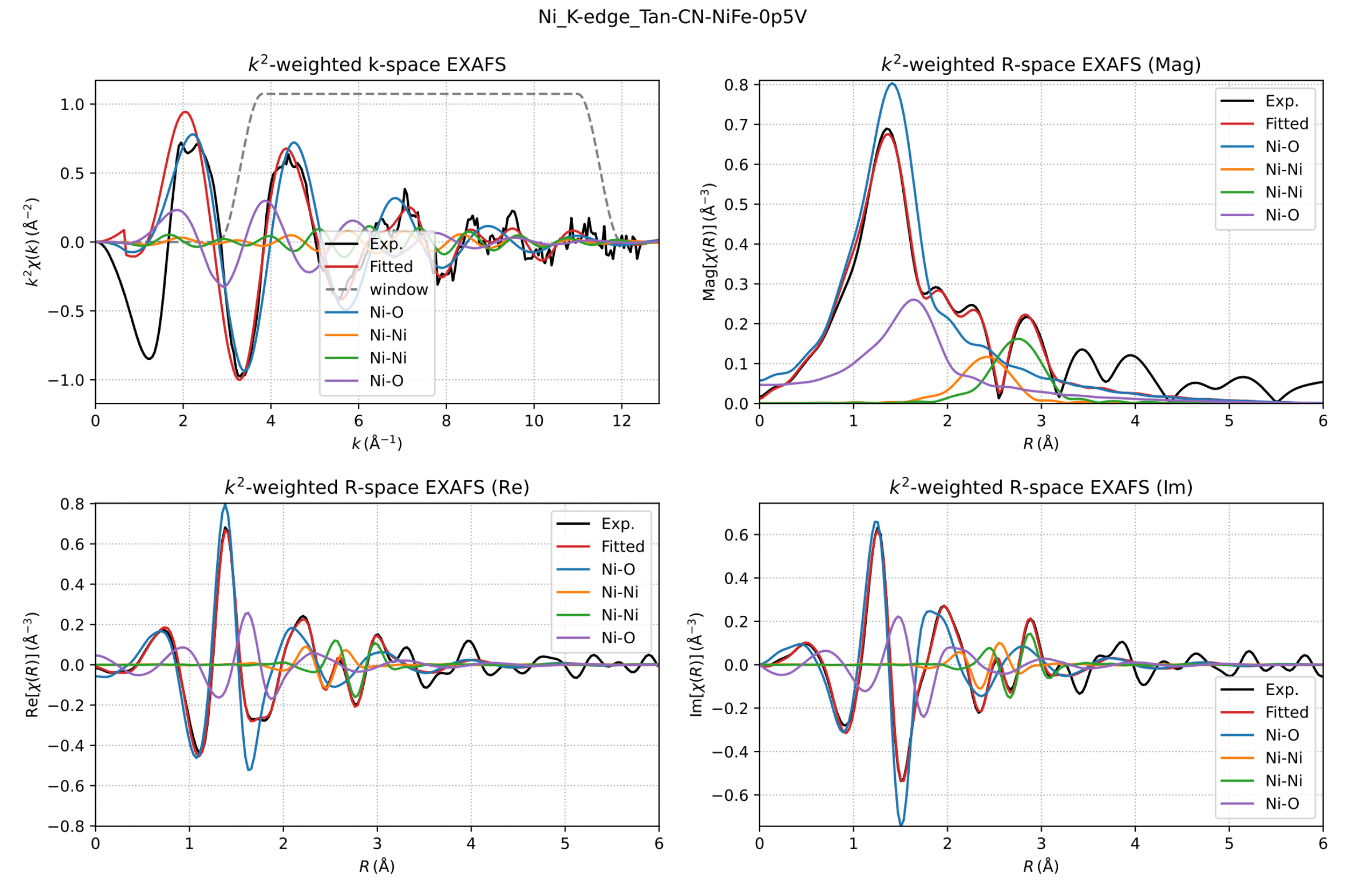
**

**Figure S81.** Fitting results of Ni K-edge *k*^2^-weighted k-space and R-space FT-EXAFS spectra of Tan-CN-NiFe at 1.4 V vs. RHE in (a) k-space, (b) R-space magnitude, (c) R-space real part and (d) R-space imaginary part. The R-space spectra are plotted without phase correction.


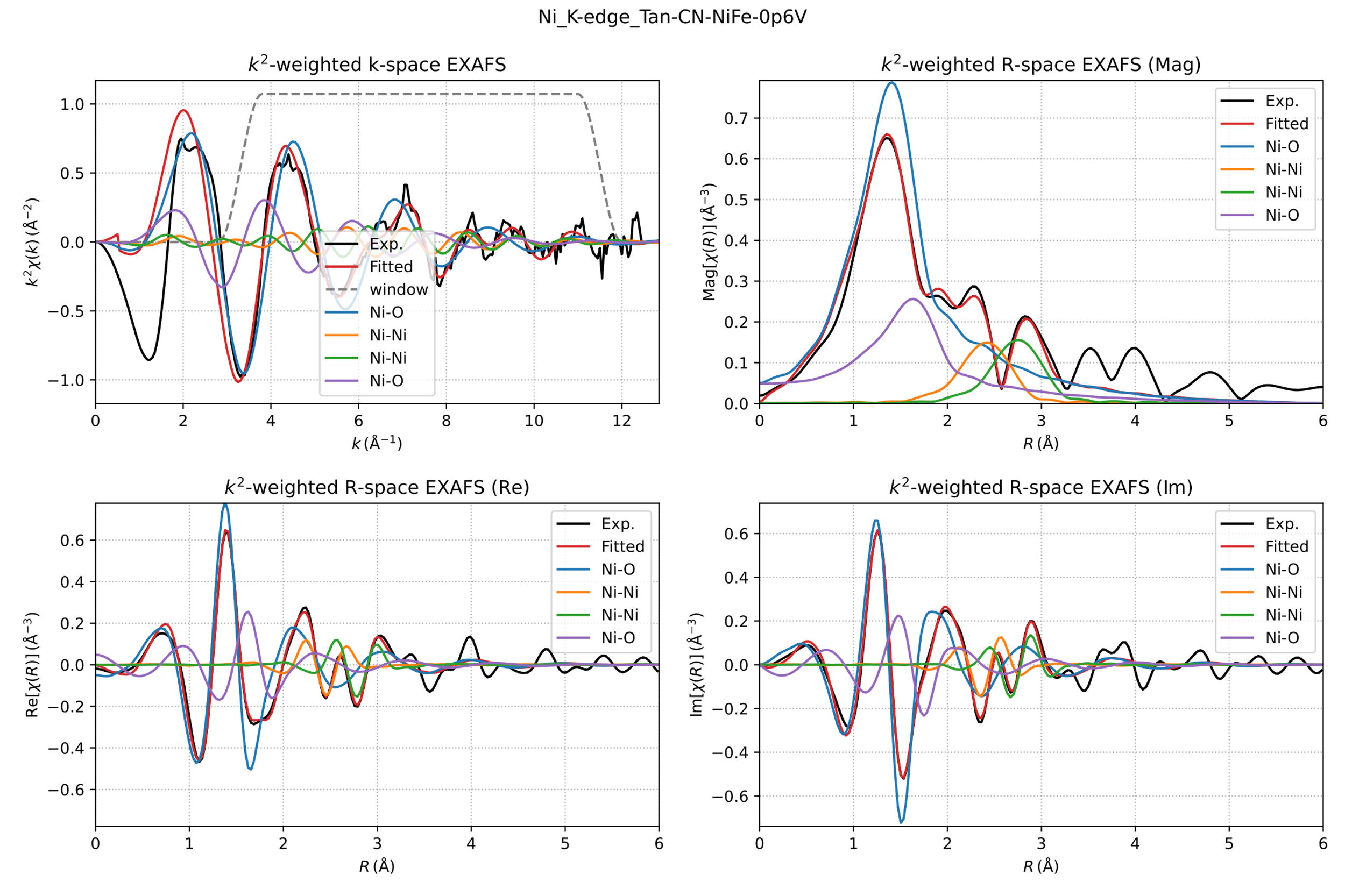


**Figure S82.** Fitting results of Ni K-edge *k*^2^-weighted k-space and R-space FT-EXAFS spectra of Tan-CN-NiFe at 1.5 V vs. RHE in (a) k-space, (b) R-space magnitude, (c) R-space real part and (d) R-space imaginary part. The R-space spectra are plotted without phase correction.

**
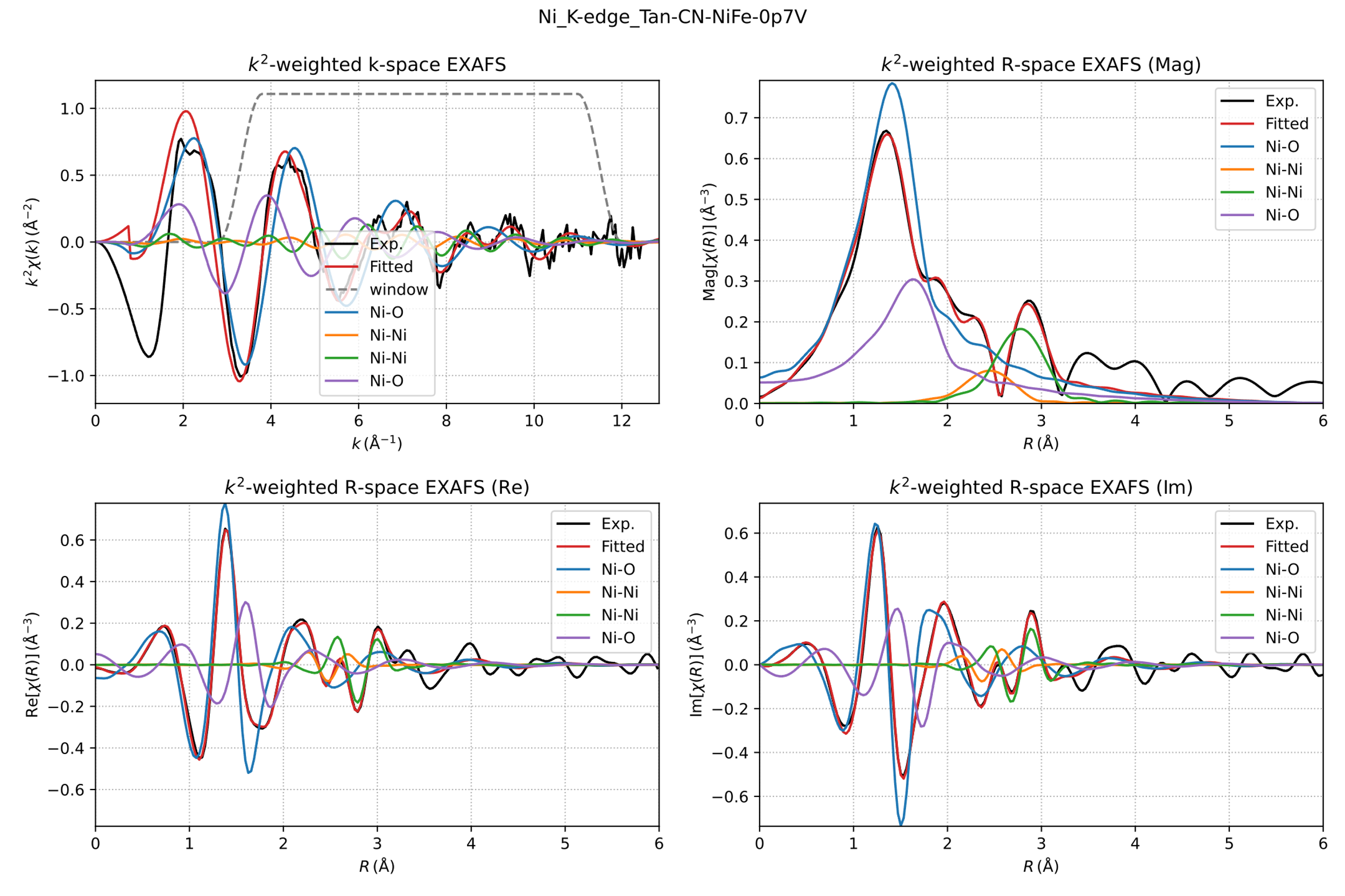
**

**Figure S83.** Fitting results of Ni K-edge *k*^2^-weighted k-space and R-space FT-EXAFS spectra of Tan-CN-NiFe at 1.6 V vs. RHE in (a) k-space, (b) R-space magnitude, (c) R-space real part and (d) R-space imaginary part. The R-space spectra are plotted without phase correction.


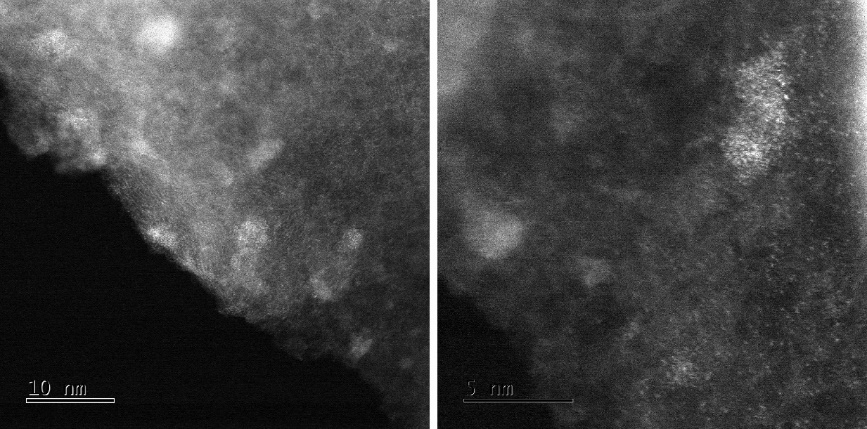


**Figure S84.** HAADF-STEM images of Tan-CN-NiFe after in situ XAS measurements.


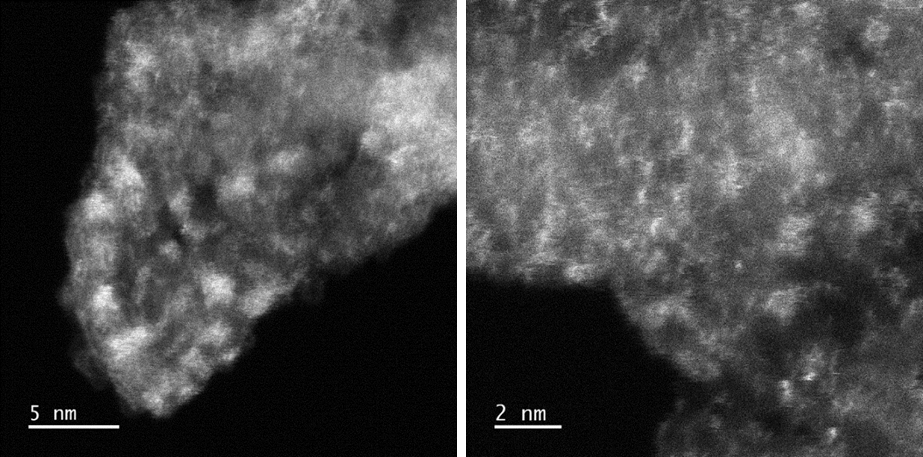


**Figure S85.** HAADF-STEM images of Tan-CN-Fe after in situ XAS measurements.


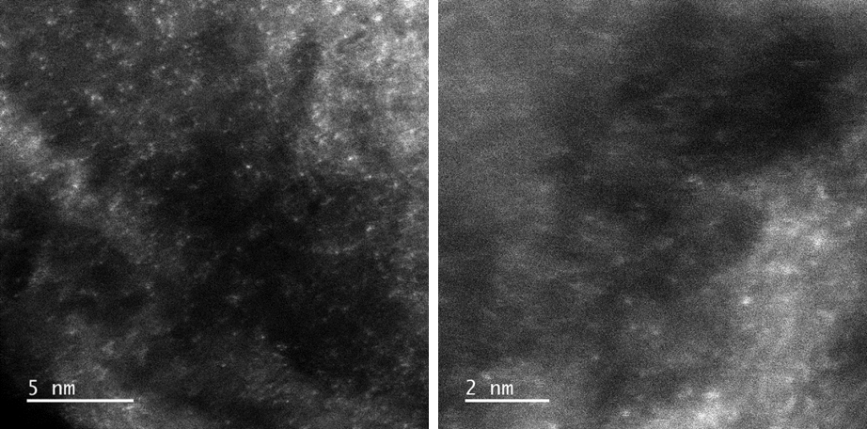


**Figure S86.** HAADF-STEM images of Tan-CN-Co after in situ XAS measurements.


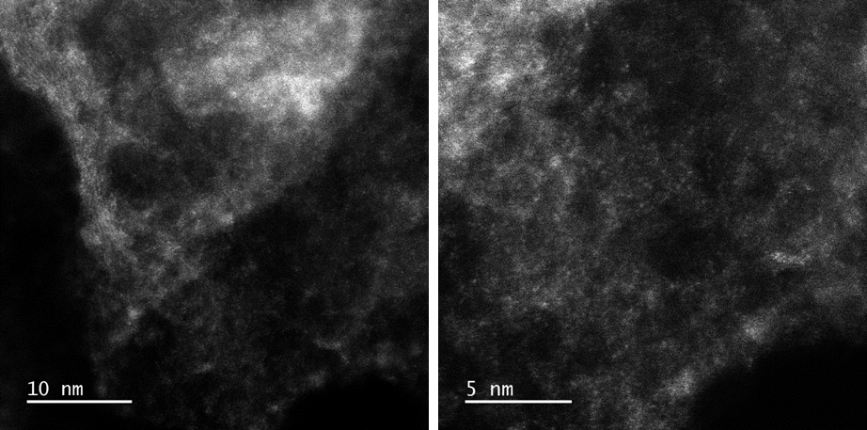


**Figure S87.** HAADF-STEM images of Tan-CN-Ni after in situ XAS measurements.


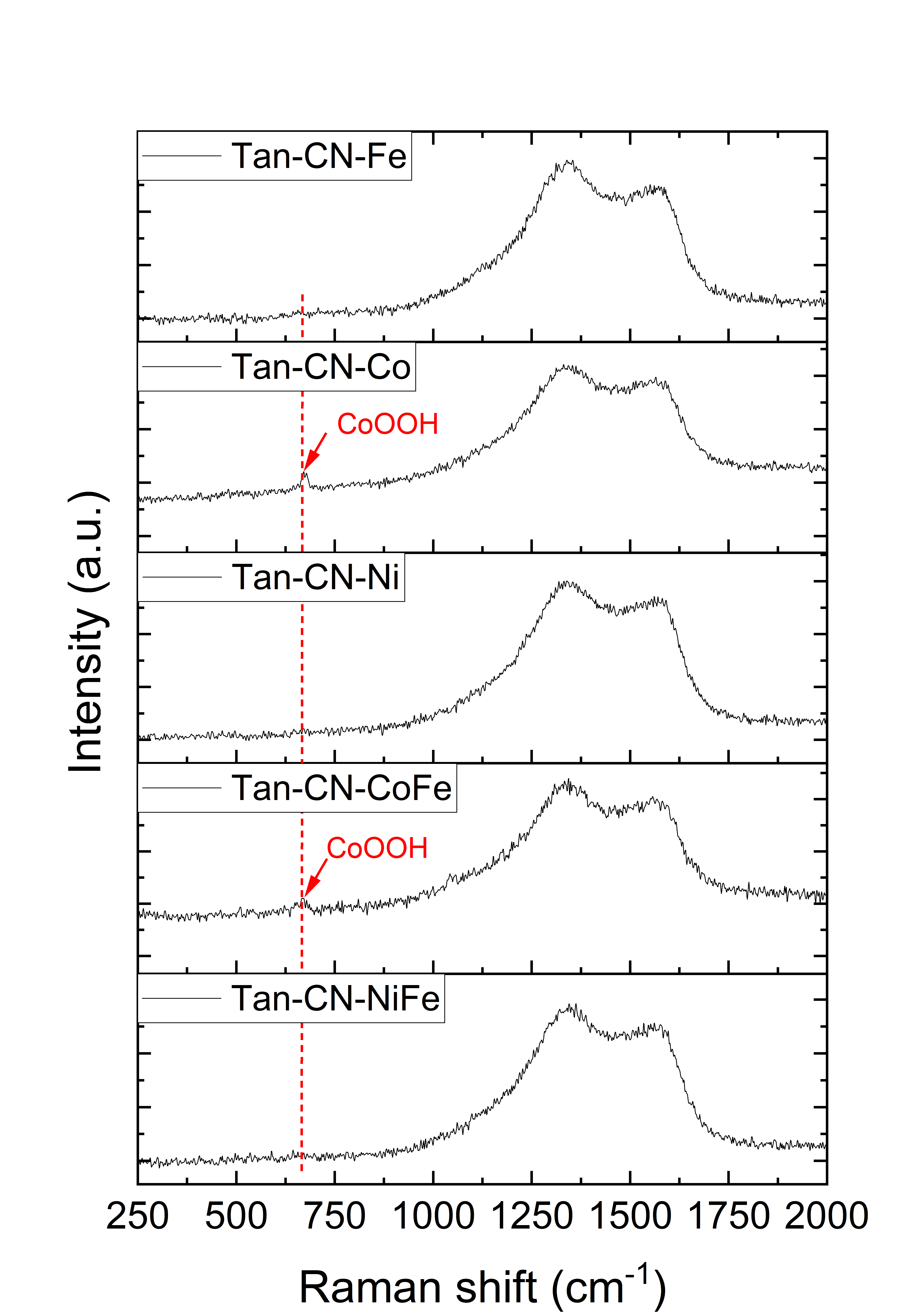


**Figure S88.** Raman spectra of the catalysts after in situ XAS measurements.


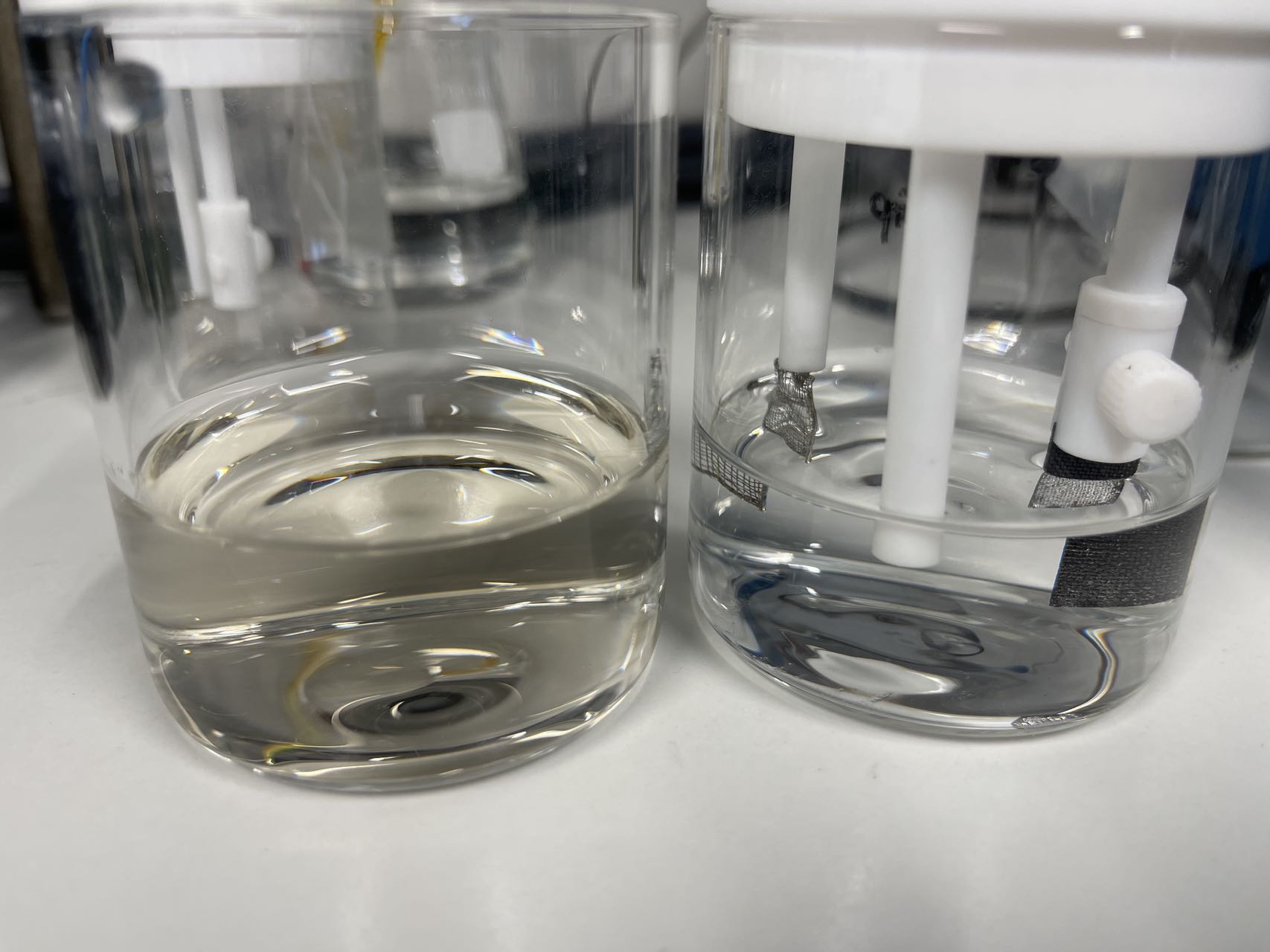


**Figure S89.** Picture of the spent electrolyte (1M KOH) after 50 hours chronopotentiometry measurements for Tan-CN-CoFe catalyst (left) and the fresh electrolyte (right).

.
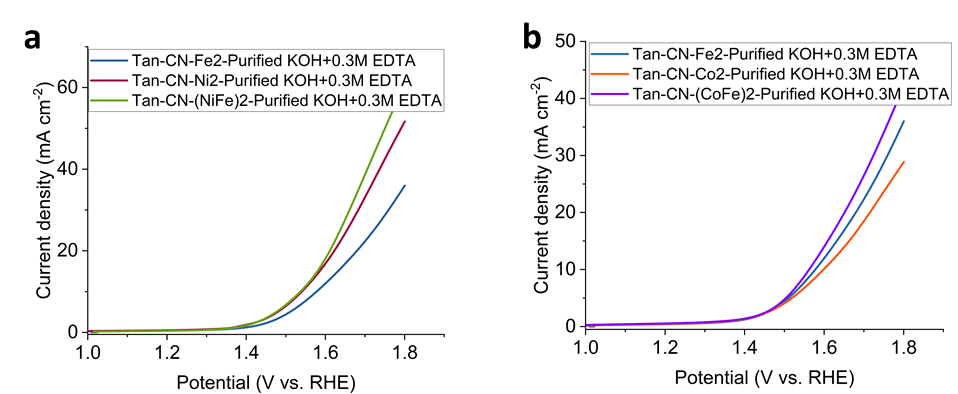


**Figure S90.** Electrochemical tests of all the catalysts in purified 1 M KOH with an additional 0.3 M EDTA.


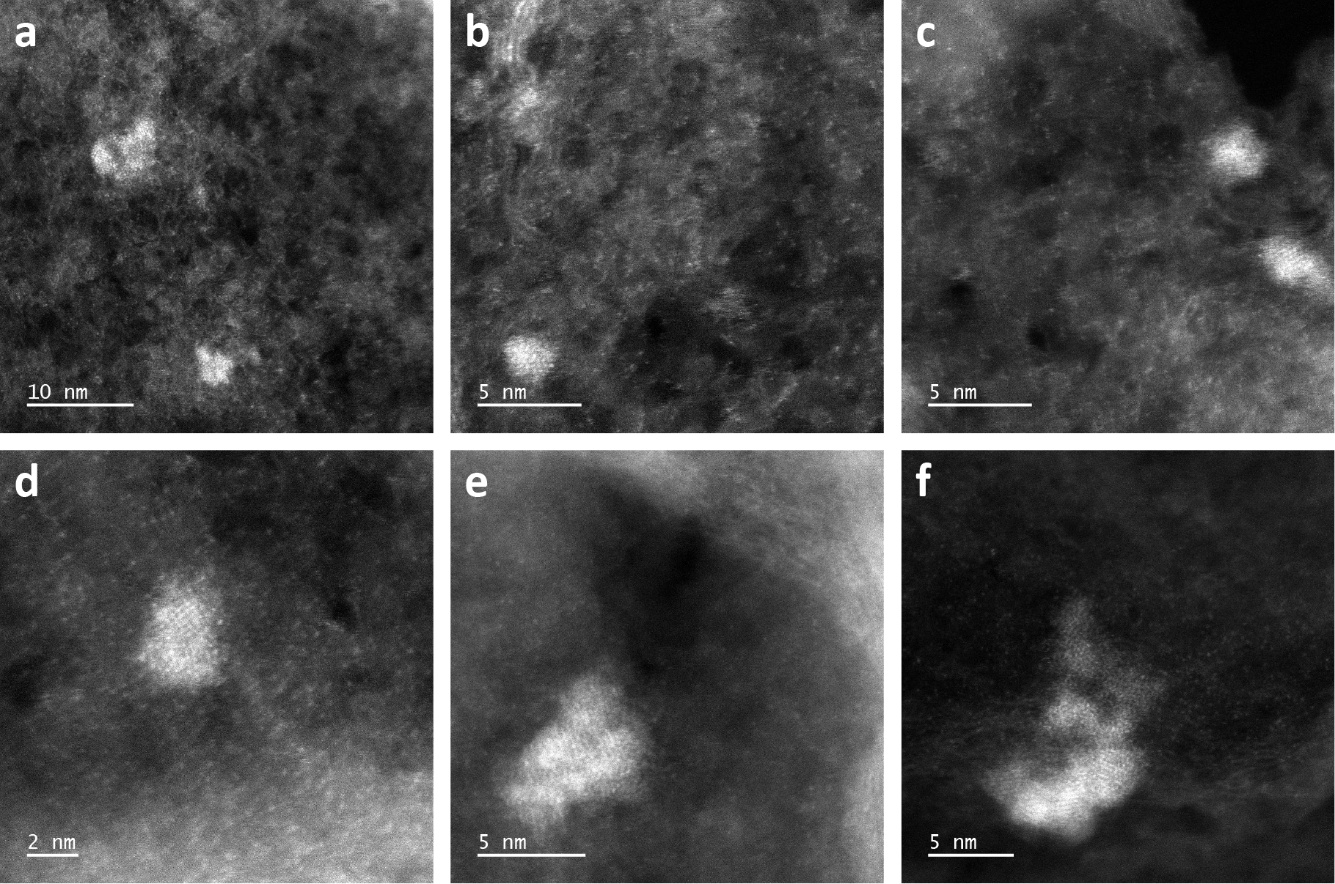


**Figure S91.** HAADF-STEM images of (a-c) Tan-CN-CoFe and (d-f) Tan-CN-NiFe after OER in purified 1 M KOH with an additional 0.3 M EDTA.


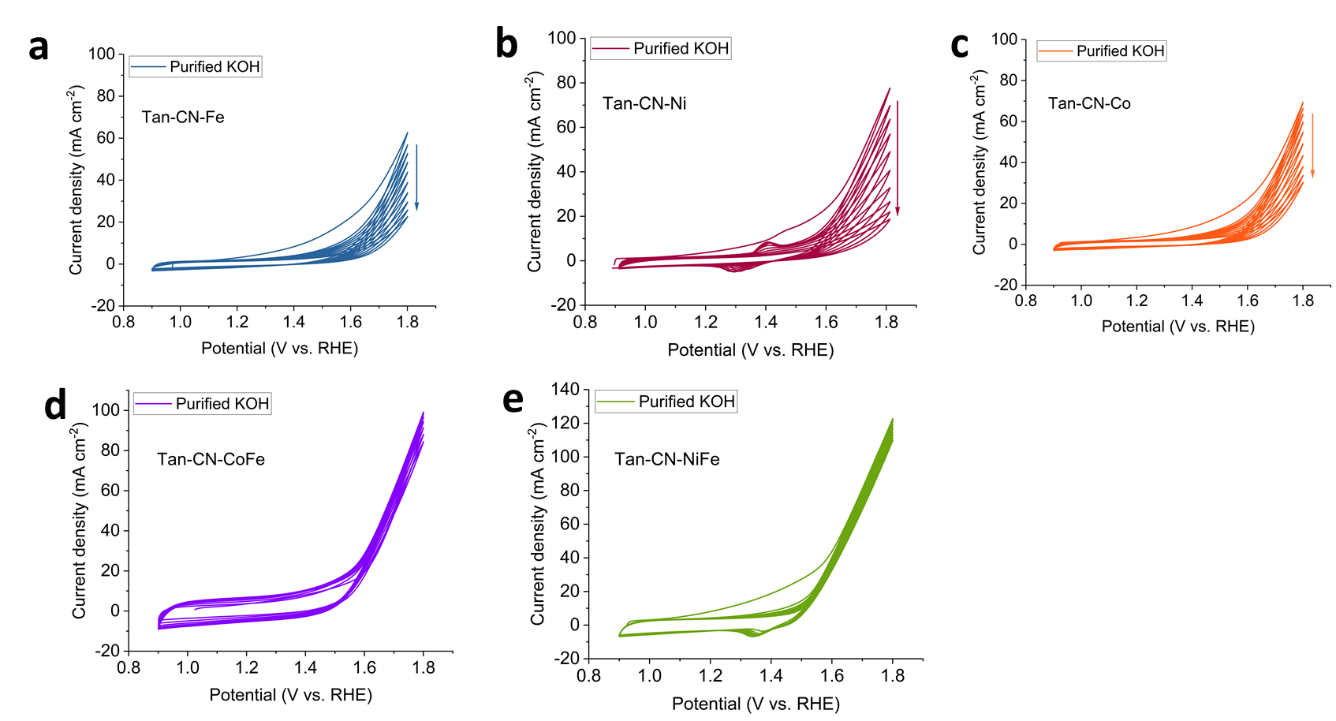


**Figure S92.** CV testes of all the SACs in 1M purified KOH using the method 2.

# 6. Supplementary Tables

**Table S1.** Elemental compositions of the catalysts determined XRF and EDS.

| **Catalysts** | **XRF (wt.%)** | | **EDS (wt. %)** | | | | |
| --- | --- | --- | --- | --- | --- | --- | --- |
|  |  |  | **Metal** | | **C** | **N** | **O** |
| **Tan-CN-Fe** | Fe:2.25 | | 0.76 | | 84.47 | 8.89 | 5.87 |
| **Tan-CN-Co** | Co:2.08 | | 3.10 | | 78.13 | 11.81 | 6.95 |
| **Tan-CN-Ni** | Ni:3.22 | | 2.47 | | 74.98 | 12.78 | 9.75 |
| **Tan-CN-CoFe** | Co:1.52 | Fe: 0.74 | Co:1.63 | Fe:0.91 | 80.86 | 9.7 | 6.72 |
| **Tan-CN-NiFe** | Ni:1.63 | Fe:0.53 | Ni:3.00 | Fe:2.23 | 79.7 | 10.84 | 4.23 |

**Table S2.** Fitting parameters of all *ex situ* samples. Main interatomic distances, atomic coordination numbers (C) and Debye-Waller factors (σ^2^) calculated from Artemis fitting of the experimental FT|*k*^2^c(*k*)| spectra. *E*_0_ denotes the threshold energy which is the starting point for the photoelectron’s kinetic energy calculation. The XAFS data for these *ex situ* samples were collected at the P65 beamline of PETRA III (see more details in the Materials Characterizations section).

| **Sample** | **Edge** | **Path** | **CN** | **R [Å]** | **σ^2^ [Å^2^]** | **E_0_ [eV]** | **R-factor** |
| --- | --- | --- | --- | --- | --- | --- | --- |
| **Fe foil** | Fe K-edge | Fe-Fe | 8 (fixed) | 2.46 ± 0.01 | 0.0050 ± 0.0009 | 7116.9 ± 1.4 | 0.0101 |
|  |  | Fe-Fe | 6 (fixed) | 2.84 ± 0.01 |  |  |  |
| **Tan-CN-CoFe** | Fe K-edge | Fe-N | 2.1 ± 0.3 | 1.96 ± 0.02 | 0.0030 (fixed) | 7122.2 ± 1.2 | 0.0048 |
|  |  | Fe-N | 2.6 ± 0.3 | 2.11 ± 0.02 |  |  |  |
|  |  | Fe-C | 1.0 ± 0.3 | 3.04 ± 0.04 |  |  |  |
| **Tan-CN-Fe** | Fe K-edge | Fe-N | 2.1 ± 0.3 | 1.93 ± 0.02 | 0.0030 (fixed) | 7123.1 ± 1.4 | 0.0044 |
|  |  | Fe-N | 2.6 ± 0.4 | 2.08 ± 0.02 |  |  |  |
|  |  | Fe-C | 1.1 ± 0.4 | 3.04 ± 0.04 |  |  |  |
| **Tan-CN-NiFe** | Fe K-edge | Fe-N | 1.9 ± 0.2 | 1.96 ± 0.02 | 0.0030 (fixed) | 7122 ± 1.0 | 0.0034 |
|  |  | Fe-N | 2.4 ± 0.3 | 2.11 ± 0.02 |  |  |  |
|  |  | Fe-C | 1.0 ± 0.3 | 3.04 ± 0.03 |  |  |  |
| **Co foil** | Co K-edge | Co-Co | 12 (fixed) | 2.49 ± 0.01 | 0.0063 ± 0.0002 | 7716.3 ± 0.3 | 0.0014 |
| **Tan-CN-CoFe** | Co K-edge | Co-N | 2.4 ± 0.5 | 1.86 ± 0.02 | 0.0057 ± 0.0022 | 7715.3 ± 2.0 | 0.0117 |
|  |  | Co-N | 1.6 ± 0.3 | 2.06 ± 0.03 |  |  |  |
|  |  | Co-C | 1.4 ± 0.6 | 2.82 ± 0.02 |  |  |  |
| **Tan-CN-Co** | Co K-edge | Co-N | 2.4 ± 0.5 | 1.90 ± 0.02 | 0.0052 ± 0.0023 | 7716.9 ± 2.0 | 0.0144 |
|  |  | Co-N | 1.8 ± 0.3 | 2.08 ± 0.03 |  |  |  |
|  |  | Co-C | 1.6 ± 0.7 | 2.86 ± 0.02 |  |  |  |
| **Ni foil** | Ni K-edge | Ni-Ni | 12 (fixed) | 2.48 ± 0.01 | 0.0058 ± 0.0003 | 8339.2 ± 0.4 | 0.0023 |
| **Tan-CN-NiFe** | Ni K-edge | Ni-N | 3.7 ± 0.4 | 1.87 ± 0.01 | 0.0069 ± 0.0014 | 8338.7 ± 0.3 | 0.0074 |
|  |  | Ni-C | 1.6 ± 0.3 | 2.78 ± 0.03 |  |  |  |
| **Tan-CN-Ni** | Ni K-edge | Ni-N | 3.8 ± 0.4 | 1.88 ± 0.01 | 0.0085 ± 0.0015 | 8338.3 ± 1.4 | 0.0078 |
|  |  | Ni-C | 1.8 ± 0.4 | 2.78 ± 0.03 |  |  |  |

**Table S3.** Fitting parameters of all *in situ* samples. Main interatomic distances, atomic coordination numbers (C.N.) and Debye-Waller factors (σ^2^) calculated from Artemis fitting of the experimental FT|*k*^2^c(*k*)| spectra. *E*_0_ denotes the threshold energy which is the starting point for the photoelectron’s kinetic energy calculation. The XAFS data for these *in situ* samples were collected at the SAMBA beamline of SOLEIL (see more details in the Materials Characterizations section).

| **Sample** | **Edge** | **Path** | **C.N.** | **R [Å]** | **σ^2^ [Å^2^]** | **E_0_ [eV]** | **R-factor** |
| --- | --- | --- | --- | --- | --- | --- | --- |
| **Fe foil** | Fe K-edge | Fe-Fe | 8 (fixed) | 2.46 ± 0.01 | 0.0052 ± 0.0007 | 7117.1 ± 1.2 | 0.0070 |
|  |  | Fe-Fe | 6 (fixed) | 2.84 ± 0.01 |  |  |  |
| **Tan-CN-Fe_OCP** | Fe K-edge | Fe- N/O | 4.5 ± 1.1 | 1.95 ± 0.02 | 0.0128 ± 0.0036 | -7119.8 ± 2.8 | 0.0216 |
|  |  | Fe-Fe | 0.9 ± 0.5 | 3.00 ± 0.05 |  |  |  |
| **Tan-CN-Fe_1.2 V** | Fe K-edge | Fe- N/O | 4.0 ± 0.4 | 1.96 ± 0.01 | 0.0116 ± 0.0016 | 7121.2 ± 1.1 | 0.0081 |
|  |  | Fe-Fe | 1.1 ± 0.3 | 3.01 ± 0.02 | 0.0136 ± 0.0026 |  |  |
| **Tan-CN-Fe_1.4 V** | Fe K-edge | Fe- N/O | 4.4 ± 0.6 | 1.96 ± 0.01 | 0.0122 ± 0.0024 | 7121.3 ± 1.6 | 0.0137 |
|  |  | Fe-Fe | 1.3 ± 0.4 | 3.02 ± 0.03 | 0.0137 ± 0.0024 |  |  |
| **Tan-CN-Fe_1.6 V** | Fe K-edge | Fe- N/O | 4.3 ± 0.2 | 1.97 ± 0.01 | 0.0110 ± 0.0008 | 7122.5 ± 0.6 | 0.0024 |
|  |  | Fe-Fe | 1.4 ± 0.2 | 3.03 ± 0.01 | 0.0126 ± 0.0016 |  |  |
| **Tan-CN-Fe_1.7 V** | Fe K-edge | Fe- N/O | 4.3 ± 0.3 | 1.97 ± 0.01 | 0.0102 ± 0.0012 | 7123.1 ± 0.8 | 0.0090 |
|  |  | Fe-Fe | 1.6 ± 0.2 | 3.04 ± 0.01 | 0.0112 ± 0.0016 |  |  |
| **Tan-CN-CoFe_OCP** | Fe K-edge | Fe- N/O | 4.5 ± 0.2 | 1.96 ± 0.01 | 0.0104 ± 0.0009 | 7120.4 ± 0.7 | 0.0019 |
|  |  | Fe-Fe/Co | 0.9 ± 0.4 | 3.00 ± 0.02 | 0.0147 ± 0.0062 |  |  |
| **Tan-CN-CoFe_1.2 V** | Fe K-edge | Fe- N/O | 4.2 ± 0.2 | 1.96 ± 0.01 | 0.0093 ± 0.0009 | 7121.8 ± 0.6 | 0.0029 |
|  |  | Fe-Fe/Co | 1.2 ± 0.4 | 3.00 ± 0.02 | 0.0121 ± 0.0040 |  |  |
| **Tan-CN-CoFe_1.3 V** | Fe K-edge | Fe- N/O | 4.6 ± 0.4 | 1.96 ± 0.01 | 0.0095 ± 0.0014 | 7122.2 ± 1.0 | 0.0077 |
|  |  | Fe-Fe/Co | 1.5 ± 0.7 | 2.97 ± 0.02 | 0.0138 ± 0.0065 |  |  |
| **Tan-CN-CoFe_1.4 V** | Fe K-edge | Fe- N/O | 4.4 ± 0.3 | 1.96 ± 0.01 | 0.0078 ± 0.0011 | 7123.1 ± 0.8 | 0.0061 |
|  |  | Fe-Fe/Co | 1.8 ± 0.8 | 2.98 ± 0.02 | 0.0143 ± 0.0054 |  |  |
| **Tan-CN-CoFe_1.5 V** | Fe K-edge | Fe- N/O | 4.5 ± 0.3 | 1.97 ± 0.01 | 0.0085 ± 0.0010 | 7123.4 ± 0.7 | 0.0041 |
|  |  | Fe-Fe/Co | 1.9 ± 0.6 | 3.00 ± 0.02 | 0.0137 ± 0.0039 |  |  |
| **Tan-CN-CoFe_1.6 V** | Fe K-edge | Fe- N/O | 4.6 ± 0.4 | 1.96 ± 0.01 | 0.0087 ± 0.0013 | 7122.8 ± 0.9 | 0.0072 |
|  |  | Fe- Fe/Co | 2.3 ± 0.9 | 2.98 ± 0.02 | 0.0161 ± 0.0056 |  |  |
| **Tan-CN-NiFe_OCP** | Fe K-edge | Fe- N/O | 4.0 ± 0.6 | 2.00 ± 0.01 | 0.0106 ± 0.0023 | 7121.7 ± 1.6 | 0.0150 |
|  |  | Fe-Fe/Ni | 1.6 ± 0.6 | 3.08 ± 0.04 | 0.0170 ± 0.0035 |  |  |
| **Tan-CN-NiFe_1.2 V** | Fe K-edge | Fe- N/O | 4.1 ± 0.2 | 1.99 ± 0.01 | 0.0096 ± 0.0008 | 7121.2 ± 0.6 | 0.0025 |
|  |  | Fe-Fe/Ni | 1.6 ± 0.2 | 3.07 ± 0.01 | 0.0177 ± 0.0015 |  |  |
| **Tan-CN-NiFe_1.3 V** | Fe K-edge | Fe- N/O | 4.1 ± 0.3 | 1.99 ± 0.01 | 0.0096 ± 0.0012 | 7122.3 ± 0.9 | 0.0054 |
|  |  | Fe-Fe/Ni | 1.5 ± 0.3 | 3.09 ± 0.02 | 0.0134 ± 0.0016 |  |  |
| **Tan-CN-NiFe_1.4 V** | Fe K-edge | Fe- N/O | 4.1 ± 0.2 | 2.00 ± 0.01 | 0.0088 ± 0.0009 | 7123.3 ± 0.6 | 0.0035 |
|  |  | Fe-Fe/Ni | 1.8 ± 0.3 | 3.10 ± 0.01 | 0.0128 ± 0.0014 |  |  |
| **Tan-CN-NiFe_1.5 V** | Fe K-edge | Fe- N/O | 4.0 ± 0.2 | 1.99 ± 0.01 | 0.0080 ± 0.0006 | 7122.9 ± 0.5 | 0.0016 |
|  |  | Fe-Fe/Ni | 1.6 ± 0.2 | 3.09 ± 0.01 | 0.0120 ± 0.0009 |  |  |
| **Tan-CN-NiFe_1.6 V** | Fe K-edge | Fe- N/O | 3.9 ± 0.2 | 1.99 ± 0.01 | 0.0070 ± 0.0006 | 7122.9 ± 0.5 | 0.0016 |
|  |  | Fe-Fe/Ni | 1.5 ± 0.2 | 3.09 ± 0.01 | 0.0111 ± 0.0010 |  |  |
| **Co foil** | Co K-edge | Co-Co | 12 (fixed) | 2.49 ± 0.01 | 0.0060 ± 0.0004 | 7716.0 ± 0.5 | 0.0023 |
| **Tan-CN-Co_OCP** | Co K-edge | Co- N/O | 3.2 ± 0.2 | 1.91 ± 0.01 | 0.0059 ± 0.0007 | 7718.5 ± 0.6 | 0.0023 |
|  |  | Co- N/O | 0.8 ± 0.2 | 2.15 ± 0.02 |  |  |  |
|  |  | Co-Co | 0.9 ± 0.1 | 2.84 ± 0.01 |  |  |  |
| **Tan-CN-Co_1.2 V** | Co K-edge | Co- N/O | 3.4 ± 0.2 | 1.91 ± 0.01 | 0.0066 ± 0.0008 | 7718.4 ± 0.6 | 0.0021 |
|  |  | Co- N/O | 0.5 ± 0.2 | 2.16 ± 0.03 |  |  |  |
|  |  | Co-Co | 1.1 ± 0.1 | 2.83 ± 0.01 |  |  |  |
| **Tan-CN-Co_1.4 V** | Co K-edge | Co- N/O | 3.5 ± 0.2 | 1.91 ± 0.01 | 0.0056 ± 0.0006 | 7719.1 ± 0.5 | 0.0017 |
|  |  | Co- N/O | 0.4 ± 0.2 | 2.15 ± 0.03 |  |  |  |
|  |  | Co-Co | 1.6 ± 0.1 | 2.83 ± 0.01 |  |  |  |
| **Tan-CN-Co_1.5 V** | Co K-edge | Co- N/O | 3.7 ± 0.2 | 1.91 ± 0.01 | 0.0055 ± 0.0006 | 7719.3 ± 0.5 | 0.0017 |
|  |  | Co- N/O | 0.5 ± 0.2 | 2.16 ± 0.03 |  |  |  |
|  |  | Co-Co | 1.6 ± 0.2 | 2.84 ± 0.01 |  |  |  |
| **Tan-CN-Co_1.6 V** | Co K-edge | Co- N/O | 3.8 ± 0.2 | 1.91 ± 0.01 | 0.0050 ± 0.0005 | 7719.6 ± 0.4 | 0.0012 |
|  |  | Co- N/O | 0.5 ± 0.2 | 2.16 ± 0.02 |  |  |  |
|  |  | Co-Co | 1.8 ± 0.1 | 2.84 ± 0.01 |  |  |  |
| **Tan-CN-CoFe_OCP** | Co K-edge | Co-N/O | 3.5 ± 0.4 | 1.91 ± 0.01 | 0.0077 ± 0.0015 | 7717.4 ± 1.0 | 0.0016 |
|  |  | Co-N/O | 0.9 ± 0.2 | 2.16 ± 0.02 |  |  |  |
|  |  | Co-Co/Fe | 1.0 ± 0.3 | 2.86 ± 0.02 | 0.0155 ± 0.0031 |  |  |
| **Tan-CN-CoFe_1.2 V** | Co K-edge | Co-N/O | 3.6 ± 0.2 | 1.89 ± 0.01 | 0.0053 ± 0.0008 | 7716.5 ± 0.7 | 0.0046 |
|  |  | Co- Co/Fe | 1.9 ± 0.6 | 2.82 ± 0.01 | 0.0127 ± 0.0034 |  |  |
| **Tan-CN-CoFe_1.3 V** | Co K-edge | Co-N/O | 3.8 ± 0.2 | 1.89 ± 0.01 | 0.0035 ± 0.0006 | 7717.5 ± 0.6 | 0.0029 |
|  |  | Co- Co/Fe | 1.7 ± 0.3 | 2.81 ± 0.01 | 0.0064 ± 0.0017 |  |  |
| **Tan-CN-CoFe_1.4 V** | Co K-edge | Co-N/O | 4.4 ± 0.4 | 1.90 ± 0.01 | 0.0055 ± 0.0010 | 7718.6 ± 0.7 | 0.0063 |
|  |  | Co-N/O | 0.5 ± 0.3 | 2.19 ± 0.05 |  |  |  |
|  |  | Co- Co/Fe | 2.0 ± 0.3 | 2.84 ± 0.01 | 0.0069 ± 0.0010 |  |  |
| **Tan-CN-CoFe_1.5 V** | Co K-edge | Co-N/O | 4.2 ± 0.2 | 1.90 ± 0.01 | 0.0046 ± 0.0007 | 7717.8 ± 0.5 | 0.0022 |
|  |  | Co-N/O | 0.3 ± 0.2 | 2.14 ± 0.06 |  |  |  |
|  |  | Co- Co/Fe | 2.3 ± 0.2 | 2.83 ± 0.01 | 0.0069 ± 0.0008 |  |  |
| **Tan-CN-CoFe_1.6 V** | Co K-edge | Co-N/O | 4.1 ± 0.2 | 1.89 ± 0.01 | 0.0046 ± 0.0008 | 7717.8 ± 0.7 | 0.0040 |
|  |  | Co-Co/Fe | 2.3 ± 0.4 | 2.83 ± 0.01 | 0.0062 ± 0.0014 |  |  |
| **Ni foil** | Ni K-edge | Ni-Ni | 12 (fixed) | 2.48 ± 0.01 | 0.0060 ± 0.0004 | 8339.7 ± 0.5 | 0.0023 |
| **Tan-CN-Ni_OCP** | Ni K-edge | Ni- N/O | 4.2 ± 0.7 | 1.85 ± 0.02 | 0.0114 ± 0.0020 | 8338.1 ± 2.5 | 0.0026 |
|  |  | Ni- N/O | 0.9 ± 0.5 | 2.12 ± 0.05 |  |  |  |
|  |  | Ni-Ni | 0.5 ± 0.2 | 2.74 ± 0.03 |  |  |  |
| **Tan-CN-Ni_1.2 V** | Ni K-edge | Ni- N/O | 3.5 ± 0.4 | 1.85 ± 0.01 | 0.0084 ± 0.0015 | 8338.5 ± 1.1 | 0.0027 |
|  |  | Ni- N/O | 0.9 ± 0.2 | 2.07 ± 0.02 |  |  |  |
|  |  | Ni-Ni | 0.5 ± 0.1 | 2.74 ± 0.02 |  |  |  |
| **Tan-CN-Ni_1.4 V** | Ni K-edge | Ni- N/O | 3.9 ± 0.2 | 1.86 ± 0.01 | 0.0082 ± 0.0007 | 8340.4 ± 0.5 | 0.0011 |
|  |  | Ni- N/O | 0.5 ± 0.2 | 2.11 ± 0.02 |  |  |  |
|  |  | Ni-Ni | 1.5 ± 0.1 | 2.83 ± 0.01 | 0.0112 ± 0.0011 |  |  |
| **Tan-CN-Ni_1.5 V** | Ni K-edge | Ni- N/O | 3.5 ± 0.3 | 1.86 ± 0.01 | 0.0083 ± 0.0009 | 8340.2 ± 0.6 | 0.0036 |
|  |  | Ni- N/O | 1.4 ± 0.1 | 2.06 ± 0.01 |  |  |  |
|  |  | Ni-Ni | 0.9 ± 0.1 | 2.82 ± 0.01 |  |  |  |
|  |  | Ni-Ni | 0.7 ± 0.1 | 3.10 ± 0.02 |  |  |  |
| **Tan-CN-Ni_1.6 V** | Ni K-edge | Ni- N/O | 3.4 ± 0.2 | 1.86 ± 0.01 | 0.0086 ± 0.0008 | 8339.9 ± 0.5 | 0.0017 |
|  |  | Ni- N/O | 1.7 ± 0.1 | 2.06 ± 0.01 |  |  |  |
|  |  | Ni-Ni | 0.8 ± 0.1 | 2.82 ± 0.01 |  |  |  |
|  |  | Ni-Ni | 0.8 ± 0.1 | 3.11 ± 0.01 |  |  |  |
| **Tan-CN-NiFe-OCP** | Ni K-edge | Ni-N/O | 3.9 ± 0.6 | 1.87 ± 0.01 | 0.0094 ± 0.0018 | 8341.8 ± 1.0 | 0.0078 |
|  |  | Ni-N/O | 1.1 ± 0.3 | 2.17 ± 0.02 |  |  |  |
|  |  | Ni- Ni/Fe | 0.3 ± 0.2 | 2.74 ± 0.03 |  |  |  |
| **Tan-CN-NiFe-1.2 V** | Ni K-edge | Ni-N/O | 3.7 ± 0.5 | 1.85 ± 0.01 | 0.0084 ± 0.0017 | 8338.8 ± 1.0 | 0.0031 |
|  |  | Ni-N/O | 0.8 ± 0.3 | 2.13 ± 0.03 |  |  |  |
|  |  | Ni- Ni/Fe | 0.4 ± 0.1 | 2.73 ± 0.02 |  |  |  |
| **Tan-CN-NiFe-1.3 V** | Ni K-edge | Ni-N/O | 3.6 ± 0.6 | 1.86 ± 0.01 | 0.0086 ± 0.0021 | 8340.3 ± 1.3 | 0.0092 |
|  |  | Ni-N/O | 1.3 ± 0.3 | 2.11 ± 0.02 |  |  |  |
|  |  | Ni-Ni/Fe | 0.3 ± 0.2 | 2.73 ± 0.04 |  |  |  |
| **Tan-CN-NiFe-1.4 V** | Ni K-edge | Ni-N/O | 4.0 ± 0.5 | 1.86 ± 0.01 | 0.0102 ± 0.0016 | 8339.8 ± 0.9 | 0.0104 |
|  |  | Ni-N/O | 1.6 ± 0.3 | 2.10 ± 0.02 |  |  |  |
|  |  | Ni- Ni/Fe | 0.6 ± 0.2 | 2.75 ± 0.02 |  |  |  |
|  |  | Ni- Ni/Fe | 1.1 ± 0.2 | 3.07 ± 0.02 |  |  |  |
| **Tan-CN-NiFe-1.5 V** | Ni K-edge | Ni-N/O | 4.2 ± 0.5 | 1.86 ± 0.01 | 0.0111 ± 0.0014 | 8339.1 ± 0.8 | 0.0067 |
|  |  | Ni-N/O | 1.7 ± 0.2 | 2.10 ± 0.01 |  |  |  |
|  |  | Ni- Ni/Fe | 0.9 ± 0.2 | 2.76 ± 0.02 |  |  |  |
|  |  | Ni- Ni/Fe | 1.1 ± 0.2 | 3.08 ± 0.02 |  |  |  |
| **Tan-CN-NiFe-1.6 V** | Ni K-edge | Ni-N/O | 3.9 ± 0.5 | 1.86 ± 0.01 | 0.0103 ± 0.0014 | 8340.2 ± 0.8 | 0.0084 |
|  |  | Ni-N/O | 1.9 ± 0.2 | 2.09 ± 0.01 |  |  |  |
|  |  | Ni- Ni/Fe | 0.4 ± 0.2 | 2.78 ± 0.03 |  |  |  |
|  |  | Ni-Ni/Fe | 1.2 ± 0.2 | 3.10 ± 0.02 |  |  |  |

**Table S4.** Comparison of OER performance and stability of the as-prepared catalysts with recent representative SACs.

| **Name** | **Overpotential**  **(mV)*** | **Stability**  **(h)** | **Loading mass**  **(mg cm^−2^)** | **Reference** |
| --- | --- | --- | --- | --- |
| **Co_SAC_-W*_x_*C** | 371 | - | 0.38 | *J. Am. Chem. Soc.* 146, 9124−9133 (**2024**) |
| **Carbon based Co SAC** | 351 | 300 | 0.205 | *J. Am. Chem. Soc.* 145, 8052−8063(**2023**) |
| **Ir SAC on NiO** | 256 | 20 | - | *J. Am. Chem. Soc*. 146, 32953−32964(**2024**) |
| **Co_SA_-MoCeO_x_@BCT** | 239 | 60 | - | *Energy Environ. Sci*. 17, 3088-3098(**2024**) |
| **Co_SA_-MoCeO_x_** | 333 | - | - | *Energy Environ. Sci*. 17, 3088-3098(**2024**) |
| **Ni−N−C** | ~370 | 0.83 | - | *Angew. Chem. Int. Ed.* 64, e202413308(**2025**) |
| **Co/Fe-SNC800** | 240 | 28 | - | *Energy Environ. Sci.*16, 1685-1696 (**2024**) |
| **Fe_1_(OH)*_x_*/P-C** | 320 | 20 | 0.85 | *Nano Lett.* 21, 4795−4801(**2021**) |
| **Fe-N-C/Fe_2_C-op** | 349 | - | - | *https://doi.org/10.1002/advs.202301656* |
| **Tan-CN-CoFe** | 370 | 60 | 0.13 | This work |
| **Tan-CN-NiFe** | 320 | 47.6 | 0.13 | This work |

* The overpotential values are recorded at 10 mA cm^-2^.

**Table S5.** Peak deconvolution results for Co 2p XPS spectra

| **Catalyst** | **Peak Components** | **B.E. (eV)** | **FWHM (eV)** | **Area (%)** |
| --- | --- | --- | --- | --- |
| **Tan-CN-Fe** | Fe 2p_3/2_ | 711.0 | 5.0 | 29.6 |
|  | Fe 2p_1/2_ | 723.8 | 5.0 | 15.9 |
|  | Fe 2p_3/2_ shake-up | 715.8 | 9.9 | 35.0 |
|  | Fe 2p_1/2_ shake-up | 729.3 | 9.9 | 19.4 |
| **Tan-CN-Co** | Co 2p_3/2_ | 781.9 | 3.6 | 37 |
|  | Co 2p_1/2_ | 797.1 | 3.6 | 19 |
|  | Co 2p_3/2_ shake-up | 786.6 | 7.3 | 29 |
|  | Co 2p_1/2_ shake-up | 801.8 | 7.3 | 15 |
| **Tan-CN-Ni** | Ni 2p_3/2_ | 855.2 | 2.4 | 40.6 |
|  | Ni 2p_1/2_ | 872.5 | 2.4 | 17.4 |
|  | Ni 2p_3/2_ shake-up | 860.1 | 11.0 | 20.26 |
|  | Ni 2p_1/2_ shake-up | 878.1 | 11.0 | 21.7 |
| **Tan-CN-CoFe** | Co 2p_3/2_ | 781.7 | 3.9 | 43 |
|  | Co 2p_1/2_ | 796.9 | 3.9 | 21 |
|  | Co 2p_3/2_ shake-up | 786.6 | 7.3 | 24 |
|  | Co 2p_1/2_ shake-up | 801.8 | 7.3 | 12 |
| **Tan-CN-NiFe** | Ni 2p_3/2_ | 855.1 | 2.2 | 63.0 |
|  | Ni 2p_1/2_ | 872.3 | 2.4 | 25.3 |
|  | Ni 2p_3/2_ shake-up | 860.5 | 5.0 | 8.8 |
|  | Ni 2p_1/2_ shake-up | 876.7 | 5.0 | 3.5 |

**Table S6.** Crystal structure parameters for Fe, Co and Ni reference compounds (data retrieved from ICSD).

| **Composition** | **Space**  **Group** | **Crystal**  **System** | **Scattering**  **paths** | **Coordination**  **numbers** | **Interatomic**  **Distances** | **ICSD**  **Index** | **PDF**  **number** | **Ref.** |
| --- | --- | --- | --- | --- | --- | --- | --- | --- |
| α-Fe | $Im\bar{3}m$ | Cubic | Fe-Fe | 8 | 2.48 Å | 52258 | 01-071-3763 | ^[11]^ |
|  |  |  | Fe-Fe | 6 | 2.87 Å |  |  |  |
| FeO | $Fm\bar{3}m$ | Cubic | Fe-O | 6 | 2.06 | 82233 | 01-089-0687 | ^[12]^ |
|  |  |  | Fe-Fe | 12 | 3.06 |  |  |  |
| α-Fe_2_O_3_ | $R\bar{3}c$ | Trigonal | Fe-O | 3 | 1.94 | 40142 | 01-086-2368 | ^[13]^ |
|  |  |  | Fe-O | 3 | 2.11 |  |  |  |
|  |  |  | Fe-Fe | 1 | 2.89 |  |  |  |
|  |  |  | Fe-Fe | 3 | 2.97 |  |  |  |
|  |  |  | Fe-Fe | 3 | 3.36 |  |  |  |
| Fe(OH)_2_ | $P\bar{3}m1$ | Trigonal | Fe-O | 6 | 2.23 | 53992 | 01-073-6991 | ^[14]^ |
|  |  |  | Fe-Fe | 6 | 3.25 |  |  |  |
| Fe(OH)_3_ *x*H_2_O | $Immm$ | Orthorhombic | Fe-O(1) | 2 | 1.99 | 73441 | 01-081-2022 | ^[15]^ |
|  |  |  | Fe-O(2) | 2 | 2.01 |  |  |  |
|  |  |  | Fe-O(3) | 2 | 2.03 |  |  |  |
|  |  |  | Fe-Fe | 2 | 3.77 |  |  |  |
|  |  |  | Fe-Fe | 4 | 3.78 |  |  |  |
| α-FeO(OH) | $Pbnm$ | Orthorhombic | Fe-O(1) | 2 | 1.94 | 239324 | 01-084-8280 | ^[16]^ |
|  |  |  | Fe-O(1) | 1 | 2.01 |  |  |  |
|  |  |  | Fe-O(2) | 1 | 2.07 |  |  |  |
|  |  |  | Fe-O(2) | 2 | 2.11 |  |  |  |
| δ-FeO(OH) | $P\bar{3}m1$ | Trigonal | Fe(1)-O | 6 | 2.04 | 38299 | 01-077-0247 | ^[17]^ |
|  |  |  | Fe(2)-O | 6 | 2.06 |  |  |  |
|  |  |  | Fe(1)-Fe(1) | 3 | 2.95 |  |  |  |
|  |  |  | Fe(2)-Fe(2) | 3 | 2.95 |  |  |  |
| β-FeO(OH) | $I4/m$ | Tetragonal | Fe-O(1) | 2 | 2.01 | 31136 | 01-075-1594 | ^[18]^ |
|  |  |  | Fe-O(1) | 1 | 2.03 |  |  |  |
|  |  |  | Fe-O(2) | 1 | 2.05 |  |  |  |
|  |  |  | Fe-O(2) | 2 | 2.20 |  |  |  |
|  |  |  | Fe-Fe | 2 | 3.02 |  |  |  |
|  |  |  | Fe-Fe | 2 | 3.09 |  |  |  |
| γ-FeO(OH) | $Cmcm$ | Orthorhombic | Fe-O(1) | 2 | 1.97 | 24885 | 01-073-2326 | ^[19]^ |
|  |  |  | Fe-O(2) | 2 | 1.98 |  |  |  |
|  |  |  | Fe-O(1) | 2 | 2.13 |  |  |  |
|  |  |  | Fe-Fe | 2 | 3.06 |  |  |  |
|  |  |  | Fe-Fe | 4 | 3.10 |  |  |  |
| Co | $Fm\bar{3}m$ | Cubic | Co-Co | 12 | 2.51 Å | 76632 | 01-089-7093 | ^[20]^ |
| Co | $P6_{3}/mmc$ | Cubic | Co-Co | 6 | 2.50 Å | 76633 | 01-089-7094 | ^[20]^ |
|  |  |  | Co-Co | 6 | 2.51 Å |  |  |  |
| CoO | $Fm\bar{3}m$ | Cubic | Co-O | 6 | 2.13 Å | 9865 | 01-071-1178 | ^[21]^ |
|  |  |  | Co-Co | 12 | 3.01 Å |  |  |  |
| Co_3_O_4_ | $Fd\bar{3}m$ | Cubic | Co(1)-O | 6 | 1.91 Å | 36256 | 01-076-1802 | ^[22]^ |
|  |  |  | Co(2)-O | 4 | 1.94 Å |  |  |  |
|  |  |  | Co(1)-Co(1) | 6 | 2.85 Å |  |  |  |
|  |  |  | Co(1)-Co(2) | 6 | 3.35 Å |  |  |  |
|  |  |  | Co(2)-Co(1) | 12 | 3.35 Å |  |  |  |
|  |  |  | Co(2)-Co(2) | 4 | 3.50 Å |  |  |  |
| β-Co(OH)_2_ | $P\bar{3}m1$ | Trigonal | Co-O | 6 | 2.12 Å | 88940 | 01-089-8616 | ^[23]^ |
|  |  |  | Co-Co | 6 | 3.19 Å |  |  |  |
| CoO(OH) | $R\bar{3}m$ | Trigonal | Co-O | 6 | 1.90 Å | 22285 | 01-073-0497 | ^[24]^ |
|  |  |  | Co-Co | 6 | 2.85 Å |  |  |  |
| Ni | $Fm\bar{3}m$ | Cubic | Ni-Ni | 12 | 2.49 Å | 52231 | 01-071-3740 | ^[25]^ |
| NiO | $Fm\bar{3}m$ | Cubic | Ni-O | 6 | 2.09 Å | 9866 | 01-071-1179 | ^[21]^ |
|  |  |  | Ni-Ni | 12 | 2.95 Å |  |  |  |
| β-Ni(OH)_2_ | $P\bar{3}m1$ | Trigonal | Ni-O | 6 | 2.06 Å | 169978 | 01-080-2855 | ^[26]^ |
|  |  |  | Ni-Ni | 6 | 3.10 Å |  |  |  |
| NiO(OH) | $C2/m$ | Monoclinic | Ni-O(1) | 3 | 1.91 Å | 230448 | 01-086-8225 | ^[27]^ |
|  |  |  | Ni-O(2) | 1 | 1.98 Å |  |  |  |
|  |  |  | Ni-O(2) | 2 | 2.12 Å |  |  |  |
|  |  |  | Ni-Ni | 4 | 2.84 Å |  |  |  |
|  |  |  | Ni-Ni | 2 | 2.91 Å |  |  |  |

**Table S7.** Crystal structure parameters for Fe, Co and Ni reference compounds (data retrieved from CCDC).

| **Composition** | **Space**  **Group** | **Crystal**  **System** | **Scattering**  **paths** | **Coordination**  **numbers** | **Interatomic**  **Distances** | **CCDC**  **Entry ID** | **Ref.** |
| --- | --- | --- | --- | --- | --- | --- | --- |
| C_32_H_16_FeN_8_ (FePc) | $P2_{1}/n$ | Monoclinic | Fe-N | 4 | 1.93 Å | 1482844 | ^[28]^ |
|  |  |  | Fe-C | 4 | 2.95 Å |  |  |
|  |  |  | Fe-C | 4 | 2.97 Å |  |  |
| C_32_H_16_CoN_8_ (FePc) | $P2_{1}/n$ | Monoclinic | Co-N | 4 | 1.92 Å | 1129688 | ^[29]^ |
|  |  |  | Co-C | 4 | 2.93 Å |  |  |
|  |  |  | Co-C | 4 | 2.96 Å |  |  |
| C_32_H_16_CoN_8_ (FePc) | $P2_{1}/n$ | Monoclinic | Ni-N | 4 | 1.90 Å | 991814 | ^[30]^ |
|  |  |  | Ni-C | 4 | 2.92 Å |  |  |
|  |  |  | Ni-C | 4 | 2.95 Å |  |  |

**Table S8.** ICP-MS measurements of the electrolyte for all catalysts before and after OER.

|  | **Fe (ppm)** | **Co (ppm)** | **Ni (ppm)** |
| --- | --- | --- | --- |
| **Commercial 1M KOH** | < 0.7 | < 0.3 | 3.4 |
| **Tan-CN-Fe** | < 0.8 | < 0.3 | 4.3 |
| **Tan-CN-Co** | < 0.8 | < 1.0 | 3.9 |
| **Tan-CN-Ni** | < 0.8 | < 0.3 | 3.4 |
| **Tan-CN-NiFe** | < 0.7 | 0.51 | 3.6 |
| **Tan-CN-CoFe** | < 0.8 | 0.46 | 3.8 |

# 7. References

[1] J. Yin, J. Jin, M. Lu, B. Huang, H. Zhang, Y. Peng, P. Xi, C.-H. Yan, *J. Am. Chem. Soc.* **2020**, *142*, 18378–18386.

[2] J. J. Velasco-Vélez, V. Pfeifer, M. Hävecker, R. Wang, A. Centeno, A. Zurutuza, G. Algara-Siller, E. Stotz, K. Skorupska, D. Teschner, P. Kube, P. Braeuninger-Weimer, S. Hofmann, R. Schlögl, A. Knop-Gericke, *Rev. Sci. Instrum.* **2016**, *87*, 053121.

[3] E. Welter, *J. Synchrotron Radiat.* **2012**, *19*, 905–910.

[4] E. Welter, R. Chernikov, M. Herrmann, R. Nemausat, Taipei, Taiwan, **2019**, p. 040002.

[5] S. Belin, V. Briois, A. Traverse, M. Idir, T. Moreno, M. Ribbens, *Phys. Scr.* **2005**, 980.

[6] E. Fonda, A. Rochet, M. Ribbens, L. Barthe, S. Belin, V. Briois, *J. Synchrotron Radiat.* **2012**, *19*, 417–424.

[7] B. Ravel, M. Newville, *J. Synchrotron Radiat.* **2005**, *12*, 537–541.

[8] M. Muñoz, P. Argoul, F. Farges, *Am. Mineral.* **2003**, *88*, 694–700.

[9] M. Newville, *J. Phys. Conf. Ser.* **2013**, *430*, 012007.

[10] T. Matsumoto, K. Nakada, T. Matsushita, S. Yokota, Y. Furukawa, A. Yamashita, M. Kodera, *AIP Conf. Proc.* **2019**, 2054, 060076.

[11] E. A. Owen, E. L. Yates, *Lond. Edinb. Dublin Philos. Mag. J. Sci.* **1933**, *15*, 472–488.

[12] H. Fjellvåg, F. Grønvold, S. Stølen, B. Hauback, *J. Solid State Chem.* **1996**, *124*, 52–57.

[13] E. N. Maslen, V. A. Streltsov, N. R. Streltsova, N. Ishizawa, *Acta Crystallogr. B* **1994**, *50*, 435–441.

[14] A. nazionale dei Lincei, matematiche Classe di scienze fisiche, naturali, *Atti Della R. Accademia Nazionale Dei Lincei. Memorie Della Classe Di Scienze Fisiche, Matematiche e Naturali*, R. Accademia Nazionale Dei Lincei, **1927**.

[15] W. D. Birch, A. Pring, A. Reller, H. W. Schmalle, *Am. Mineral.* **1993**, *78*, 827–834.

[16] E. Zepeda-Alarcon, H. Nakotte, A. F. Gualtieri, G. King, K. Page, S. C. Vogel, H.-W. Wang, H.-R. Wenk, *J. Appl. Crystallogr.* **2014**, *47*, 1983–1991.

[17] G. Patrat, F. De Bergevin, M. Pernet, J. C. Joubert, *Acta Crystallogr. B* **1983**, *39*, 165–170.

[18] A. L. Mackay, *Mineral. Mag. J. Mineral. Soc.* **1960**, *32*, 545–557.

[19] A. Oleś, A. Szytuła, A. Wanic, *Phys. Status Solidi B* **1970**, *41*, 173–177.

[20] A. Taylor, R. W. Floyd, *Acta Crystallogr.* **1950**, *3*, 285–289.

[21] S. Sasaki, K. Fujino, Y. Takéuchi, *Proc. Jpn. Acad. Ser. B* **1979**, *55*, 43–48.

[22] J. P. Picard, G. Baud, J. P. Besse, R. Chevalier, *J. Common Met.* **1980**, *75*, 99–104.

[23] F. Pertlik, *Monatshefte Für Chem. Chem. Mon.* **1999**, *130*, 1083–1088.

[24] R. G. Delaplane, J. A. Ibers, J. R. Ferraro, J. J. Rush, *J. Chem. Phys.* **1969**, *50*, 1920–1927.

[25] E. A. Owen, E. L. Yates, *Lond. Edinb. Dublin Philos. Mag. J. Sci.* **1936**, *21*, 809–819.

[26] V. Yu. Kazimirov, M. B. Smirnov, L. Bourgeois, L. Guerlou-Demourgues, L. Servant, A. M. Balagurov, I. Natkaniec, N. R. Khasanova, E. V. Antipov, *Solid State Ion.* **2010**, *181*, 1764–1770.

[27] M. Casas-Cabanas, M. D. Radin, J. Kim, C. P. Grey, A. Van Der Ven, M. R. Palacín, *J. Mater. Chem. A* **2018**, *6*, 19256–19265.

[28] L. A. Rochford, D. S. Keeble, O. J. Holmes, G. J. Clarkson, T. S. Jones, *J Mater Chem C* **2014**, *2*, 6056–6060.

[29] P. A. Reynolds, B. N. Figgis, E. S. Kucharski, S. A. Mason, *Acta Crystallogr. B* **1991**, *47*, 899–904.

[30] Y. Takahashi, K. Hayakawa, K. Takayama, S. Yokokura, J. Harada, H. Hasegawa, T. Inabe, *Chem. Mater.* **2014**, *26*, 993–998.
